# Bioinspired synthesis of pentacyclic onocerane triterpenoids†
†Electronic supplementary information (ESI) available: Detailed experimental procedures, spectral data, DFT calculation, X-ray crystallographic data for **4** (CIF), **13** (CIF), **10** (CIF), **22** (CIF). CCDC 1529115–1529118. For ESI and crystallographic data in CIF or other electronic format see DOI: 10.1039/c7sc03903d


**DOI:** 10.1039/c7sc03903d

**Published:** 2017-10-16

**Authors:** Florian Bartels, Young J. Hong, Daijiro Ueda, Manuela Weber, Tsutomu Sato, Dean J. Tantillo, Mathias Christmann

**Affiliations:** a Institute of Chemistry and Biochemistry , Freie Universität Berlin , Takustraße 3 , 14195 Berlin , Germany . Email: mathias.christmann@fu-berlin.de; b Department of Chemistry , University of California–Davis , Davis , California 95616 , USA . Email: djtantillo@ucdavis.edu; c Department of Applied Biological Chemistry , Graduate School of Science and Technology , Niigata University , Ikarashi 2-8050, Nishi-ku , Niigata 950-2181 , Japan . Email: satot@agr.niigata-u.ac.jp

## Abstract

The first chemical synthesis of pentacyclic onocerane triterpenoids has been achieved. A putative biomimetic tricyclization cascade is employed to forge a fused decalin-/oxepane ring system. The synthetic route proceeds to (+)-cupacinoxepin in seven steps and to (+)-onoceranoxide in eight steps in the longest linear sequence, when starting from geranyl chloride and (+)-sclareolide. The bioinspired epoxypolyene cyclization is supported by computational and enzymatic studies.

## Abstract 2

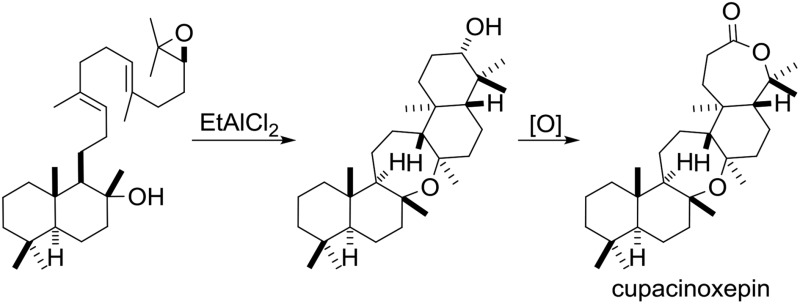
The first chemical synthesis of pentacyclic onocerane triterpenoids (+)-cupacinoxepin and (+)-onoceranoxide is described.

## 

Triterpenoids constitute an important family of diverse natural products with unique biological activities.^1^ Their structural complexity is generated by cyclase enzymes that convert simple acyclic isoprenoid precursors into polycyclic molecules.^2^,^3^ For example, onocerane triterpenes were shown to be biosynthesized from squalene (**1**) or its oxidized derivatives (**2**, **3**) by cyclizations initiated at both termini.^4^ The intermediates and products can be distinguished by their oxidation level (OL), (carbo-)cyclization level (CL), and the hydration level (HL), *i.e.* the number of incorporated water molecules. Following core assembly, functional group modifications (FGMs) or C–H-oxidations^5^ may occur (Scheme 1). In a bioassay-guided screening for anti-malarial compounds, Schuehly and coworkers reported the isolation of a novel triterpenoid from the bark of *Cupania cinerea*.^6^ Cupacinoxepin (**4**) showed moderate activity against the *Plasmodium falciparum* K1 strain (8.7 μM) and features a novel fused pentacyclic onocerane scaffold composed of three six-membered carbocycles and two oxepanes. Although data from a single crystal suitable for X-ray crystallography was obtained, the absolute configuration could not be determined.^6^ Previous synthetic work on the onceranes^7^–^10^ was focused on the tetracyclic *C*_2_-symmetrical congeners onocerandiol (**5**)^11^–^14^ and α-onocerin (**6**).^15^–^22^ The biosynthesis of onoceranoxide (**7**)^23^ and α-onocerin (**6**) *via***8**^24^,^25^ was hypothesized to proceed *via* cyclization of squalene and diepoxysqualene, respectively (Scheme 1). This process was elegantly mimicked by Corey's double allylsilane epoxypolyene cyclization.^16^ However, the highly substituted oxepane in cupacinoxepin (**4**) and onoceranoxide (**7**)^26^,^27^ required a novel synthetic strategy.

**Scheme 1:**
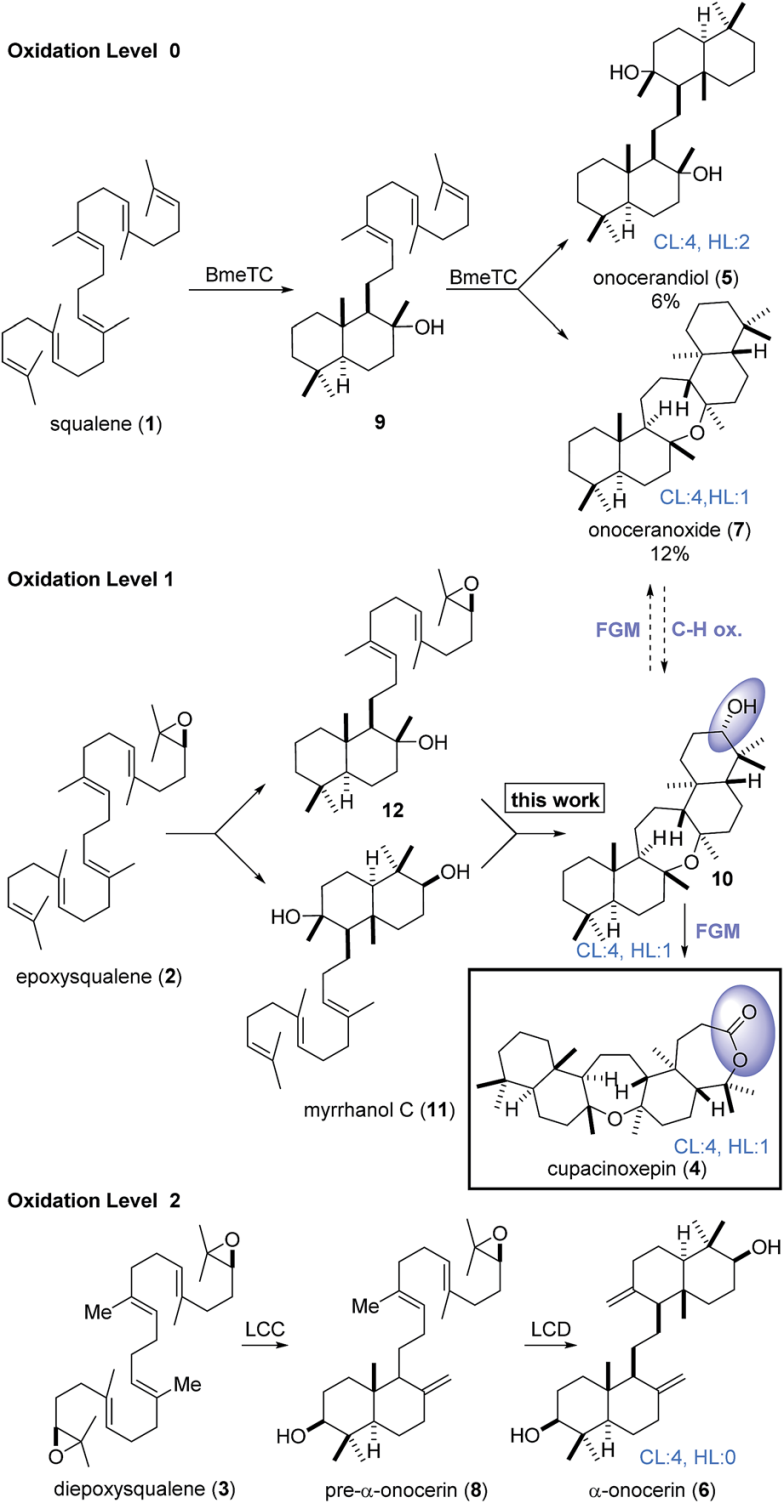
Core assembly of onocerane triterpenes with regard to the oxidation level (OL), cyclization level (CL) and the hydration level (HL). LCC and LCD = *Lycopodium clavatum* C and D respectively.

Chemical synthesis of ditertiary ethers is a daunting task.^28^–^38^ Unfortunately, the seemingly obvious approach to form the oxepane from two tertiary alcohols is outpaced by competing cationic pathways.^13^,^14^ Inspired by the cyclization of squalene to **7***via***9** catalyzed by *Bacillus megaterium* tetraprenyl-β-curcumene cyclase (BmeTC),^23^ we identified myrrhanol C (**11**)^39^ or epoxy dienol **12** as potential precursors for the synthesis of **10**. While the actual biosynthetic pathway is unknown, the realization of an epoxydiene tricyclization appeared more feasible in a laboratory setting. Finally, oxidation of the secondary alcohol **10** to cupacinoxepin (**4**) (*via* the intermediacy of a ketone) completes the putative biosynthesis. Only a few examples of polyene cyclizations^40^–^45^ using tertiary alcohols as nucleophiles are known,^46^–^48^ and to the best of our knowledge polyene tricyclizations using tertiary alcohols to form oxepanes have only been achieved enzymatically so far.^23^,^49^ In order to probe this key cyclization (**12** → **10**) in the laboratory, we selected epoxy dienol **12** as our retrosynthetic target (Scheme 2).

**Scheme 2:**
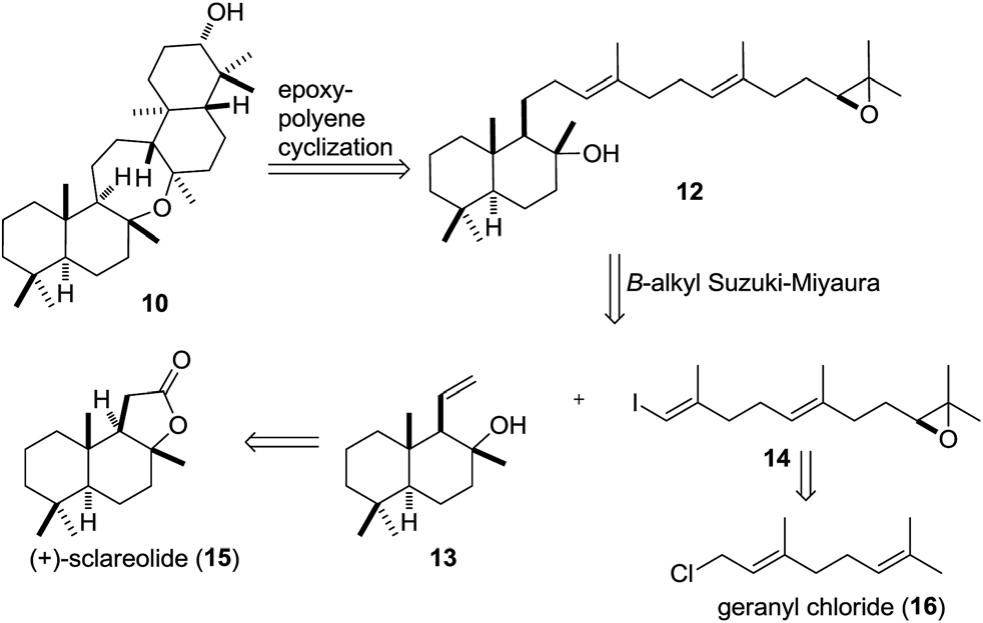
Retrosynthetic analysis of **10** based on an epoxypolyene cyclization of precursor **12**.

We envisioned the cyclization precursor **12** to be generated in a *B*-alkyl Suzuki–Miyaura coupling between an alkylborane derived from **13** and vinyl iodide **14**.^50^ The two fragments were traced back to the readily available starting materials (+)-sclareolide (**15**) and geranyl chloride (**16**), respectively.^51^–^53^

The synthesis of fragment **13**^54^ started with the conversion of **15** into the corresponding benzyl ether **17** using a one-pot^55^,^56^ reduction/alkylation sequence (Scheme 3). To this end, reduction of (+)-sclareolide with LiAlH_4_ in THF at 0 °C 57 was followed by treatment with Rochelle salt, DMF, KOH and 2-Me-C_6_H_4_CH_2_Br to afford benzyl ether **17** in excellent yield. A [2,3]-Wittig-type fragmentation mediated by *n*-BuLi directly yielded fragment **13** in an acceptable yield of 44% over two steps.^58^,^59^ The subtle deviation of the ether group from the literature-known benzyl ether to its 2-Me-benzyl derivative increased the yield from 21% to 44%. A screening of different ether derivatives showed alkyl substituents with benzylic hydrogen atoms in the 2-position of the aromatic ring to give higher yields of **13**. Moreover, we observed a temperature dependence of the fragmentation yield, maximizing at –13 °C.^46^ The configuration of **13** was confirmed by X-ray crystallography.^60^

**Scheme 3:**
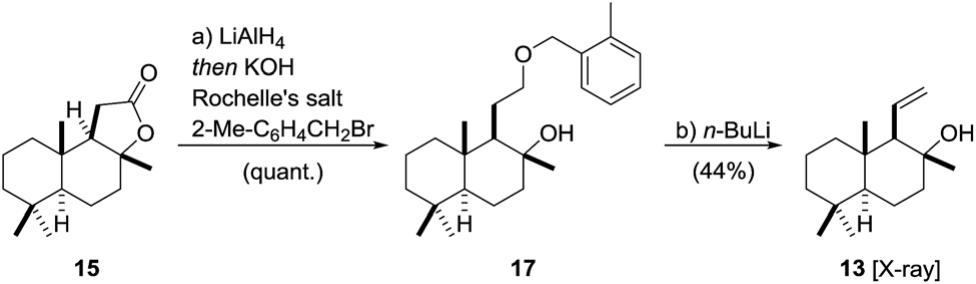
Synthesis of fragment **13**. Reagents and conditions: (a) LiAlH_4_ (0.7 eq.), THF, 0 °C, 40 min then 2-Me-C_6_H_4_CH_2_Br (2.1 eq.), KOH (4.0 eq.), Rochelle's salt (1.2 eq.), DMF, 45 °C, 27 h, quant.; (b) *n*-BuLi (4.0 eq.), THF, –78 °C, 10 min to –13 °C, 90 min, 44% (over 2 steps).

Vinyl iodide **14** was prepared from geranyl chloride *via* nucleophilic substitution with lithiated **18**^61^ followed by desilylation with TBAF (Scheme 4). Negishi's zirconium-catalyzed carboalumination^62^,^63^ of **19** with AlMe_3_ and subsequent trapping of the vinyl aluminium intermediate with iodine afforded vinyl iodide **20**.^64^,^65^ Dihydroxylation of the dimethyl-substituted alkene with the (DHQD)_2_PHAL ligand^66^ (33% yield, 97% ee) proceeded with low position-selectivity which is reflected by the small amount of recovered starting material (25%). Using the Corey–Noe–Lin (CNL) ligand^67^ increased the position-selectivity to give the terminal diol in 36% yield (94% ee) with 49% of recovered alkene.^68^ Mesylation of the secondary alcohol and subsequent treatment with K_2_CO_3_ afforded epoxide **14** in 81% yield.^69^ With alkene **13** and vinyl iodide **14** in hand, the crucial coupling was investigated (Scheme 5). Treatment of alkene **13** (neat) with 9-BBN dimer at 85 °C for 4 h led to the expected borane, which was directly used in the *B*-alkyl Suzuki–Miyaura reaction^69^–^71^ to afford epoxy dienol **12** in 77% yield on gram scale. The stage was then set to examine the putative biomimetic tricyclization.^72^ We anticipated the formation of the oxepane to be unfavorable both for entropic and enthalpic reasons.^73^ A variety of Brønsted and Lewis acids^46^ failed to give the desired product, but gratifyingly, treatment of **12** with EtAlCl_2_ at –78 °C under high dilution (CH_2_Cl_2_, 1 mM) afforded a separable mixture of target compound **10** (20%) along with another pentacyclic product **21** (12%).^74^–^76^ Spirocyclic motifs similar to **21** have been found in several bioactive natural products.^77^–^80^ Further conditions for the tricyclization of **9** were investigated but no product formation could be observed.^46^

**Scheme 4:**
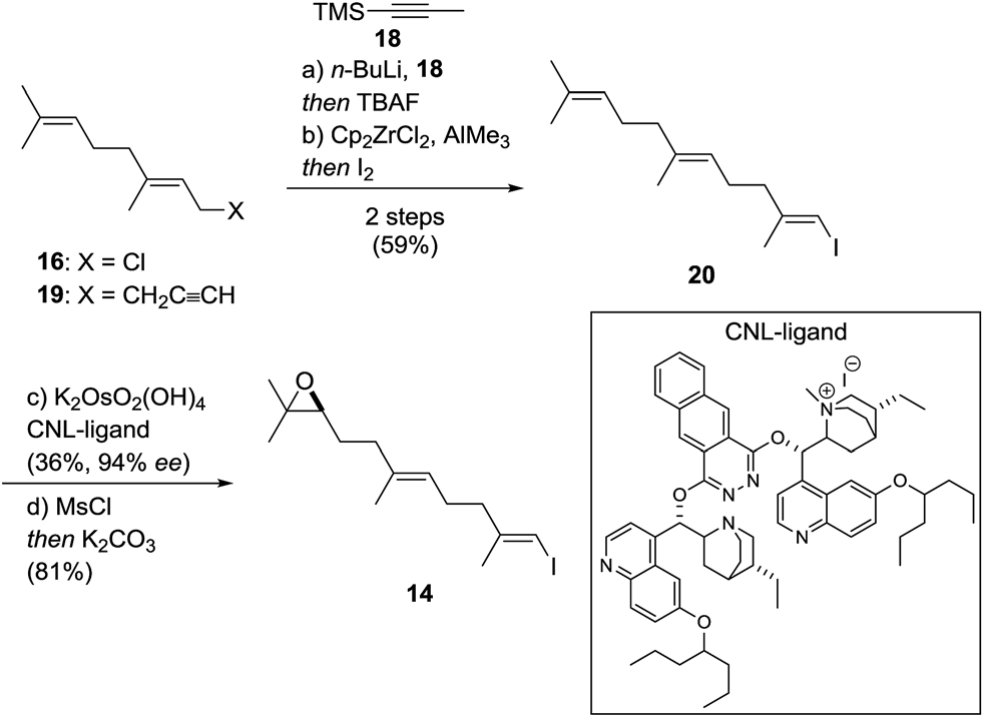
Synthesis of fragment **14**. Reagents and conditions: (a) *n*-BuLi (1.2 eq.), **18** (1.2 eq.), THF, –78 °C, 2.5 h then TBAF (1.3 eq.), –78 °C to 23 °C, 24 h, 82%; (b) Cp_2_ZrCl_2_ (0.25 eq.), AlMe_3_ (3.0 eq.), H_2_O (1.0 eq.), CH_2_Cl_2_, –23 °C, 1 h then I_2_ (1.2 eq.), THF, –23 °C to 23 °C, 16 h, 72%; (c) K_2_OsO_2_(OH)_4_ (0.3 mol%), CNL-ligand (0.2 mol%), K_3_Fe(CN)_6_ (3.0 eq.), MeSO_2_NH_2_ (1.0 eq.), K_2_CO_3_ (3.0 eq.), *t*-BuOH, H_2_O, 1 °C, 53 h, 36%, 94% ee, and 49% of **20**; (d) MsCl (1.1 eq.), pyridine (15 eq.), CH_2_Cl_2_, 0 °C to 23 °C, 18 h then K_2_CO_3_ (10 eq.), MeOH, 2.5 h, 81%.

**Scheme 5:**
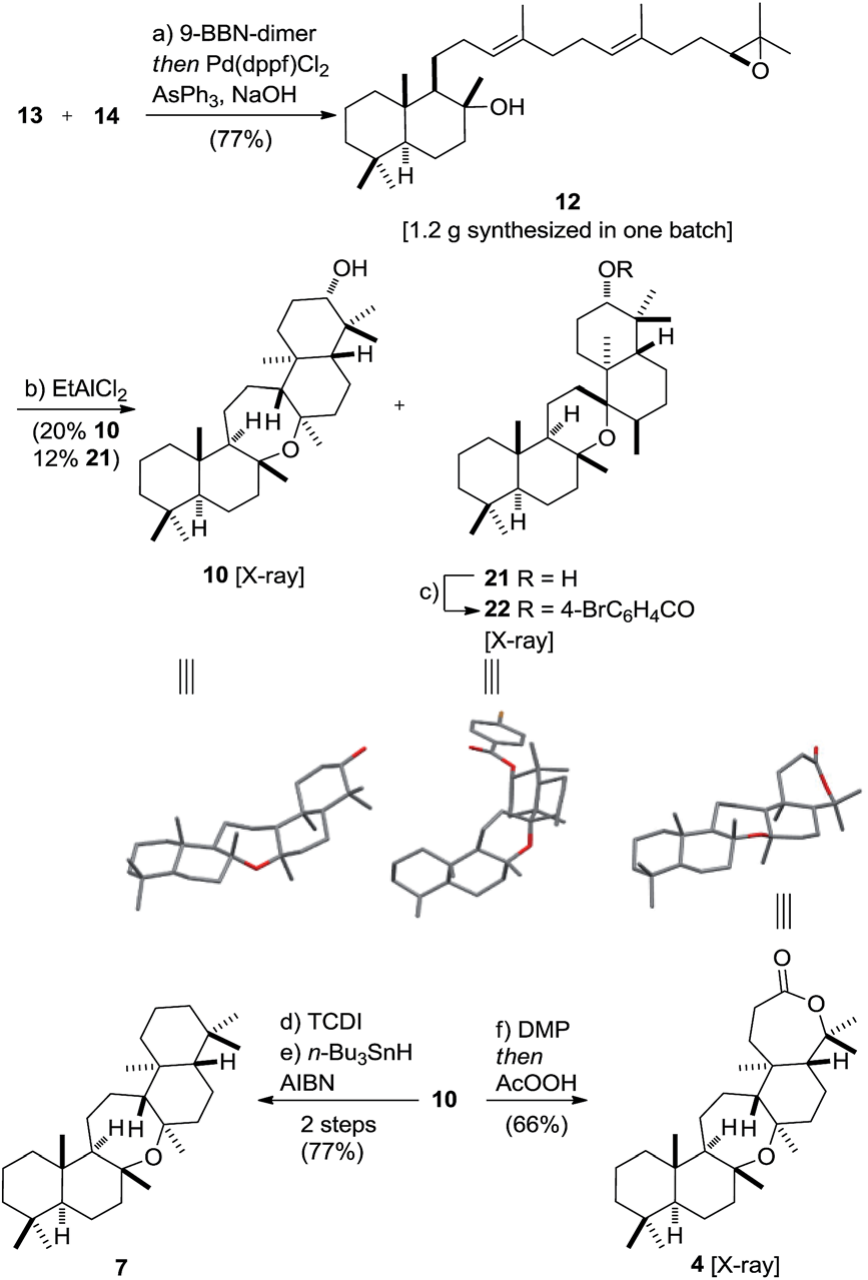
Synthesis of cupacinoxepin **4** and onoceranoxide **7**. Reagents and conditions: (a) **13** (1.4 eq.), 9-BBN dimer (2.8 eq.), 85 °C, 4 h then **14** (1.0 eq.), Pd(dppf)Cl_2_ (0.1 eq.), AsPh_3_ (0.4 eq.), NaOH (6.0 eq.), 1 °C, 17 h, 77%; (b) EtAlCl_2_ (3.0 eq.), CH_2_Cl_2_ (1 mM), –78 °C, 1.5 h, 20% **10**, 12% **21**; (c) 4-BrC_6_H_4_COCl (5.0 eq.), 4-DMAP (20 eq.), 50 °C, CH_2_Cl_2_, 3 d, 73%; (d) TCDI (20 eq.), 4-DMAP (20 eq.), CH_2_Cl_2_, 70 °C 13.5 h, 83%; (e) *n*-Bu_3_SnH (3.0 eq.), AIBN (cat.), toluene, 160 °C, 10 min to 120 °C, 20 min then *n*-Bu_3_SnH (3.0 eq.), AIBN (cat.), 160 °C, 10 min to 120 °C, 20 min, 93%; (f) DMP (2.0 eq.), CH_2_Cl_2_, 23 °C, 4 h then AcOOH (10 eq.), NaOAc (20 eq.), 17 h then AcOOH (10 eq.), NaOAc (20 eq.), 5 h, 66%. DMP = Dess–Martin periodinane, TCDI = 1,1′-thiocarbonyldiimidazole, AIBN = azobisisobutyronitrile.

The structures of oxepane **10** and *p*-Br-benzoate derivative **22** were confirmed by X-ray crystallography. Despite its rather low yield, the cyclization generated four stereogenic centers and the remaining carbon skeleton in a single transformation. Additionally, the secondary alcohol in **10** might serve as a handle in future SAR studies and enable the evaluation of this class of pentacyclic onocerane triterpenoids as potential antimalarial drug leads.^81^,^82^ In addition, recent work in the field of C–H functionalization has demonstrated the sclareolide scaffold to be amenable for the selective introduction of other functional groups.^83^–^90^ Next, the major cyclization product **10** was subjected to a one-pot Dess–Martin/Baeyer–Villiger oxidation^91^–^94^ to afford cupacinoxepin in 66% yield (1.7% overall yield starting from **16**). The spectroscopic data, including the optical rotation, matched those reported in the literature, thereby determining the absolute configuration of (+)-cupacinoxepin.^6^ In addition, we were able to obtain a crystal suitable for the direct determination of the absolute configuration of **4** by X-ray crystallography. Onoceranoxide **7** was formed from **10***via* formation of the thiocarbamate and subsequent reduction with tributyltin hydride in 77% yield (2 steps) (2.0% overall yield starting from **16**).^95^ The spectroscopic data matched those reported in the literature.^26^

In order to investigate the mechanism of the epoxypolyene cyclization in more detail and to get insight into the low selectivity for the desired *trans*–*anti*–*trans* pathway, well established density functional theory (DFT) methods were applied (mPW1PW91/6-31+G(d,p)//B3LYP/6-31+G(d)).^46^ Two epimeric *trans*-decalin-type structures (*S*-epimer **23** and *R*-epimer **24**, Scheme 6) were predicted, based on inherent reactivity preferences (*i.e.*, in the absence of solvent or enzyme),^96^ to result from epoxydiene cyclization, consistent with previous work by Corey and Shenvi on related systems.^75^,^97^ The predicted major intermediate **23** is derived from a chair–chair conformation, while **24** is derived from a chair–boat conformation.^75^,^80^

**Scheme 6:**
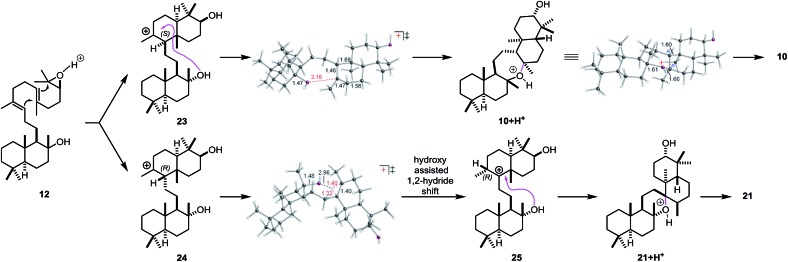
Carbocation rearrangements leading to **10** and **21**, modeled with H^+^ in place of Lewis acid (LA).

For **23**, 7-membered ring formation was predicted to be preferred over other pathways by several kcal mol^–1^. For **24**, 1,2-hydride shift to form **25**/6-membered ring formation was predicted to be preferred over formation of a 7-membered ring by several kcal mol^–1^. As shown in the transition state structure for formation of **25**, the hydride shift appears to be assisted by the tertiary hydroxyl group (*via* a favorable electrostatic interaction between the partially negatively charged oxygen and the partially positively charged migrating hydrogen; related interactions have been described previously).^98^,^99^ In addition, the biosynthetic relevance of the epoxypolyene cyclization was probed by incubation of **12** with BmeTC. GC-MS analysis revealed the formation of **10** along with an elimination product.^46^ The absence of **21** indicates the influence of BmeTC in overriding inherent reactivity and enforcing the chair–chair conformation leading to **10**.

In conclusion, we have provided access to a new class of pentacyclic onocerane triterpenoids. Additionally, we have completed the first asymmetric synthesis of antiprotozoal agent (+)-cupacinoxepin and (+)-onoceranoxide and determined their absolute configuration. By using an epoxypolyene cascade tricyclization as the key step, we were able to rapidly assemble the fused pentacyclic structure in a single synthetic operation. The putative biosynthetic precursor was assembled from two terpene derived fragments using a *B*-alkyl Suzuki–Miyaura reaction. Our synthetic route to cupacinoxepin consists of seven steps from geranyl chloride, four of which are C–C bond formations.

## Conflicts of interest

There are no conflicts to declare.

## Supplementary Material

Supplementary information

Crystal structure data
