# Supplementary material for: Bioinspired synthesis of pentacyclic onocerane triterpenoids
Source: Chem Sci. 2017 Oct 16;8(12):8285–90. doi: 10.1039/c7sc03903d (PMC5858022; doi:10.1039/c7sc03903d)
Supplement: Supplementary file 1 [file SC-008-C7SC03903D-s001.pdf]

Electronic Supplementary Material (ESI) for Chemical Science.
This journal is © The Royal Society of Chemistry 2017

Supporting Information

Bioinspired Synthesis of Pentacyclic Onocerane Triterpenoids

Florian Bartels, Young J. Hong, Daijiro Ueda, Manuela Weber, Tsutomu Sato,* Dean J. Tantillo,* and Mathias Christmann*

List of contents

1. General Methods 3
2. Synthesis of Compounds...4
3. Screening of Reaction Conditions 57
4. NMR Spectra...63
5. Literature Comparison: Polyene Cyclizations terminated by Aliphatic Alcohols 108
6. Enzymatic Studies 110
7. Density Functional Calculations 114
8. Crystallographic Data 241
9. References...242

1. General Methods

All reactions sensitive to moisture and/or air were carried out using heat-gun dried (630 °C) glassware under an argon atmosphere. Dry solvents (CH_2Cl_2, Et_2O, THF) were purified by Solvent Purification System M-BRAUN Glovebox Technology SPS-800. Dry DMF was obtained from Acros Organics 99.8%, extra dry over molecular sieves. Dry pyridine was obtained from Sigma Aldrich, anhydrous, 99.8%. Dry $MeNO_2$ was obtained by distillation over CaH_2. Dry 1,2-dimethoxyethane was obtained by distillation over CaH_2. Dry TMEDA was obtained by distillation over CaH_2. Dry methanol (HiPerSolv CHROMANORM HPLC GRADE) was obtained from VWR Chemicals. Solvents for column chromatography were used after short path distillation using a rotary evaporator. Commercial reagents were used as received unless otherwise stated. All reactions were carried out under magnetic stirring with Teflon coated stirring bars and were monitored by TLC analysis on 0.20 mm silica gel plates (Macherey-Nagel G/UV254). Staining of TLC plates was performed with an acidic vanillin solution (1 g vanillin, 20 mL conc. acetic acid, 10 mL conc. sulfuric acid, 170 mL methanol) and heat. Column chromatography was carried out on silica gel 60 M (0.04–0.063 mm) from Macherey-Nagel and aluminium oxide (basic, Brockmann I, for chromatography, 50-200 µm, 60A) from ACROS Organics. Concentration under reduced pressure was performed by rotary evaporation at 40 °C. ^1H NMR and ^{13}C NMR spectra were recorded on Bruker (ECP 400, AC 500, AV 700) or JEOL (ECX 400, Eclipse 500) instruments. Chemical shifts are reported relative to $CDCl_3$ (^1H: 7.26 ppm; ^{13}C: 77.16 ppm) and C_6D_6 (^1H: 7.16 ppm; ^{13}C: 128.06 ppm). Chemical shifts are reported in parts per million as follows: chemical shift, multiplicity (s = singlet, d = doublet, t = triplet, q = quartet, p = pentet, sext = sextet, m = multiplet, $_{br}$ = broad, and combinations thereof), coupling constant and integration. Integrals are in accordance with assignments, coupling constants are given in Hz. For detailed peak assignments, 2D spectra were recorded when necessary (COSY, DEPT, HMQC, HMBC, TOCSY, GOESY, NOESY). IR spectra were measured on a JASCO FT/IR–4100 instrument equipped with an ATR unit. High resolution ESI analyses were performed on a Varian Inc. Ionspec QFT-7. High resolution EI spectra were performed on a Waters Autospec Premier. Optical rotation measurements were performed on a P-2000 polarimeter from Jasco in a 10 cm optical-path length cell with the frequency of the Na*D* line measured at the temperature and concentration (in g/100 mL) indicated. The enantiomeric excess was determined by chiral HPLC using Agilent Technologies 1200 series with a diode array detector. Melting points were measured with a Stuart melting point apparatus SMP30 and on a Reichert Thermovar Kofler hot-stage-microscope and are uncorrected.

2. Synthesis of Compounds

Compound **17**

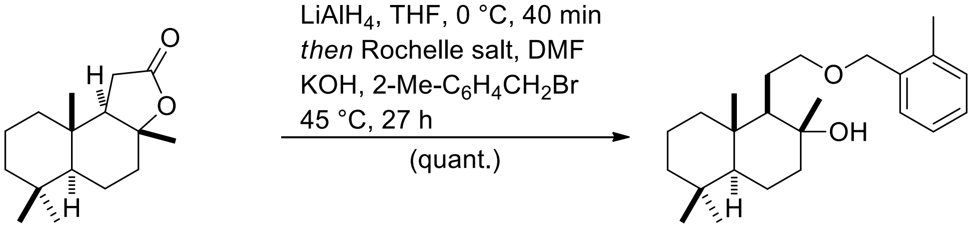

15 $C_{16}H_{26}O_2$ (250.38)

17 $C_{24}H_{38}O_2$ (358.56)

To $LiAlH_4$ (107 mg, 2.81 mmol, 0.70 eq.) at 0 °C was added a solution of lactone **15** (1.00 g, 4.01 mmol, 1.0 eq.) in THF (6 mL, flask rinsed with 2 x 2 mL) over 5 min. After 40 min of stirring at the same temperature Rochelle salt (1.36 g, 4.81 mmol, 1.2 eq.), DMF (10 mL), freshly ground KOH (900 mg, 16.0 mmol, 4.0 eq.) and 2-Me-$C_6H_4CH_2Br$ (1.10 mL, 1.52 g, 8.22 mmol, 2.1 eq.) were added successively and the reaction mixture was heated to 45 °C for 27 h. Water was added to the reaction and the mixture was diluted with EtOAc. The organic phase was separated and the aqueous phase was extracted with EtOAc. The combined organic extracts were washed repeatedly with brine, dried over $MgSO_4$, filtered and concentrated under reduced pressure. The residue was purified by flash column chromatography (SiO_2, *n*-pentane/EtOAc 1:0 to 10:1) to afford ether **17** (1.43 g, 4.01 mmol, quant.) as a light-yellow oil.

$\mathbf{R_f}$ = 0.52 (*n*-pentane/EtOAc 5:1).

$[\alpha]_D^{28}$ = −14.2° (*c* = 1.33, $CHCl_3$).

^{1}H-NMR (500 MHz, $CDCl_3$): δ [ppm] = 7.30 – 7.28 (m, 1H), 7.22 – 7.14 (m, 3H), 4.58 – 4.48 (m, 2H), 3.64 (ddd, *J* = 8.7, 5.4, 4.1 Hz, 1H), 3.38 (ddd, *J* = 10.1, 8.7, 4.5 Hz, 1H), 3.24 (s, 1H), 2.34 (s, 3H), 1.90 (dt, *J* = 12.5, 3.2 Hz, 1H), 1.77 (ddt, *J* = 15.3, 10.2, 5.2 Hz, 1H), 1.68 – 1.53 (m, 4H), 1.46 – 1.34 (m, 3H), 1.30 – 1.20 (m, 2H), 1.18 – 1.10 (m, 4H), 0.93 – 0.82 (m, 5H), 0.79 (s, 6H).

^{13}C-NMR (126 MHz, $CDCl_3$): δ [ppm] = 136.7, 135.9, 130.4, 128.7, 128.0, 125.9, 72.4, 72.2, 71.6, 59.4, 56.3, 44.2, 42.1, 39.7, 39.2, 33.6, 33.4, 25.5, 24.5, 21.6, 20.6, 19.0, 18.6, 15.4.

IR (ν/cm^{-1}, ATR) = 3440, 2926, 2863, 1715, 1460, 1383, 1364, 1288, 1079, 933, 744.

HRMS (ESI): *m/z* calculated for $C_{24}H_{38}O_2Na^+$ $[M+Na]^+$: 381.2764, found 381.2762.

Note: On 4.5 g scale 96% yield was obtained.

Compound **13** from **17**

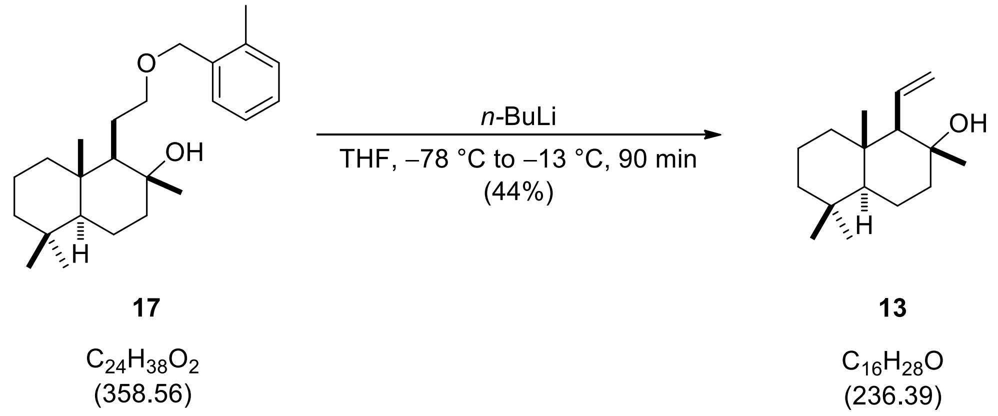

To a solution of ether **17** (177 mg, 458 μmol, 1.0 eq.) in THF (5 mL) at −78 °C was added a solution of *n*-BuLi (0.733 mL, 2.5 M in hexane, 1.83 mmol, 4.0 eq.). After 10 min at that temperature the reaction mixture was warmed to −13 °C (change of cooling baths) and stirred for 90 min. Water was added and the reaction mixture was diluted with EtOAc. The organic phase was separated and the aqueous phase was extracted with EtOAc. The combined organic extracts were washed with brine, dried over $MgSO_4$, filtered and concentrated under reduced pressure. The residue was purified by flash column chromatography (SiO_2, *n*-pentane/EtOAc 1:0 to 20:1 to 15:1 to 10:1) to afford alkene **13** (48.2 mg, 204 μmol, 44%) as a white solid. Crystals suitable for single crystal X-ray diffraction were obtained after slow evaporation from EtOAc/*n*-hexane.

$\mathbf{R_f}$ = 0.50 (*n*-pentane/EtOAc, 9:1).

$[\alpha]^{28}_{D}$ = −16.4 (*c* = 2.03, $CHCl_3$).

^{1}H-NMR (500 MHz, $CDCl_3$): δ [ppm] = 5.82 (dt, *J* = 16.8, 10.2 Hz, 1H), 5.26 (dd, *J* = 10.2, 2.5 Hz, 1H), 5.15 (dd, *J* = 16.9, 2.4 Hz, 1H), 1.94 (s_{br}, 1H), 1.90 (dt, *J* = 12.6, 3.3 Hz, 1H), 1.75 (d, *J* = 10.2 Hz, 1H), 1.71 – 1.64 (m, 1H), 1.56 (tt, *J* = 14.4, 3.7 Hz, 1H), 1.51 – 1.36 (m, 4H), 1.38 – 1.23 (m, 1H), 1.19 (s, 3H), 1.14 (td, *J* = 13.5, 12.8, 4.3 Hz, 1H), 0.93 – 0.89 (m, 4H), 0.89 – 0.85 (m, 4H), 0.81 (s, 3H).

^{13}C-NMR (126 MHz, $CDCl_3$): δ [ppm] = 135.1, 120.6, 71.5, 67.7, 55.9, 42.2, 42.1, 40.9, 37.2, 33.6, 33.5, 25.2, 21.8, 20.2, 18.6, 15.9.

IR (ν/cm^{-1}, ATR) = 3462, 2993, 2923, 2869, 1462, 1386, 1188, 1128, 935, 911.

HRMS (ESI): *m/z* calculated for $C_{16}H_{28}OK^+$ $[M+K]^+$: 275.1772, found 275.1784.

Mp = 89 – 90 °C.

Note: For an attempted synthesis of **13**, see:[1].

Compound **13** from **17** (gram-scale reaction)

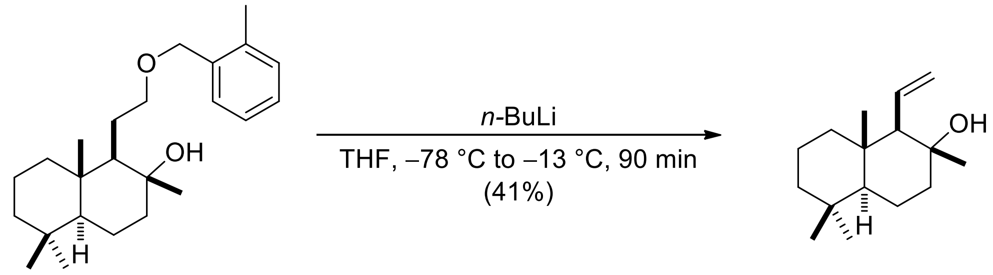

17 $C_{24}H_{38}O_2$ (358.56)

13 $C_{16}H_{28}O$ (236.39)

To a solution of ether **17** (4.72 g, 13.2 mmol, 1.0 eq.) in THF (132 mL) at −78 °C was added a solution of *n*-BuLi (21.1 mL, 2.5 M in hexane, 52.7 mmol, 4.0 eq.). After 10 min at that temperature the reaction mixture was warmed to −13 °C (change of cooling baths) and stirred for 90 min. Water was added and the reaction mixture was diluted with EtOAc. The organic phase was separated and the aqueous phase was extracted with EtOAc. The combined organic extracts were washed with brine, dried over $MgSO_4$, filtered and concentrated under reduced pressure. The residue was purified by flash column chromatography (SiO_2, *n*-pentane/EtOAc 1:0 to 20:1 to 15:1 to 10:1) to afford alkene **13** (1.27 g, 5.45 mmol, 41%) as a white solid.

Compound **19**

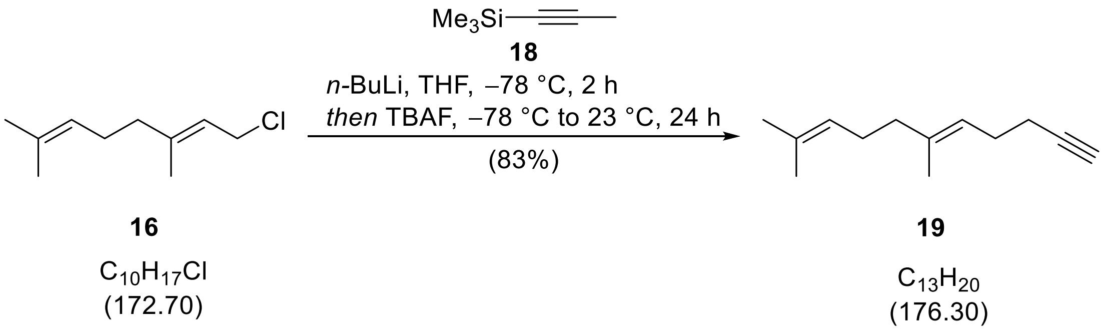

To a solution of 1-(trimethylsilyl)propyne (**18**) (9.52 mL, 7.22 g, 64.3 mmol, 1.2 eq.) in THF (120 mL) at −78 °C was added *n*-BuLi (25.1 mL, 2.5 M in hexane, 62.7 mmol, 1.2 eq.). The yellow solution was stirred for 2 h at that temperature. Geranyl chloride (**16**) (9.03 g, 52.3 mmol, 1.0 eq.) in THF (8 mL, flask rinsed with 2 x 1 mL) was added. The reaction mixture was stirred for 2 h at −78 °C. Tetrabutylammonium fluoride (68.0 mL, 1 M in THF, 68.0 mmol, 1.3 eq.) was added to the flask and the reaction mixture was allowed to reach 23 °C over 24 h. Water was added and the reaction mixture was diluted with EtOAc. The organic phase was separated and the aqueous phase was extracted with EtOAc. The combined organic extracts were washed with brine, dried over $MgSO_4$, filtered and concentrated under reduced pressure. The residue was purified by flash column chromatography (SiO_2, *n*-pentane/CH_2Cl_2, 0:1 to 1:1) to afford alkyne **19** (7.64 g, 43.4 mmol, 83%) as a slightly yellow oil.

$\mathbf{R}_f$ = 0.80 (*n*-pentane).

^{1}H-NMR (700 MHz, $CDCl_3$): δ [ppm] = 5.19 – 5.16 (m, 1H), 5.12 – 5.07 (m, 1H), 2.26 – 2.18 (m, 4H), 2.10 – 2.05 (m, 2H), 2.03 – 1.98 (m, 2H), 1.94 (t, *J* = 2.5 Hz, 1H), 1.68 (s, 3H), 1.62 (s, 3H), 1.60 (s, 3H).

^{13}C-NMR (176 MHz, $CDCl_3$): δ [ppm] = 136.9, 131.6, 124.4, 122.6, 84.7, 68.2, 39.8, 27.3, 26.8, 25.8, 19.1, 17.8, 16.3.

IR (ν/cm^{-1}, ATR) = 3309, 2966, 2921, 2856, 2118, 1445, 1377, 1325, 1242, 1108.

HRMS (EI): *m/z* calculated for $C_{13}H_{19}^+$ [M-H]$^+$ 175.1481, found 175.1488.

The spectral data matched previously obtained data.[2, 3]

Compound **20**

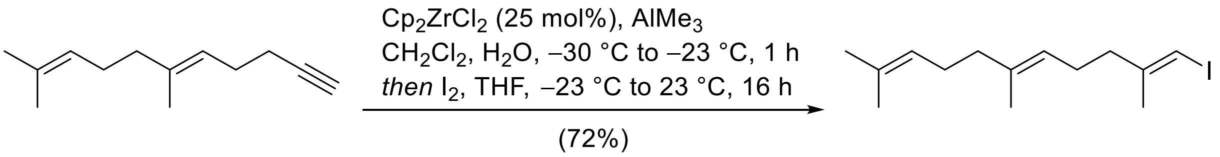

19 $C_{13}H_{20}$ (176.30)

20 $C_{14}H_{23}I$ (318.24)

To a suspension of Cp_2ZrCl_2 (3.79 g, 13.0 mmol, 25 mol%) in CH_2Cl_2 (200 mL) at −30 °C was added $AlMe_3$ (77.8 mL, 2.0 M in toluene, 156 mmol, 3.0 eq.). H_2O (935 µL, 51.9 mmol, 1.0 eq.) was added slowly. The reaction mixture was warmed to −23 °C over 1 h. Alkyne **19** (9.14 g 51.9 mmol, 1.0 eq.) in CH_2Cl_2 (75 mL) was added dropwise to the reaction mixture at that temperature. After 1 h a solution of iodine (15.8 g, 62.2 mmol, 1.2 eq.) in THF (75 mL) was added dropwise at the same temperature. The reaction was allowed to reach 23 °C over 15 h. A saturated aqueous solution of K_2CO_3 (15 mL) was added dropwise. The reaction mixture was diluted with CH_2Cl_2. The organic phase was separated and the aqueous phase was extracted with CH_2Cl_2. The combined organic extracts were washed with brine, dried over $MgSO_4$, filtered and concentrated under reduced pressure. The residue was purified by flash column chromatography (SiO_2, *n*-pentane) to afford vinyl iodide **20** (11.9 g, 37.2 mmol, 72%) as a colorless oil.

$\mathbf{R_f}$ = 0.92 (*n*-pentane).

^{1}H-NMR (700 MHz, $CDCl_3$): δ [ppm] = 5.87 (sext, *J* = 1.1 Hz, 1H), 5.10 – 5.05 (m, 2H), 2.25 – 2.20 (m, 2H), 2.16 – 2.10 (m, 2H), 2.09 – 2.04 (m, 2H), 2.00 – 1.96 (m, 2H), 1.84 (d, *J* = 1.1 Hz, 3H), 1.69 (d, *J* = 1.4 Hz, 3H), 1.61 (s, 3H), 1.59 (s, 3H).

^{13}C-NMR (176 MHz, $CDCl_3$): δ [ppm] = 148.0, 136.2, 131.6, 124.4, 123.1, 74.9, 39.8, 39.7, 26.9, 26.4, 25.9, 24.1, 17.9, 16.2.

IR (ν/cm^{-1}, ATR) = 2963, 2918, 2850, 1442, 1377, 1267, 1141, 833, 766, 664.

HRMS (EI): *m/z* calculated $C_{14}H_{23}^+$ $[M-I]^+$ 191.1794, found 191.1799.

The spectral data matched previously obtained data.[3]

Note: The use of fresh Cp_2ZrCl_2 and $AlMe_3$ is necessary for reproducible yield.

Compound **S2**

Procedure using Corey-Noe-Lin ligand

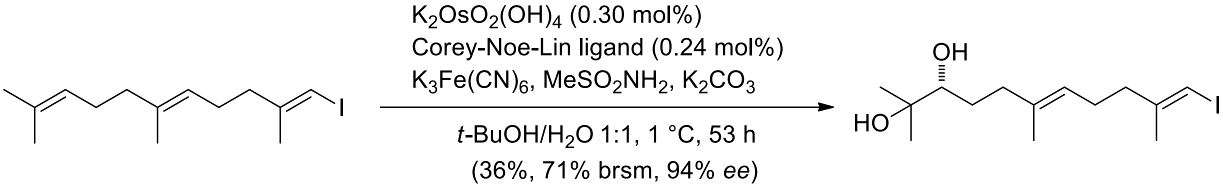

20

$C_{14}H_{23}I$
(318.24)

S2

$C_{14}H_{25}IO_2$
(352.25)

Corey-Noe-Lin ligand

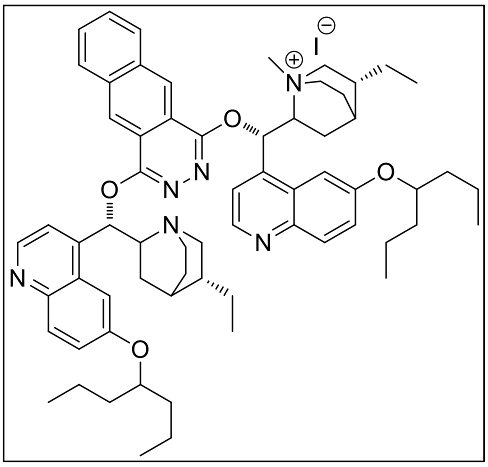

$K_2OsO_2(OH)_4$ (3.5 mg, 9.5 µmol, 0.30 mol%), $K_3Fe(CN)_6$ (3.10 g, 9.43 mmol, 3.0 eq.), $MeSO_2NH_2$ (299 mg, 3.14 mmol, 1.0 eq.), K_2CO_3 (1.30 g, 9.43 g, 3.0 eq.) and Corey-Noe-Lin ligand (8.6 mg, 7.5 µmol, 0.24 mol%) were dissolved under stirring in *t*-BuOH/H_2O (32 mL, 1:1) at 23 °C. After solvation the reaction mixture was cooled to 1 °C and stirred for 30 min. Vinyl iodide **20** (1.00 g, 3.14 mmol, 1.0 eq.) was added and the reaction mixture was stirred for 53 h at the same temperature. Na_2SO_3 (1.98 g, 15.7 g, 5.0 eq.) and a saturated aqueous solution of Na_2SO_3 (5 mL) were added at 1 °C. After 30 min the reaction mixture was allowed to warm to 23 °C and was diluted with EtOAc. The organic phase was separated and the aqueous phase was extracted with EtOAc. The combined organic extracts were washed with KOH (2 M) and brine, dried over $MgSO_4$, filtered and concentrated under reduced pressure. The residue was purified by flash column chromatography (SiO_2, *n*-pentane/EtOAc 5:1 to 4:1) to afford diol **S2** (399 mg, 1.13 mmol, 36%, 71% brsm, 94% *ee*) and recovered vinyl iodide **20** (490 mg, 1.54 mmol) as colorless oils.

$\mathbf{R}_f$ = 0.11 (*n*-pentane/EtOAc, 5:1).

$[\alpha]_D^{29}$ = +9.4 (*c* = 1.38, $CHCl_3$).

^{1}H-NMR (700 MHz, $CDCl_3$): δ [ppm] = 5.86 (sext, *J* = 1.1 Hz, 1H), 5.15 – 5.10 (m, 1H), 3.33 (dd, *J* = 10.6, 2.0 Hz, 1H), 2.25 – 2.21 (m, 3H), 2.19 (s, 1H), 2.16 – 2.11 (m, 2H), 2.09 – 2.03 (m, 1H), 2.01 (s, 1H), 1.83 (d, *J* = 1.1 Hz, 3H), 1.61 (q, *J* = 0.9 Hz, 3H), 1.57 (dddd, *J* = 13.8, 9.1, 7.1, 2.0 Hz, 1H), 1.40 (dddd, *J* = 14.0, 10.6, 8.8, 5.4 Hz, 1H), 1.20 (s, 3H), 1.16 (s, 3H).
^{13}C-NMR (176 MHz, $CDCl_3$): δ [ppm] = 147.8, 136.1, 123.9, 78.3, 75.0, 73.2, 39.5, 36.9, 29.9, 26.7, 26.3, 24.0, 23.4, 16.1.
IR (ν/cm^{-1}, ATR) = 3407, 3057, 2924, 2853, 1448, 1379, 1268, 1142, 1076, 766.
HRMS (ESI): *m/z* calculated for $C_{14}H_{25}IO_2Na^+$ $[M+Na]^+$ 375.0791, found 375.0805.

Procedure using $(DHQD)_2PHAL$

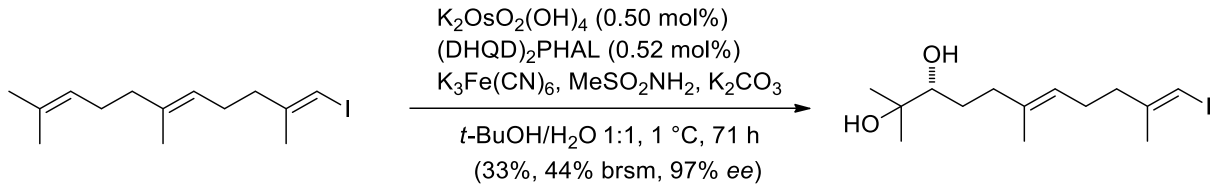

20 $C_{14}H_{23}I$ (318.24)

S2 $C_{14}H_{25}IO_2$ (352.25)

$K_2OsO_2(OH)_4$ (20.4 mg, 56.0 µmol, 0.50 mol%), $K_3Fe(CN)_6$ (11.0 g, 33.5 mmol, 3.0 eq.), $MeSO_2NH_2$ (1.05 g, 11.0 mmol, 1.0 eq.), K_2CO_3 (4.61 g, 33.4 g, 3.0 eq.) and $(DHQD)_2PHAL$ (44.9 mg, 58.0 µmol, 0.52 mol%) were dissolved under stirring in *t*-BuOH/H_2O (116 mL, 1:1) at 23 °C. After solvation the reaction mixture was cooled to 1 °C and stirred for 30 min. Vinyl iodide **20** (3.54 g, 11.1 mmol, 1.0 eq.) was added and the reaction mixture was stirred for 71 h at the same temperature. Na_2SO_3 (7.01 g, 55.6 g, 5.0 eq.) and a saturated aqueous solution of Na_2SO_3 (5 mL) were added at 1 °C. After 30 min the reaction mixture was allowed to warm to 23 °C and was diluted with EtOAc. The organic phase was separated and the aqueous phase was extracted with EtOAc. The combined organic extracts were washed with KOH (2 M) and brine, dried over $MgSO_4$, filtered and concentrated under reduced pressure. The residue was purified by flash column chromatography (SiO_2, *n*-pentane/EtOAc 5:1 to 4:1) to afford diol **S2** (1.28 g, 3.64 mmol, 33%, 44% brsm, 97% *ee*) and recovered vinyl iodide **20** (896 mg, 2.81 mmol) as colorless oils.

Determination of absolute configuration: Mosher ester analysis of S2

(*S*)-Mosher ester

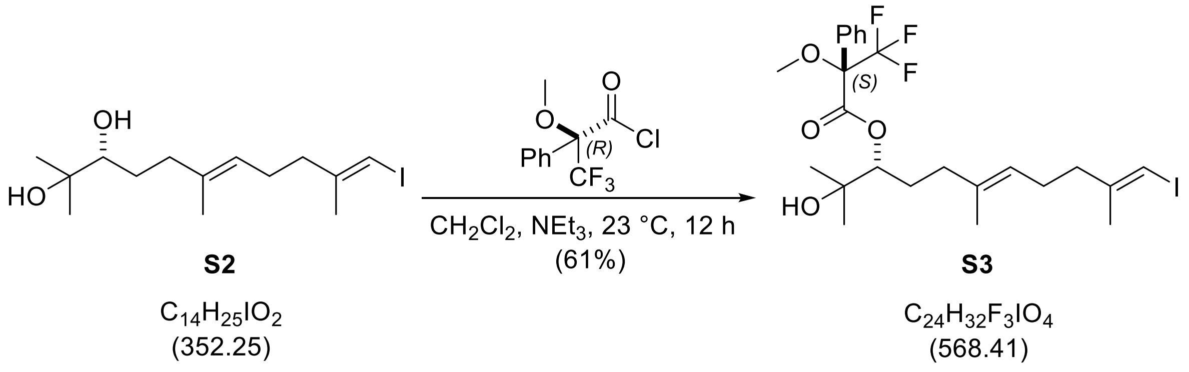

To a solution of diol **S2** (8.0 mg, 23 μmol, 1.0 eq.) in CH_2Cl_2 (0.5 mL) at 23 °C was added NEt_3 (25.2 μL, 182 μmol, 8.0 eq.), (*R*)-MTPA-Cl (8.50 μL, 45.4 μmol, 2.0 eq.) and DMAP (one crystal) successively. The solution was stirred for 12 h. The solution was directly purified by flash column chromatography (SiO_2, *n*-pentane/EtOAc 1:0 to 9:1) to afford ester **S3** as a white solid (7.9 mg, 14 μmol, 61%).

^{1}H-NMR (500 MHz, $CDCl_3$): δ [ppm] = 7.64 – 7.58 (m, 2H), 7.43 – 7.38 (m, 3H), 5.92 – 5.83 (m, 1H), 5.05 – 5.01 (m, 1H), 4.98 (dd, *J* = 9.9, 2.2 Hz, 1H), 3.58 (s, 3H), 2.24 – 2.20 (m, 2H), 2.16 – 2.09 (m, 2H), 2.00 – 1.92 (m, 2H), 1.83 (s, 3H), 1.82 – 1.75 (m, 1H), 1.65 (dtd, *J* = 14.5, 9.6, 5.4 Hz, 1H), 1.55 (s, 3H), 1.51 (s_{br}, 1H), 1.18 (s, 3H), 1.14 (s, 3H).

HRMS (ESI): *m/z* calculated for $C_{24}H_{32}F_3IO_4Na^+$ $[M+Na]^+$ 591.1190, found 591.1199.

(*R*)-Mosher ester

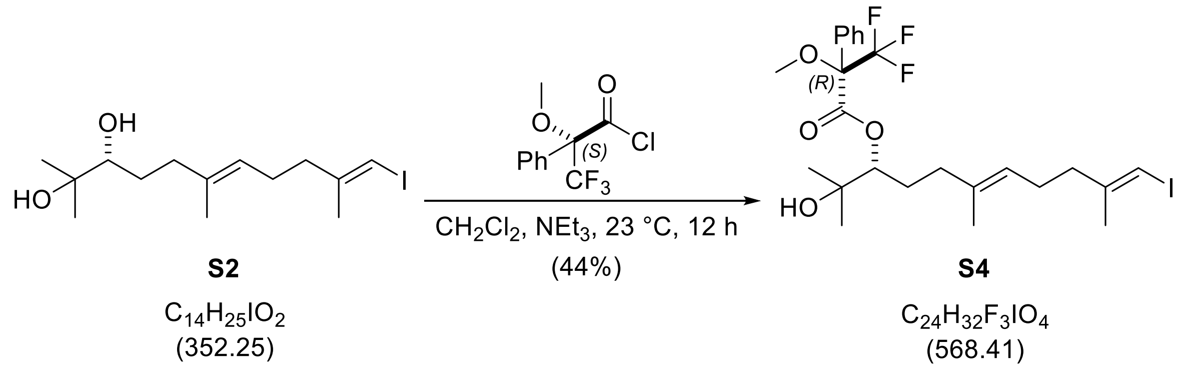

To a solution of diol **S2** (7.3 mg, 21 μmol, 1.0 eq.) in CH_2Cl_2 (0.5 mL) at 23 °C was added NEt_3 (23.0 μL, 166 μmol, 8.0 eq.), (*S*)-MTPA-Cl (7.76 μL, 41.4 μmol, 2.0 eq.) and DMAP (one crystal) successively. The solution was stirred for 12 h. The solution was directly purified by flash column chromatography (SiO_2, *n*-pentane/EtOAc 1:0 to 9:1) to afford ester **S4** as a white solid (5.2 mg, 9.1 μmol, 44%).

^{1}H-NMR (500 MHz, $CDCl_3$): δ [ppm] = 7.65 – 7.61 (m, 2H), 7.43 – 7.39 (m, 3H), 5.88 – 5.86 (m, 1H), 5.01 – 4.98 (m, 1H), 4.97 (dd, *J* = 10.2, 2.1 Hz, 1H), 3.57 (s, 3H), 2.25 – 2.19 (m, 2H), 2.14 – 2.08 (m, 2H), 1.89 – 1.84 (m, 2H), 1.83 (s, 3H), 1.75 – 1.65 (m, 1H), 1.62 – 1.57 (m, 1H), 1.51 (s, 3H), 1.28 (s, 1H), 1.23 (s, 3H), 1.16 (s, 3H).

HRMS (ESI): *m/z* calculated for $C_{24}H_{32}F_3IO_4Na^+$ $[M+Na]^+$ 591.1190, found 591.1186.

The assignments were resolved using NMR spectroscopic methods: ^{1}H, COSY, HMQC, HMBC. The comparison of both esters shows (*R*)-configuration at C-3.[4]

Δ δ [(*S*)-MTPA – (*R*)-MTPA] =

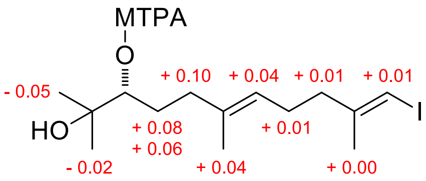

HPLC-Analysis of S2

HPLC-Analysis was performed using Chiralpak IA, 3% EtOH/hexane, 20 °C, 1 mL/min.

1) Racemic mixture

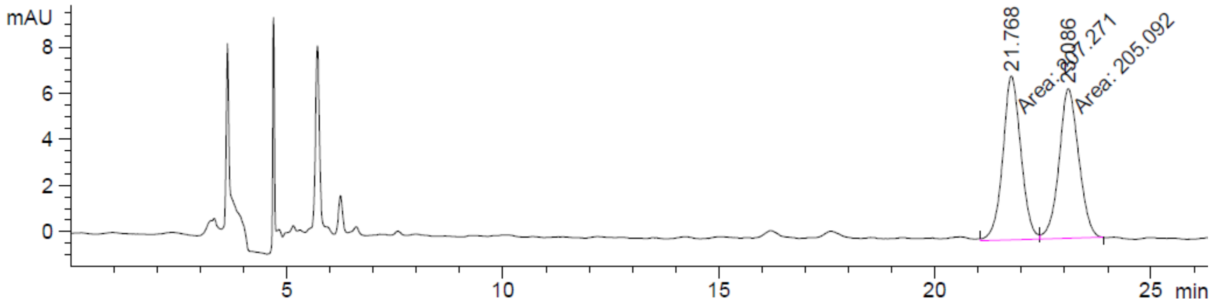

Signal 4: DAD1 E, Sig=270,4 Ref=360,100

Peak #	RetTime [min]	Type	Width [min]	Area [mAU*s]	Height [mAU]	Area %
1	21.768	MF	0.4864	207.27090	7.10185	50.2642
2	23.086	FM	0.5272	205.09232	6.48366	49.7358
Totals :				412.36322	13.58551	

2) **S2** synthesized using CNL-ligand

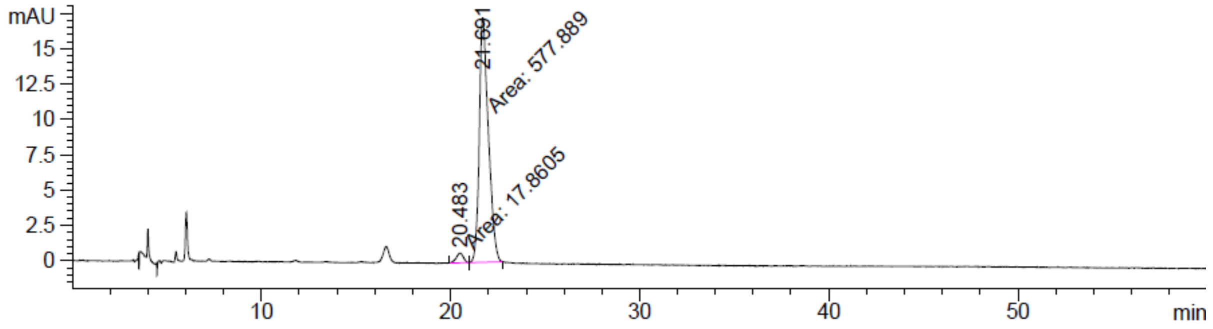

Signal 4: DAD1 E, Sig=270,4 Ref=360,100

Peak #	RetTime [min]	Type	Width [min]	Area [mAU*s]	Height [mAU]	Area %
1	20.483	MM	0.4261	17.86053	6.98630e-1	2.9980
2	21.691	MM	0.5563	577.88861	17.31263	97.0020
Totals :				595.74914	18.01126	

3) **S2** synthesized using $(DHQD)_2$PHAL-ligand

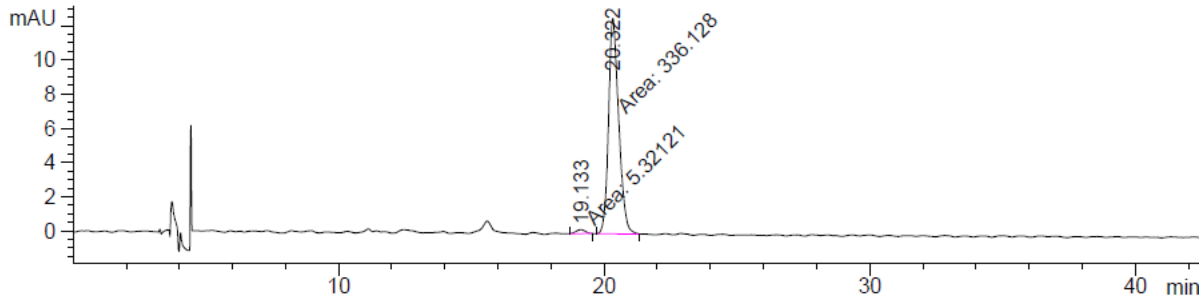

Signal 4: DAD1 E, Sig=270,4 Ref=360,100

Peak #	RetTime [min]	Type	Width [min]	Area [mAU*s]	Height [mAU]	Area %
1	19.133	MM	0.3903	5.32121	2.27222e-1	1.5584
2	20.322	MM	0.4445	336.12796	12.60288	98.4416

Totals : 341.44917 12.83010

Compound **14**

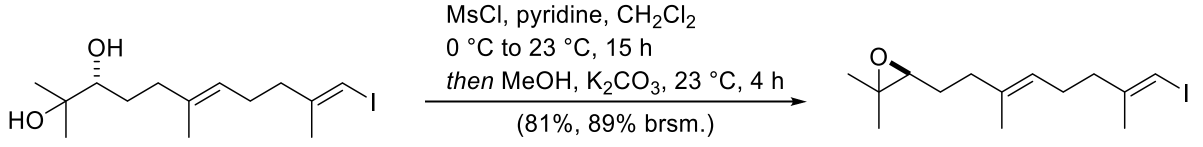

S2
$C_{14}H_{25}IO_2$
(352.25)

14
$C_{14}H_{23}IO$
(334.24)

To a solution of diol **S2** (1.52 g, 4.31 mmol, 1.0 eq.) in CH_2Cl_2 (13 mL) at 0 °C was added pyridine (5.20 mL, 65.0 mmol, 15 eq.) and MsCl (367 μL, 543 mg, 4.74 mmol, 1.1 eq.) sequentially. The reaction mixture was allowed to warm to 23 °C over 15 h. Methanol (60 mL) and K_2CO_3 (5.95 g, 43.1 mmol, 10 eq.) were added sequentially at 23 °C and the reaction mixture was stirred for 4 h. The reaction mixture was concentrated under reduced pressure. The residue was diluted with CH_2Cl_2 and water. The organic phase was separated and the aqueous phase was extracted with CH_2Cl_2. The combined organic extracts were washed with a saturated aqueous solution of $CuSO_4$ and brine, dried over $MgSO_4$, filtered and concentrated under reduced pressure. The residue was purified by flash column chromatography (SiO_2, *n*-pentane/EtOAc 50:1 to 3:1) to afford epoxide **14** (1.16 g, 3.48 mmol, 81%, 89% brsm.) as a slightly yellow oil and recovered diol **S2** (140 mg, 397 μmol) as a colorless oil.

$\mathbf{R_f}$ = 0.54 (*n*-pentane/EtOAc, 10:1).

$[\alpha]^{29}_{D}$ = −2.66 (*c* = 0.45, $CHCl_3$).

^{1}H-NMR (700 MHz, $CDCl_3$): δ [ppm] = 5.89 – 5.86 (m, 1H), 5.15 – 5.10 (m, 1H), 2.69 (t, *J* = 6.2 Hz, 1H), 2.25 – 2.21 (m, 2H), 2.19 – 2.11 (m, 3H), 2.08 (dt, *J* = 14.4, 7.5 Hz, 1H), 1.84 (s, 3H), 1.64 – 1.60 (m, 5H), 1.31 (s, 3H), 1.26 (s, 3H).

^{13}C-NMR (176 MHz, $CDCl_3$): δ [ppm] = 147.8, 135.4, 123.7, 74.9, 64.3, 58.5, 39.6, 36.5, 27.6, 26.4, 25.1, 24.1, 18.9, 16.2.

IR (ν/cm^{-1}, ATR) = 2958, 2923, 2855, 1447, 1377, 1322, 1267, 1139, 1120, 872.

HRMS (ESI): *m/z* calculated for $C_{14}H_{24}IO^+$ $[M+H]^+$: 335.0867, found 335.0869.

Compound **12**

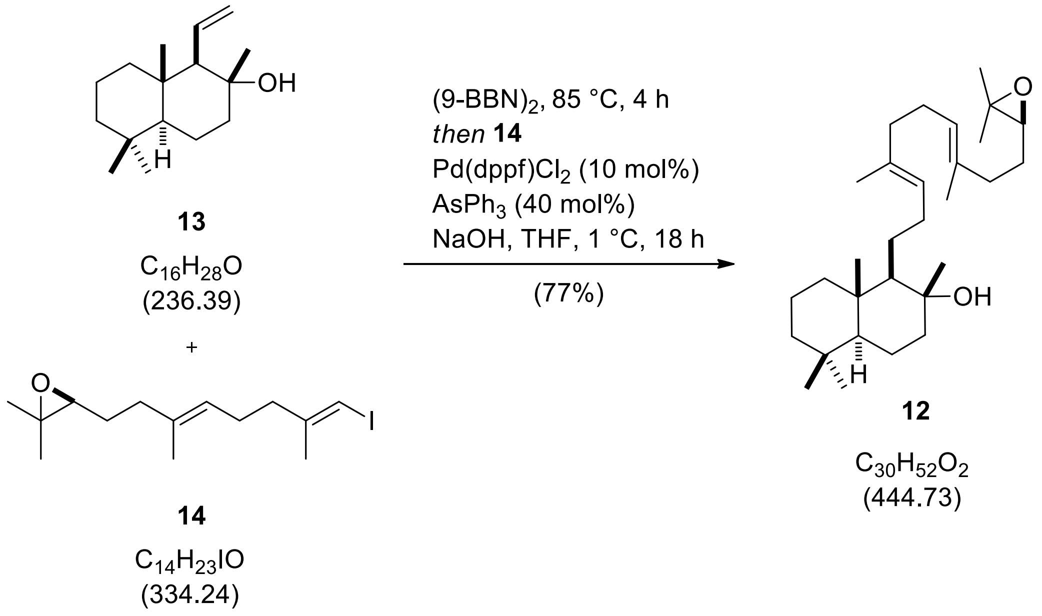

Alkene **13** (1.18 g, 4.97 mmol, 1.4 eq.) and 9-BBN dimer[5] (1.21 g, 9.94 mmol, 2.8 eq.) were combined and stirred for 5 min, then the reaction flask was heated to 85 °C for 4 h. The reaction flask was allowed to cool to 23 °C and THF (10 mL, degassed by freeze pump thaw 3x) was added. The reaction flask was cooled to 0 °C and NaOH (7.10 mL, 3 M, 21.3 mmol, 6.0 eq., purged with Ar for 20 min) was added dropwise. A solution of epoxide **14** (1.19 g, 3.55 mmol, 1.0 eq.) and $AsPh_3$ (435 mg, 1.42 mmol, 40 mol%) in THF (6 mL, purged with Ar for 20 min, the flask was rinsed 2x with 1 mL) was added to the reaction mixture. The reaction mixture was purged with Ar for 5 min. $Pd(dppf)Cl_2$ (260 mg, 355 µmol, 10 mol%) was added and the reaction mixture was stirred at 1 °C for 18 h. Water and EtOAc were added to the reaction mixture sequentially. The organic phase was separated and the aqueous phase was extracted with EtOAc. The combined organic extracts were washed with brine, dried over $MgSO_4$, filtered and concentrated under reduced pressure. The residue was purified by flash column chromatography (SiO_2, *n*-pentane/EtOAc 40:1 to 1:1) to afford epoxy dienol **12** (1.22 g, 2.75 mmol, 77%) as a colorless oil.

$\mathbf{R_f}$ = 0.64 (*n*-pentane/EtOAc, 5:1).

$[\alpha]_D^{29}$ = +0.52 (*c* = 0.64, $CHCl_3$).

^{1}H-NMR (700 MHz, $CDCl_3$): δ [ppm] = 5.20 – 5.13 (m, 2H), 2.70 (t, *J* = 6.3 Hz, 1H), 2.19 – 2.11 (m, 1H), 2.11 – 2.03 (m, 5H), 1.99 (t, *J* = 7.7 Hz, 2H), 1.86 (dt, *J* = 12.3, 3.2 Hz, 1H), 1.69 – 1.62 (m, 4H), 1.62 – 1.61 (m, 3H), 1.61 – 1.60 (m, 3H), 1.60 – 1.55 (m, 2H), 1.46 – 1.40 (m, 2H), 1.40 – 1.33 (m, 2H), 1.29 (s, 3H), 1.27 – 1.24 (m, 4H), 1.20 (s, 1H), 1.15 (dd, *J* = 13.6, 4.2

Hz, 1H), 1.12 (s, 3H), 1.03 (t, J = 4.0 Hz, 1H), 0.97 (td, J = 13.0, 3.8 Hz, 1H), 0.91 (dd, J = 12.2, 2.4 Hz, 1H), 0.86 (s, 3H), 0.78 (s, 6H).

^{13}C-NMR (176 MHz, $CDCl_3$): δ [ppm] = 135.1, 134.2, 125.3, 125.1, 74.2, 64.3, 61.7, 58.5, 56.3, 44.7, 42.2, 39.9, 39.8, 39.3, 36.5, 33.6, 33.4, 31.6, 27.6, 26.8, 25.7, 25.1, 24.0, 21.7, 20.7, 18.9, 18.6, 16.4, 16.2, 15.6.

IR (ν/cm^{-1}, ATR) = 3479, 2924, 2852, 1458, 1384, 1249, 1158, 1044, 938, 756.

HRMS (ESI): *m/z* calculated for $C_{30}H_{52}O_2Na^+$ $[M+Na]^+$ 467.3859, found 467.3867.

Compound **10** and compound **21**

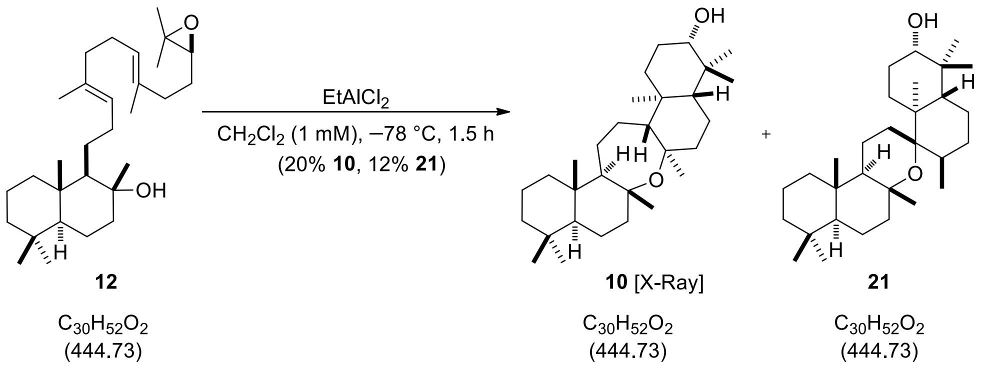

To a solution of $EtAlCl_2$ (0.20 mL, 1 M in hexane, 0.20 mmol, 3.0 eq.) in CH_2Cl_2 (33 mL) at −78 °C was added a precooled solution of epoxy dienol **12** (29.3 mg, 66.0 µmol, 1.0 eq.) in CH_2Cl_2 (33 mL) over 1 h (cannula passing through dry ice). The reaction mixture was then stirred for 30 min at the same temperature. NH_3 (aq., 0.2 mL) and MeOH/H_2O (4:1, 0.2 mL) were added successively. The reaction mixture was allowed to reach 23 °C, and then a solution of saturated aqueous Rochelle salt was added. The organic phase was separated and the aqueous phase was extracted with CH_2Cl_2. The combined organic extracts were washed with brine, dried over $MgSO_4$, filtered and concentrated under reduced pressure. The residue was purified by flash column chromatography (SiO_2, *n*-pentane/EtOAc 1:0 to 15:1 to 10:1 to 5:1) to afford alcohol **10** (5.9 mg, 13 µmol, 20%) as a white solid and oxane **21** (3.4 mg, 7.6 µmol, 12%) as a colorless oil. Crystals of **10** suitable for single crystal X-ray diffraction were obtained after slow evaporation from CH_2Cl_2/MeCN.

Compound **10**

R*f* = 0.48 (*n*-pentane/EtOAc, 9:1).

$[\alpha]_D^{28}$ = +6.3 (*c* = 0.49, CH_2Cl_2).

^{1}H-NMR (700 MHz, C_6D_6): δ [ppm] = 3.03 – 2.96 (m, 1H), 1.94 (ddt, *J* = 16.0, 12.8, 3.3 Hz, 2H), 1.82 – 1.65 (m, 4H), 1.64 – 1.48 (m, 3H), 1.47 – 1.39 (m, 6H), 1.37 – 1.32 (m, 3H), 1.36 (s, 3H), 1.35 (s, 3H), 1.22 – 1.15 (m, 4H), 0.95 (s, 3H), 0.91 – 0.89 (m, 1H), 0.87 – 0.82 (m, 3H), 0.85 (s, 3H), 0.79 (s, 3H), 0.75 – 0.73 (m, 1H), 0.74 (s, 3H), 0.74 (s, 3H), 0.68 (s, 3H).

^{13}C-NMR (176 MHz, C_6D_6): δ [ppm] = 80.1, 79.9, 78.4, 61.1 (2C), 56.6, 55.5, 45.8, 45.6, 42.3, 40.6, 39.2, 39.1, 38.9, 38.7, 33.7, 33.6, 28.4, 28.1, 25.7, 25.5 (2C), 25.3, 21.8, 21.2, 20.8, 19.3, 16.1 (2C), 15.7.

IR (ν/cm^{-1}, ATR) = 3394, 2924, 2855, 1458, 1377, 1283, 1187, 1126, 1084, 1043.

HRMS (ESI): *m/z* calculated for $C_{30}H_{52}O_2Na^+$ $[M+Na]^+$ 467.3859, found 467.3848. **Mp** = 197 – 199 °C.

Note: The use of fresh $EtAlCl_2$ is necessary for reproducible yield. **10** showed signs of decomposition after treatment with $CDCl_3$ (unpurified) for 12 h.

Compound **21**

$\mathbf{R_f}$ = 0.50 (*n*-pentane/EtOAc, 9:1).

$[\alpha]_D^{28}$ = −3.9 (*c* = 0.28, CH_2Cl_2).

^{1}**H-NMR** (700 MHz, C_6D_6): δ [ppm] = 3.01 (dd, *J* = 11.2, 4.1 Hz, 1H), 1.99 – 1.90 (m, 1H), 1.86 – 1.81 (m, 1H), 1.71 – 1.67 (m, 1H), 1.66 – 1.28 (m, 20H), 1.30 (s, 3H), 1.17 (s, 3H), 1.11 – 1.09 (m, 1H), 1.03 (s, 3H), 1.01 (d, *J* = 6.7 Hz, 3H), 0.91 – 0.89 (m, 1H), 0.86 – 0.84 (m, 2H), 0.85 (s, 3H), 0.81 (s, 3H), 0.78 (s, 3H), 0.68 (s, 3H).

^{13}**C NMR** (176 MHz, C_6D_6): δ [ppm] = 80.8, 78.9, 75.0, 57.1, 54.5, 47.5, 45.2, 43.6, 42.4, 40.0, 39.1, 38.3, 37.3, 33.5, 33.4, 32.9, 32.1, 28.8, 28.2, 26.4, 24.3, 21.5 (2C), 21.2, 19.1, 18.0, 17.5, 16.0, 15.5, 15.2.

IR (ν/cm^{-1}, ATR) = 3415, 2927, 2867, 1462, 1380, 1322, 1045, 1017, 998, 976.

HRMS (ESI): *m/z* calculated for $C_{30}H_{52}O_2Na^+$ $[M+Na]^+$ 467.3859, found 467.3850.

Compound **22**

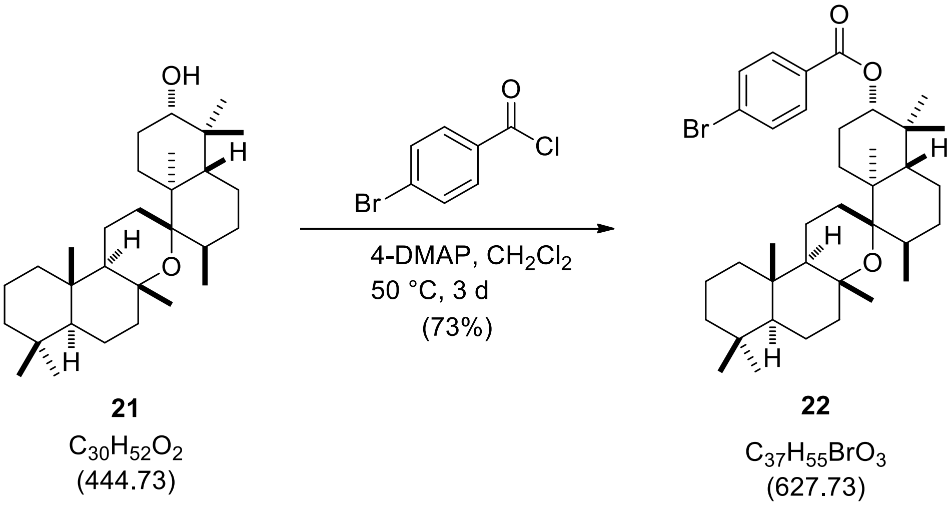

To a solution of oxane **21** (3.6 mg, 8.1 µmol, 1.0 eq.) in CH_2Cl_2 (1 mL) at 23 °C was added *N*,*N*-dimethyl-4-aminopyridine (19.8 mg, 162 µmol, 20 eq.) and 4-bromobenzoyl chloride (9.1 mg, 41 µmol, 5.0 eq.). The reaction mixture was stirred for 3 d at 50 °C in a closed vial. The residue was directly purified by flash column chromatography (SiO_2, *n*-pentane/EtOAc 1:0 to 20:1 to 5:1) to afford ester **22** (3.7 mg, 5.9 µmol, 73%) as a white solid. Crystals suitable for single crystal X-ray diffraction were obtained after slow evaporation from 1,2-dichloroethane, 2-propanol and benzene.

R*f* = 0.80 (*n*-pentane/EtOAc, 9:1).

$[\alpha]_D^{25}$ = –2.4 (*c* = 0.07, $CHCl_3$).

^{1}H-NMR (700 MHz, $CDCl_3$): δ [ppm] = δ 7.91 (d, *J* = 8.5 Hz, 2H), 7.58 (d, *J* = 8.5 Hz, 2H), 4.69 – 4.62 (m, 1H), 1.94 – 1.86 (m, 1H), 1.85 – 1.79 (m, 1H), 1.79 – 1.69 (m, 3H), 1.68 – 1.58 (m, 6H), 1.49 – 1.35 (m, 6H), 1.27 – 1.24 (m, 6H), 1.22 – 1.16 (m, 2H), 1.14 – 1.08 (m, 2H), 1.04 (s, 3H), 1.01 (s, 3H), 0.92 (d, *J* = 6.6 Hz, 3H), 0.89 (s, 3H), 0.89 – 0.86 (m, 2H), 0.85 (s, 3H), 0.78 (s, 3H), 0.73 (s, 3H).

^{13}C-NMR (176 MHz, $CDCl_3$): δ [ppm] = 165.9, 131.8 (2C), 131.2 (2C), 130.0, 128.0, 82.6, 80.7, 75.1, 56.9, 54.0, 47.4, 44.8, 43.3, 42.3, 39.7, 38.2, 38.1, 37.2, 33.5, 33.4, 32.4, 31.7, 28.7, 26.2, 24.2, 24.0, 21.4, 21.2, 21.0, 18.8, 17.6, 17.2, 16.7, 15.9, 15.0.

IR (ν/cm^{-1}, ATR) = 2925, 2854, 1718, 1591, 1461, 1396, 1377, 1271, 847, 757.

HRMS (EI): *m/z* calculated for $C_{37}H_{55}O_3Br^+$ $[M]^+$: 626.3329, found 626.3308.

Mp = 182 – 184 °C.

Cupacinoxepin **4**

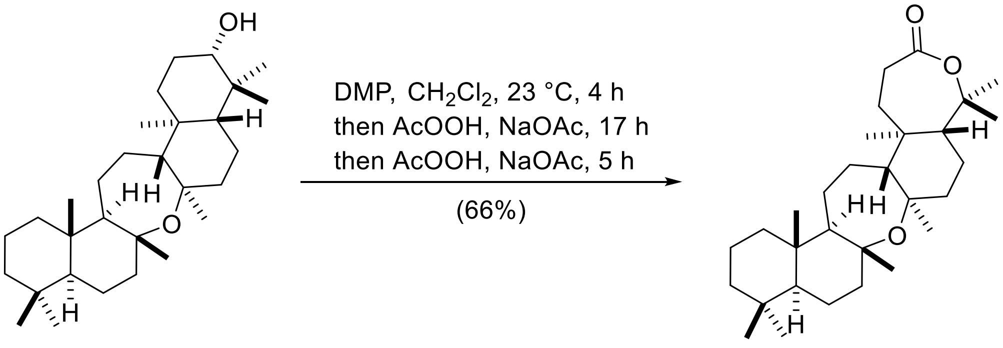

10
$C_{30}H_{52}O_2$
(444.73)

4
$C_{30}H_{50}O_3$
(458.72)

To a solution of alcohol **10** (5.7 mg, 13 μmol, 1.0 eq.) in CH_2Cl_2 (2.5 mL) at 23 °C was added Dess-Martin periodinane (DMP)[6] (10.9 mg, 26 μmol, 2.0 eq.). The reaction mixture was stirred for 4 h. Afterwards, NaOAc (34.9 mg, 256 μmol, 20 eq.) and AcOOH (24.6 μL, 27.8 mg, 128 μmol, 35% in acetic acid, 10 eq.) were added successively. The reaction flask was covered with aluminum foil and stirred for 17 h. Another portion of NaOAc (34.9 mg, 256 μmol, 20 eq.) and AcOOH (24.6 μL, 27.8 mg, 128 μmol, 35% in acetic acid, 10 eq.) were added successively. The reaction mixture was stirred for 5 h. The residue was directly purified by flash column chromatography (SiO_2, *n*-pentane/EtOAc 1:0 to 10:1 to 5:1 and Al_2O_3, *n*-pentane/EtOAc 1:0 to 5:1 and SiO_2, toluene/Et_2O 5:1 to 1:1) to afford cupacinoxepin **4** (3.9 mg, 8.5 μmol, 66%) as a white solid. Crystals suitable for single crystal X-ray diffraction were obtained after slow evaporation from acetone/*n*-hexane.

$\mathbf{R_f}$ = 0.40 (*n*-pentane/EtOAc, 4:1).

$[\alpha]_D^{26}$ = +49.0 (*c* = 0.04, $CHCl_3$) [Lit[7]:$[\alpha]_D^{22}$ = +56.4 (*c* = 1.3, $CHCl_3$)].

^{1}H-NMR (500 MHz, $CDCl_3$): δ [ppm] = 2.66 (td, *J* = 13.8, 4.8 Hz, 1H), 2.48 (ddd, *J* = 14.2, 5.2, 3.5 Hz, 1H), 1.93 – 1.81 (m, 3H), 1.80 – 1.69 (m, 4H), 1.69 – 1.58 (m, 3H), 1.59 – 1.49 (m, 5H), 1.48 (s, 3H), 1.47 – 1.39 (m, 2H), 1.36 (s, 3H), 1.36 – 1.32 (m, 2H), 1.29 (s, 3H), 1.25 (s, 3H), 1.24 – 1.17 (m, 2H), 1.13 (td, *J* = 13.5, 4.3 Hz, 1H), 1.00 (s, 3H), 0.93 – 0.86 (m, 2H), 0.85 (s, 3H), 0.77 (s, 3H), 0.74 (s, 3H).

^{13}C-NMR (176 MHz, $CDCl_3$): δ [ppm] = 175.0, 85.9, 80.5, 78.9, 61.4, 61.0, 56.3, 52.2, 45.3, 43.7, 42.1, 41.2, 40.5, 40.3, 39.0, 33.6 (2C), 32.6, 30.8, 27.4, 26.5, 26.0, 25.5, 25.2, 24.2, 21.6, 20.9, 19.0, 18.4, 16.0.

IR (ν/cm^{-1}, ATR) = 2922, 2854, 1719, 1459, 1375, 1286, 1131, 1022, 977, 753.

HRMS (EI): *m/z* calculated for $C_{30}H_{50}O_3^+$ $[M]^+$: 458.3754, found 458.3776.

Mp: 204 – 207 °C.

Thiocarbamate **S5**

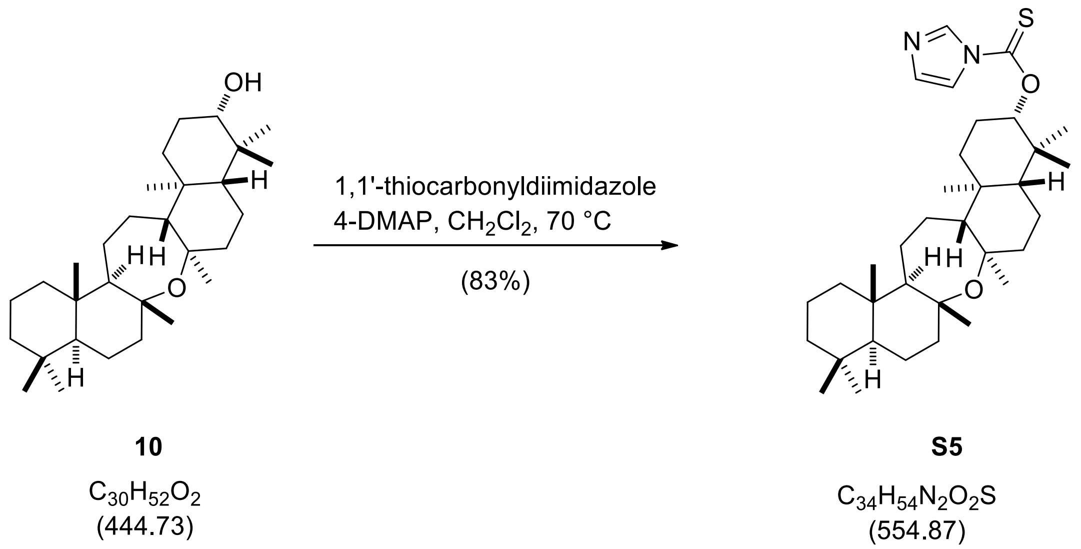

To a solution of alcohol **10** (8.2 mg, 18 μmol, 1.0 eq.) in CH_2Cl_2 (2.5 mL) at 23 °C was added 1,1'-thiocarbonyldiimidazole (69.2 mg, 369 μmol, 20 eq.) and *N*,*N*-dimethyl-4-aminopyridine (45.1 mg, 369 μmol, 20 eq.). The reaction mixture was stirred for 13.5 h in a pressure tube at 70 °C. The residue was directly purified by flash column chromatography (SiO_2, *n*-pentane/EtOAc 1:0 to 10:1 to 5:1) to afford thiocarbamate **S5** (8.5 mg, 15 μmol, 83%) as a white solid.

R*f* = 0.32 (*n*-pentane/EtOAc, 9:1).

$[\alpha]_D^{25}$ = +36.2 (*c* = 0.71, CH_2Cl_2).

^{1}H-NMR (700 MHz, C_6D_6): δ [ppm] = 8.41 (s, 1H), 7.52 – 7.48 (m, 1H), 7.02 – 6.95 (m, 1H), 5.12 (dd, *J* = 12.0, 4.7 Hz, 1H), 1.97 (dt, *J* = 12.6, 3.2 Hz, 1H), 1.87 (dt, *J* = 12.8, 3.2 Hz, 1H), 1.84 – 1.73 (m, 4H), 1.68 – 1.55 (m, 4H), 1.51 (dt, *J* = 13.3, 3.7 Hz, 1H), 1.48 – 1.45 (m, 1H), 1.45 – 1.40 (m, 1H), 1.40 – 1.35 (m, 5H), 1.34 – 1.31 (m, 4H), 1.27 – 1.11 (m, 6H), 1.08 – 1.00 (m, 1H), 0.89 – 0.87 (m, 4H), 0.86 – 0.83 (m, 1H), 0.82 (s, 3H), 0.79 (s, 3H), 0.70 (dd, *J* = 12.3, 2.3 Hz, 1H), 0.66 (s, 3H), 0.65 (s, 3H), 0.60 (s, 3H).

^{13}C-NMR (176 MHz, C_6D_6): δ [ppm] = 184.6, 136.7, 131.6, 118.3, 91.1, 80.3, 79.6, 60.9, 60.7, 56.6, 55.0, 45.5, 45.5, 42.3, 40.7, 39.2, 38.6, 38.4, 38.1, 33.7, 33.6, 28.0, 25.8, 25.6, 25.3, 25.1, 22.6, 21.8, 21.3, 20.3, 19.3, 17.3, 16.2, 15.9.

IR (ν/cm^{-1}, ATR) = 2948, 2865, 1530, 1461, 1385, 1281, 1230, 1127, 1092, 970.

HRMS (ESI): *m/z* calculated for $C_{34}H_{55}N_2O_2S^+$ $[M+H]^+$: 555.3979, found 555.3988.

Mp: 199 - 202 °C.

Onoceranoxide **7**

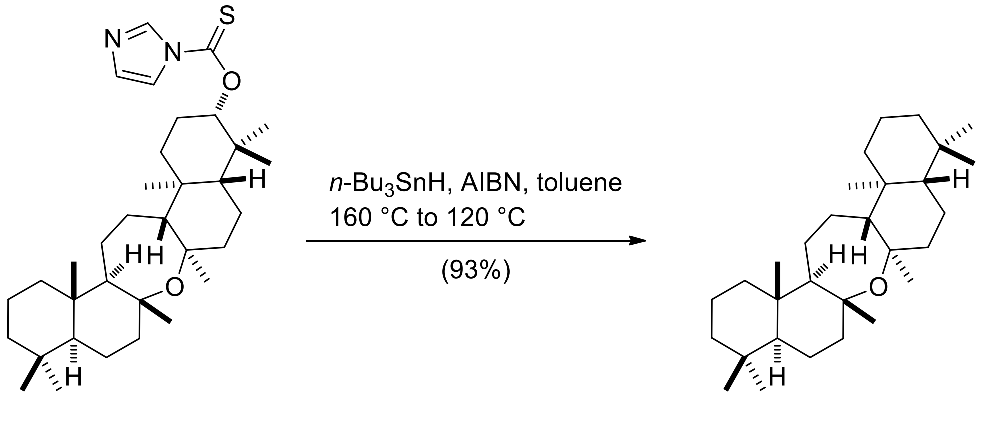

S5 | **7**

$C_{34}H_{54}N_2O_2S$ (554.87) | $C_{30}H_{52}O$ (428.73)

To a solution of thiocarbamate **S5** (6.0 mg, 11 μmol, 1.0 eq.) in toluene (4.0 mL, degassed by sparging with Ar for 20 min) at 23 °C was added *n*-Bu_3SnH (8.7 μL, 9.4 mg, 32 μmol, 3.0 eq.) and AIBN (one crystal). The reaction mixture was stirred for 10 min at 160 °C and for 20 min at 120 °C in a pressure tube. Another portion of *n*-Bu_3SnH (8.7 μL, 9.4 mg, 32 μmol, 3.0 eq.) and AIBN (one crystal) were added and the reaction mixture was stirred for 10 min at 160 °C and for 20 min at 120 °C in a pressure tube. The solvent was evaporated and the residue was directly purified by flash column chromatography (SiO_2, *n*-pentane/EtOAc 1:0 to 50:1 to 10:1) to afford onoceranoxide **7** (4.3 mg, 10 μmol, 93%) as a white solid.

$\mathbf{R_f}$ = 0.25 (*n*-pentane).

$[\alpha]_D^{26}$ = +19.7 (*c* = 0.36, $CHCl_3$) [Lit[8]:$[\alpha]_D$ = +7.9] [Lit[9]:$[\alpha]_D^{25}$ = +0.02 (*c* = 0.16, $CHCl_3$].

^{1}H-NMR (700 MHz, $CDCl_3$): δ [ppm] = 1.79 – 1.72 (m, 6H), 1.60 – 1.56 (m, 6H), 1.44 – 1.38 (m, 4H), 1.35 – 1.33 (m, 2H), 1.27 – 1.25 (m, 8H), 1.22 – 1.21 (m, 2H), 1.14 – 1.10 (m, 2H), 0.89 – 0.87 (m, 4H), 0.85 (s, 6H), 0.77 (s, 6H), 0.74 (s, 6H).

^{1}H-NMR (700 MHz, C_6D_6): δ [ppm] = 1.97 (dt, *J* = 12.7, 3.3 Hz, 2H), 1.84 – 1.71 (m, 6H), 1.63 – 1.54 (m, 4H), 1.50 – 1.46 (m, 2H), 1.42 – 1.26 (m, 12H), 1.20 – 1.11 (m, 4H), 0.89 – 0.82 (m, 10H), 0.80 (s, 6H), 0.76 (s, 6H).

^{13}C-NMR (176 MHz, $CDCl_3$): δ [ppm] = 80.1 (2C), 60.9 (2C), 56.3 (2C), 45.4 (2C), 42.2 (2C), 40.5 (2C), 39.0 (2C), 33.6 (2C), 33.6 (2C), 25.4 (2C), 25.1 (2C), 21.7 (2C), 20.9 (2C), 19.1 (2C), 15.9 (2C).

^{13}C-NMR (176 MHz, C_6D_6): δ [ppm] = 80.1 (2C), 61.3 (2C), 56.5 (2C), 45.8 (2C), 42.3 (2C), 40.7 (2C), 39.1 (2C), 33.7 (2C), 33.6 (2C), 25.7 (2C), 25.4 (2C), 21.8 (2C), 21.2 (2C), 19.3 (2C), 16.1 (2C).

IR (ν/cm^{-1}, ATR) = 2989, 2927, 2857, 1459, 1371, 1261, 1193, 1086, 1024, 970.

HRMS (ESI): *m/z* calculated for $C_{30}H_{52}ONa^+$ $[M+Na]^+$: 451.3910, found 451.3907.

Mp: 203 - 205 °C.

Note: For a previous enzymatic synthesis, see:[9]

8α-hydroxypolypoda-13,17,21 **9**

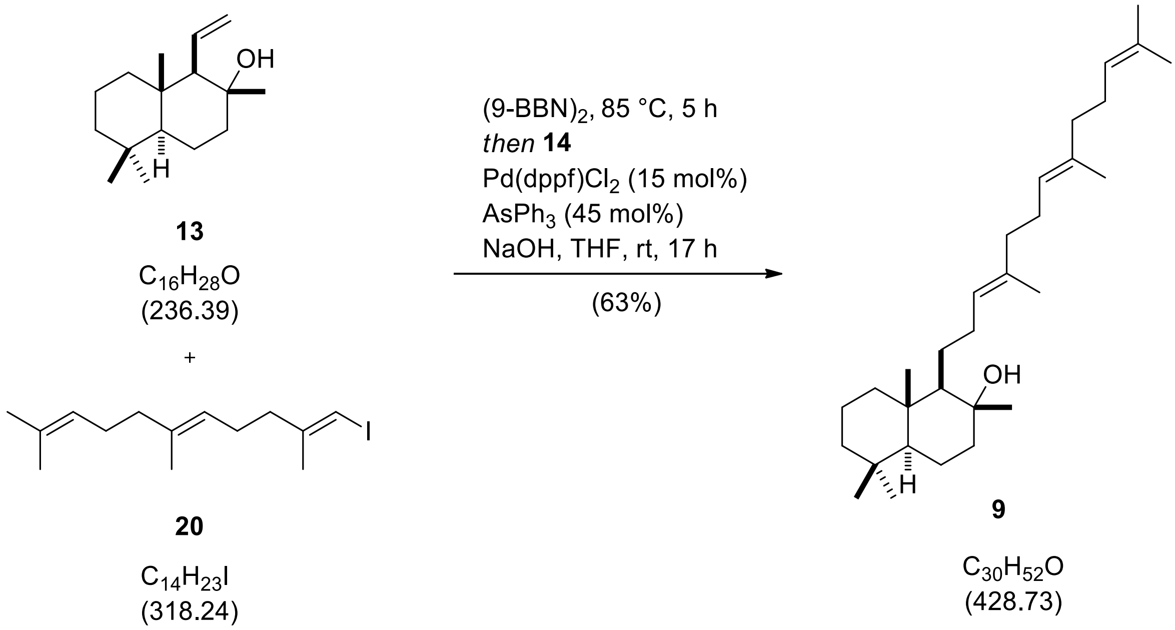

Alkene **13** (0.201 g, 850 µmol, 1.0 eq.) and 9-BBN dimer[5] (259 mg, 2.13 mmol, 2.5 eq.) were combined and stirred for 5 min, then the reaction flask was heated to 85 °C for 5 h. The reaction flask was allowed to cool to 23 °C and THF (10 mL, degassed by freeze pump thaw 3x) and NaOH (1.70 mL, 3 M, 5.10 mmol, 6.0 eq., purged with Ar for 20 min) was added. A solution of vinyl iodide **20** (547 mg, 1.72 mmol, 2.0 eq.) and $AsPh_3$ (117 mg, 0.383 µmol, 45 mol%) in THF (4 mL, purged with Ar for 20 min, the flask was rinsed 2x with 0.5 mL) was added to the reaction mixture. The reaction mixture was purged with Ar for 5 min. $Pd(dppf)Cl_2$ (93.3 mg, 128 µmol, 15 mol%) was added and the reaction mixture was stirred at 23 °C for 17 h. Water and EtOAc were added to the reaction mixture sequentially. The organic phase was separated and the aqueous phase was extracted with EtOAc. The combined organic extracts were washed with brine, dried over $MgSO_4$, filtered and concentrated under reduced pressure. The residue was purified by flash column chromatography (SiO_2, *n*-pentane/EtOAc 50:1 to 10:1) to afford triene **9** (229 mg, 534 µmol, 63%) as a slightly yellow oil.

$\mathbf{R_f}$ = 0.54 (*n*-pentane/EtOAc, 19:1).

$[\alpha]_D^{25}$ = −0.39 (*c* = 0.77, $CHCl_3$), [Lit[10]:$[\alpha]_D^{23}$ = −0.9 (*c* = 0.4, $CHCl_3$)], [Lit[11]:$[\alpha]_D^{24}$ = −0.6 (*c* = 0.75, $CHCl_3$)], [Lit[12]:$[\alpha]_D^{23}$ = −1.8 (*c* = 0.1, $CHCl_3$)].

^{1}H-NMR (500 MHz, $CDCl_3$): δ [ppm] = 5.21 – 5.16 (m, 1H), 5.14 – 5.06 (m, 2H), 2.12 – 2.03 (m, 6H), 2.02 – 1.93 (m, 4H), 1.86 (dt, *J* = 12.1, 3.1 Hz, 1H), 1.68 (s, 3H), 1.67 – 1.62 (m, 1H), 1.61 (s, 3H), 1.60 (s, 6H), 1.58 – 1.54 (m, 1H), 1.48 – 1.14 (m, 9H), 1.13 (s, 3H), 1.03 (t, *J* =

4.0 Hz, 1H), 0.98 (td, J = 13.0, 3.7 Hz, 1H), 0.91 (dd, J = 12.1, 2.3 Hz, 1H), 0.87 (s, 3H), 0.79 (s, 6H).

^{13}C-NMR (126 MHz, $CDCl_3$): δ [ppm] = 135.3, 135.1, 131.4, 125.2, 124.6, 124.4, 74.2, 61.7, 56.3, 44.7, 42.2, 39.9 (3C), 39.3, 33.6, 33.4, 31.6, 26.9, 26.8, 25.9, 25.7, 23.9, 21.7, 20.7, 18.6, 17.8, 16.4, 16.2, 15.6.

IR (ν/cm^{-1}, ATR) = 3424, 2925, 2860, 1450, 1383, 1157, 1080, 934, 822, 802.

HRMS (ESI): *m/z* calculated for $C_{30}H_{52}ONa^+$ $[M+Na]^+$: 451.3910, found 451.3931.

Note: **9** was isolated from fresh rhizomes of *Polypodiodes formosana* in 1992.[10] **9** was synthesized in 3 steps in the longest linear synthesis from (+)-sclareolide with an overall yield of 28% and was used as a tricyclization substrate. However, no productive cyclization could be detected under a variety of conditions. For selected conditions see Section 3.

For a previous 13 step racemic synthesis, see:[13].

For a previous enzymatic synthesis, see:[9, 12].

For a previous 10 step asymmetric synthesis, see:[11].

Compound **S6**

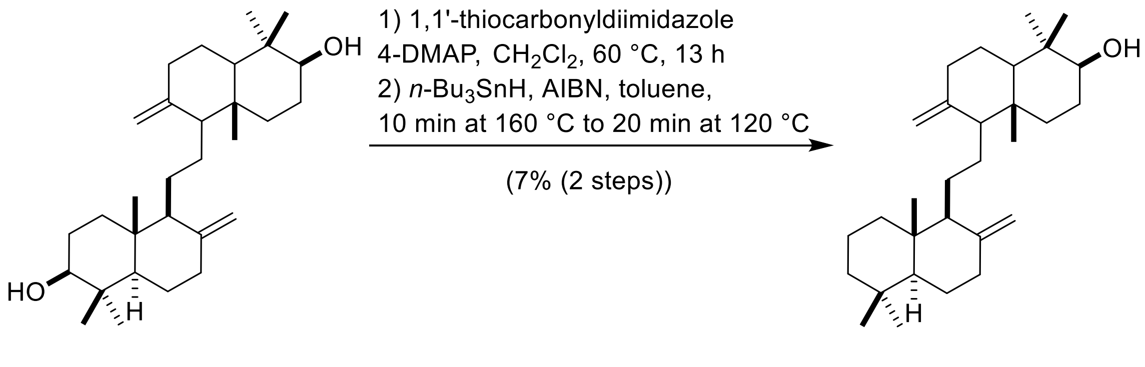

α-onocerin $C_{30}H_{50}O_2$ (442.72)

S6 $C_{30}H_{50}O$ (426.72)

To a solution of α-onocerin (20.0 mg, 45.2 µmol, 1.0 eq.) in CH_2Cl_2 (4.0 mL) at 23 °C was added *N*,*N*-dimethyl-4-aminopyridine (11.0 mg, 90.4 µmol, 2.0 eq.) and 1,1'-thiocarbonyldiimidazole (16.1 mg, 90.4 µmol, 2.0 eq.). Then the reaction mixture was stirred for 13 h at 60 °C in a closed vial. The residue was directly purified by flash chromatography (SiO_2, *n*-pentane/EtOAc 30:1 to 4:1 to 2:1) to afford an inseparable mixture (17.5 mg). An aliquot of the obtained material (6.2 mg) was dissolved in toluene (degassed by sparging with Ar for 20 min, 4 mL) in a pressure tube and AIBN (one crystal) and Bu_3SnH (9.10 µL, 33.6 µmol, 0.74 eq.) were added successively. The reaction mixture was stirred at 160 °C for 10 min and at 120 °C for 20 min. The residue was directly purified by flash column chromatography (SiO_2, *n*-pentane/EtOAc 4:1) to afford alcohol **S6** (1.3 mg, 3.05 µmol, 7%) as a white solid.

R_f = 0.81 (*n*-pentane/EtOAc, 4:1).

$[\alpha]^{23}_{D}$ = +11.6 (*c* = 0.48, $CHCl_3$) [Lit[14]: **$[\alpha]_D$** = +21°(*c* = 1.70, $CHCl_3$)].

^{1}H-NMR (700 MHz, $CDCl_3$): δ [ppm] = δ 4.87 – 4.77 (m, 2H), 4.60 – 4.49 (m, 2H), 3.25 (dd, *J* = 11.8, 4.3 Hz, 1H), 2.40 (dddd, *J* = 13.0, 11.1, 4.3, 2.4 Hz, 2H), 1.98 (td, *J* = 12.9, 5.0 Hz, 2H), 1.76 – 1.65 (m, 5H), 1.60 – 1.07 (m, 17H), 0.99 (s, 3H), 0.94 (td, *J* = 12.9, 3.8 Hz, 1H), 0.87 (s, 3H), 0.79 (s, 3H), 0.76 (s, 3H), 0.64 (s, 3H), 0.63 (s, 3H).

^{13}C-NMR (176 MHz, $CDCl_3$): δ [ppm] = 149.3, 148.6, 106.8, 106.4, 79.1, 58.1, 57.8, 55.8, 54.8, 42.4, 39.7, 39.4, 39.3, 39.2, 38.6, 38.4, 37.2, 33.8 (2C), 28.5, 28.1, 24.7, 24.2, 22.9, 22.7, 21.9, 19.6, 15.5, 14.7 (2C).

IR (ν/cm^{-1}, ATR) = 3350, 3082, 2932, 2846, 1460, 1385, 1227, 1182, 1032, 886.

HRMS (ESI): *m/z* calculated for $C_{30}H_{50}ONa^+$ $[M+Na]^+$: 449.3754, found 449.3739.

Mp: 178 – 180 °C [Lit[14]: 182 – 184].

Note: This procedure is unoptimized. α-Onocerin was extracted from spiny restharrow roots.[15]

Compound **S6** is used as a GC-MS reference to get insight into the structure of the elimination product of the BmeTC catalyzed reaction with **12**. See section 6.

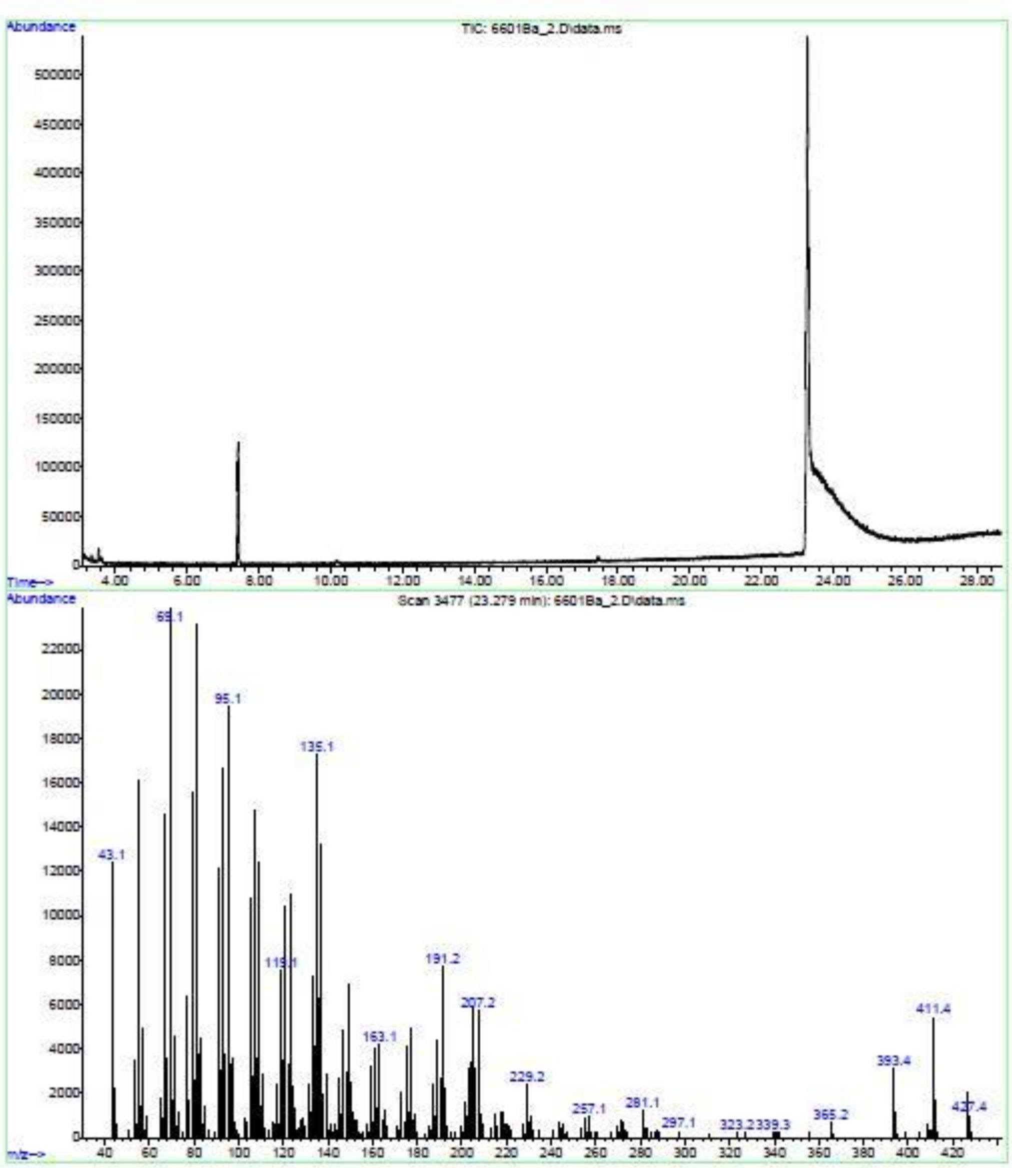

Fig. 1 GC-MS Data for **S6**.

GC/MS system consisting of a 5977E MSD single-quadrupole mass spectrometer (EI-Mode (70 eV)) with a 7820A GC by Agilent Technologies (Agilent 190915-433UI, 30 m x 250 μm x 0.25 μm). Injection temperature: 300 °C, column temperature: 220-300 °C (increment 3 °C/min).

Optimization of the Wittig-type fragmentation: Synthesis of the Ethers S8 - S21

Compound **S7**

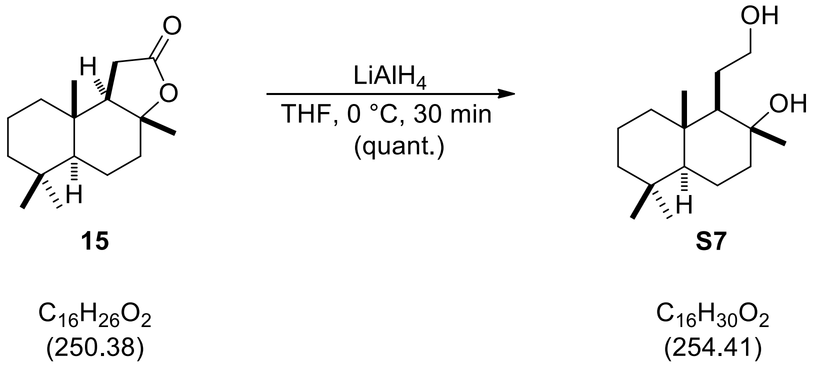

To a stirred suspension of $LiAlH_4$ (823 mg, 21.7 mmol, 1.0 eq.) in THF (40 mL) at 0 °C was added a solution of lactone **15** (5.41 g, 21.6 mmol, 1.0 eq.) in THF (20 mL, flask rinsed with 2 x 20 mL) over 10 min. After stirring for 30 min, KOH (10 mL, 2 M aq.) was added dropwise. The mixture was diluted with CH_2Cl_2. The organic phase was separated and the aqueous phase was extracted with CH_2Cl_2. The combined organic extracts were washed with brine, dried over $MgSO_4$, filtered and concentrated under reduced pressure. Diol **S7** (5.49 g, 21.6 mmol, quant.) was obtained as a white solid and was used without further purification.

R*f* = 0.12 (*n*-pentane/EtOAc 3:1).

$[\alpha]_D^{23}$ = −16.9° (*c* = 1.05, $CHCl_3$).

^{1}H-NMR (500 MHz, $CDCl_3$): δ [ppm] = 3.79 (dt, *J* = 10.1, 4.3 Hz, 1H), 3.47 (ddd, *J* = 10.1, 8.2, 5.6 Hz, 1H), 2.75 (s, 2H), 1.90 (dt, *J* = 12.4, 3.2 Hz, 1H), 1.71 – 1.62 (m, 4H), 1.57 (tt, *J* = 13.5, 3.5 Hz, 1H), 1.53 – 1.40 (m, 2H), 1.40 – 1.34 (m, 1H), 1.33 – 1.25 (m, 2H), 1.20 (s, 3H), 1.14 (td, *J* = 13.4, 4.2 Hz, 1H), 0.98 – 0.92 (m, 2H), 0.87 (s, 3H), 0.79 (s, 6H).

^{13}C-NMR (126 MHz, $CDCl_3$): δ [ppm] = 73.4, 64.3, 59.3, 56.2, 44.4, 42.1, 39.5, 39.1, 33.6, 33.4, 28.0, 24.8, 21.6, 20.6, 18.6, 15.5.

IR (ν/cm^{-1}, ATR) = 3267, 2923, 2867, 1461, 1386, 1189, 1085, 1051, 935, 727.

HRMS (ESI): *m/z* calculated for $C_{16}H_{30}O_2Na^+$ $[M+Na]^+$: 277.2138, found 277.2147.

Mp = 128 – 130 °C.

The spectral data matched previously obtained data.[16]

Compound **S8**

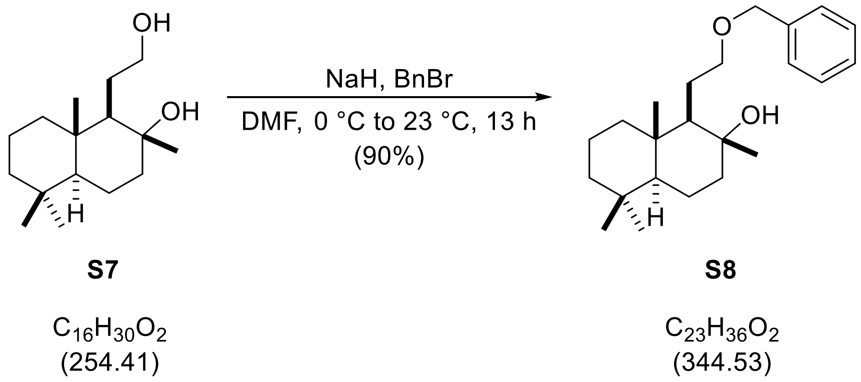

To a stirred suspension of sodium hydride (60% in mineral oil, 1.13 g, 28.3 mmol, 1.3 eq.) in DMF (30 mL) at 0 °C was added a solution of diol **S7** (5.46 g, 21.5 mmol, 1.0 eq.) in DMF (20 mL, flask rinsed with 2 x 5 mL). After 30 min benzyl bromide (3.83 mL, 5.51 g, 32.2 mmol, 1.5 eq.) was added and the mixture was allowed to warm to 23 °C over 13 h. Water and EtOAc were added to the reaction mixture sequentially. The organic phase was separated and the aqueous phase was extracted with EtOAc. The combined organic extracts were washed repeatedly with brine, dried over $MgSO_4$, filtered and concentrated under reduced pressure. The residue was purified by flash column chromatography (SiO_2, *n*-pentane/EtOAc 20:1 to 10:1) to afford ether **S8** (6.65 g, 19.3 mmol, 90%) as a colorless oil.

$\mathbf{R}_f$ = 0.88 (*n*-pentane/EtOAc 3:1).

$[\alpha]_D^{23}$ = −16.1° (*c* = 0.88, $CHCl_3$).

^{1}**H-NMR** (700 MHz, $CDCl_3$): δ [ppm] = 7.38 – 7.30 (m, 4H), 7.30 – 7.25 (m, 1H), 4.53 (d, *J* = 1.9 Hz, 2H), 3.63 (ddd, *J* = 9.2, 5.4, 4.1 Hz, 1H), 3.37 (ddd, *J* = 10.2, 8.8, 4.3 Hz, 1H), 3.32 (s, 1H), 1.90 (dt, *J* = 12.6, 3.2 Hz, 1H), 1.80 – 1.74 (m, 1H), 1.65 (ddd, *J* = 13.8, 6.1, 3.7 Hz, 1H), 1.64 – 1.53 (m, 3H), 1.46 – 1.33 (m, 3H), 1.30 – 1.24 (m, 1H), 1.24 – 1.20 (m, 1H), 1.15 – 1.14 (m, 3H), 1.12 (dd, *J* = 13.5, 4.2 Hz, 1H), 0.91 (dd, *J* = 12.3, 2.3 Hz, 1H), 0.87 (s, 3H), 0.85 (dd, *J* = 13.0, 3.7 Hz, 1H), 0.79 (s, 6H).

^{13}**C-NMR** (176 MHz, $CDCl_3$): δ [ppm] = 138.0, 128.6 (2C), 127.8 (3C), 73.3, 72.4, 72.2, 59.4, 56.2, 44.2, 42.1, 39.6, 39.1, 33.6, 33.4, 25.4, 24.5, 21.6, 20.6, 18.6, 15.4.

IR (ν/cm^{-1}, ATR) = 3438, 2925, 2863, 1457, 1384, 1366, 1076, 930, 910, 732.

HRMS (ESI): *m/z* calculated for $C_{23}H_{36}O_2K^+$ $[M+K]^+$: 383.2347, found 383.2356.

The spectral data matched previously obtained data.[17]

Compound **S9**

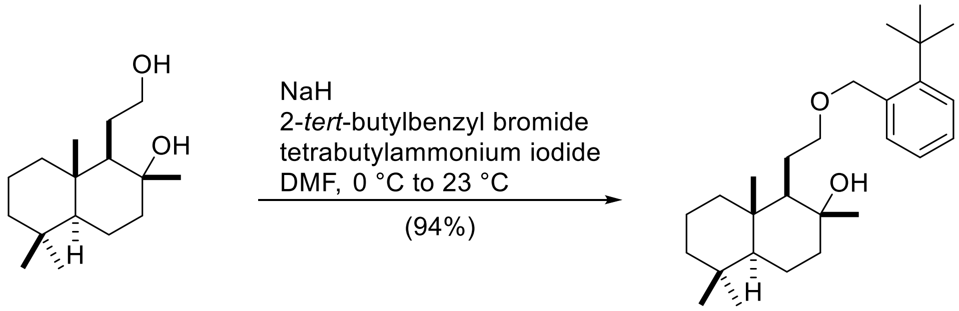

S7
$C_{16}H_{30}O_2$
(254.41)

S9
$C_{27}H_{44}O_2$
(400.64)

To a stirred solution of diol **S7** (256 mg, 1.01 mmol, 1.0 eq.) in DMF (4 mL) was added sodium hydride (60% in mineral oil, 60.3 mg, 1.51 mmol, 1.5 eq.) at 0 °C. After 30 min tetrabutylammonium iodide (18.6 mg, 50.3 μmol, 0.05 eq.) and 2-*tert*-butylbenzyl bromide (341 mg, 1.51 mmol, 1.5 eq.) in DMF (2.0 mL) were added and the mixture was allowed to warm to 23 °C over 20 h. Water and EtOAc were added to the reaction mixture sequentially. The organic phase was separated and the aqueous phase was extracted with EtOAc. The combined organic extracts were washed repeatedly with brine, dried over $MgSO_4$, filtered and concentrated under reduced pressure. The residue was purified by flash column chromatography (SiO_2, *n*-pentane/EtOAc 1:0 to 5:1) to afford ether **S9** (381 mg, 950 μmol, 94%) as a colorless oil.

$\mathbf{R}_f$ = 0.39 (*n*-pentane/EtOAc, 9:1).

$[\alpha]_D^{27}$ = −3.68 (*c* = 1.34, $CHCl_3$).

^{1}H-NMR (700 MHz, $CDCl_3$): δ [ppm] = 7.48 – 7.45 (m, 1H), 7.39 – 7.36 (m, 1H), 7.23 – 7.17 (m, 2H), 4.83 – 4.68 (m, 2H), 3.70 – 3.65 (m, 1H), 3.46 – 3.40 (m, 1H), 3.19 (s_{br}, 1H), 1.89 (dt, *J* = 12.6, 3.3 Hz, 1H), 1.82 – 1.76 (m, 1H), 1.67 – 1.56 (m, 4H), 1.43 – 1.40 (m, 10H), 1.39 – 1.36 (m, 1H), 1.29 – 1.22 (m, 2H), 1.18 – 1.15 (m, 1H), 1.14 (s, 3H), 0.94 – 0.90 (m, 2H), 0.88 (s, 3H), 0.83 – 0.80 (m, 1H), 0.80 (s, 6H).

^{13}C-NMR (176 MHz, $CDCl_3$): δ [ppm] = 147.9, 136.3, 131.1, 127.7, 126.3, 126.2, 72.5, 72.5, 71.9, 59.4, 56.3, 44.3, 42.1, 39.7, 39.2, 35.9, 33.6, 33.4, 32.0 (3C), 25.5, 24.5, 21.7, 20.6, 18.6, 15.4.

IR (ν/cm^{-1}, ATR) = 3453, 2932, 2867, 1463, 1386, 1363, 1248, 1188, 1074, 934.

HRMS (ESI): *m/z* calculated for $C_{27}H_{44}O_2Na^+$ $[M+Na]^+$: 423.3233, found 423.3240.

Compound **S10**

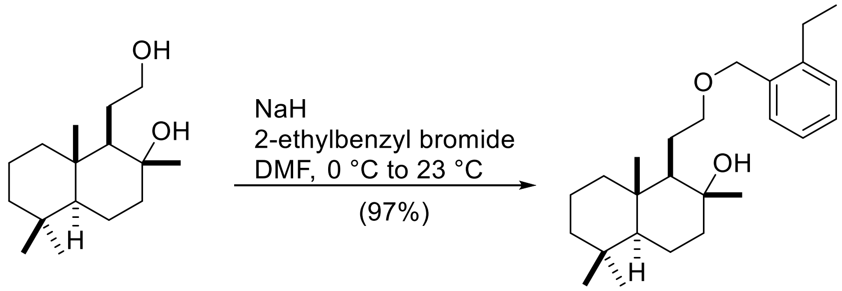

S7

$C_{16}H_{30}O_2$
(254.41)

S10

$C_{25}H_{40}O_2$
(372.58)

To a stirred suspension of sodium hydride (60% in mineral oil, 62.9 mg, 1.57 mmol, 2.0 eq.) in DMF (3 mL) at 0 °C was added a solution of diol **S7** (200 mg, 786 μmol, 1.0 eq.) in DMF (3 mL). After 30 min 2-ethylbenzyl bromide (221 mg, 1.11 mmol, 1.4 eq.) in DMF (3 mL) was added and the mixture was allowed to warm to 23 °C over 13 h. Water and EtOAc were added to the reaction mixture sequentially. The organic phase was separated and the aqueous phase was extracted with EtOAc. The combined organic extracts were washed repeatedly with brine, dried over $MgSO_4$, filtered and concentrated under reduced pressure. The residue was purified by flash column chromatography (SiO_2, *n*-pentane/EtOAc 20:1 to 5:1) to afford ether **S10** (283 mg, 759 μmol, 97%) as a slightly yellow oil.

R*f* = 0.61 (*n*-pentane/EtOAc, 6:1).

$[\alpha]_D^{27}$ = −16.1 (*c* = 0.62, $CHCl_3$).

^{1}H-NMR (500 MHz, $CDCl_3$): δ [ppm] = 7.31 (d, *J* = 7.5 Hz, 1H), 7.25 – 7.14 (m, 3H), 4.65 – 4.48 (m, 2H), 3.66 – 3.60 (m, 1H), 3.38 (td, *J* = 9.3, 4.5 Hz, 1H), 3.20 (s_{br}, 1H), 2.69 (q, *J* = 7.6 Hz, 2H), 1.89 (dt, *J* = 12.5, 3.3 Hz, 1H), 1.81 – 1.72 (m, 1H), 1.69 – 1.53 (m, 4H), 1.45 – 1.34 (m, 3H), 1.28 – 1.20 (m, 5H), 1.17 – 1.10 (m, 4H), 0.94 – 0.89 (m, 1H), 0.89 – 0.85 (m, 4H), 0.79 (s, 6H).

^{13}C-NMR (126 MHz, $CDCl_3$): δ [ppm] = 142.7, 135.3, 129.1, 128.6, 128.2, 125.9, 72.5, 72.2, 71.2, 59.4, 56.3, 44.2, 42.1, 39.7, 39.2, 33.6, 33.4, 25.5, 25.4, 24.5, 21.6, 20.6, 18.6, 15.4, 15.4.

IR (ν/cm^{-1}, ATR) = 3439, 2930, 2867, 1458, 1384, 1183, 1086, 935, 843, 756.

HRMS (ESI): *m/z* calculated for $C_{25}H_{40}O_2Na^+$ $[M+Na]^+$: 395.2921, found 395.2938.

Compound **S11**

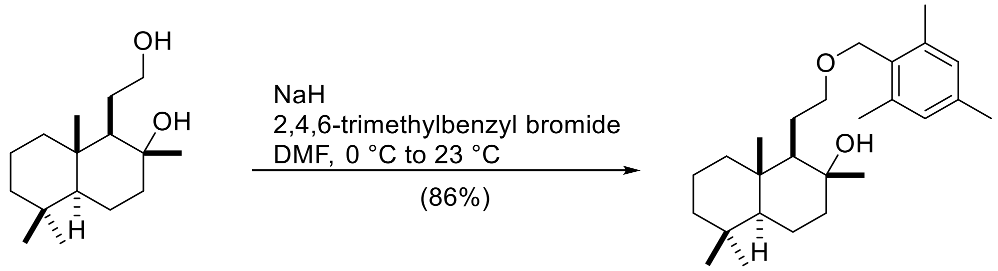

S7 $C_{16}H_{30}O_2$ (254.41)

S11 $C_{26}H_{42}O_2$ (386.61)

To a stirred suspension of sodium hydride (60% in mineral oil, 299 mg, 7.48 mmol, 2.0 eq.) in DMF (7 mL) at 0 °C was added a solution of diol **S7** (951 mg, 3.74 mmol, 1.0 eq.) in DMF (7 mL). After 30 min 2,4,6-trimethylbenzyl bromide (1.19 g, 5.61 mmol, 1.5 eq.) in DMF (6 mL) was added and the mixture was allowed to warm to 23 °C over 22 h. Water and EtOAc were added to the reaction mixture sequentially. The organic phase was separated and the aqueous phase was extracted with EtOAc. The combined organic extracts were washed repeatedly with brine, dried over $MgSO_4$, filtered and concentrated under reduced pressure. The residue was purified by flash column chromatography (SiO_2, *n*-pentane/EtOAc 30:1 to 10:1) to afford ether **S11** (1.24 g, 3.20 mmol, 86%) as a slightly yellow oil.

R*f* = 0.56 (*n*-pentane/EtOAc, 6:1).

$[\alpha]_D^{27}$ = −14.9 (*c* = 3.98, $CHCl_3$).

^{1}H-NMR (500 MHz, $CDCl_3$): δ [ppm] = 6.83 (s, 2H), 4.53 (s, 2H), 3.69 – 3.56 (m, 1H), 3.47 – 3.15 (m, 2H), 2.36 (s, 6H), 2.25 (s, 3H), 1.88 (dt, *J* = 12.5, 3.2 Hz, 1H), 1.76 – 1.53 (m, 5H), 1.45 – 1.35 (m, 3H), 1.29 – 1.18 (m, 2H), 1.18 – 1.13 (m, 1H), 1.10 (s, 3H), 0.92 (d, *J* = 2.3 Hz, 1H), 0.89 – 0.85 (m, 4H), 0.79 (s, 3H), 0.78 (s, 3H).

^{13}C-NMR (126 MHz, $CDCl_3$): δ [ppm] = 137.8 (2C), 137.6, 131.0, 129.1 (2C), 72.2, 71.8, 67.2, 59.5, 56.3, 44.2, 42.1, 39.7, 39.1, 33.6, 33.4, 25.6, 24.6, 21.6, 21.1, 20.6, 19.8 (2C), 18.6, 15.4.

IR (ν/cm^{-1}, ATR) = 3439, 2925, 2864, 1613, 1459, 1383, 1235, 1079, 935, 849.

HRMS (ESI): *m/z* calculated for $C_{26}H_{42}O_2Na^+$ $[M+Na]^+$: 409.3077, found 409.3097.

Compound **S12**

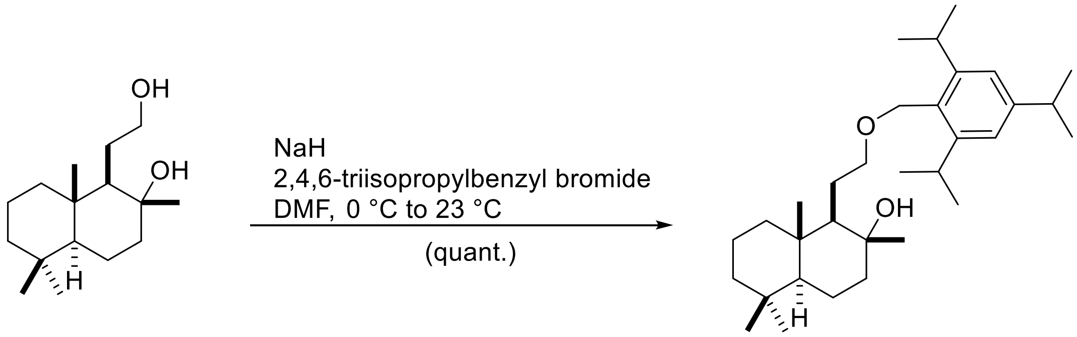

S7

$C_{16}H_{30}O_2$
(254.41)

S12

$C_{32}H_{54}O_2$
(470.77)

To a stirred suspension of sodium hydride (60% in mineral oil, 281 mg, 7.02 mmol, 2.0 eq.) in DMF (7 mL) at 0 °C was added a solution of diol **S7** (893 mg, 3.51 mmol, 1.0 eq.) in DMF (7 mL). After 30 min 2,4,6-triisopropylbenzyl bromide (1.50 g, 5.05 mmol, 1.4 eq.) in DMF (6 mL) was added and the mixture was allowed to warm to 23 °C over 12 h. Water and EtOAc were added to the reaction mixture sequentially. The organic phase was separated and the aqueous phase was extracted with EtOAc. The combined organic extracts were washed repeatedly with brine, dried over $MgSO_4$, filtered and concentrated under reduced pressure. The residue was purified by flash column chromatography (SiO_2, *n*-pentane/EtOAc 20:1 to 10:1) to afford ether **S12** (1.69 g, 3.60 mmol, quant.) as a slightly yellow oil.

$\mathbf{R}_f$ = 0.35 (*n*-pentane/EtOAc, 19:1).

$[\alpha]_D^{27}$ = −20.9 (*c* = 1.97, $CHCl_3$).

^{1}H-NMR (500 MHz, $CDCl_3$): δ [ppm] = 7.00 (s, 2H), 4.60 – 4.50 (m, 2H), 3.71 – 3.64 (m, 1H), 3.46 – 3.39 (m, 1H), 3.28 (hept, *J* = 6.8 Hz, 2H), 3.06 (s_{br}, 1H), 2.87 (hept, *J* = 6.9 Hz, 1H), 1.86 (dt, *J* = 12.4, 3.1 Hz, 1H), 1.80 – 1.70 (m, 1H), 1.69 – 1.57 (m, 4H), 1.47 – 1.33 (m, 4H), 1.29 – 1.23 (m, 19H), 1.19 – 1.13 (m, 1H), 1.10 (s, 3H), 0.97 – 0.90 (m, 2H), 0.89 (s, 3H), 0.80 (s, 6H).

^{13}C-NMR (126 MHz, $CDCl_3$): δ [ppm] = 148.8, 148.5 (2C), 128.7, 121.0 (2C), 72.3, 72.1, 65.5, 59.1, 56.4, 44.3, 42.2, 39.8, 39.2, 34.5, 33.6, 33.4, 29.5 (2C), 25.6, 24.7, 24.6 (2C), 24.5 (2C), 24.1 (2C), 21.6, 20.6, 18.6, 15.4.

IR (ν/cm^{-1}, ATR) = 3443, 2957, 2929, 2868, 1607, 1460, 1384, 1074, 877, 753.

HRMS (ESI): *m/z* calculated for $C_{32}H_{54}O_2Na^+$ $[M+Na]^+$: 493.4016, found 493.4041.

Compound **S13**

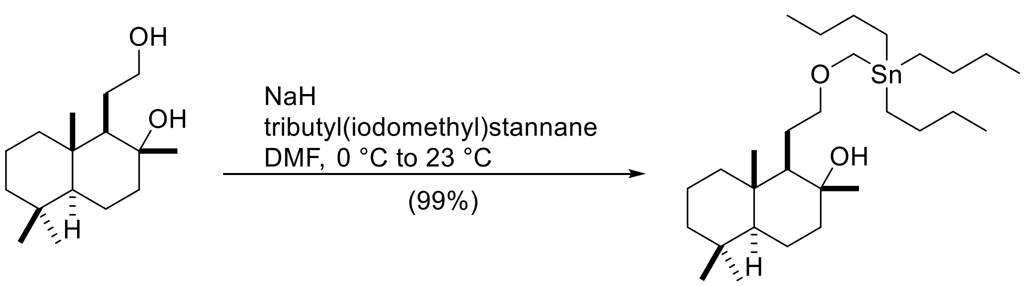

S7
$C_{16}H_{30}O_2$
(254.41)

S13
$C_{29}H_{58}O_2Sn$
(557.48)

To a stirred suspension of sodium hydride (60% in mineral oil, 191 mg, 4.77 mmol, 2.0 eq.) in DMF (5 mL) at 0 °C was added a solution of diol **S7** (607 mg, 2.38 mmol, 1.0 eq.) in DMF (5 mL). After 30 min tributyl(iodomethyl)stannane (5.52 g, 3.46 mmol, 1.5 eq.) in DMF (5 mL) was added and the mixture was allowed to warm to 23 °C over 24 h. Water and EtOAc were added to the reaction mixture sequentially. The organic phase was separated and the aqueous phase was extracted with EtOAc. The combined organic extracts were washed repeatedly with brine, dried over $MgSO_4$, filtered and concentrated under reduced pressure. The residue was purified by flash column chromatography (SiO_2, *n*-pentane/EtOAc 1:0 to 10:1) to afford ether **S13** (1.32 g, 2.37 mmol, 99%) as a slightly yellow oil.

$\mathbf{R_f}$ = 0.61 (*n*-pentane/EtOAc, 19:1).

$[\alpha]_D^{27}$ = −16.8 (*c* = 1.88, $CHCl_3$).

^{1}H-NMR (500 MHz, $CDCl_3$): δ [ppm] = 3.78 – 3.67 (m, 2H), 3.62 (s_{br}, 1H), 3.49 (dt, *J* = 8.6, 4.2 Hz, 1H), 3.24 – 3.16 (m, 1H), 1.89 (dt, *J* = 12.6, 3.2 Hz, 1H), 1.74 – 1.59 (m, 4H), 1.59 – 1.45 (m, 8H), 1.45 – 1.34 (m, 4H), 1.34 – 1.27 (m, 6H), 1.27 – 1.21 (m, 2H), 1.15 (dd, *J* = 13.4, 4.0 Hz, 1H), 1.12 (s, 3H), 0.93 – 0.86 (m, 18H), 0.79 (s, 3H), 0.78 (s, 3H).

^{13}C-NMR (126 MHz, $CDCl_3$): δ [ppm] = 77.5, 72.0, 62.4, 59.6, 56.3, 44.1, 42.1, 39.8, 39.1, 33.6, 33.4, 29.3 (3C), 27.5 (3C), 25.4, 24.6, 21.7, 20.6, 18.7, 15.4, 13.9 (3C), 9.1 (3C).

IR (ν/cm^{-1}, ATR) = 3437, 2951, 2923, 2849, 1460, 1381, 1339, 1287, 1073, 934.

HRMS (ESI): *m/z* calculated for $C_{29}H_{58}O_2SnNa^+$ $[M+Na]^+$: 581.3351, found 581.3339.

Compound **S14**

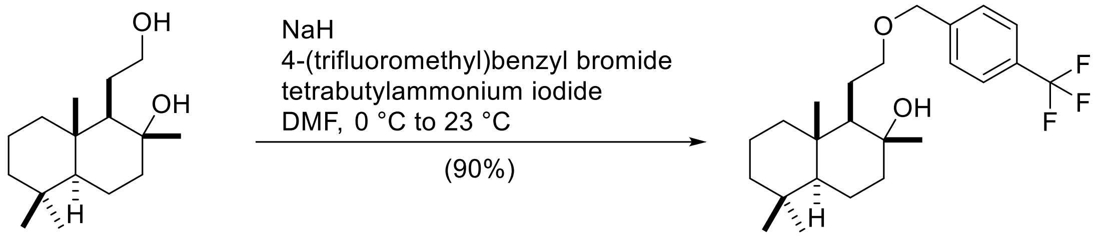

S7 $C_{16}H_{30}O_2$ (254.41)

S14 $C_{24}H_{35}F_3O_2$ (412.53)

To a stirred solution of diol **S7** (132 mg, 518 μmol, 1.0 eq.) in DMF (2.5 mL) was added sodium hydride (60% in mineral oil, 26.9 mg, 673 μmol, 1.3 eq.) at 0 °C. After 30 min tetrabutylammonium iodide (9.6 mg, 26 μmol, 0.05 eq.) and 4-(trifluoromethyl)benzyl bromide (173 mg, 725 μmol, 1.4 eq.) in DMF (0.5 mL) were added and the mixture was allowed to warm to 23 °C over 22 h. Water and EtOAc were added to the reaction mixture sequentially. The organic phase was separated and the aqueous phase was extracted with EtOAc. The combined organic extracts were washed repeatedly with brine, dried over $MgSO_4$, filtered and concentrated under reduced pressure. The residue was purified by flash column chromatography (SiO_2, *n*-pentane/EtOAc 1:0 to 5:1) to afford ether **S14** (192 mg, 466 μmol, 90%) as a colorless oil.

$\mathbf{R}_f$ = 0.40 (*n*-pentane/EtOAc, 5:1).

$[\alpha]_D^{26}$ = −18.3 (*c* = 0.85, $CHCl_3$).

^{1}H-NMR (700 MHz, $CDCl_3$): δ [ppm] = 7.60 (d, *J* = 8.0 Hz, 2H), 7.44 (d, *J* = 8.0 Hz, 2H), 4.58 (s, 2H), 3.68 – 3.61 (m, 1H), 3.45 – 3.39 (m, 1H), 2.99 (s_{br}, 1H), 1.90 (dt, *J* = 12.6, 3.2 Hz, 1H), 1.80 (ddt, *J* = 15.2, 10.2, 5.3 Hz, 1H), 1.69 – 1.54 (m, 4H), 1.45 – 1.35 (m, 3H), 1.29 – 1.22 (m, 1H), 1.21 (t, *J* = 4.3 Hz, 1H), 1.17 – 1.14 (m, 3H), 1.12 (dd, *J* = 13.5, 4.2 Hz, 1H), 0.91 (dd, *J* = 12.2, 2.3 Hz, 1H), 0.90 – 0.84 (m, 4H), 0.79 (s, 3H), 0.79 (s, 3H).

^{13}C-NMR (176 MHz, $CDCl_3$): δ [ppm] = 142.2, 130.0 (q, *J* = 32.4 Hz), 127.8 (2C), 125.6 (q, *J* = 3.7 Hz, 2C), 125.1 (q, *J* = 272.2 Hz), 72.8, 72.7, 72.5, 59.4, 56.3, 44.3, 42.1, 39.7, 39.2, 33.5, 33.4, 25.4, 24.5, 21.6, 20.6, 18.6, 15.4.

^{19}F-NMR (471 MHz, $CDCl_3$): δ [ppm] = −62.4.

IR (ν/cm^{-1}, ATR) = 3455, 2930, 2865, 1622, 1462, 1387, 1325, 1164, 1125, 1070.

HRMS (ESI): *m/z* calculated for $C_{24}H_{35}F_3O_2Na^+$ $[M+Na]^+$: 435.2481, found 435.2490.

Compound **S15**

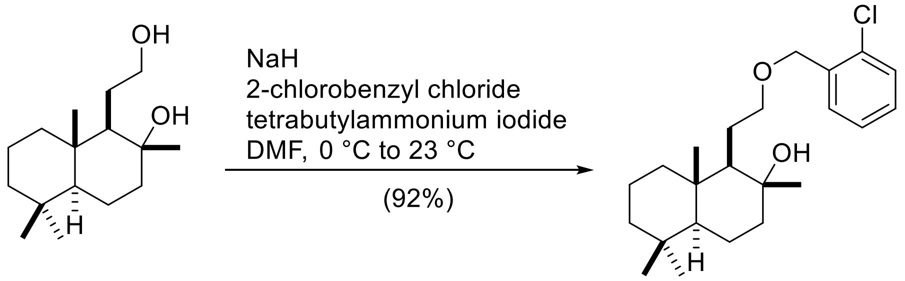

S7 **S15**

$C_{16}H_{30}O_2$ (254.41) $C_{23}H_{35}ClO_2$ (378.98)

To a stirred solution of diol **S7** (301 mg, 1.18 mmol, 1.0 eq.) in DMF (4.0 mL) was added sodium hydride (60% in mineral oil, 70.8 mg, 1.77 mmol, 1.5 eq.) at 0 °C. After 30 min tetrabutylammonium iodide (43.7 mg, 118 μmol, 0.1 eq.) and 2-chlorobenzyl chloride (268 μL, 286 mg, 1.77 mmol, 1.5 eq.) were added and the mixture was allowed to warm to 23 °C over 2 h. Water and EtOAc were added to the reaction mixture sequentially. The organic phase was separated and the aqueous phase was extracted with EtOAc. The combined organic extracts were washed repeatedly with brine, dried over $MgSO_4$, filtered and concentrated under reduced pressure. The residue was purified by flash column chromatography (SiO_2, *n*-pentane/EtOAc 1:0 to 5:1) to afford ether **S15** (413 mg, 1.09 mmol, 92%) as a colorless oil.

R*f* = 0.52 (*n*-pentane/EtOAc, 5:1).

$[\alpha]_D^{27}$ = −20.6 (*c* = 1.03, $CHCl_3$).

^{1}H-NMR (700 MHz, $CDCl_3$): δ [ppm] = 7.44 (dd, *J* = 7.6, 1.6 Hz, 1H), 7.35 – 7.32 (m, 1H), 7.27 – 7.24 (m, 1H), 7.20 (td, *J* = 7.6, 1.7 Hz, 1H), 4.62 (s, 2H), 3.75 – 3.61 (m, 1H), 3.50 – 3.37 (m, 1H), 3.05 (s_{br}, 1H), 1.90 (dt, *J* = 12.6, 3.3 Hz, 1H), 1.80 (ddt, *J* = 15.2, 10.2, 5.2 Hz, 1H), 1.67 – 1.54 (m, 4H), 1.45 – 1.34 (m, 3H), 1.29 – 1.21 (m, 2H), 1.14 (s, 3H), 1.12 (dd, *J* = 13.6, 4.1 Hz, 1H), 0.91 (dd, *J* = 12.2, 2.3 Hz, 1H), 0.90 – 0.84 (m, 4H), 0.79 (s, 3H), 0.79 (s, 3H).

^{13}C-NMR (176 MHz, $CDCl_3$): δ [ppm] = 135.8, 133.0, 129.4, 129.1, 128.8, 127.0, 72.7, 72.6, 70.3, 59.2, 56.2, 44.2, 42.0, 39.7, 39.1, 33.5, 33.4, 25.4, 24.4, 21.6, 20.6, 18.5, 15.4.

IR (ν/cm^{-1}, ATR) = 3448, 2929, 2865, 1446, 1385, 1363, 1102, 1047, 935, 753.

HRMS (ESI): *m/z* calculated for $C_{23}H_{35}ClO_2Na^+$ $[M+Na]^+$: 401.2218, found 401.2230.

Compound **S16**

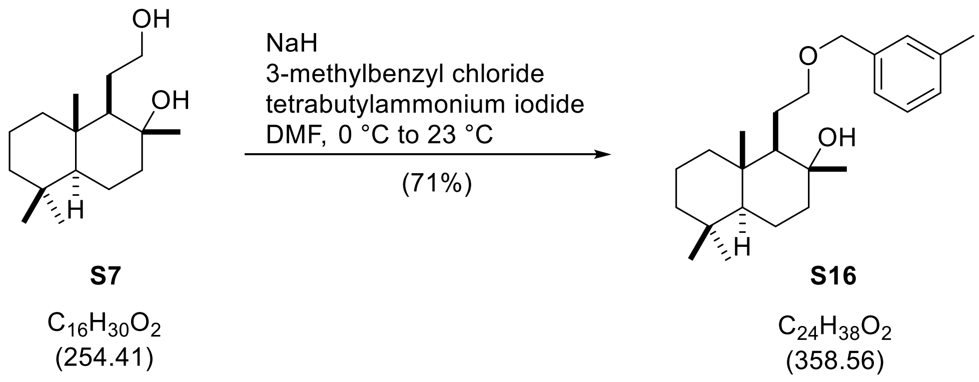

To a stirred solution of diol **S7** (301 mg, 1.18 mmol, 1.0 eq.) in DMF (4.0 mL) was added sodium hydride (60% in mineral oil, 70.9 mg, 1.77 mmol, 1.5 eq.) at 0 °C. After 30 min tetrabutylammonium iodide (43.7 mg, 118 µmol, 0.1 eq.) and 3-methylbenzyl chloride (268 µL, 286 mg, 1.77 mmol, 1.5 eq.) were added and the mixture was allowed to warm to 23 °C over 2 d. Water and EtOAc were added to the reaction mixture sequentially. The organic phase was separated and the aqueous phase was extracted with EtOAc. The combined organic extracts were washed repeatedly with brine, dried over $MgSO_4$, filtered and concentrated under reduced pressure. The residue was purified by flash column chromatography (SiO_2, *n*-pentane/EtOAc 1:0 to 5:1) to afford ether **S16** (317 mg, 837 µmol, 71%) as a colorless oil.

$\mathbf{R_{\mathit{f}}}$ = 0.61 (*n*-pentane/EtOAc, 5:1).

$[\alpha]_D^{27}$ = −17.7 (*c* = 1.36, $CHCl_3$).

^{1}H-NMR (700 MHz, $CDCl_3$): δ [ppm] = 7.23 (t, *J* = 7.6 Hz, 1H), 7.14 (s, 1H), 7.12 (d, *J* = 7.6 Hz, 1H), 7.09 (d, *J* = 7.6 Hz, 1H), 4.49 (s, 2H), 3.66 – 3.59 (m, 1H), 3.38 – 3.32 (m, 1H), 3.30 (s_{br}, 1H), 2.35 (s, 3H), 1.90 (dt, *J* = 12.6, 3.3 Hz, 1H), 1.80 – 1.73 (m, 1H), 1.67 – 1.62 (m, 1H), 1.62 – 1.54 (m, 3H), 1.45 – 1.35 (m, 3H), 1.26 (ddd, *J* = 13.7, 12.2, 3.1 Hz, 1H), 1.23 (s, 1H), 1.17 – 1.13 (m, 3H), 1.11 (dd, *J* = 13.2, 4.1 Hz, 1H), 0.90 (dd, *J* = 12.3, 2.3 Hz, 1H), 0.87 (s, 3H), 0.86 – 0.81 (m, 1H), 0.79 (s, 3H), 0.78 (s, 3H).

^{13}C-NMR (176 MHz, $CDCl_3$): δ [ppm] = 138.1, 138.0, 128.7, 128.6, 128.5, 125.1, 73.3, 72.4, 72.0, 59.3, 56.3, 44.2, 42.1, 39.6, 39.1, 33.6, 33.4, 25.4, 24.5, 21.6, 21.6, 20.6, 18.6, 15.4.

IR (ν/cm^{-1}, ATR) = 3444, 2925, 2862, 1610, 1460, 1384, 1246, 1156, 1081, 934.

HRMS (ESI): *m/z* calculated for $C_{24}H_{38}O_2Na^+$ $[M+Na]^+$: 381.2764, found 381.2780.

Compound **S17**

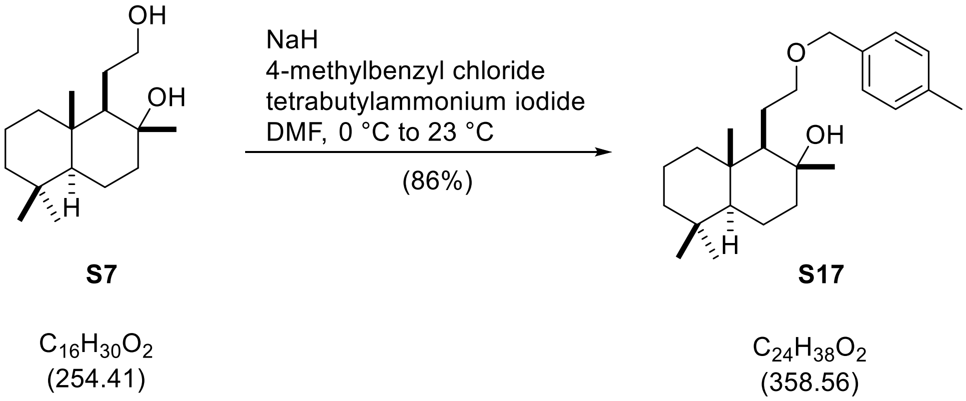

To a stirred solution of diol **S7** (301 mg, 1.18 mmol, 1.0 eq.) in DMF (4.0 mL) was added sodium hydride (60% in mineral oil, 70.9 mg, 1.77 mmol, 1.5 eq.) at 0 °C. After 30 min tetrabutylammonium iodide (43.7 mg, 118 µmol, 0.1 eq.) and 4-methylbenzyl chloride (269 µL, 286 mg, 1.77 mmol, 1.5 eq.) were added and the mixture was allowed to warm to 23 °C over 2 h. Water and EtOAc were added to the reaction mixture sequentially. The organic phase was separated and the aqueous phase was extracted with EtOAc. The combined organic extracts were washed repeatedly with brine, dried over $MgSO_4$, filtered and concentrated under reduced pressure. The residue was purified by flash column chromatography (SiO_2, *n*-pentane/EtOAc 1:0 to 5:1) to afford ether **S17** (387 mg, 1.02 mmol, 86%) as a colorless oil.

$\mathbf{R_f}$ = 0.50 (*n*-pentane/EtOAc, 5:1).

$[\alpha]_D^{27}$ = −20.4 (*c* = 1.43, $CHCl_3$).

^{1}H-NMR (700 MHz, $CDCl_3$): δ [ppm] = 7.21 (d, *J* = 7.7 Hz, 2H), 7.15 (d, *J* = 7.7 Hz, 2H), 4.48 (s, 2H), 3.65 – 3.59 (m, 1H), 3.52 – 3.15 (m, 2H), 2.33 (s, 3H), 1.90 (dt, *J* = 12.7, 3.3 Hz, 1H), 1.79 – 1.72 (m, 1H), 1.68 – 1.62 (m, 1H), 1.62 – 1.53 (m, 3H), 1.45 – 1.34 (m, 3H), 1.28 – 1.19 (m, 2H), 1.14 (s, 3H), 1.11 (dd, *J* = 13.4, 4.2 Hz, 1H), 0.90 (dd, *J* = 12.4, 2.3 Hz, 1H), 0.87 (s, 3H), 0.86 – 0.81 (m, 1H), 0.79 (s, 3H), 0.78 (s, 3H).

^{13}C-NMR (176 MHz, $CDCl_3$): δ [ppm] = 137.5, 135.0, 129.2 (2C), 128.0 (2C), 73.2, 72.4, 72.0, 59.4, 56.2, 44.2, 42.1, 39.6, 39.2, 33.6, 33.4, 25.4, 24.6, 21.6, 21.3, 20.6, 18.6, 15.4.

IR (ν/cm^{-1}, ATR) = 3443, 2925, 2861, 1515, 1459, 1384, 1241, 1089, 934, 802.

HRMS (ESI): *m/z* calculated for $C_{24}H_{38}O_2Na^+$ $[M+Na]^+$: 381.2764, found 381.2767.

Compound **S18**

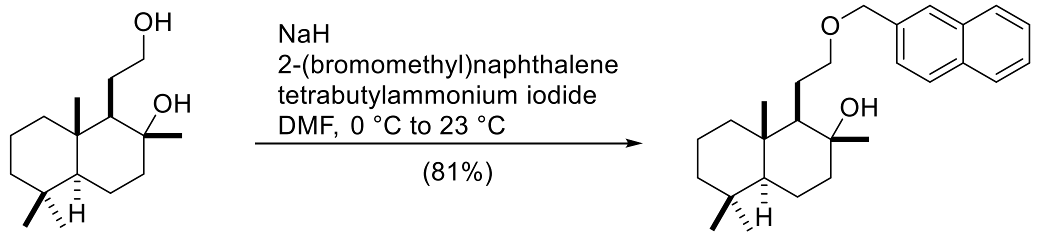

S7 $C_{16}H_{30}O_2$ (254.41) **S18** $C_{27}H_{38}O_2$ (394.59)

To a stirred solution of diol **S7** (300 mg, 1.18 mmol, 1.0 eq.) in DMF (4.0 mL) was added sodium hydride (60% in mineral oil, 70.9 mg, 1.77 mmol, 1.5 eq.) at 0 °C. After 30 min tetrabutylammonium iodide (21.8 mg, 59.0 μmol, 0.05 eq.) and 2-(bromomethyl)napthalene (391 mg, 1.77 mmol, 1.5 eq.) in DMF (0.5 mL) were added and the mixture was allowed to warm to 23 °C over 16 h. Water and EtOAc were added to the reaction mixture sequentially. The organic phase was separated and the aqueous phase was extracted with EtOAc. The combined organic extracts were washed repeatedly with brine, dried over $MgSO_4$, filtered and concentrated under reduced pressure. The residue was purified by flash column chromatography (SiO_2, *n*-pentane/EtOAc 1:0 to 5:1) to afford ether **S18** (375 mg, 950 μmol, 81%) as a colorless oil.

$\mathbf{R_f}$ = 0.41 (*n*-pentane/EtOAc, 5:1).

$[\alpha]_D^{27}$ = −15.4 (*c* = 1.36, $CHCl_3$).

^{1}H-NMR (700 MHz, $CDCl_3$): δ [ppm] = 7.85 – 7.81 (m, 3H), 7.77 (s, 1H), 7.51 – 7.43 (m, 3H), 4.74 – 4.66 (m, 2H), 3.69 – 3.64 (m, 1H), 3.44 – 3.38 (m, 1H), 3.30 (s_{br}, 1H), 1.91 (dt, *J* = 12.6, 3.3 Hz, 1H), 1.84 – 1.76 (m, 1H), 1.67 – 1.52 (m, 4H), 1.42 (td, *J* = 13.2, 4.1 Hz, 1H), 1.39 – 1.31 (m, 2H), 1.28 – 1.23 (m, 1H), 1.21 (t, *J* = 4.2 Hz, 1H), 1.15 (s, 3H), 1.09 – 1.03 (m, 1H), 0.88 – 0.86 (m, 1H), 0.85 (s, 3H), 0.83 – 0.79 (m, 1H), 0.79 (s, 3H), 0.78 (s, 3H).

^{13}C-NMR (176 MHz, $CDCl_3$): δ [ppm] = 135.5, 133.4, 133.2, 128.4, 128.0, 127.8, 126.8, 126.2, 126.0 (2C), 73.4, 72.5, 72.1, 59.3, 56.2, 44.2, 42.0, 39.6, 39.1, 33.5, 33.4, 25.4, 24.5, 21.6, 20.6, 18.5, 15.4.

IR (ν/cm^{-1}, ATR) = 3444, 3054, 2927, 2862, 1602, 1508, 1461, 1162, 1092, 934.

HRMS (ESI): *m/z* calculated for $C_{27}H_{38}O_2Na^+$ $[M+Na]^+$: 417.2764, found 417.2776.

Compound **S19**

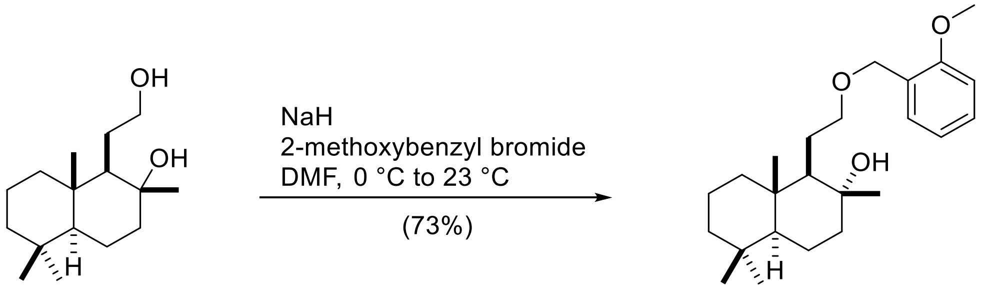

S7

$C_{16}H_{30}O_2$
(254.41)

S19

$C_{24}H_{38}O_3$
(374.56)

To a stirred solution of diol **S7** (105 mg, 413 μmol, 1.0 eq.) in DMF (2.0 mL) was added sodium hydride (60% in mineral oil, 26.1 mg, 652 μmol, 1.6 eq.) at 0 °C. After 30 min 2-methoxybenzyl bromide (151 mg, 731 μmol, 1.8 eq.) in DMF (1.0 mL) was added and the mixture was allowed to warm to 23 °C over 20 h. Water and EtOAc were added to the reaction mixture sequentially. The organic phase was separated and the aqueous phase was extracted with EtOAc. The combined organic extracts were washed repeatedly with brine, dried over $MgSO_4$, filtered and concentrated under reduced pressure. The residue was purified by flash column chromatography (SiO_2, *n*-pentane/EtOAc 30:1 to 1:1) to afford ether **S19** (112 mg, 300 μmol, 73%) as a colorless oil.

$\mathbf{R}_f$ = 0.24 (*n*-pentane/EtOAc, 10:1).

$[\alpha]_D^{24}$ = −8.14 (*c* = 0.55, $CHCl_3$).

^{1}H-NMR (500 MHz, $CDCl_3$): δ [ppm] = 7.33 (dd, *J* = 7.4, 1.7 Hz, 1H), 7.28 – 7.22 (m, 1H), 6.95 (td, *J* = 7.5, 1.1 Hz, 1H), 6.86 (d, *J* = 8.2 Hz, 1H), 4.56 (q, *J* = 12.4 Hz, 2H), 3.83 (s, 3H), 3.72 – 3.63 (m, 1H), 3.43 – 3.34 (m, 1H), 3.09 (s_{br}, 1H), 1.90 (dt, *J* = 12.5, 3.2 Hz, 1H), 1.82 – 1.70 (m, 1H), 1.66 – 1.52 (m, 4H), 1.44 – 1.32 (m, 3H), 1.29 – 1.22 (m, 1H), 1.20 (t, *J* = 4.3 Hz, 1H), 1.13 (s, 3H), 1.12 – 1.07 (m, 1H), 0.90 – 0.81 (m, 5H), 0.78 (s, 6H).

^{13}C-NMR (126 MHz, $CDCl_3$): δ [ppm] = 157.2, 129.2, 128.8, 126.5, 120.6, 110.3, 72.3, 72.2, 68.2, 59.3, 56.2, 55.4, 44.2, 42.1, 39.6, 39.1, 33.6, 33.4, 25.4, 24.4, 21.6, 20.6, 18.6, 15.4.

IR (ν/cm^{-1}, ATR) = 3378, 2918, 2851, 1599, 1463, 1374, 1241, 1086, 970, 754.

HRMS (ESI): *m/z* calculated for $C_{24}H_{38}O_3Na^+$ $[M+Na]^+$: 397.2713, found 397.2713.

Compound **S20**

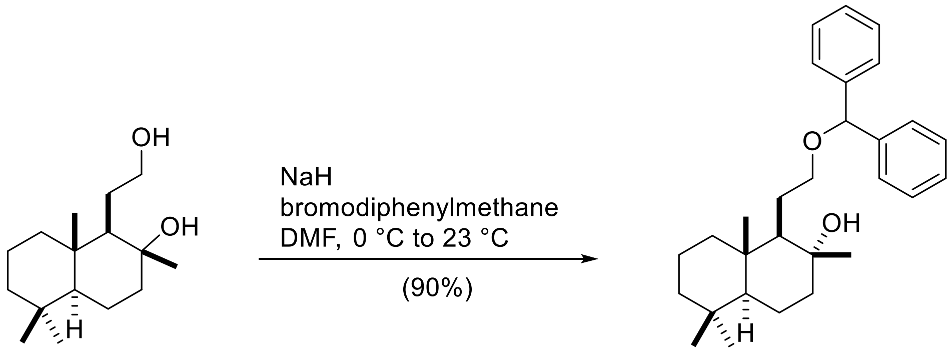

S7

$C_{16}H_{30}O_2$
(254.41)

S20

$C_{29}H_{40}O_2$
(420.63)

To a stirred solution of diol **S7** (515 mg, 2.02 mmol, 1.0 eq.) in DMF (10 mL) was added sodium hydride (60% in mineral oil, 123 mg, 3.03 mmol, 1.5 eq.) at 0 °C. After 30 min bromodiphenylmethane (1.03 g, 4.05 mmol, 2.0 eq.) was added and the mixture was allowed to warm to 23 °C over 20 h. Water and EtOAc were added to the reaction mixture sequentially. The organic phase was separated and the aqueous phase was extracted with EtOAc. The combined organic extracts were washed repeatedly with brine, dried over $MgSO_4$, filtered and concentrated under reduced pressure. The residue was purified by flash column chromatography (SiO_2, *n*-pentane/EtOAc 30:1 to 1:1) to afford ether **S20** (765 mg, 1.82 mmol, 90%) as a colorless oil.

$\mathbf{R}_f$ = 0.47 (*n*-pentane/EtOAc, 10:1).

$[\alpha]_D^{24}$ = −12.8 (*c* = 3.15, $CHCl_3$).

^{1}**H-NMR** (500 MHz, $CDCl_3$): δ [ppm] = 7.38 – 7.29 (m, 8H), 7.27 – 7.21 (m, 2H), 5.37 (s, 1H), 3.63 (dt, *J* = 9.1, 4.6 Hz, 1H), 3.42 – 3.32 (m, 1H), 3.03 (s_{br}, 1H), 1.91 (dt, *J* = 12.5, 3.2 Hz, 1H), 1.89 – 1.79 (m, 1H), 1.68 – 1.49 (m, 4H), 1.44 – 1.32 (m, 3H), 1.28 – 1.20 (m, 2H), 1.16 (s, 3H), 1.09 (td, *J* = 13.4, 12.7, 4.2 Hz, 1H), 0.91 – 0.81 (m, 4H), 0.78 (s, 6H), 0.75 – 0.70 (m, 1H).

^{13}**C-NMR** (126 MHz, $CDCl_3$): δ [ppm] = 142.1, 142.1, 128.6 (2C), 128.6 (2C), 127.7, 127.6, 127.2 (2C), 126.9 (2C), 84.5, 72.6, 70.9, 59.0, 56.2, 44.2, 42.1, 39.5, 39.1, 33.5, 33.4, 25.4, 24.4, 21.6, 20.6, 18.5, 15.4.

IR (ν/cm^{-1}, ATR) = 3401, 2927, 2865, 1600, 1490, 1450, 1182, 1070, 1024, 931.

HRMS (ESI): *m/z* calculated for $C_{29}H_{40}O_2Na^+$ $[M+Na]^+$: 443.2920, found 443.2910.

Compound **S21**

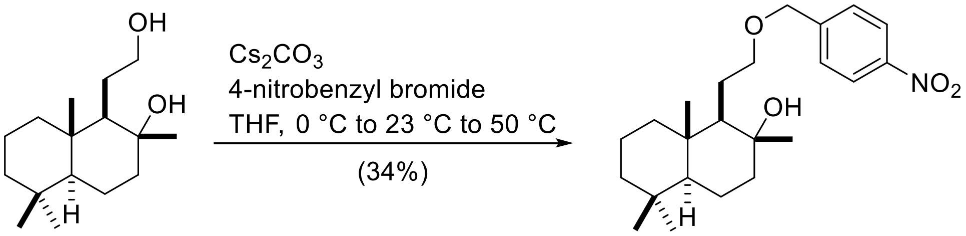

S7

$C_{16}H_{30}O_2$
(254.41)

S21

$C_{23}H_{35}NO_4$
(389.53)

To a stirred solution of caesium carbonate (273 mg, 838 μmol, 2.0 eq.) in THF (2.0 mL) was added diol **S7** (107 mg, 419 μmol, 1.0 eq.) at 0 °C. After 10 min 4-nitrobenzyl bromide (181 mg, 838 μmol, 2.0 eq.) in THF (1.0 mL) was added and the mixture was allowed to warm to 23 °C over 3 d then heated to 50 °C for 3 h. Water and EtOAc were added to the reaction mixture sequentially. The organic phase was separated and the aqueous phase was extracted with EtOAc. The combined organic extracts were washed with brine, dried over $MgSO_4$, filtered and concentrated under reduced pressure. The residue was purified by flash column chromatography (SiO_2, *n*-pentane/EtOAc 9:1 to 1:1) to afford ether **S21** (54.7 mg, 140 μmol, 34%, 42% brsm) as a yellow oil and diol **S7** (21.1 mg, 82.9 μmol) as a white solid.

$\mathbf{R}_f$ = 0.75 (*n*-pentane/EtOAc, 4:1).
$[\alpha]_D^{23}$ = −14.9 (*c* = 1.10, $CHCl_3$).
^{1}H-NMR (500 MHz, $CDCl_3$): δ [ppm] = 8.20 (d, *J* = 8.5 Hz, 2H), 7.49 (d, *J* = 8.5 Hz, 2H), 4.62 (s, 2H), 3.70 – 3.63 (m, 1H), 3.50 – 3.44 (m, 1H), 2.75 (s_{br}, 1H), 1.91 (dt, *J* = 12.5, 3.3 Hz, 1H), 1.85 – 1.78 (m, 1H), 1.68 – 1.56 (m, 4H), 1.45 – 1.36 (m, 3H), 1.31 – 1.23 (m, 1H), 1.21 (t, *J* = 4.3 Hz, 1H), 1.16 (s, 3H), 1.15 – 1.10 (m, 1H), 0.92 (dd, *J* = 12.4, 2.3 Hz, 1H), 0.91 – 0.88 (m, 1H), 0.87 (s, 3H), 0.80 (s, 3H), 0.79 (s, 3H).
^{13}C-NMR (126 MHz, $CDCl_3$): δ [ppm] = 147.6, 145.8, 127.9 (2C), 123.9 (2C), 73.2, 72.8, 72.1, 59.3, 56.3, 44.3, 42.1, 39.8, 39.2, 33.6, 33.4, 25.5, 24.5, 21.6, 20.6, 18.6, 15.4.
IR (ν/cm^{-1}, ATR) = 3458, 2925, 2862, 1603, 1519, 1460, 1385, 1342, 1098, 1015.
HRMS (ESI): *m/z* calculated for $C_{23}H_{35}NO_4Na^+$ $[M+Na]^+$: 412.2458, found 412.2473.
Note: This procedure is unoptimized.

Compound **13** from **S8**[18]

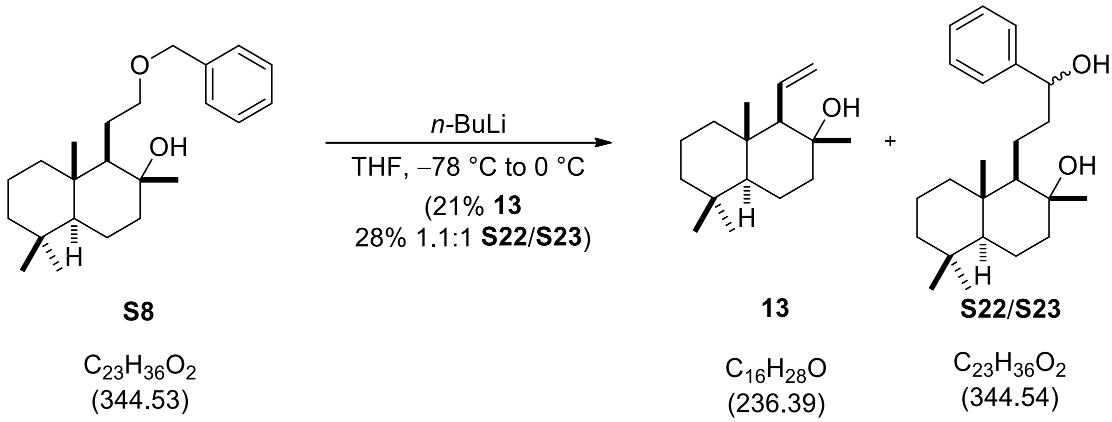

To a solution of ether **S8** (100 mg, 290 µmol, 1.0 eq.) in THF (4 mL) at −78 °C was added a solution of *n*-BuLi (396 µL, 2.5 M in hexane, 871 µmol, 3.0 eq.). After stirring for 30 min at that temperature the reaction mixture was warmed to 0 °C and stirred for 12 h while reaching 23 °C. Water was added and the reaction mixture was diluted with EtOAc. The organic phase was separated and the aqueous phase was extracted with EtOAc. The combined organic extracts were washed with brine, dried over $MgSO_4$, filtered and concentrated under reduced pressure. The residue was purified by flash column chromatography (SiO_2, *n*-pentane/EtOAc 10:1 to 1:1) to afford alkene **13** (14.2 mg, 60.1 µmol, 21%) as a white solid and **S22** (15.2 mg, 44.1 µmol, 15%) as a light-yellow oil and **S23** (13.4 mg, 38.9 µmol, 13%) as a light-yellow oil.

S22

R_f = 0.45 (*n*-pentane/EtOAc, 3:1).

$[\alpha]_D^{23}$ = −9.10 (*c* = 1.12, $CHCl_3$).

^{1}H-NMR (500 MHz, $CDCl_3$): δ [ppm] = 7.37 (d, *J* = 7.2 Hz, 2H), 7.33 (t, *J* = 7.5 Hz, 2H), 7.24 (t, *J* = 7.3 Hz, 1H), 4.80 (dd, *J* = 10.2, 3.4 Hz, 1H), 3.56 – 2.51 (m, 1H), 1.86 – 1.76 (m, 2H), 1.73 – 1.67 (m, 1H), 1.67 – 1.55 (m, 4H), 1.51 – 1.34 (m, 4H), 1.31 – 1.23 (m, 3H), 1.20 – 1.08 (m, 4H), 0.97 – 0.89 (m, 2H), 0.88 (s, 3H), 0.80 (s, 3H), 0.79 (s, 3H).

^{13}C-NMR (126 MHz, $CDCl_3$): δ [ppm] = 145.3, 128.4 (2C), 127.2, 125.9 (2C), 74.6, 71.8, 58.6, 56.2, 43.9, 42.1, 41.3, 34.0, 39.3, 33.5, 33.3, 24.8, 21.7, 20.6, 20.4, 18.5, 15.4.

IR (ν/cm^{-1}, ATR) = 3371, 2991, 2922, 2876, 1456, 1364, 1203, 1157, 1068, 759.

HRMS (ESI): *m/z* calculated for $C_{23}H_{36}O_2K^+$ $[M+K]^+$: 383.2347, found 383.2362.

S23

R_f = 0.36 (*n*-pentane/EtOAc, 3:1).

$[\alpha]_D^{23}$ = +20.0 (*c* = 0.76, $CHCl_3$).

^{1}H-NMR (700 MHz, $CDCl_3$): δ [ppm] = 7.36 – 7.34 (m, 2H), 7.34 – 7.31 (m, 2H), 7.26 – 7.23 (m, 1H), 4.70 (dd, *J* = 9.2, 4.0 Hz, 1H), 1.89 – 1.77 (m, 3H), 1.70 – 1.62 (m, 3H), 1.59 – 1.53 (m, 1H), 1.53 – 1.49 (m, 1H), 1.49 – 1.43 (m, 1H), 1.43 – 1.32 (m, 4H), 1.29 – 1.22 (m, 2H), 1.17 (s, 3H), 1.15 – 1.11 (m, 1H), 0.98 – 0.93 (m, 2H), 0.86 (s, 3H), 0.78 (s, 3H), 0.76 (s, 3H).

^{13}C-NMR (176 MHz, $CDCl_3$): δ [ppm] = 145.3, 128.5 (2C), 127.4, 125.9 (2C), 76.5, 74.9, 61.6, 56.1, 44.4, 42.5, 42.1, 39.8, 39.3, 33.5, 33.4, 24.5, 22.5, 21.6, 20.7, 18.5, 15.3.

IR (ν/cm^{-1}, ATR) = 3352, 2922, 2867, 1737, 1457, 1387, 1126, 1082, 970, 762.

HRMS (ESI): *m/z* calculated for $C_{23}H_{36}O_2K^+$ $[M+K]^+$: 383.2347, found 383.2363.

Note: No attempt was made to determine the relative configuration of the epimeric alcohols **S22/S23**

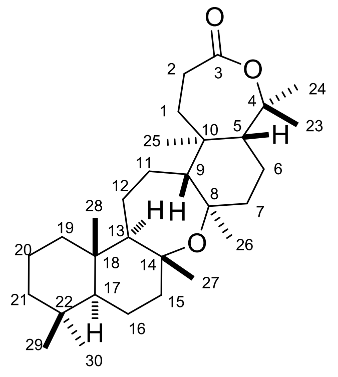

cupacinoxepin (**4**)

Table 1.: Comparison of the ^{1}H-NMR spectra of cupacinoxepin

No.	Isolated cupacinoxepin[7]<br>600 MHz, $CDCl_3$<br>δ_H mult (*J*, Hz)	Synthetic cupacinoxepin<br>500 MHz, $CDCl_3$<br>δ_H mult (*J*, Hz)
1	1.66 m	1.67 m
	1.89[a]	1.88[a]
2	2.66 (td, J = 4.2, 14.4)	2.66 (td, *J* = 14.0, 4.8)
	2.48 (dt, 3.6, 14.4)	2.48 (ddd, *J* = 14.0, 5.2, 3.5)
3		
4		
5	1.87[a]	1.86[a]
6	1.48-1.56[a]	1.47-1.56[a]
7	1.74[a]	1.73[a]
	1.55[a]	1.54[a]
8		
9	1.51[a]	1.50[a]
10		
11	1.73[a]	1.72[a]
	1.35[a]	1.37[a]
12	1.84[a]	1.83[a]
	1.22[a]	1.23[a]
13	1.41[a]	1.41[a]
14		
15	1.76[a]	1.76[a]
	1.55[a]	1.54[a]
16	1.22[a]	1.21[a]
	1.60[a]	1.60[a]
17	0.87[a]	0.87[a]
18		
19	1.77[a]	1.78[a]
	0.89[a]	0.88[a]
20	1.58[a]	1.60[a]
	1.44[a]	1.43[a]
21	1.36[a]	1.35[a]

	1.13 dt (3.6, 13.2)	1.13 dt (13.5, 4.3)
22		
23	1.48 s	1.48 s
24	1.36 s	1.36 s
25	1.00 s	1.00 s
26	1.29 s	1.29 s
27	1.25 s	1.25 s
28	0.74 s	0.74 s
29	0.77 s	0.77 s
30	0.85 s	0.85 s

[a] Signal overlapping

Table 2.: Comparison of the ^{13}C-NMR spectra of cupacinoxepin

No.	^{13}C-NMR isolated cupacinoxepin[7] 150 MHz, $CDCl_3$	^{13}C-NMR synthetic cupacinoxepin 176 MHz, $CDCl_3$	Deviation
1	40.2	40.3	−0.1
2	32.4	32.6	−0.2
3	175.0	175.0	0.0
4	85.8	85.9	−0.1
5	52.1	52.2	−0.1
6	25.9	26.0	−0.1
7	43.7	43.7	0.0
8	78.8	78.9	−0.1
9	61.2	61.4	−0.2
10	41.0	41.2	−0.2
11	26.4	26.5	−0.1
12	25.1	25.2	−0.1
13	60.8	61.0	−0.2
14	80.4	80.5	−0.1
15	45.3	45.3	0.0
16	20.8	20.9	−0.1
17	56.2	56.3	−0.1
18	39.3	39.0	+0.3
19	40.5	40.5	0.0
20	18.9	19.0	−0.1
21	42.0	42.1	−0.1
22	33.5	33.6	−0.1
23	30.7	30.8	−0.1
24	27.3	27.4	−0.1
25	18.3	18.4	−0.1
26	24.1	24.2	−0.1
27	25.4	25.5	−0.1
28	15.9	16.0	−0.1

29	21.6	21.6	0.0
30	33.5	33.6	−0.1

Isolated Cupacinoxepin (^{1}H, 600 MHz, $CDCl_3$)[7]

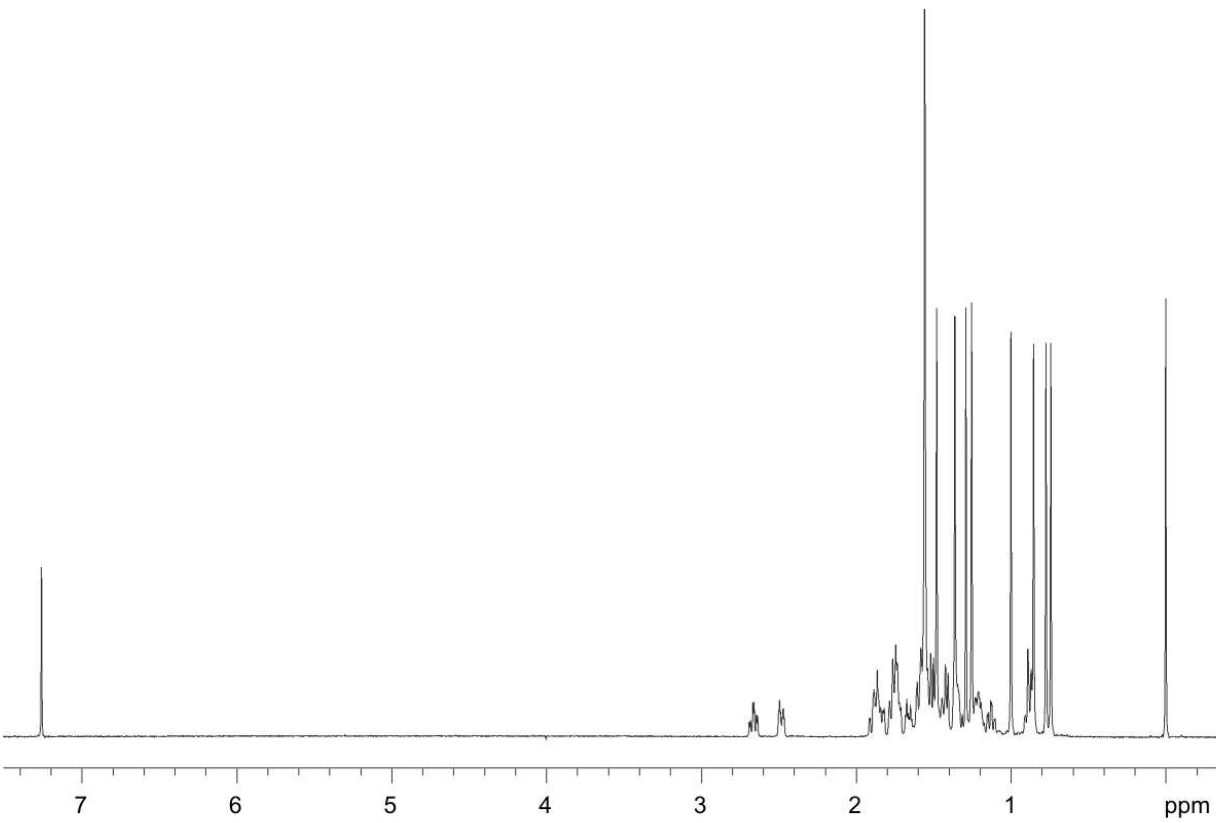

Synthetic Cupacinoxepin (^{1}H, 500 MHz, $CDCl_3$)

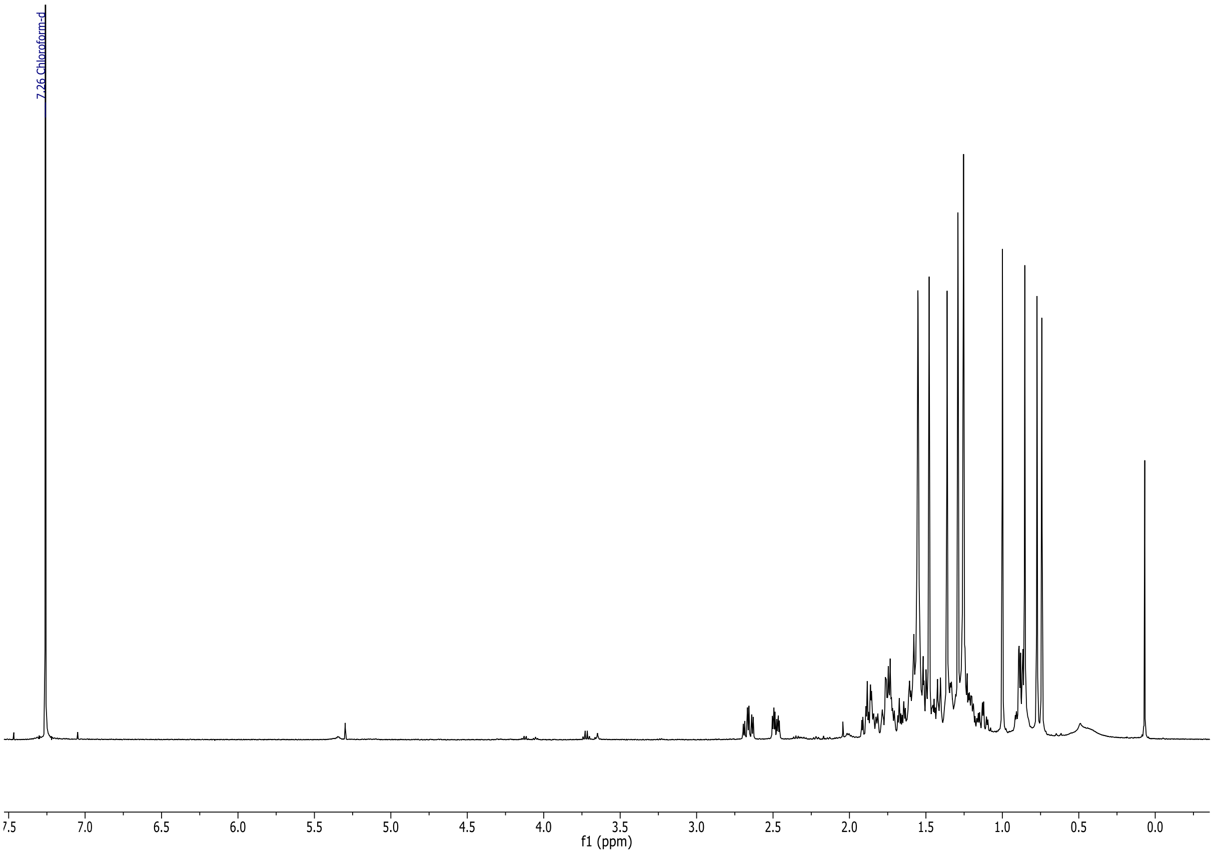

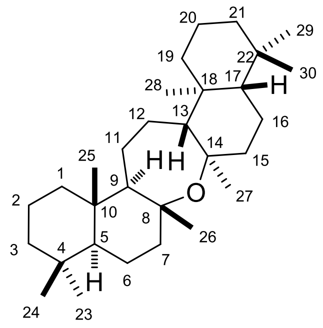

onoceranoxide (**7**)

Table 3.: Comparison of the ^{1}H-NMR spectra of onoceranoxide **7**

no.	Isolated onoceranoxide[8]<br>100 MHz, $CDCl_3$<br>δ_H mult (*J*, Hz)	Synthetic onoceranoxide<br>700 MHz, $CDCl_3$<br>δ_H mult (*J*, Hz)
1, 19	/	1.76 (m), 0.87 (m)
2, 20	/	1.58 (m), 1.40 (m)
3, 21	/	1.34 (m), 1.11 (m)
4, 22	/	/
5, 17	/	0.88 (m)
6, 16	/	1.58 (m), 1.21 (m)
7, 15	/	1.75 (m), 1.56 (m)
8, 14	/	/
9, 13	/	1.38 (m)
10, 18	/	/
11, 12	/	1.77 (m), 1.21 (m)
23, 30	0.845	0.85 (s)
24, 29	0.735	0.74 (s)
25, 28	0.767	0.77 (s)
26, 27	1.259	1.26 (s)

Table 4.: Comparison of the ^{13}C-NMR spectra of onoceranoxide **7**

no.	^{13}C-NMR isolated onoceranoxide[8]<br>25 MHz, $CDCl_3$	^{13}C-NMR synthetic onoceranoxide<br>176 MHz, $CDCl_3$	Deviation
1, 19	40.5	40.5	0
2, 20	19.0	19.1	−0.1
3, 21	42.2	42.2	0
4, 22	33.5	33.6	−0.1
5, 17	56.3	56.3	0
6, 16	20.8	20.9	−0.1
7, 15	45.4	45.4	0
8, 14	79.9	80.1	−0.2
9, 13	60.9	60.9	0

10, 18	38.9	39.0	−0.1
11, 12	25.0	25.1	−0.1
23, 30	33.5	33.6	−0.1
24, 29	21.5	21.7	−0.2
25, 28	15.8	15.9	−0.1
26, 27	25.4	25.4	0

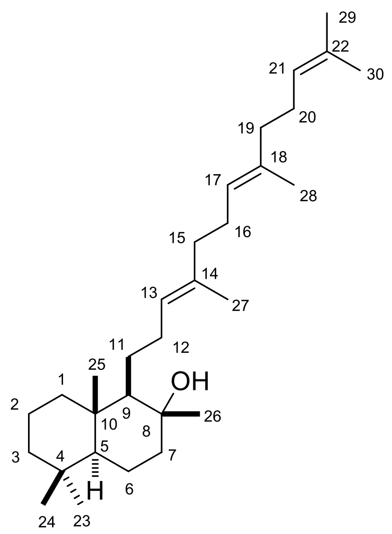

8α-hydroxypolypoda-13,17,21 (**9**)

Table 5.: Comparison of the ^{1}H-NMR spectra of 8α-hydroxypolypoda-13,17,21

no.	Isolated **9**[10] (270 MHz, $CDCl_3$) δ_H mult (*J*, Hz)	Synthetic **9** (500 MHz, $CDCl_3$) δ_H mult (*J*, Hz)
		1.65[a]
1	/	0.98 td (13.0, 3.7)
		1.64[a]
2	/	1.40[a]
		1.39[a]
3	/	1.14[a]
4	/	/
5	/	0.91 dd (12.1, 2.3)
		1.66[a]
6	/	1.26[a]
		1.86 dt (12.1, 3.1)
7	/	1.36[a]
8	/	/
9	/	0.91 dd (12.1, 2.3)
10	/	/
		1.44[a]
11	/	1.26[a]
		2.07[a]
12	/	2.07[a]
13	5.07 – 5.21 bt (6.2)	5.21 – 5.16 m
14	/	/
		1.99[a]
15	/	1.99[a]
		1.96 – 2.11 m[a]
16	/	1.96 – 2.11 m[a]
17	5.07 – 5.21 bt (6.2)	5.14 – 5.06 m[a]
18	/	/
19	/	1.99 (m)
20	/	1.96 – 2.11 m[a]

		1.96 – 2.11 m[a]
21	5.07 – 5.21 bt (6.2)	5.14 – 5.06 m[a]
22	/	/
23	0.869	0.87 s
24	0.789	0.79 s
25	0.789	0.79 s
26	1.130	1.13 s
27	1.616	1.60 s[a] or 1.61 s
28	1.602	1.60 s[a] or 1.61 s
29	1.602	1.68 s
30	1.681	1.60 s[a]

[a] Signal overlapping

Table 6.: Comparison of the ^{13}C-NMR spectra of 8α-hydroxypolypoda-13,17,21

no.	^{13}C Natural **9**[10] (68 MHz, $CDCl_3$)	^{13}C Synthetic **9** (176 MHz, $CDCl_3$)	Deviation
1	39.7	39.9	−0.2
2	18.5	18.6	−0.1
3	42.5	42.2	−0.3
4	33.3	33.4	−0.1
5	56.3	56.3	0
6	20.6	20.7	−0.1
7	44.6	44.7	−0.1
8	74.1	74.2	−0.1
9	61.6	61.7	−0.1
10	39.2	39.3	−0.1
11	25.6	25.7	−0.1
12	31.5	31.6	−0.1
13	125.1	125.2	−0.1
14	135.0	135.1	−0.1
15	39.8	39.9	−0.1
16	26.7	26.8 or 26.9	
17	124.3	124.4	−0.1
18	135.2	135.3	−0.1
19	39.8	39.9	−0.1
20	26.8	26.8 or 26.9	
21	124.5	124.6	−0.1
22	131.3	131.4	−0.1
23	33.4	33.6	−0.2
24	21.6	21.7	−0.1
25	15.4	15.6	−0.2
26	23.9	23.9	0
27	16.0	16.2 or 16.4	

28	16.1	16.2 or 16.4	
29	25.7	25.9	−0.2
30	17.7	17.8	−0.1

3. Screening of Reaction Conditions

Scheme 1.: Selected screening conditions for the formation of alkene **13**.

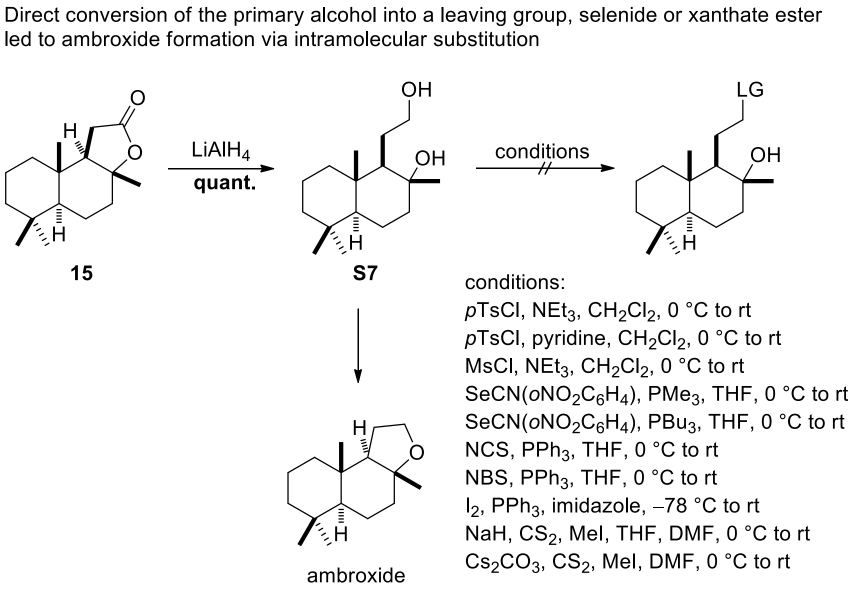

Table 7.: Selected screening conditions for the Wittig-type fragmentation.

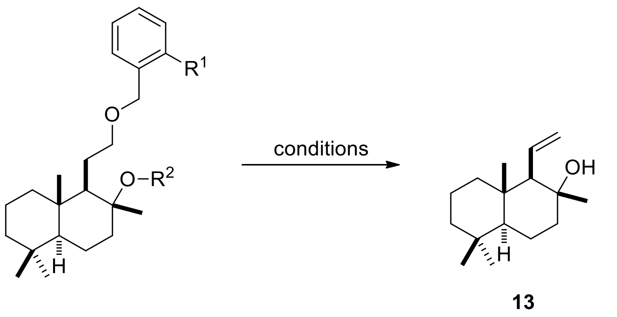

Entry	R^1	R^2	Conditions	Yield (%)/Observation
1	H	H	*n*-BuLi (3 eq.), THF, −78 °C, 30 min to 0 °C[18]	20
2	H	H	*n*-BuLi (4 eq.), Et_2O, −78 °C, 30 min to 0 °C	<5
3	H	H	*n*-BuLi (4 eq.), 1,2-dimethoxyethane, −78 °C, 30 min to 0 °C	16
4	H	H	LDA (4 eq.), THF, −78 °C, 30 min to 0 °C	<5
5	H	H	*s*-BuLi (4 eq.), THF, −78 °C, 30 min to 0 °C	28
6	H	H	*s*-BuLi (5 eq.), THF, −78 °C, 30 min to 0 °C	26
7	H	H	*t*-BuLi (4 eq.), THF, −78 °C, 30 min to 0 °C	20
8	H	H	*n*-BuLi (4 eq.), THF, TMEDA (4 eq.), −78 °C, 30 min to 0 °C	17
9	H	$SiMe_3$	*n*-BuLi (3 eq.), THF, −78 °C, 30 min to 0 °C	24[a]
10	Me	H	*n*-BuLi (4 eq.), THF, −78 °C, 30 min to −40 °C	No product formation
11	Me	H	*n*-BuLi (4 eq.), THF, −78 °C, 30 min to −28 °C	42
12	Me	H	*n*-BuLi (4 eq.), THF, −78 °C, 10 min to −13 °C (0.46 mmol scale)	44

13	Me	H	*n*-BuLi (4 eq.), THF, −78 °C, 30 min to −13 °C (13.2 mmol scale)	41
14	Me	H	*n*-BuLi (4 eq.), THF, −78 °C, 30 min to 0 °C	37
15	Me	H	*n*-BuLi (4 eq.), THF, −78 °C, 30 min to 50 °C	34
16	Me	H	*s*-BuLi (4 eq.), THF, −78 °C, 30 min to 0 °C	35[a]
17	Me	H	*n*-BuLi (8 eq.), THF, −78 °C, 30 min to 0 °C	29

[a] determined by H-NMR using an internal standard

Table 8.: Screening of ether derivatives for Wittig-type fragmentation

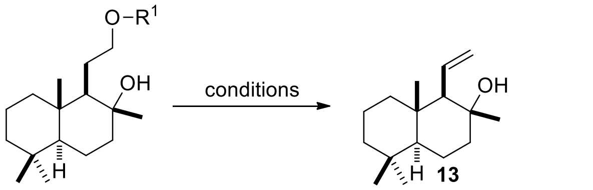

13

conditions A = *n*-BuLi (4 eq.), THF, –78 °C 30 min to 0 °C
conditions B = *n*-BuLi (4 eq.), THF, –78 °C 10 min to –13 °C
conditions C = *n*-BuLi (4 eq.), THF, –78 °C to rt, overnight

R^1 =

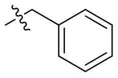	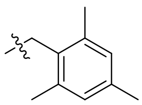	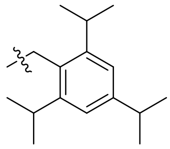	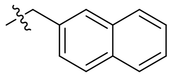
conditions A: 20%[a]<br>conditions C: 20%	conditions A: <5%<br>trace product formation<br>conditions C: <5%<br>trace product formation	conditions C: 0%	conditions B: 12%
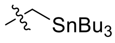	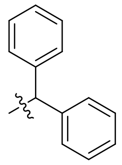	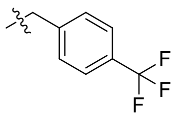	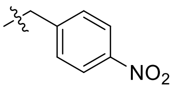
conditions C: 0%	conditions C: 0%	conditions A: 0%[a]<br>conditions B: 0%	conditions A: 0%[a]
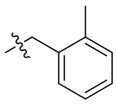	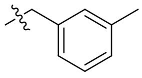	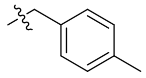	
conditions A: 37%<br>conditions B: 44%<br>conditions C: 42%	conditions B: 24%	conditions B: 22%	
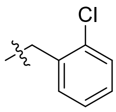	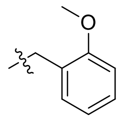	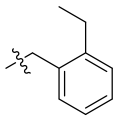	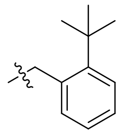
conditions A: 7%	conditions C: 0%	conditions B: 41%	conditions B: <5%<br>trace product formation

[a] 3.0 eq. *n*-BuLi used

Ether derivatives with alkyl substituents and benzylic hydrogen atoms in the 2-position of the aromatic ring gave higher yields of alkene **13** than ethers with electron withdrawing-, electron donating-, or sterically hindered substituents.

Table 9.: Tricyclization attempts of substrate **9** to give pentacyclic derivatives

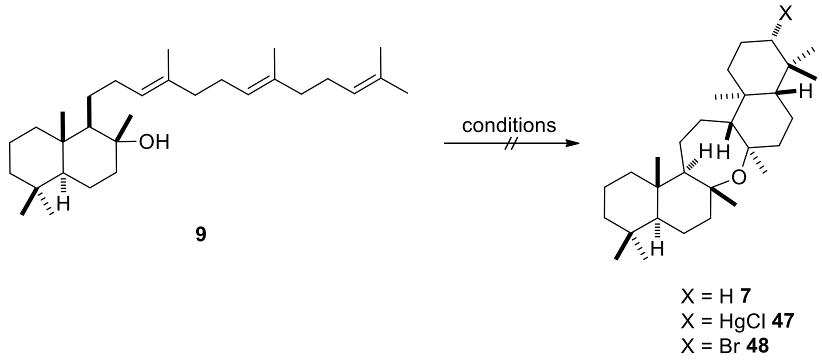

Entry	Conditions	Concentraion [mmol/mL]	Observation
1	Hg(OTFA)$_2$ (1.2 eq.), $MeNO_2$, −20 °C, Work up: NaCl (aq.)	0.004	Complex mixture
2	Hg(OTf)$_2$ (1.4 eq.), $MeNO_2$, Me_2NPh (1.5 eq.), −20 °C, Work up: NaCl (aq.)	0.006	Complex mixture
3	Hg(OTf)$_2$ (1.2 eq.), $MeNO_2$, tetramethylurea (1.3 eq.), −20 °C, Work up: NaCl (aq.)	0.003	Complex mixture
4	Hg(OTf)$_2$ (1.2 eq.), CH_2Cl_2, tetramethylurea (2.4 eq.), −20 °C, Work up: NaCl (aq.)	0.007	Complex mixture
5	Hg(OTf)$_2$ (1.2 eq.), MeCN, tetramethylurea (2.4 eq.), −20 °C, Work up: NaCl (aq.)	0.005	Complex mixture
6	$SnCl_4$ (2.0 eq.), *rac*-BINOL (2.2 eq.), CH_2Cl_2, −78 °C	0.01	Complex mixture
7	HFIP, eosin Y, rt, green LED	0.1	Complex mixture
8	BDSB, $MeNO_2$/CH_2Cl_2 (3:1), −20 °C	0.003	Complex mixture

Table 10.: Screening conditions **12** to **10**:

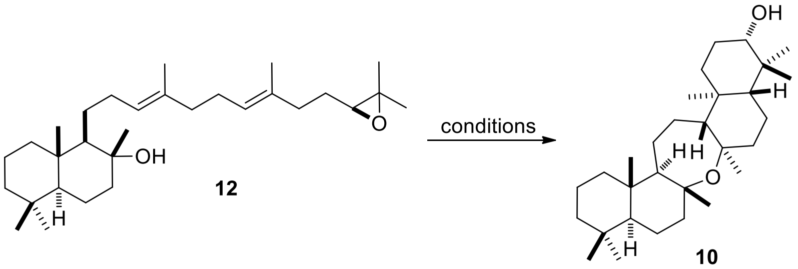

Entry	Conditions	Concentration (mmol/mL)	Observation
1	$SnCl_4$ (1.0 eq.), CH_2Cl_2, −78 °C	0.005	Complex mixture
2	$SnCl_4$ (2.0 eq.), *rac*-BINOL (2.2 eq.), CH_2Cl_2, −78 °C	0.002	Complex mixture
3	$SnCl_4$ (2.0 eq.), *rac*-BINOL (2.2 eq.), CH_2Cl_2, −90 °C	0.001	Incomplete conversion
4	Picric acid (1.8 eq.), $MeNO_2$, no stirring, 1 °C	0.01	Complex mixture Incomplete conversion
5	CSA (0.1 eq.), $MeNO_2$, 0 °C	0.005	Complex mixture
6	HFIP, 1 °C	0,003	Incomplete conversion
7	HFIP, TFA (0.05 eq.), CH_2Cl_2, 1 °C	0.003	Complex mixture Incomplete conversion
8	HFIP, Ph_4PBF_4 (10 eq.), 1 °C	0,001	Complex mixture
9	HFIP, $NaSbF_6$ (10 eq.), 1 °C	0.003	Complex mixture
10	HFIP, $AgSbF_6$ (10 eq.), 1 °C	0.003	Complex mixture
11	HFIP, Bu_4NPF_6 (10 eq.), 1 °C	0.003	Complex mixture
12	BF_3•OEt_2 (2.3 eq.), CH_2Cl_2, −78 °C	0.005	Complex mixture
13	Cp_2TiCl_2 (2.2 eq.), Mn (100 eq.), THF, rt	0.005	Complex mixture
14	Cp_2TiCl_2 (0.55 eq.), Mn (24 eq.), TMSCl (8 eq.), 2,4,6-collidine (14 eq.), THF, rt	0.005	Complex mixture
14	TIPSOTf (5.0 eq.), 2,6-lutidine, CH_2Cl_2, −78 °C to rt	0.0015	Complex mixture Incomplete conversion
15	$InCl_3$ (1.0 eq.), CH_2Cl_2, −78 °C to rt	0.005	Complex mixture
16	Me_2AlCl (3.0 eq.), CH_2Cl_2, −78 °C[a]	0.001	Incomplete conversion
17	Et_2AlCl (3.0 eq.), CH_2Cl_2, −78 °C[a]	0.001	Incomplete conversion
18	$MeAlCl_2$ (3.0 eq.), CH_2Cl_2, Et_2O, −78 °C[a]	0.001	Complex mixture
19	$EtAlCl_2$ (2.0 eq.), CH_2Cl_2, −78 °C[a,b]	0.001	14% **10**, 8% **21**

20	$EtAlCl_2$ (3.0 eq.), CH_2Cl_2, −78 °C[a]	0.001	20% **10**, 12% **21**
21	$EtAlCl_2$ (3.0 eq.), CH_2Cl_2, −78 °C[a]	0.0005	17% **10**, 16% **21**
22	$EtAlCl_2$ (3.0 eq.), CH_2Cl_2, −90 °C[a]	0.001	Complex mixture
23	$EtAlCl_2$ (5.0 eq.), CH_2Cl_2, −78 °C[a]	0.001	Complex mixture; trace product formation detected
24	$EtAlCl_2$ (3.0 eq.), CH_2Cl_2, −78 °C[c]	0.001	Complex mixture

[a] Substrate in CH_2Cl_2 (precooled by passing the needle through dry ice) was added to a solution of Lewis acid in CH_2Cl_2 at −78 °C.

[b] No tricyclization product was obtained if the corresponding trimethylsilyl ether (OTMS) was used under the same conditions as starting material instead of the tertiary alcohol.

[c] $EtAlCl_2$ in CH_2Cl_2 (precooled by passing the needle through dry ice) was added to a solution of substrate in CH_2Cl_2 at −78 °C.

Note: No signals corresponding to **S6** were observed in ^{1}H-NMR analysis of the crude reaction mixture.

4. NMR Spectra

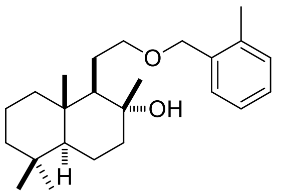

^{1}H-NMR (500 MHz, $CDCl_3$)

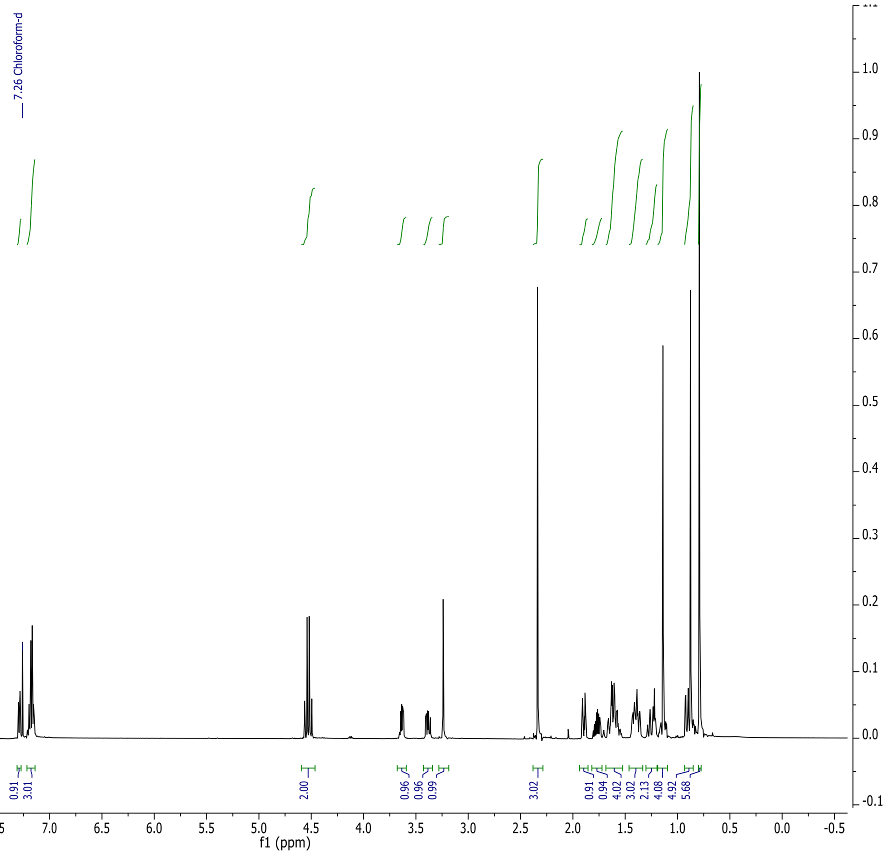

^{13}C-NMR (126 MHz, $CDCl_3$)

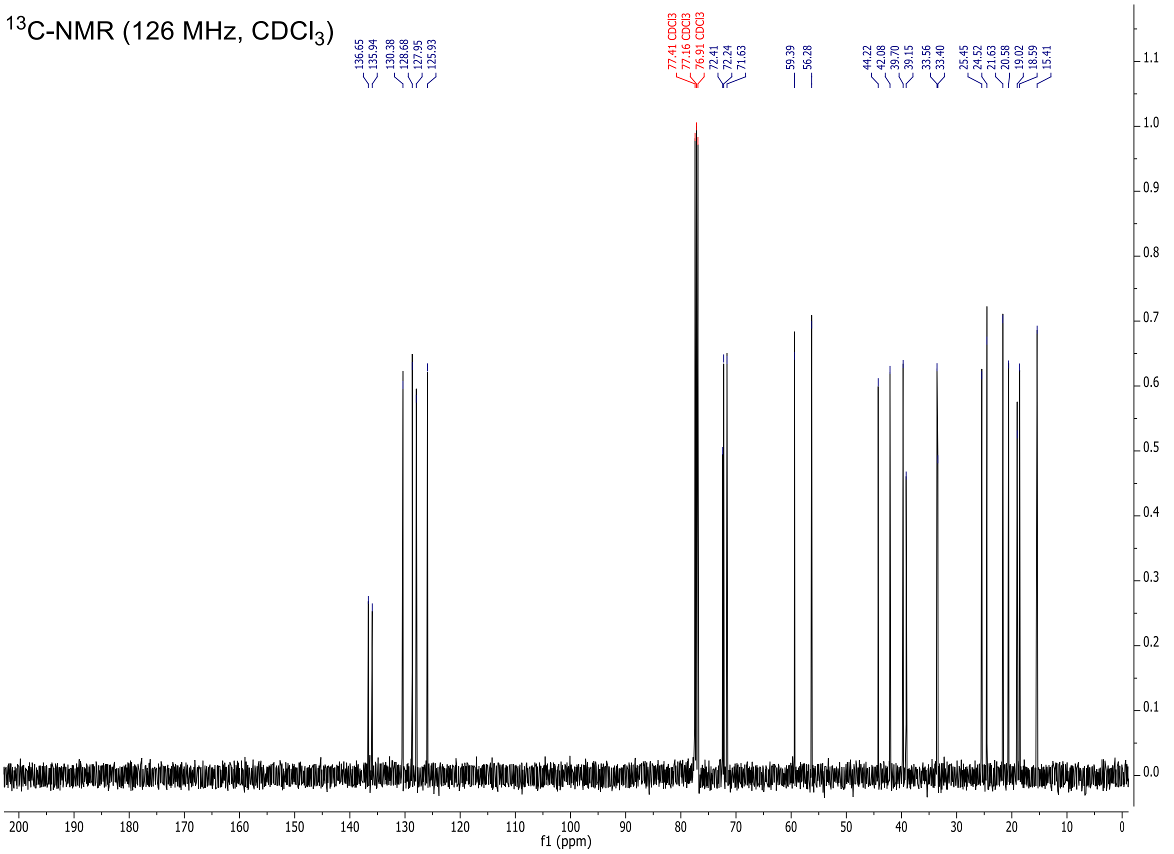

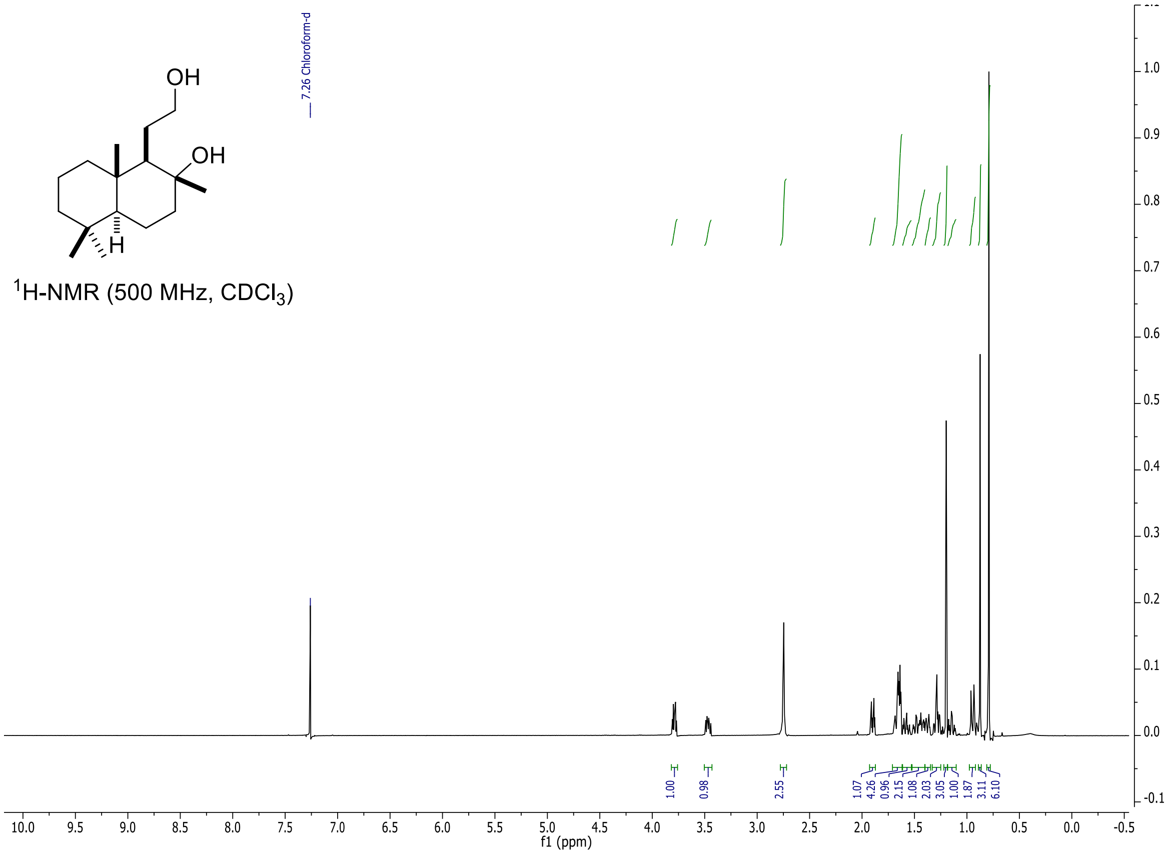

^{1}H-NMR (500 MHz, $CDCl_3$)

^{13}C-NMR (126 MHz, $CDCl_3$)

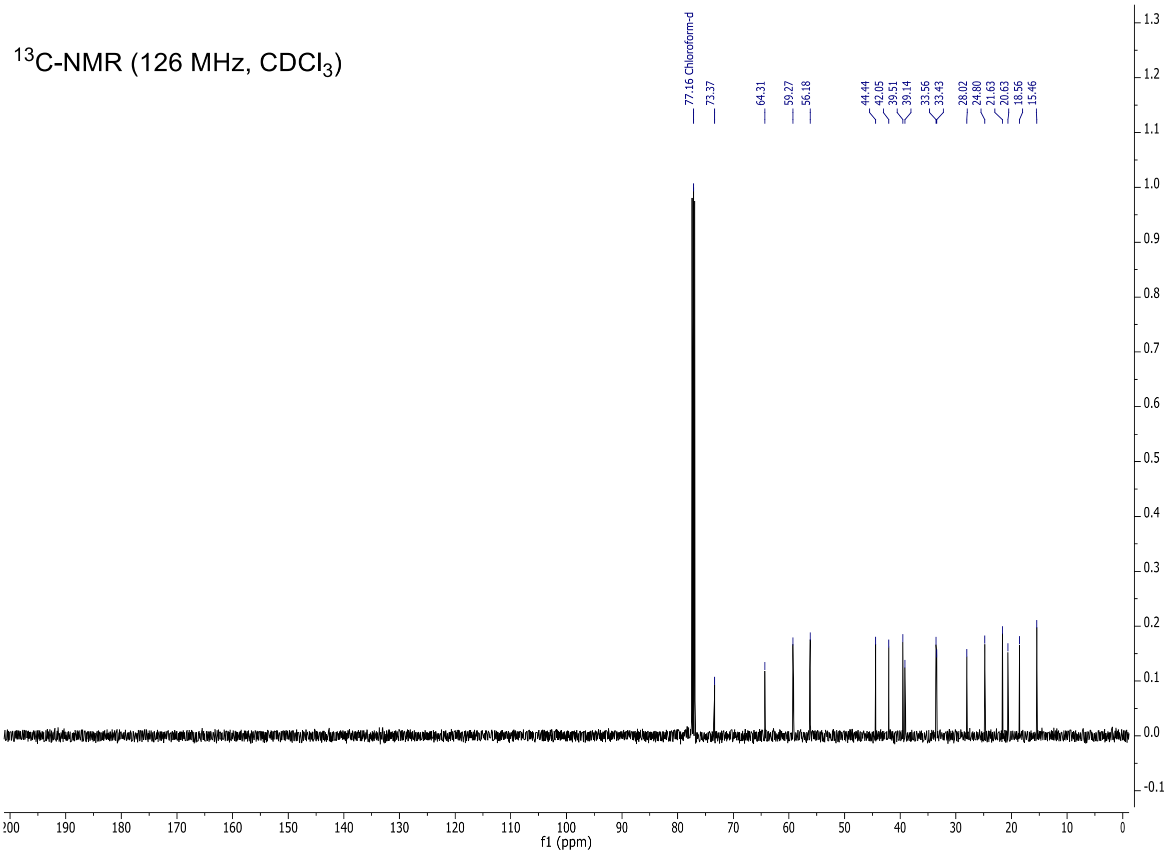

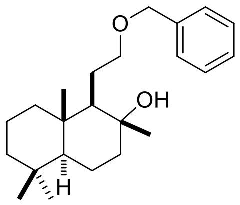

^{1}H-NMR (700 MHz, $CDCl_3$)

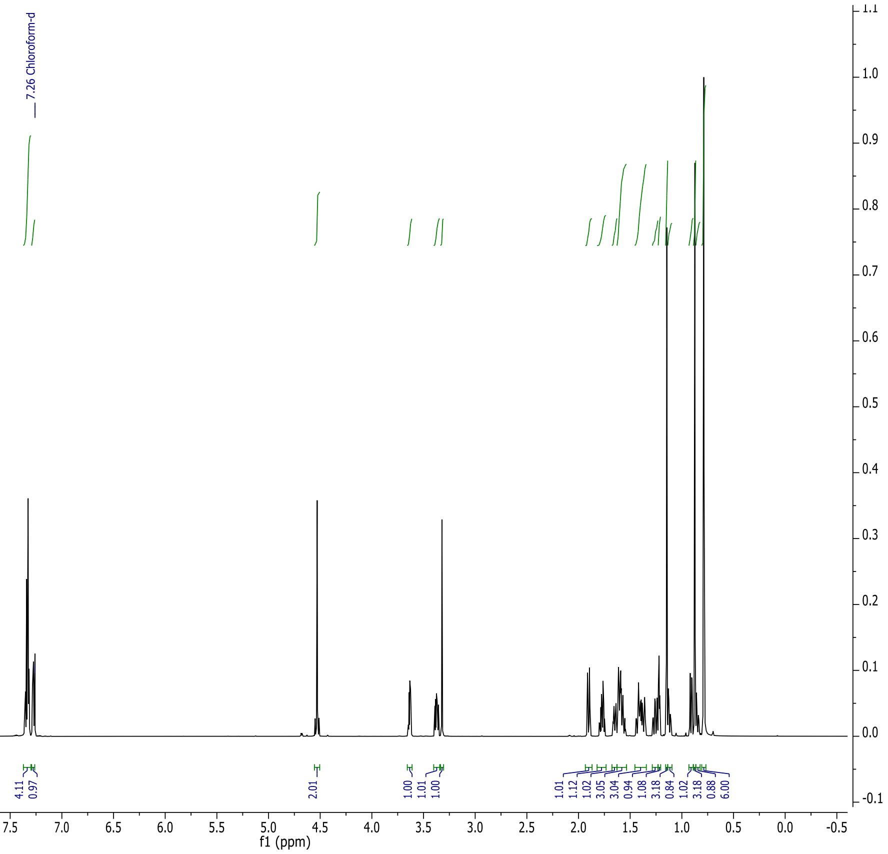

^{13}C-NMR (176 MHz, $CDCl_3$)

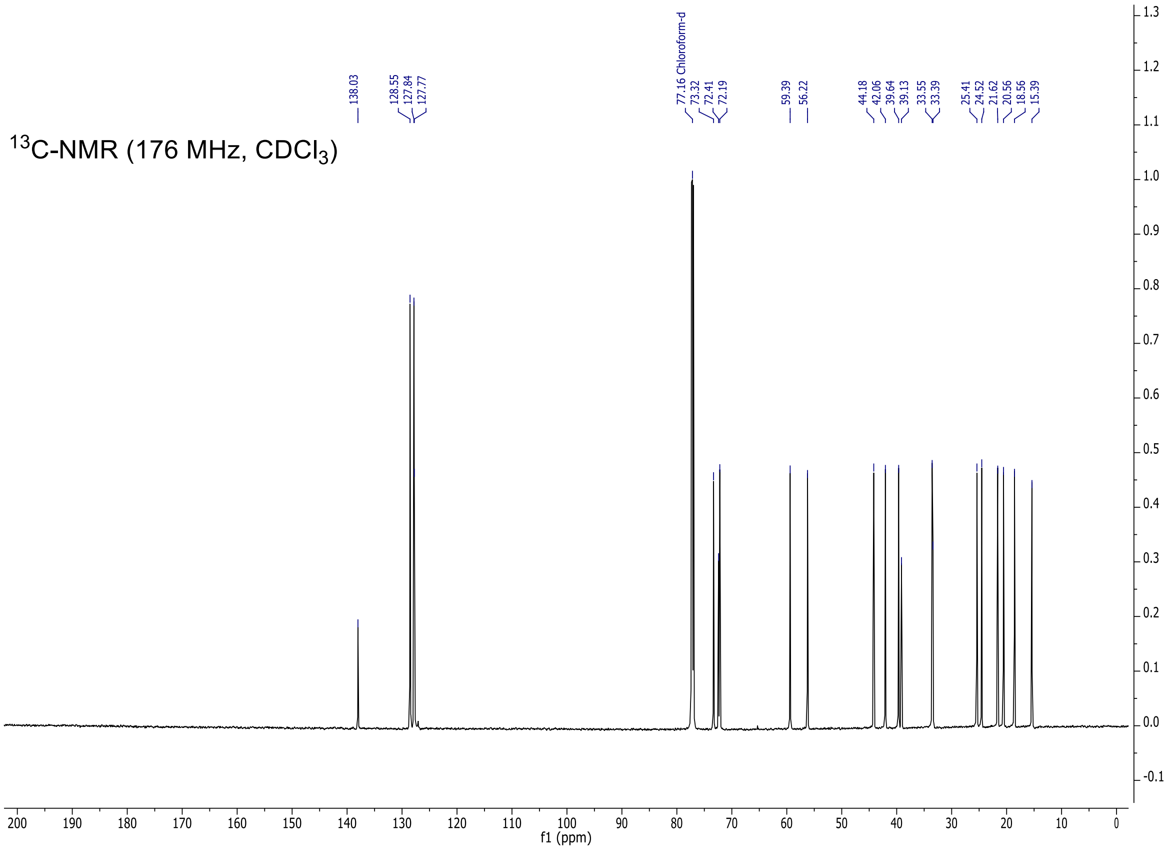

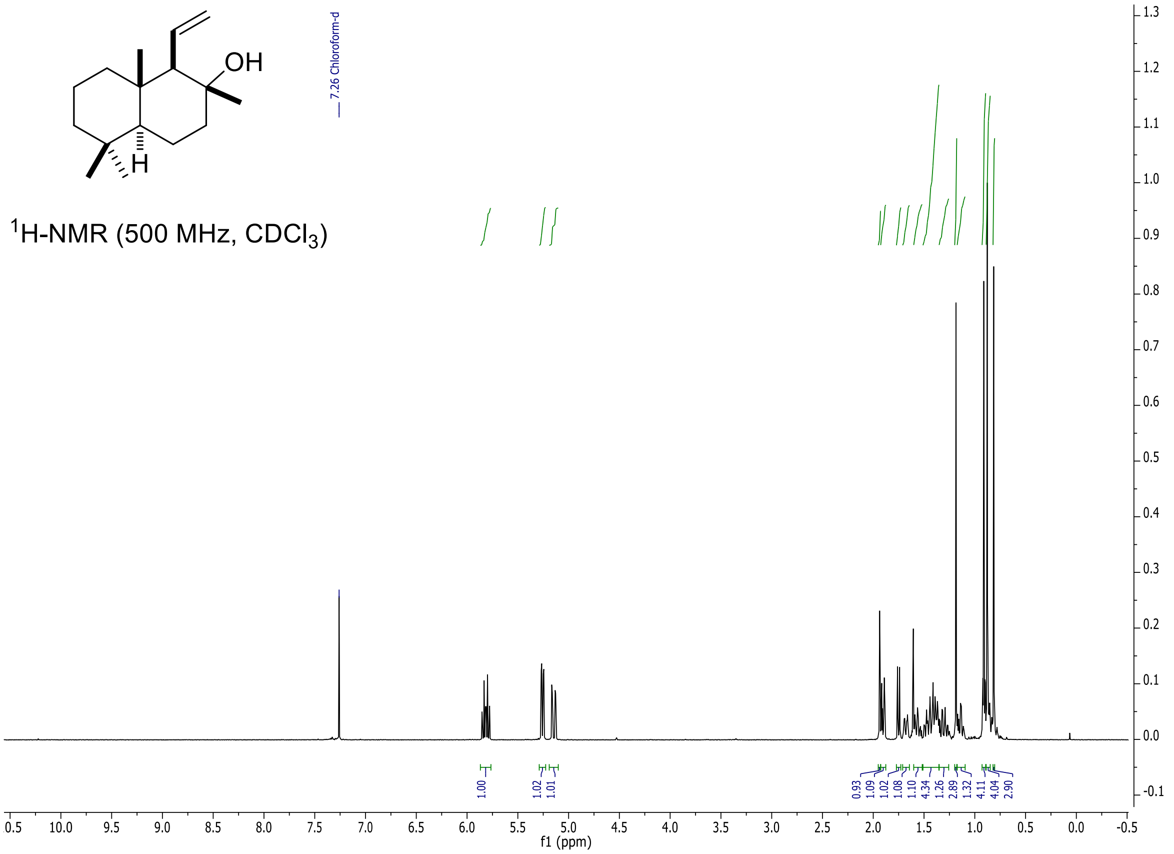

OH
H
^{1}H-NMR (500 MHz, $CDCl_3$)
7.26 Chloroform-d
1.00
1.02
1.01
0.93
1.09
1.02
1.08
1.10
4.34
1.26
2.89
1.32
4.11
4.04
2.90
f1 (ppm)

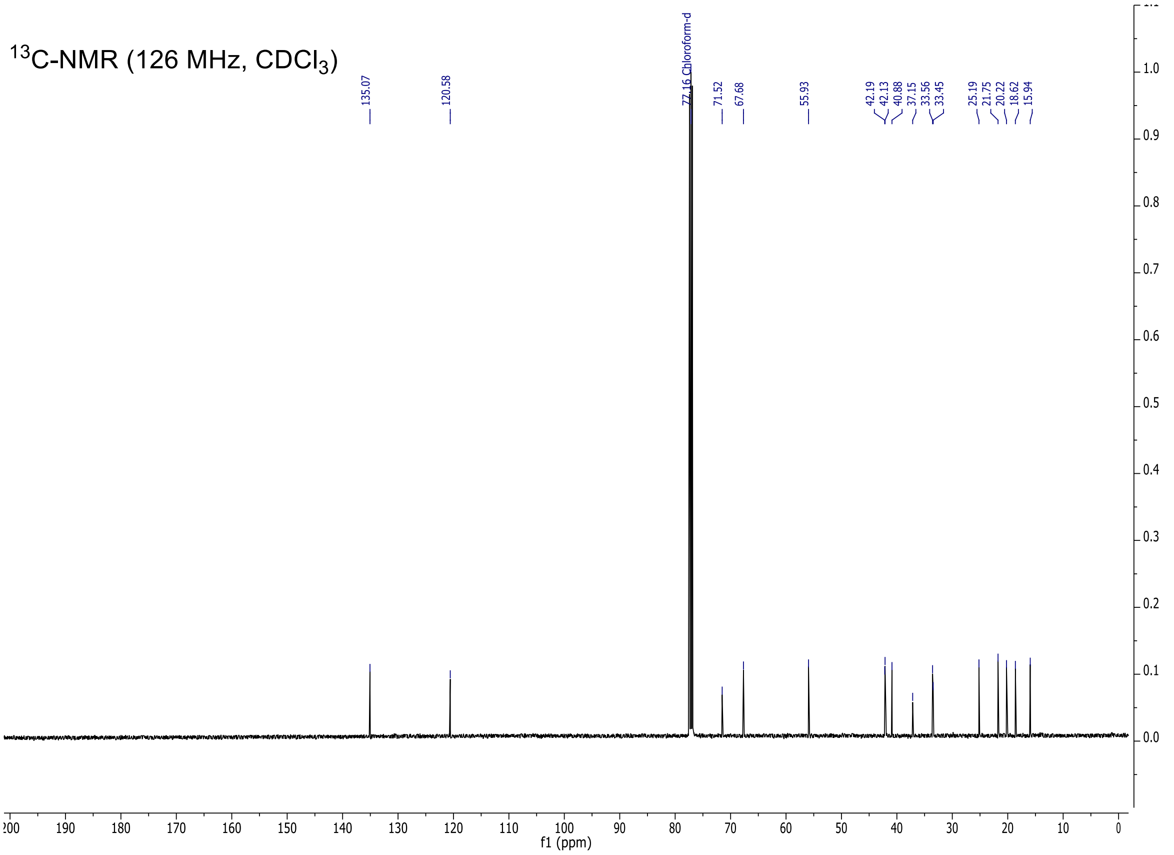

^{13}C-NMR (126 MHz, $CDCl_3$)
135.07
120.58
77.16 Chloroform-d
71.52
67.68
55.93
42.19
42.13
40.88
37.15
33.56
33.45
25.19
21.75
20.22
18.62
15.94
f1 (ppm)

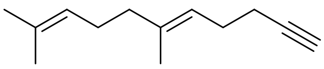

^{1}H-NMR (700 MHz, $CDCl_3$)

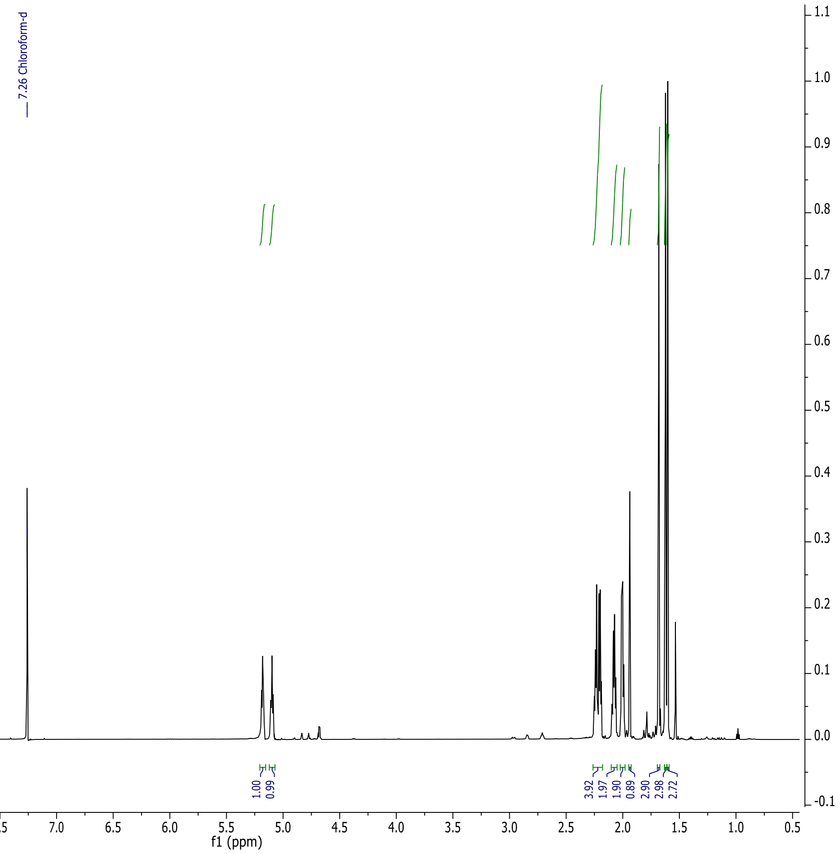

^{13}C-NMR (176 MHz, $CDCl_3$)

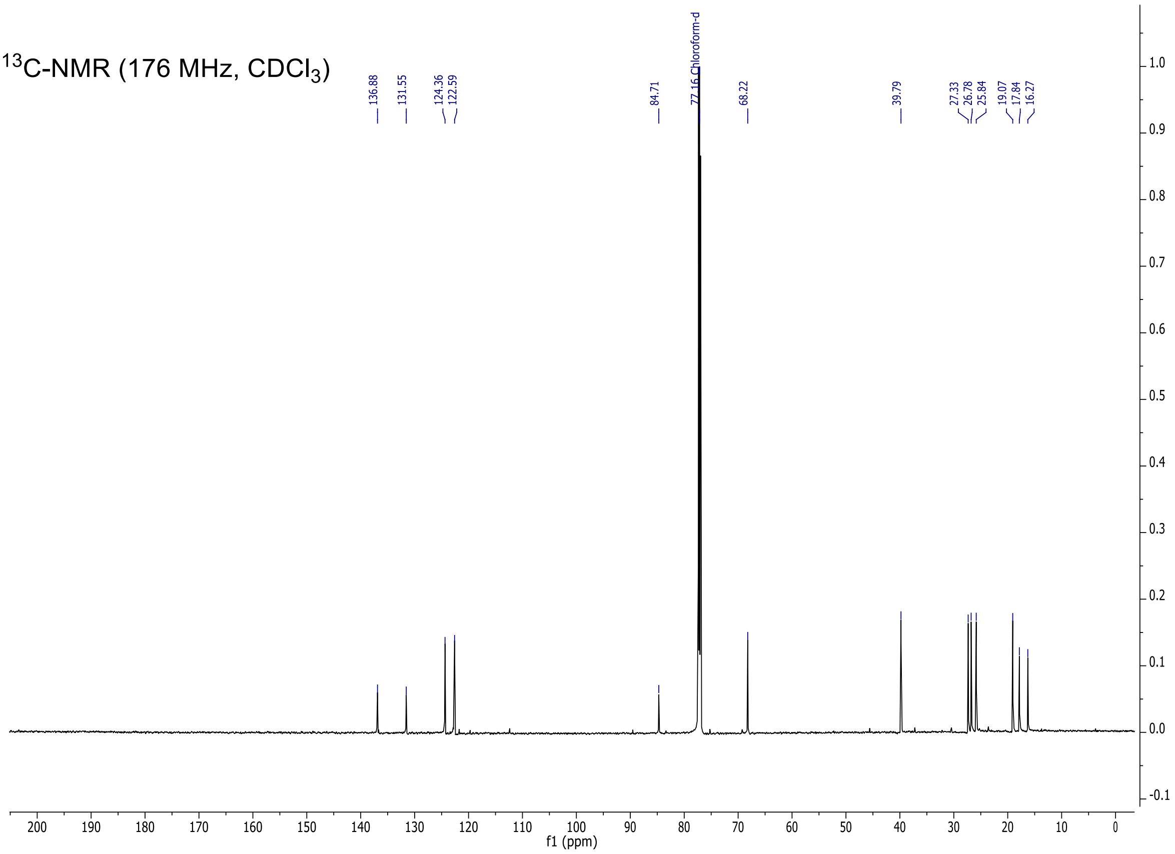

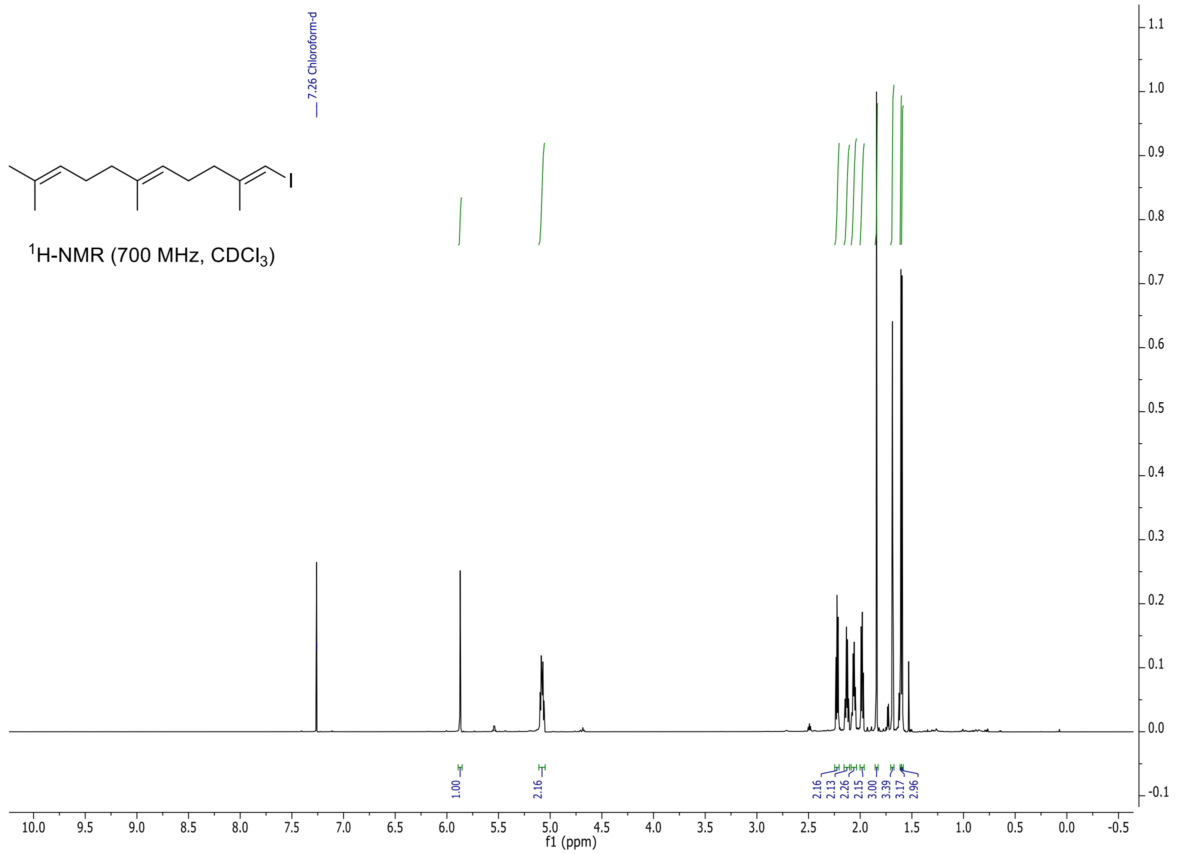

I
^{1}H-NMR (700 MHz, CDCl$_3$)
7.26 Chloroform-d
1.00
2.16
2.16
2.13
2.26
2.15
3.00
3.39
3.17
2.96
10.0 9.5 9.0 8.5 8.0 7.5 7.0 6.5 6.0 5.5 5.0 4.5 4.0 3.5 3.0 2.5 2.0 1.5 1.0 0.5 0.0 -0.5
f1 (ppm)

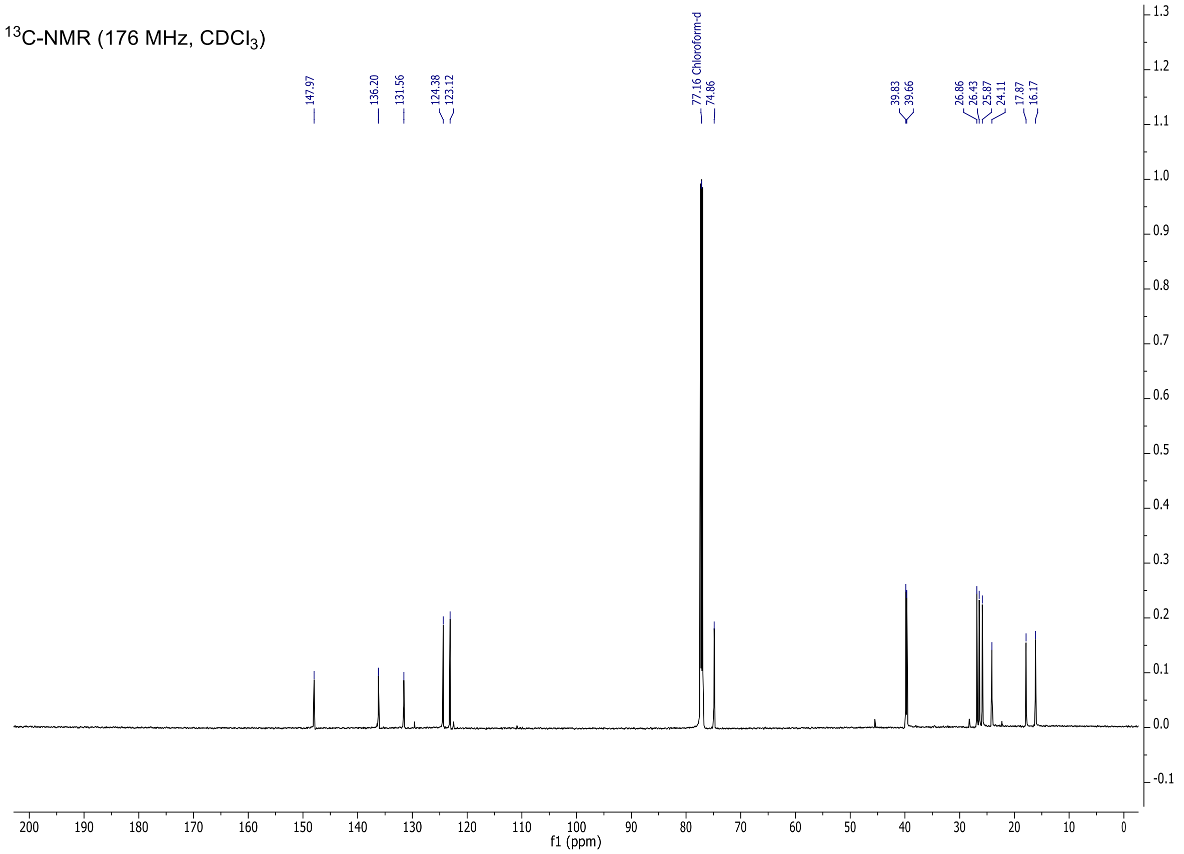

^{13}C-NMR (176 MHz, CDCl$_3$)
147.97
136.20
131.56
124.38
123.12
77.16 Chloroform-d
74.86
39.83
39.66
26.86
26.43
25.87
24.11
17.87
16.17
200 190 180 170 160 150 140 130 120 110 100 90 80 70 60 50 40 30 20 10 0
f1 (ppm)

^{1}H-NMR (700 MHz, $CDCl_3$)

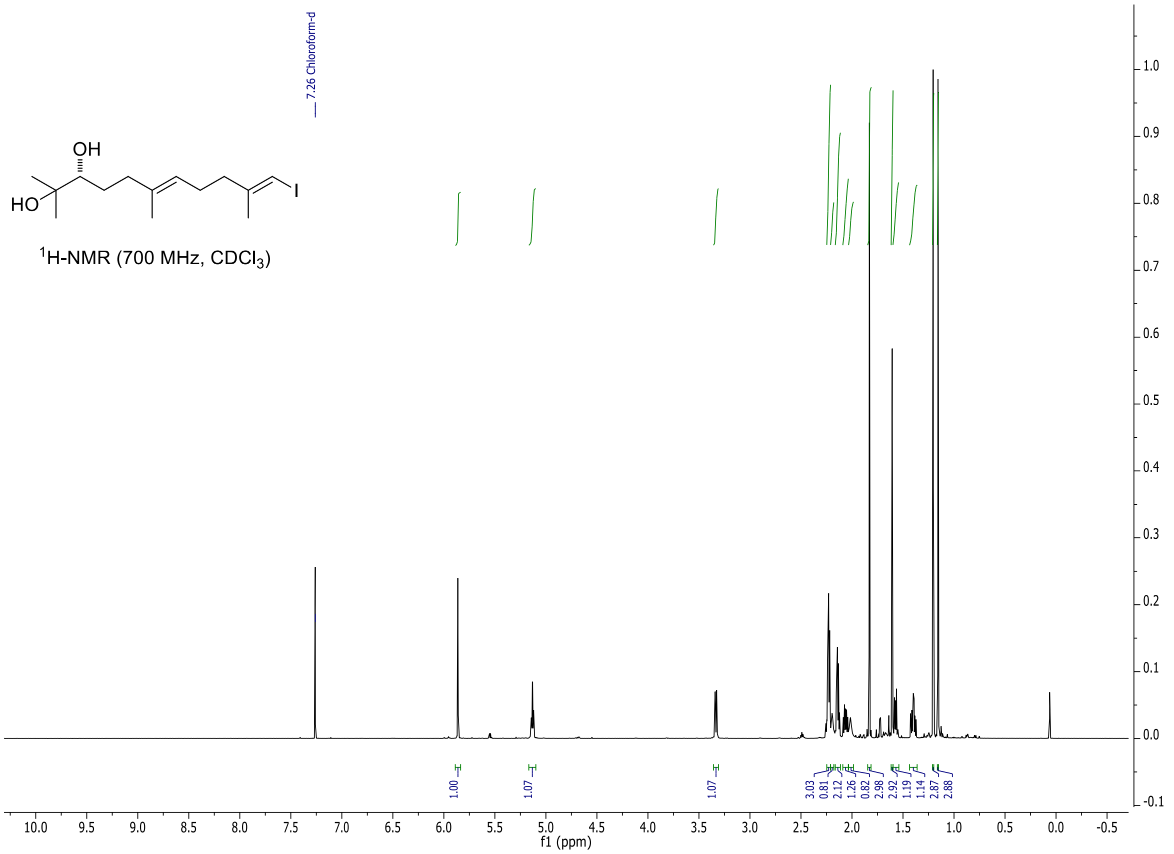

^{13}C-NMR (176 MHz, $CDCl_3$)

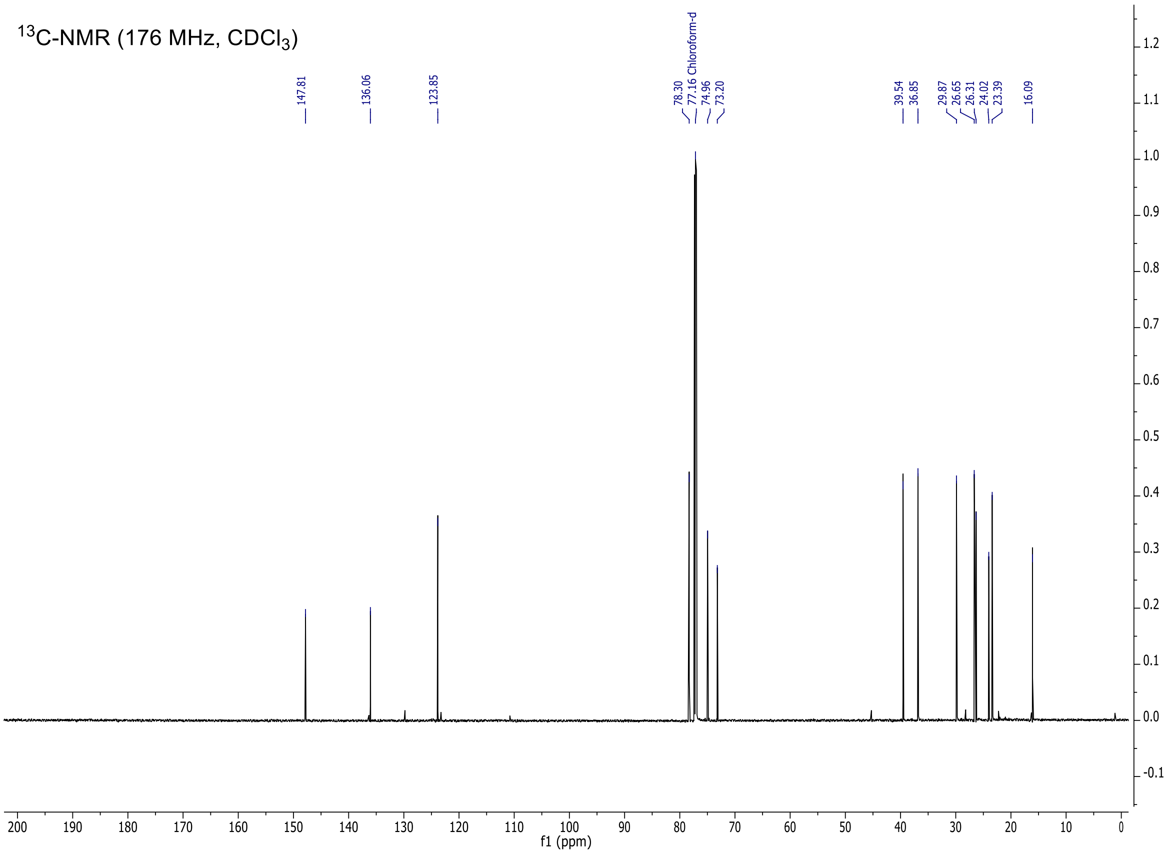

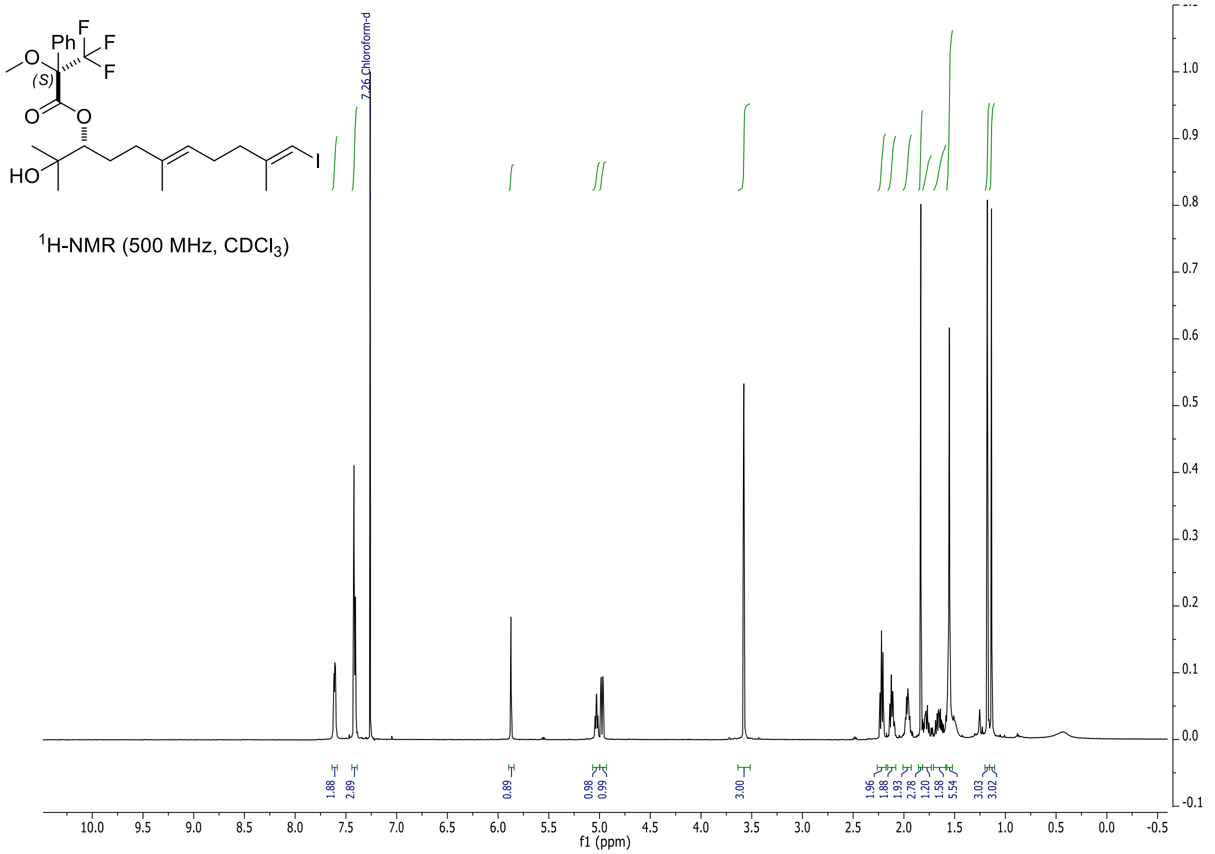
(S)
1H-NMR (500 MHz, CDCl3)
7.26 Chloroform-d
f1 (ppm)

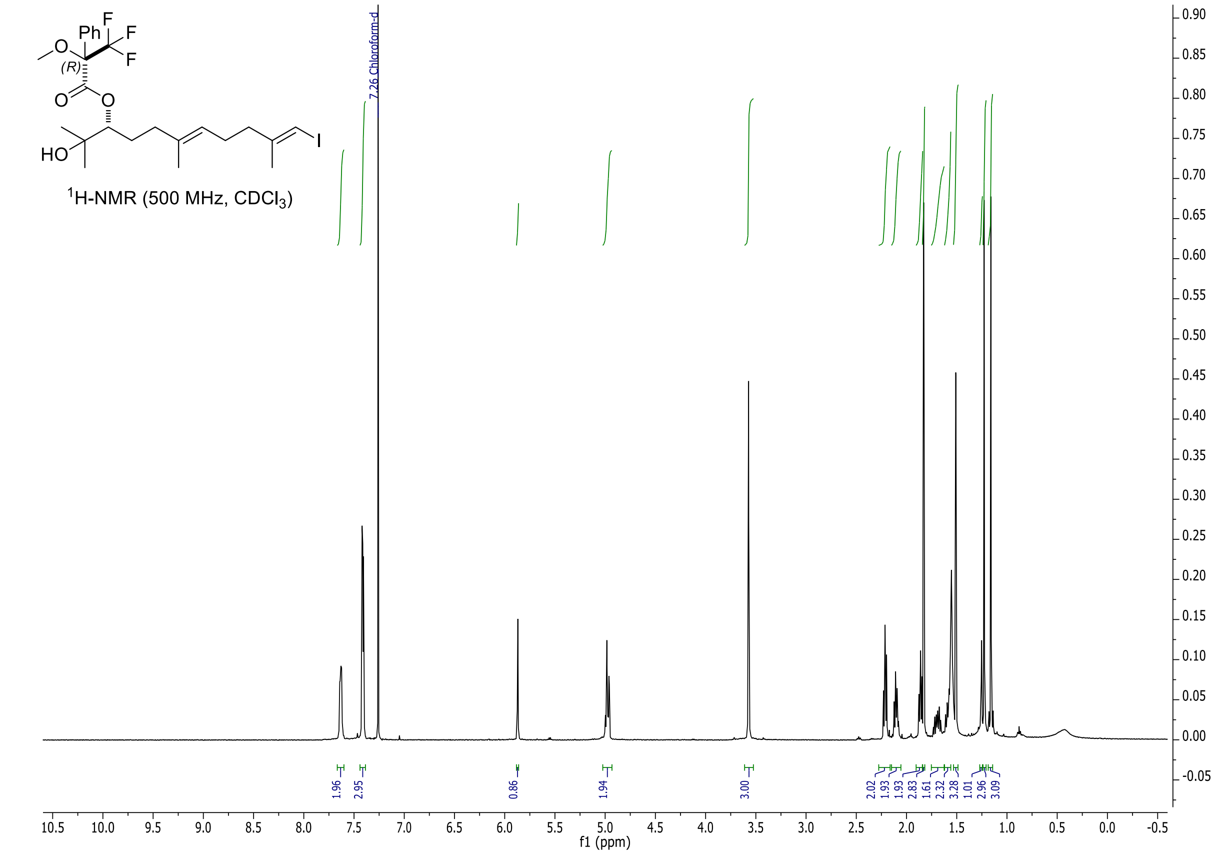
(R)
1H-NMR (500 MHz, CDCl3)
7.26 Chloroform-d
f1 (ppm)

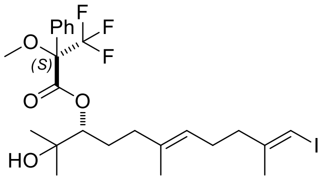

^1H-^1H-COSY (700 MHz, $CDCl_3$)

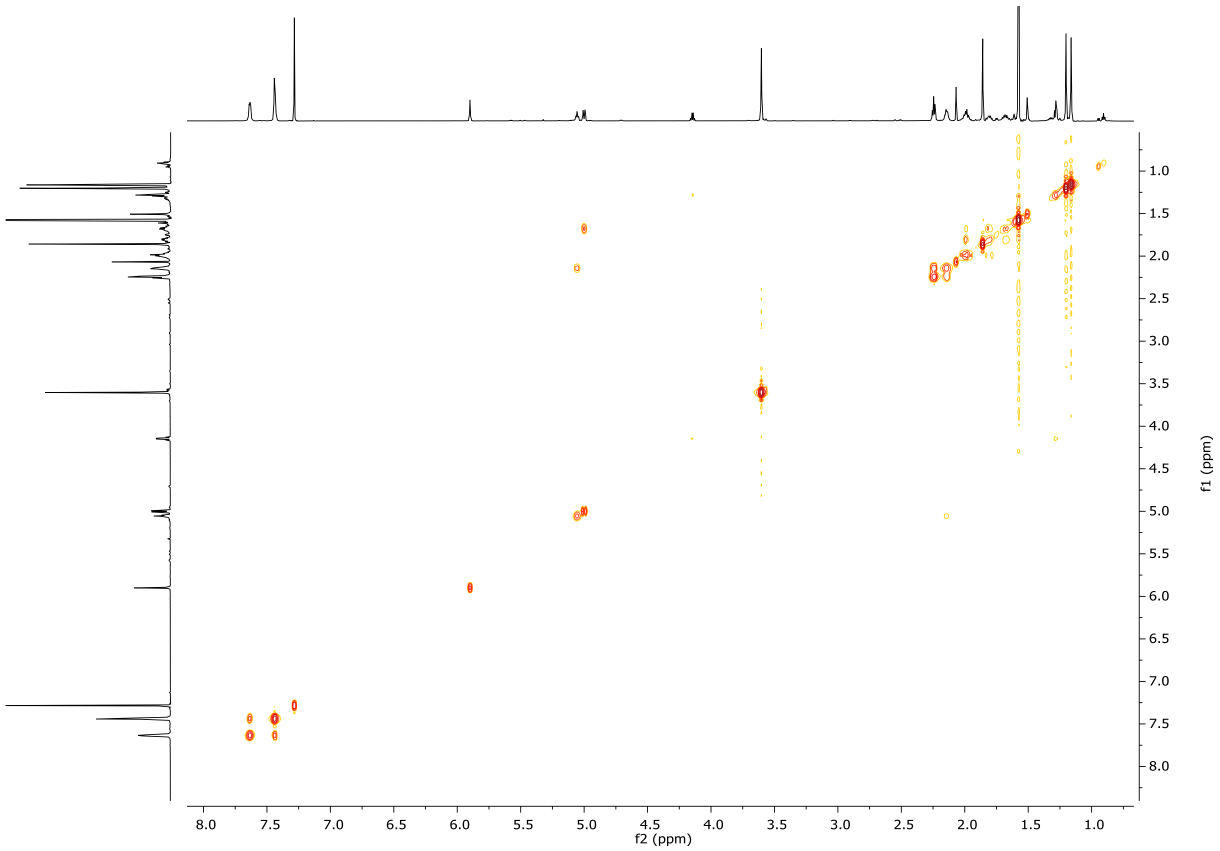

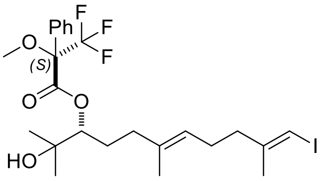

HMQC (700 MHz, $CDCl_3$)

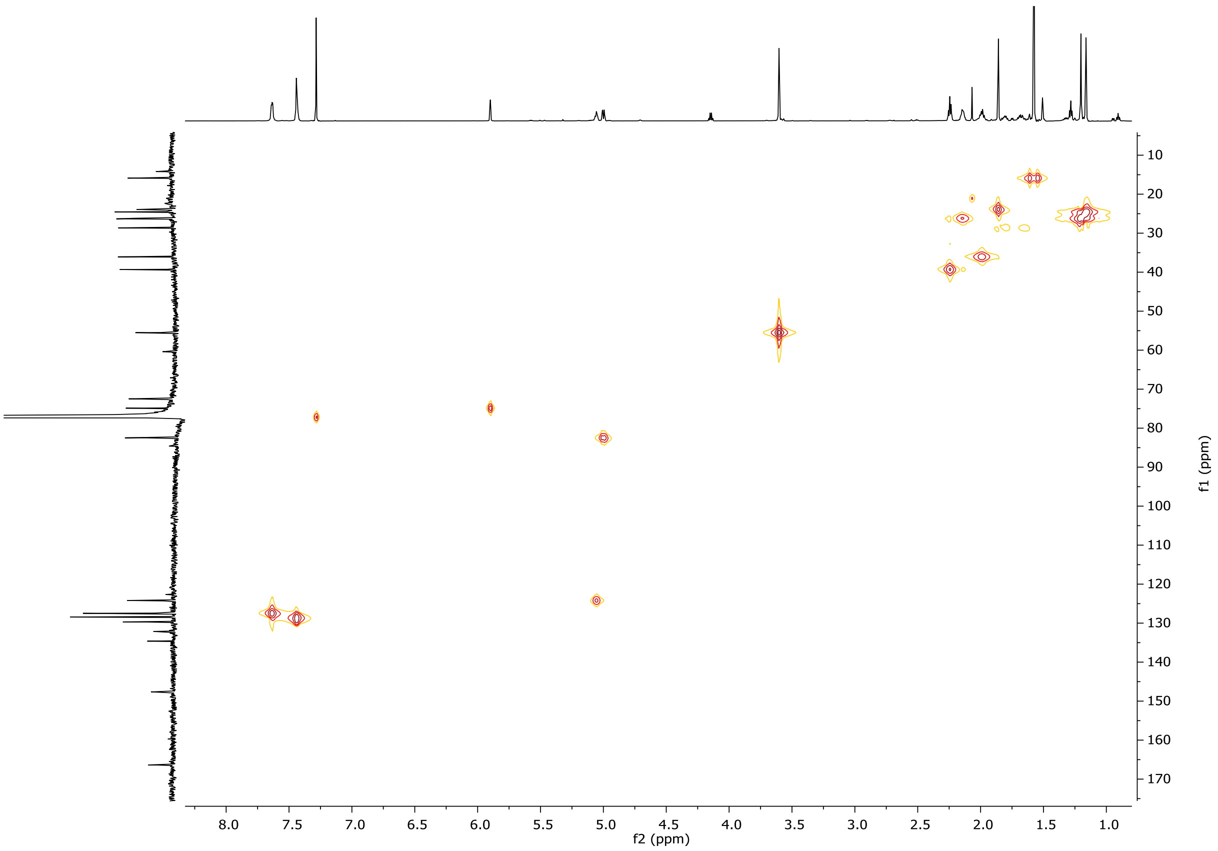

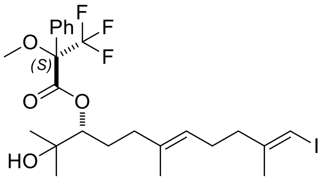

HMBC (700 MHz, $CDCl_3$)

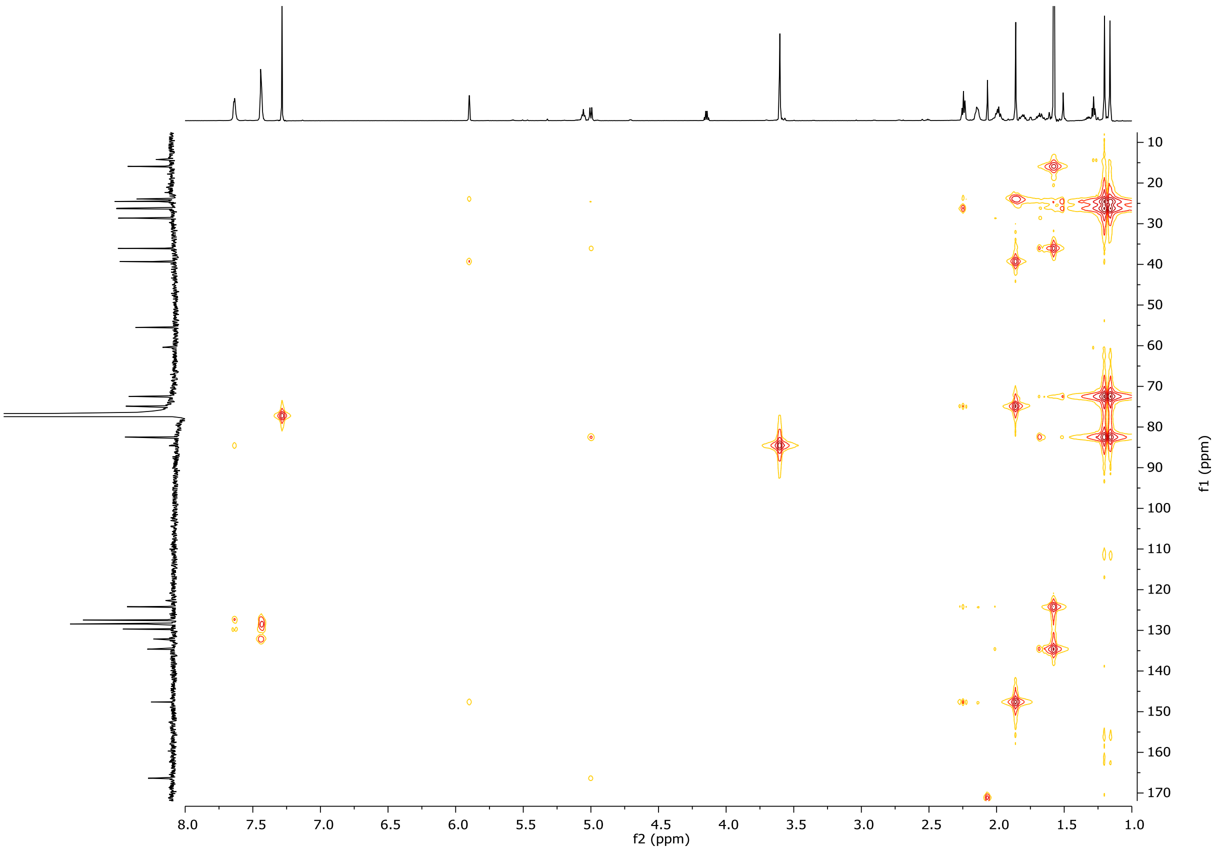

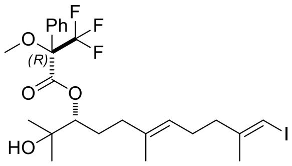

^{1}H-^{1}H-COSY (700 MHz, $CDCl_3$)

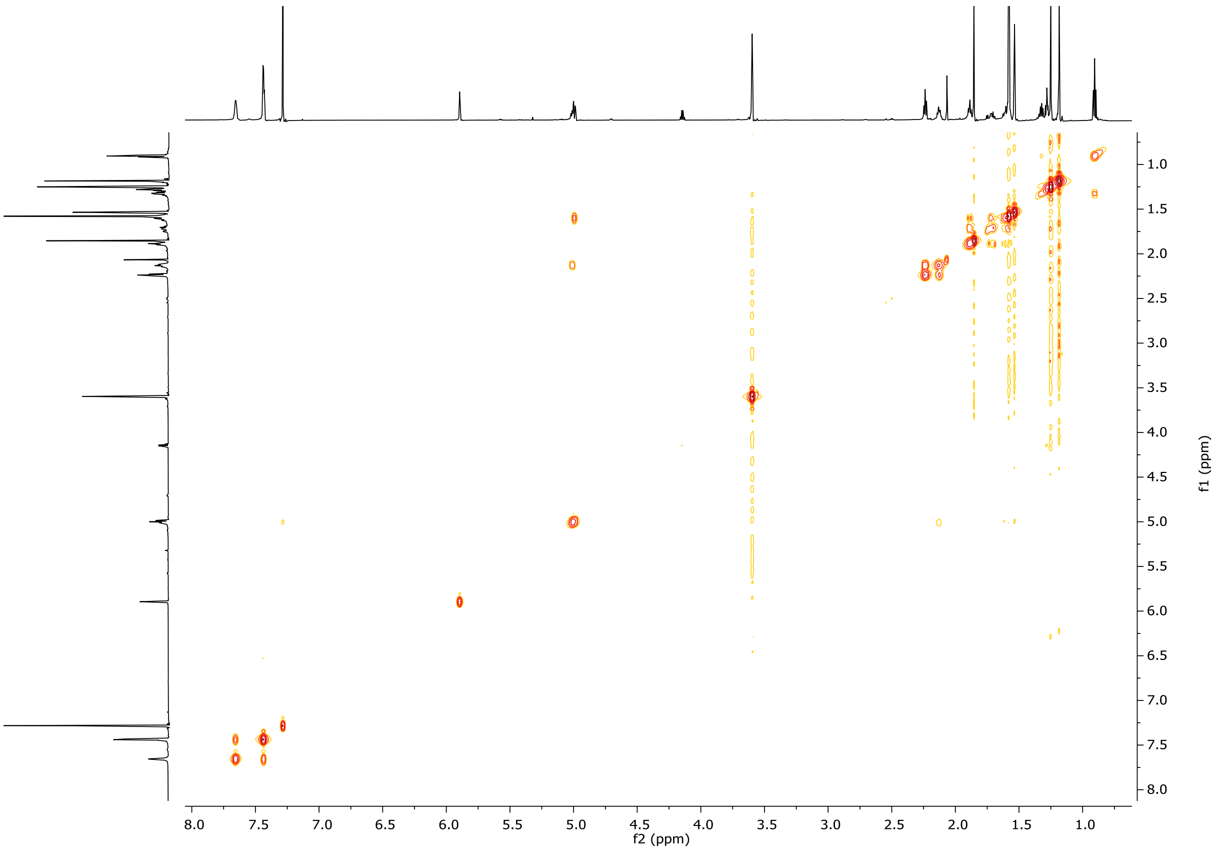

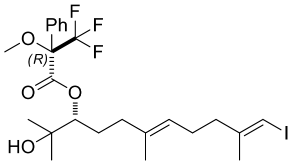

HMQC (700 MHz, CDCl$_3$)

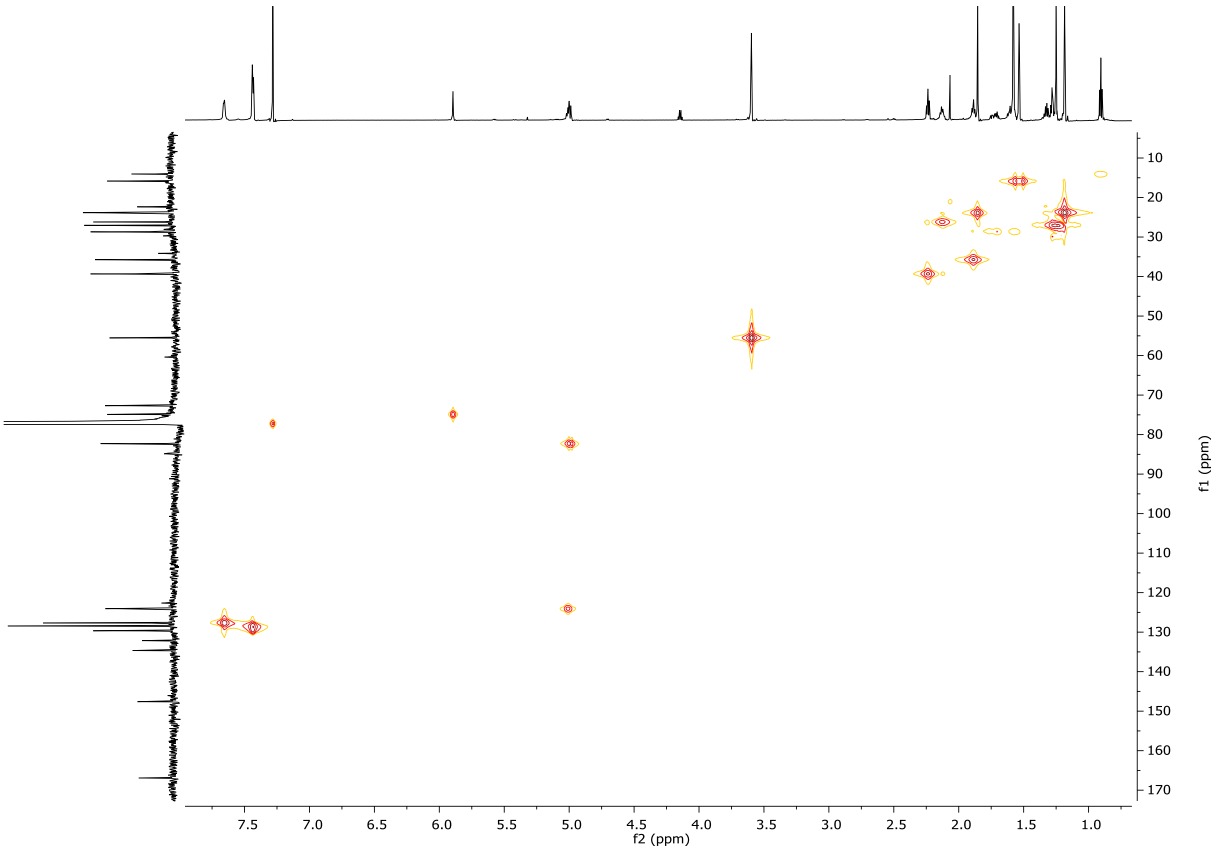

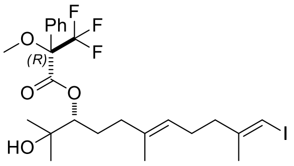

HMBC (700 MHz, $CDCl_3$)

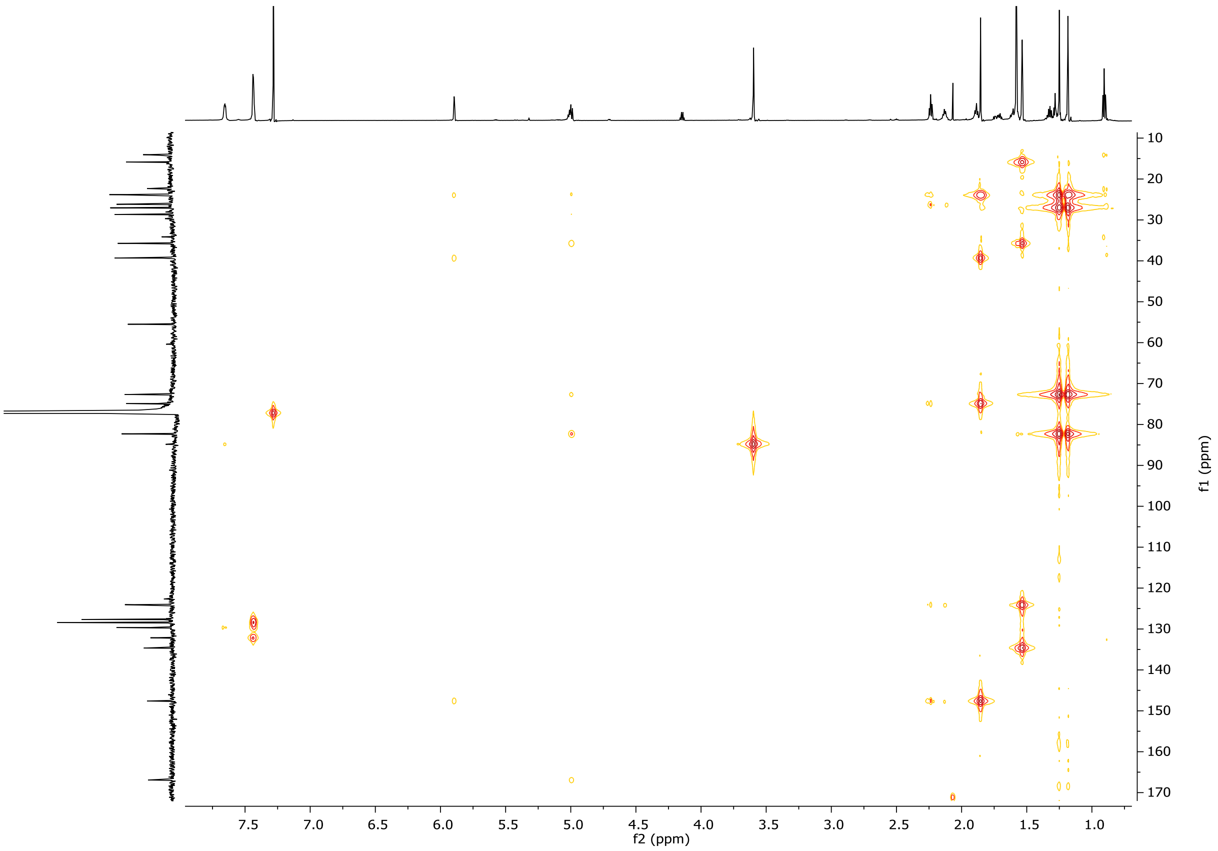

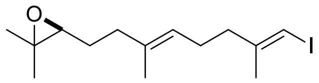

^{1}H-NMR (700 MHz, $CDCl_3$)

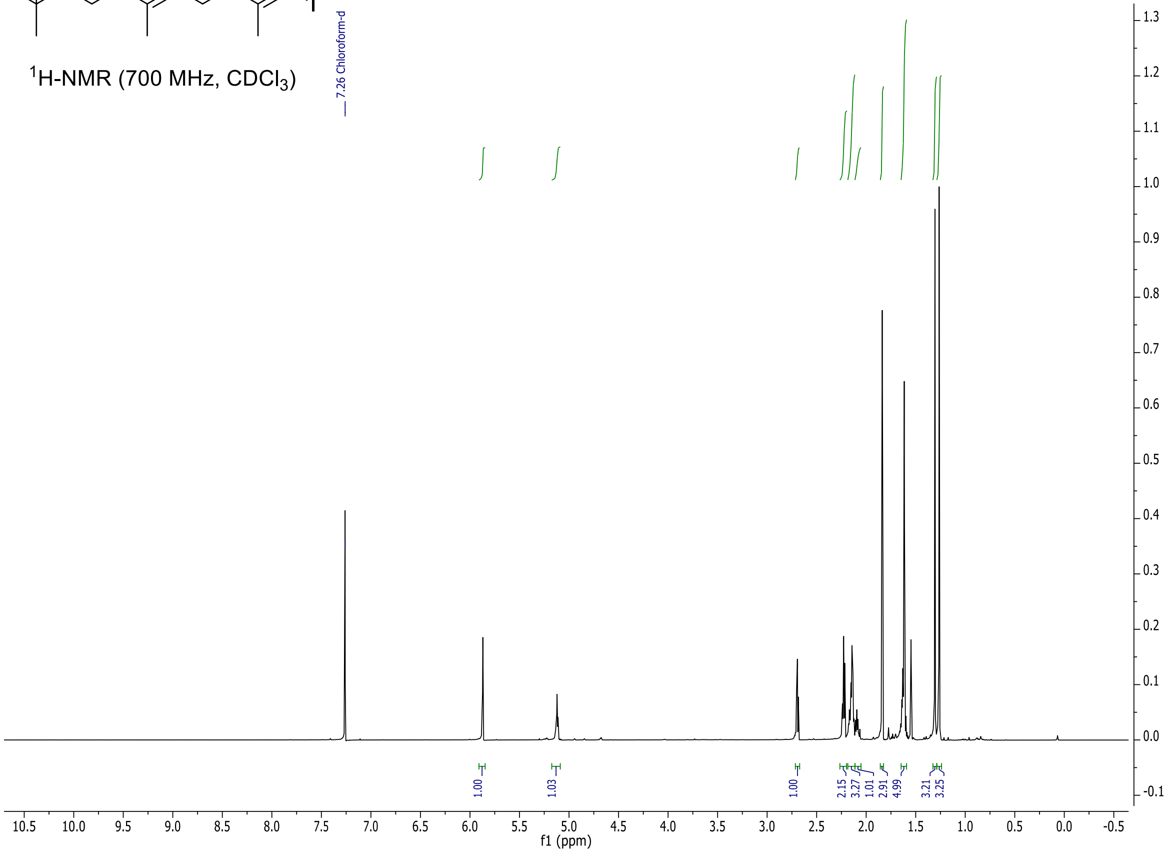

^{13}C-NMR (176 MHz, $CDCl_3$)

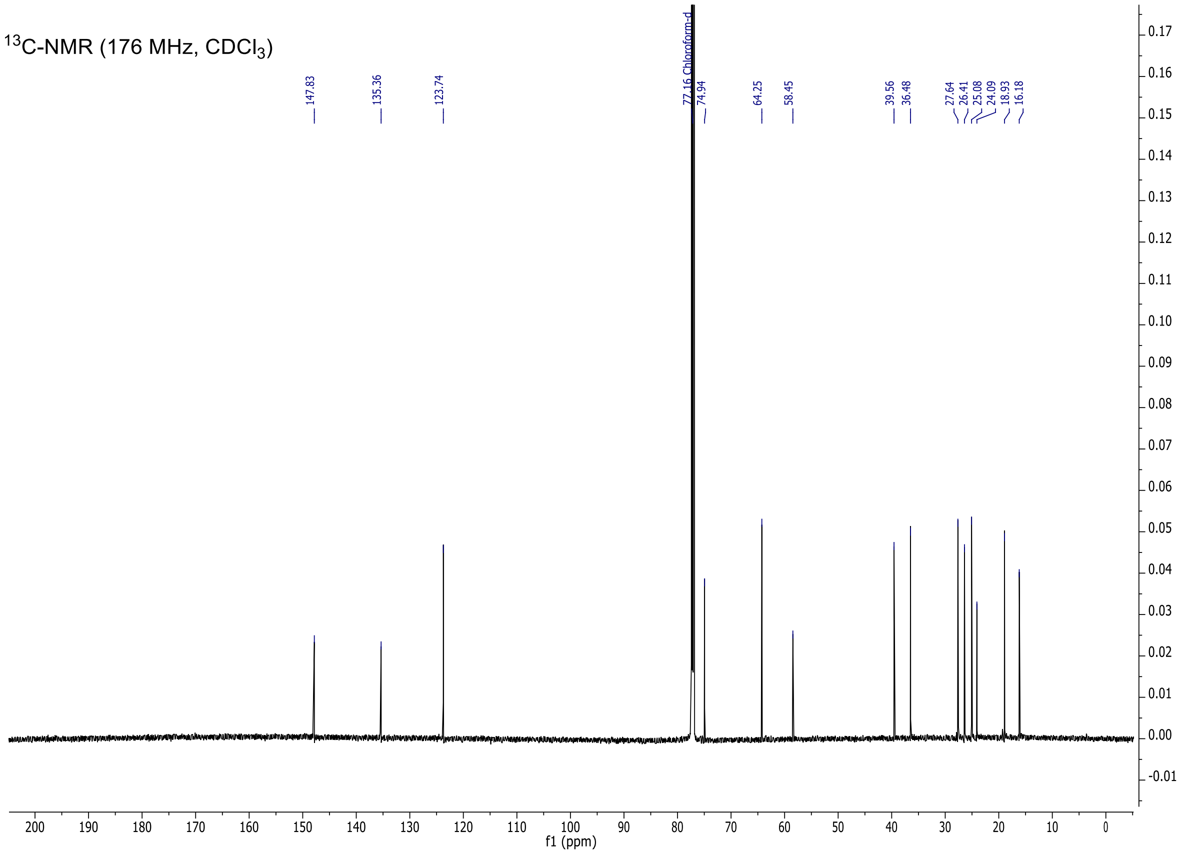

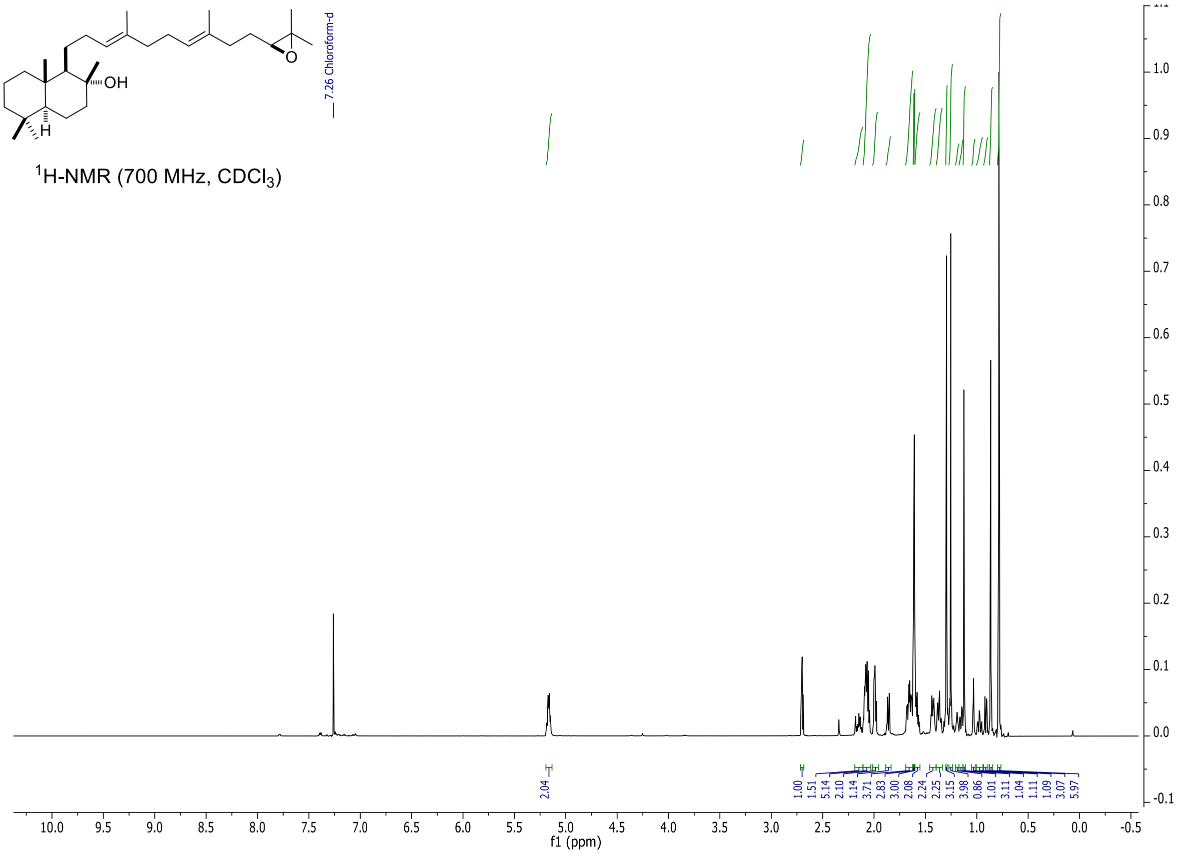

^{1}H-NMR (700 MHz, $CDCl_3$)

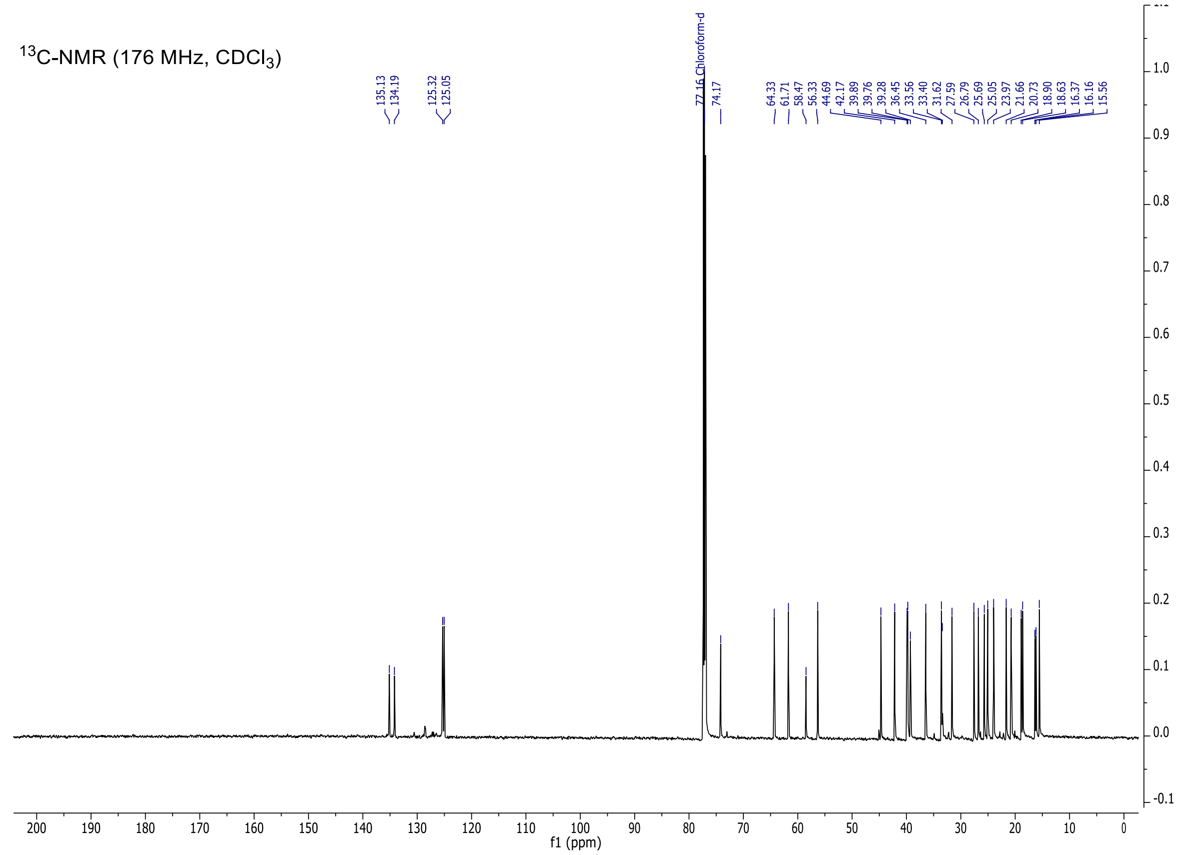

^{13}C-NMR (176 MHz, $CDCl_3$)

^{1}H-NMR (700 MHz, C_6D_6)

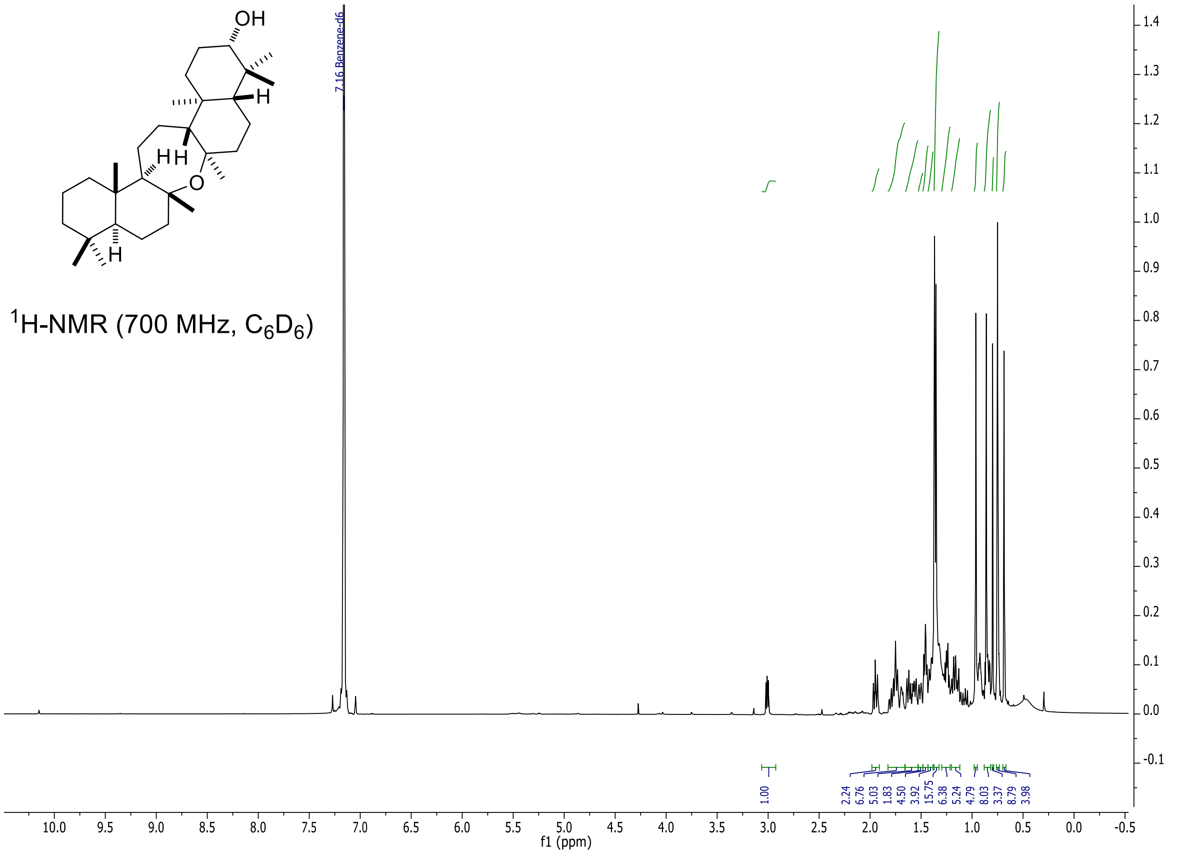

^{13}C-NMR (176 MHz, C_6D_6)

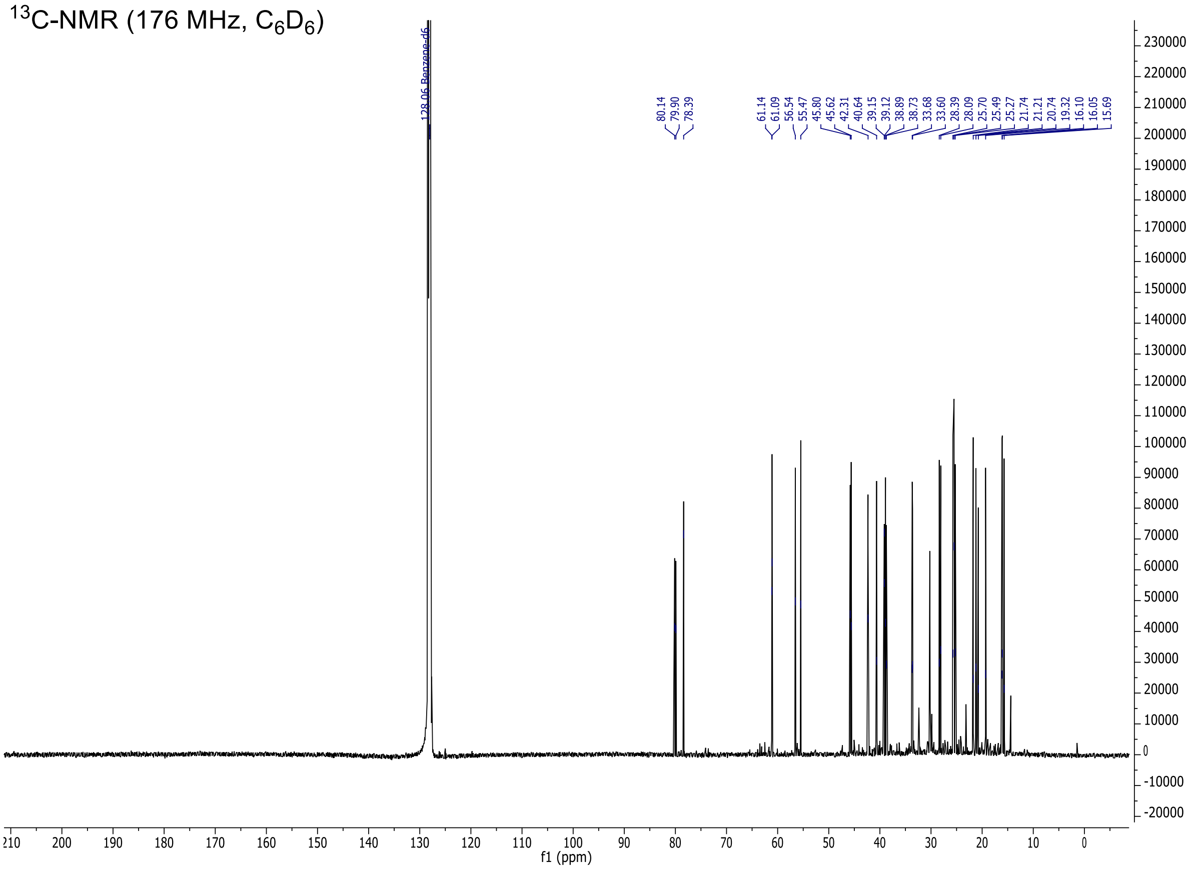

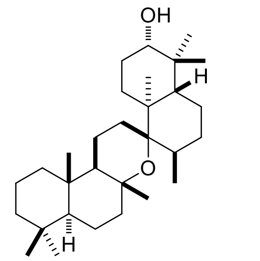

^{1}H-NMR (700 MHz, C_6D_6)

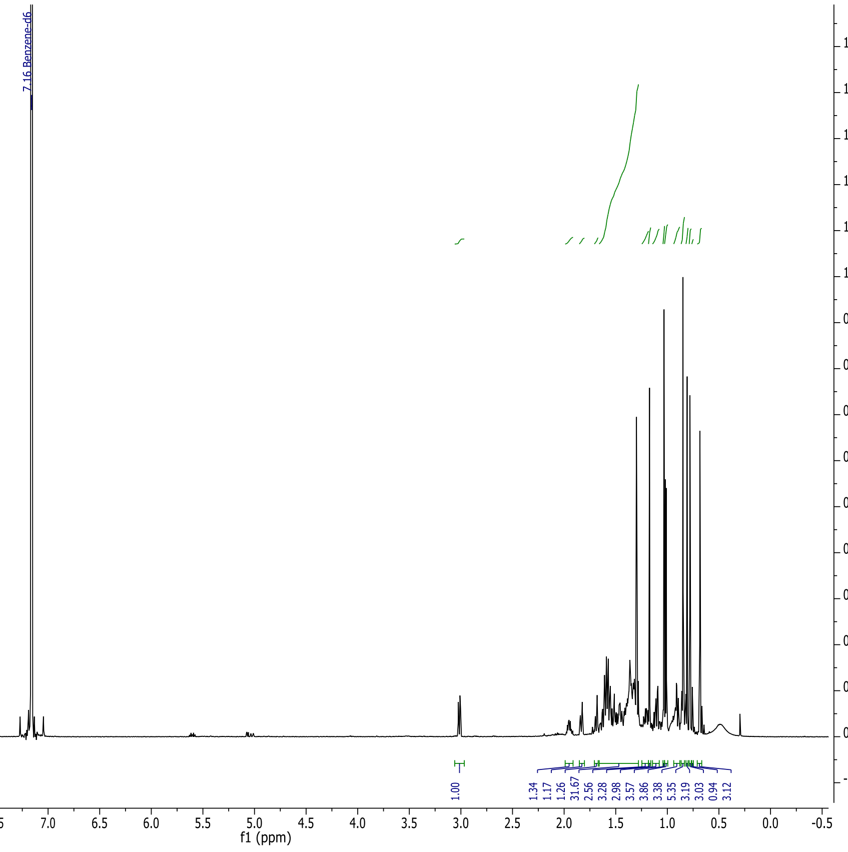

^{13}C-NMR (176 MHz, C_6D_6)

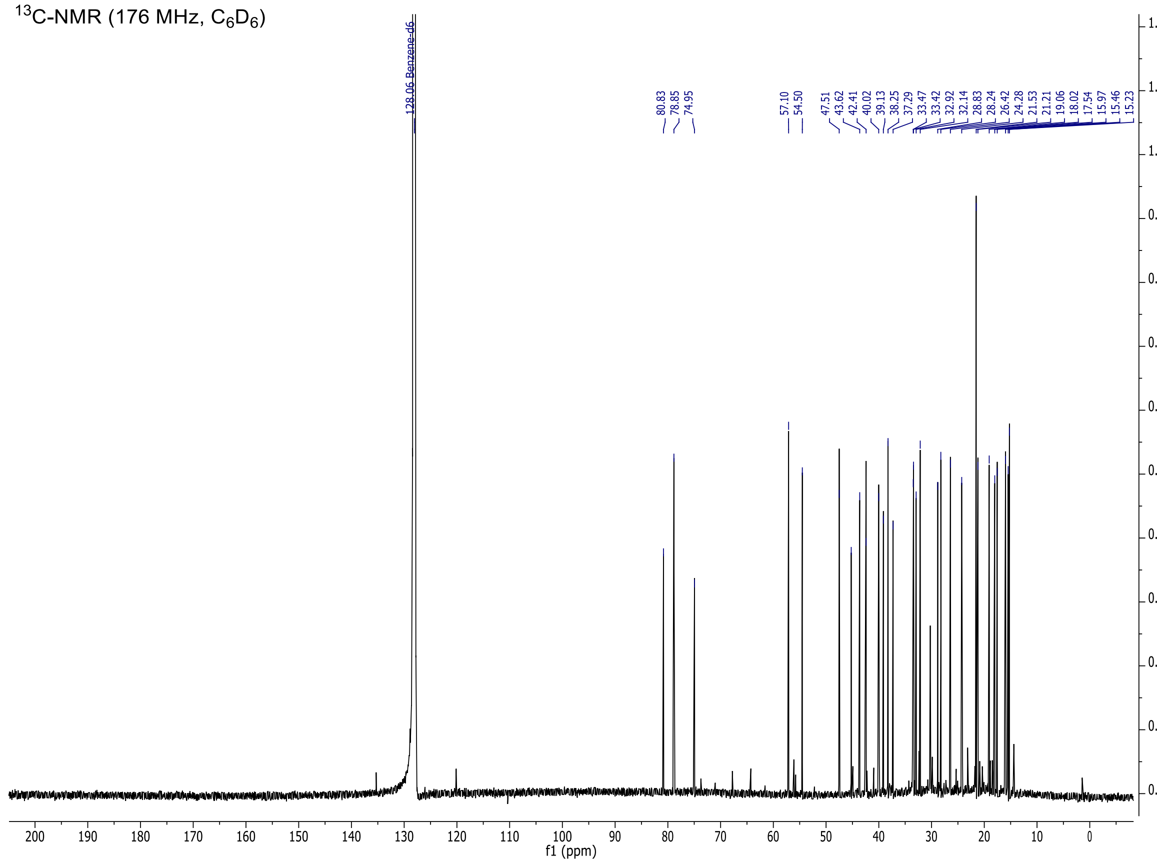

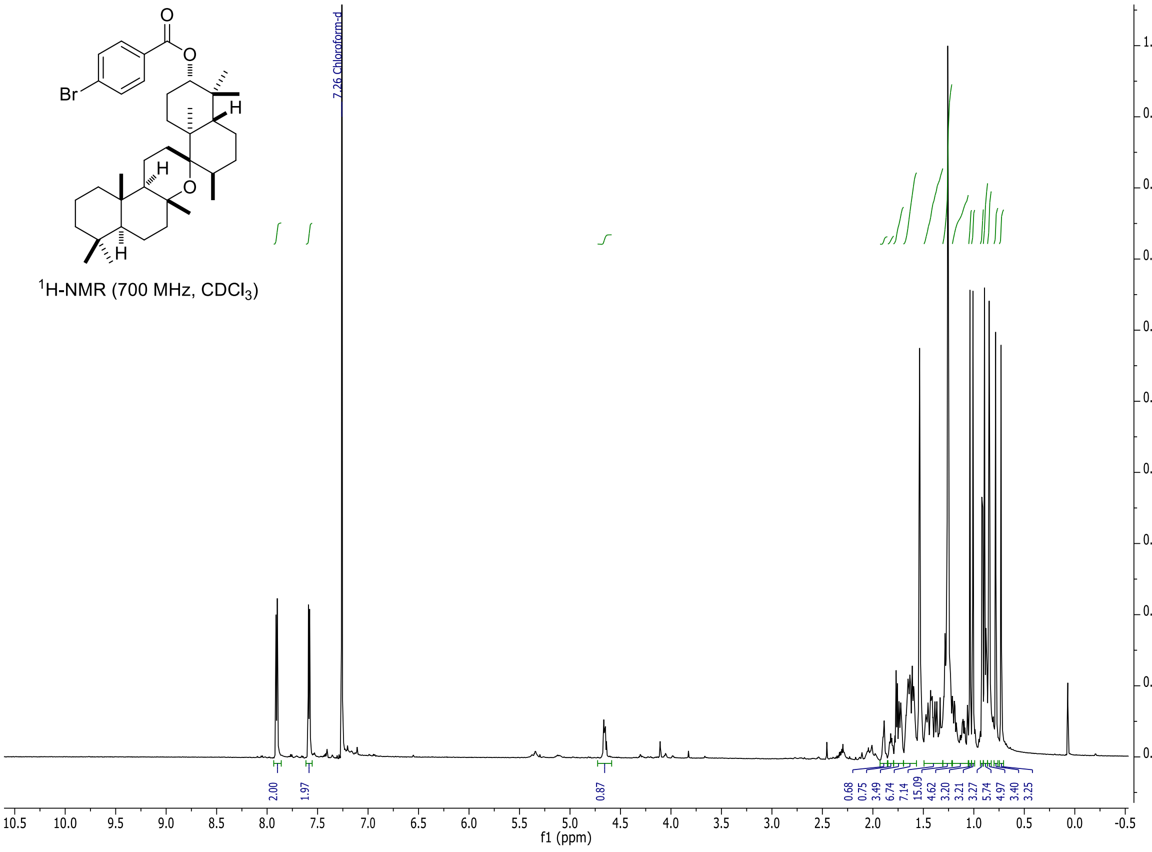
Br
O
O
H
H
O
H
^{1}H-NMR (700 MHz, CDCl$_3$)
7.26 Chloroform-d
2.00
1.97
0.87
0.68
0.75
3.49
6.74
7.14
15.09
4.62
3.20
3.21
3.27
5.74
4.97
3.40
3.25
f1 (ppm)

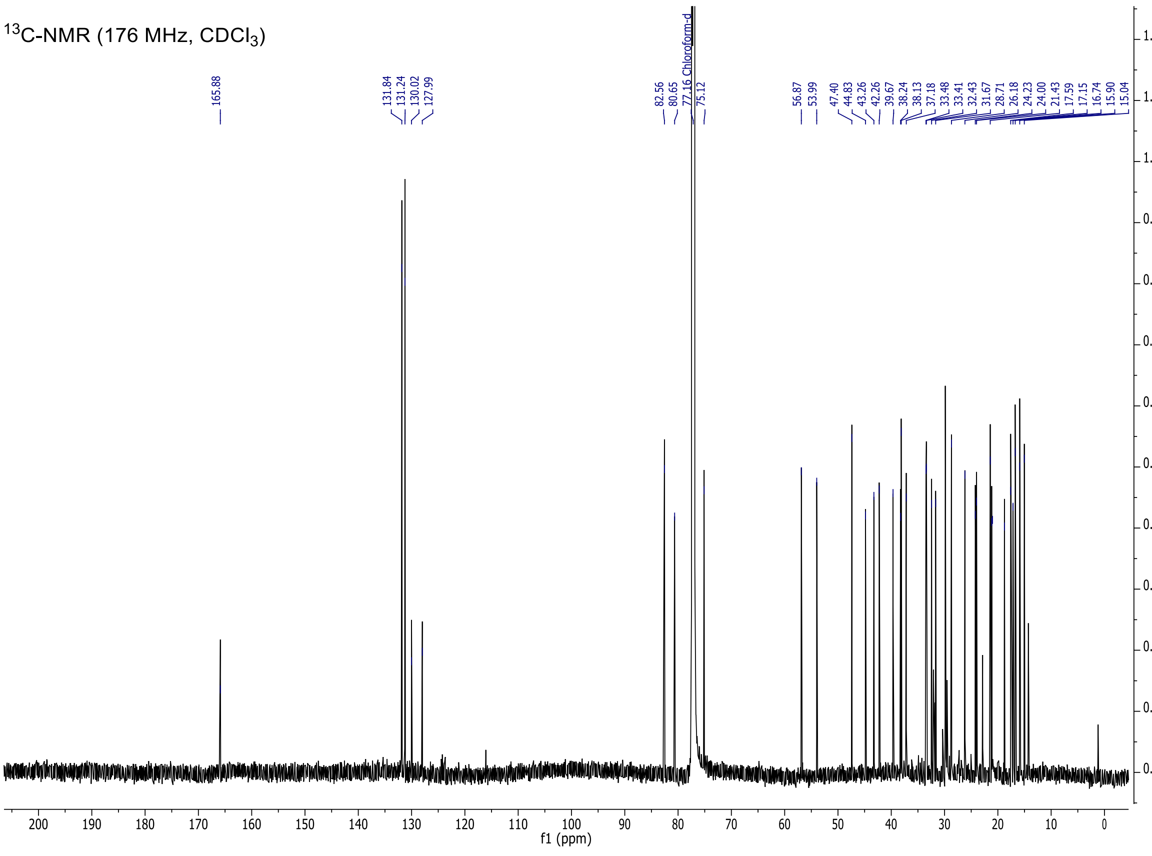
^{13}C-NMR (176 MHz, CDCl$_3$)
165.88
131.84
131.24
130.02
127.99
82.56
80.65
77.16 Chloroform-d
75.12
56.87
53.99
47.40
44.83
43.26
42.26
39.67
38.24
38.13
37.18
33.48
33.41
32.43
31.67
28.71
26.18
24.23
24.00
21.43
17.59
17.15
16.74
15.90
15.04
f1 (ppm)

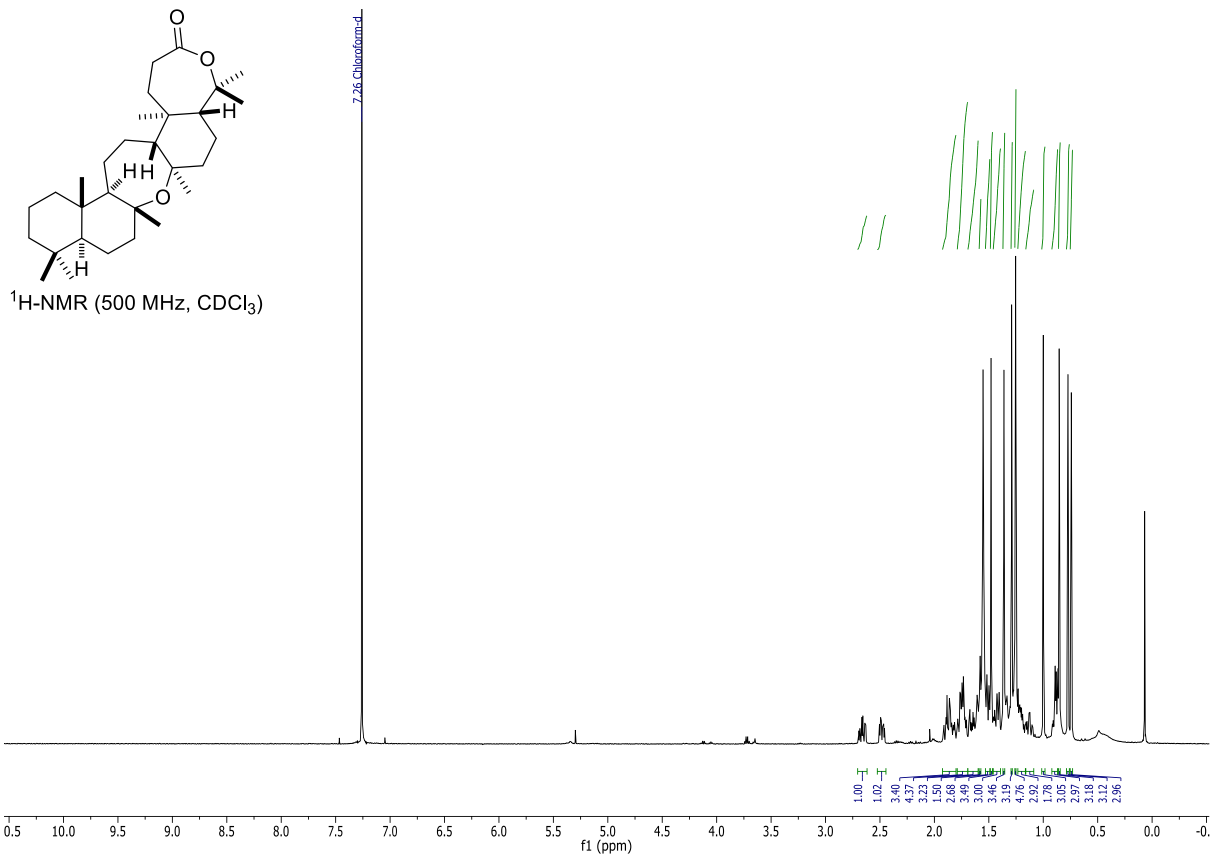

^{1}H-NMR (500 MHz, $CDCl_3$)

^{13}C-NMR (176 MHz, $CDCl_3$)

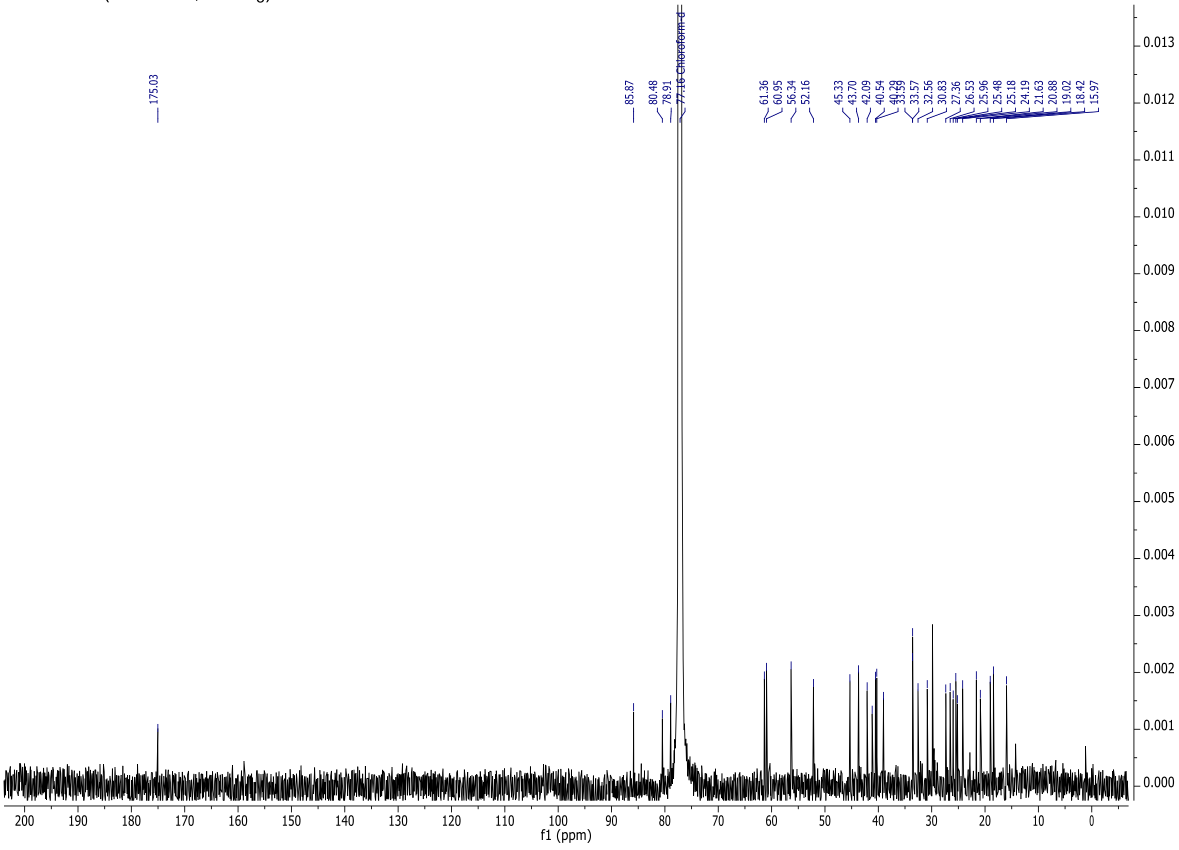

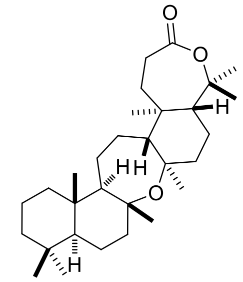

HMQC (700 MHz, $CDCl_3$)

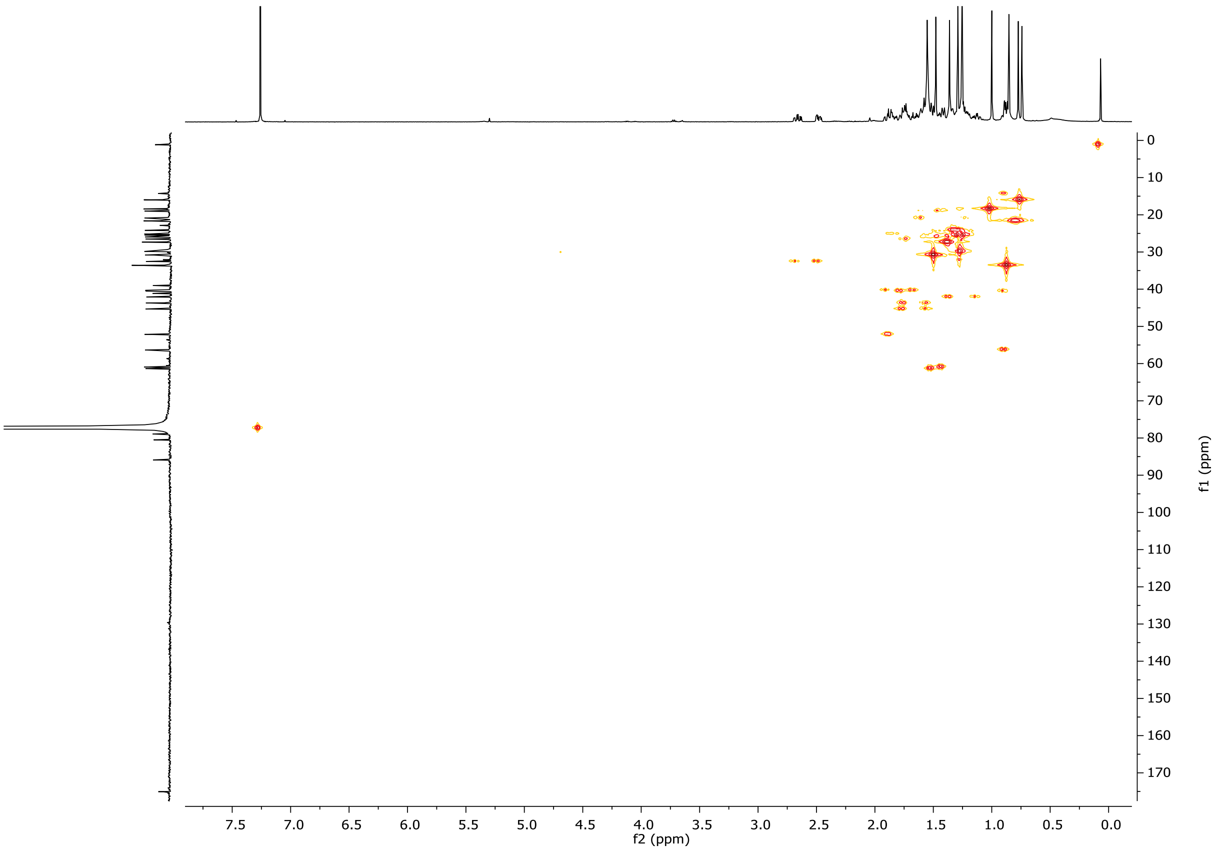

Zoom in HMQC spectrum

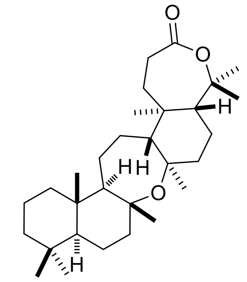

HMQC (700 MHz, $CDCl_3$)

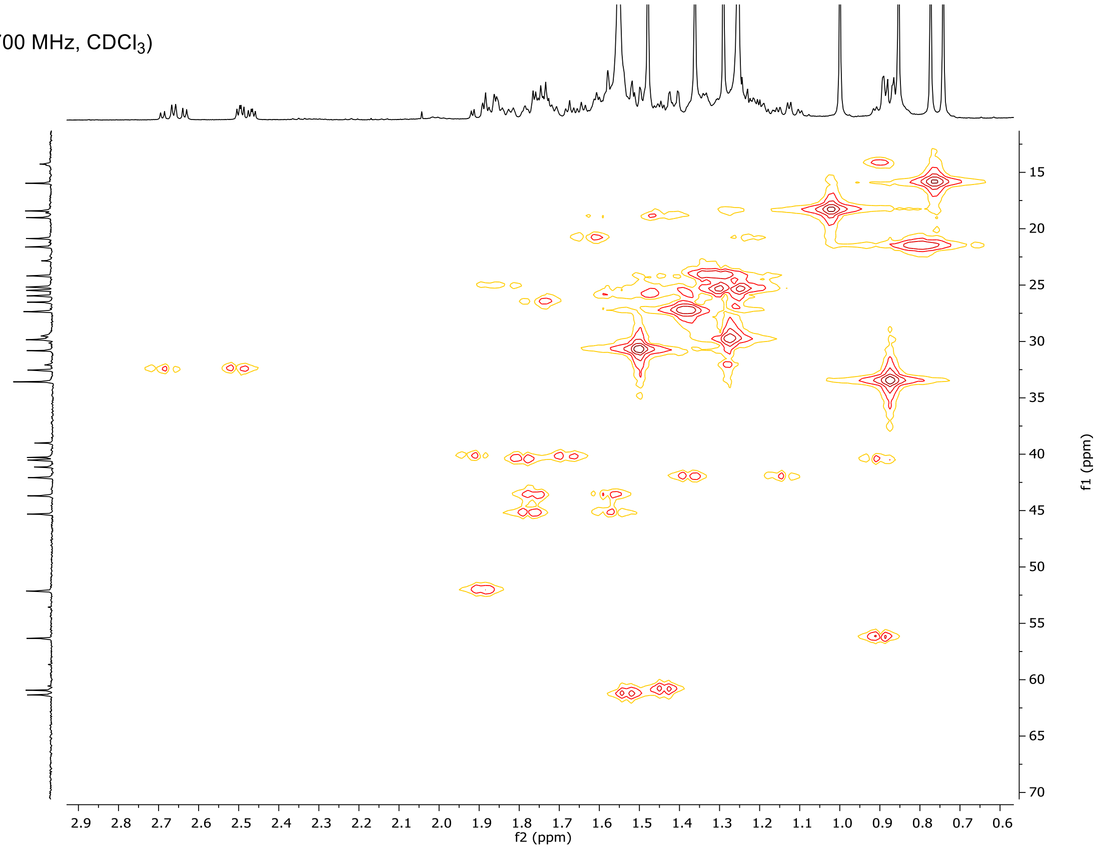

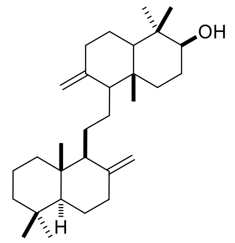

^{1}H-NMR (700 MHz, $CDCl_3$)

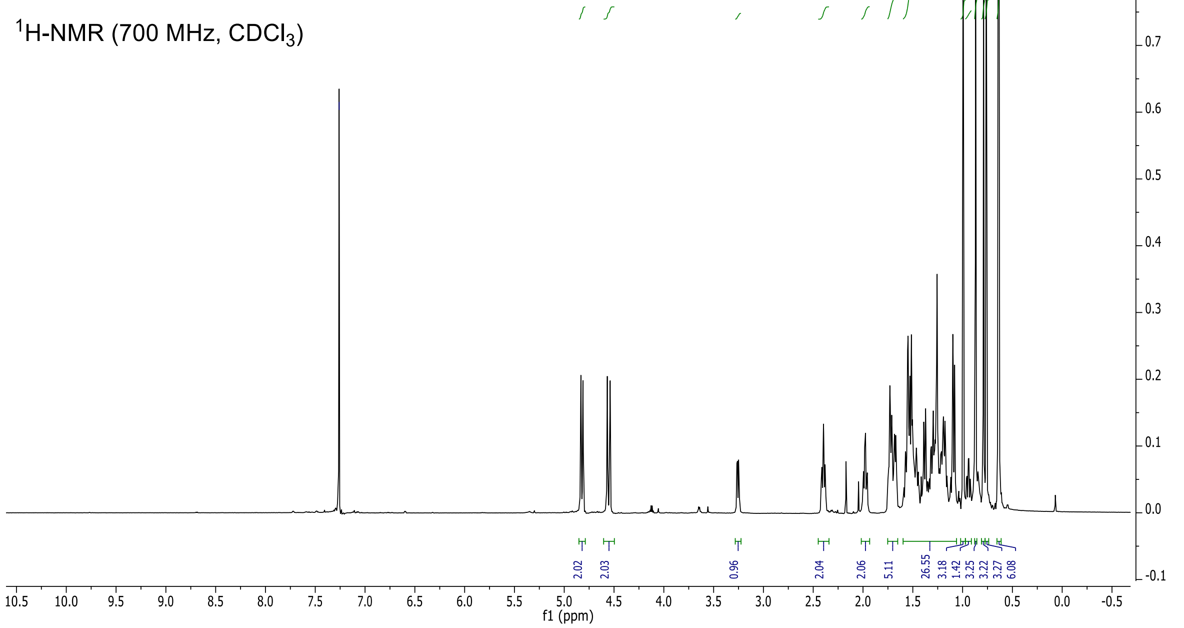

^{13}C-NMR (176 MHz, $CDCl_3$)

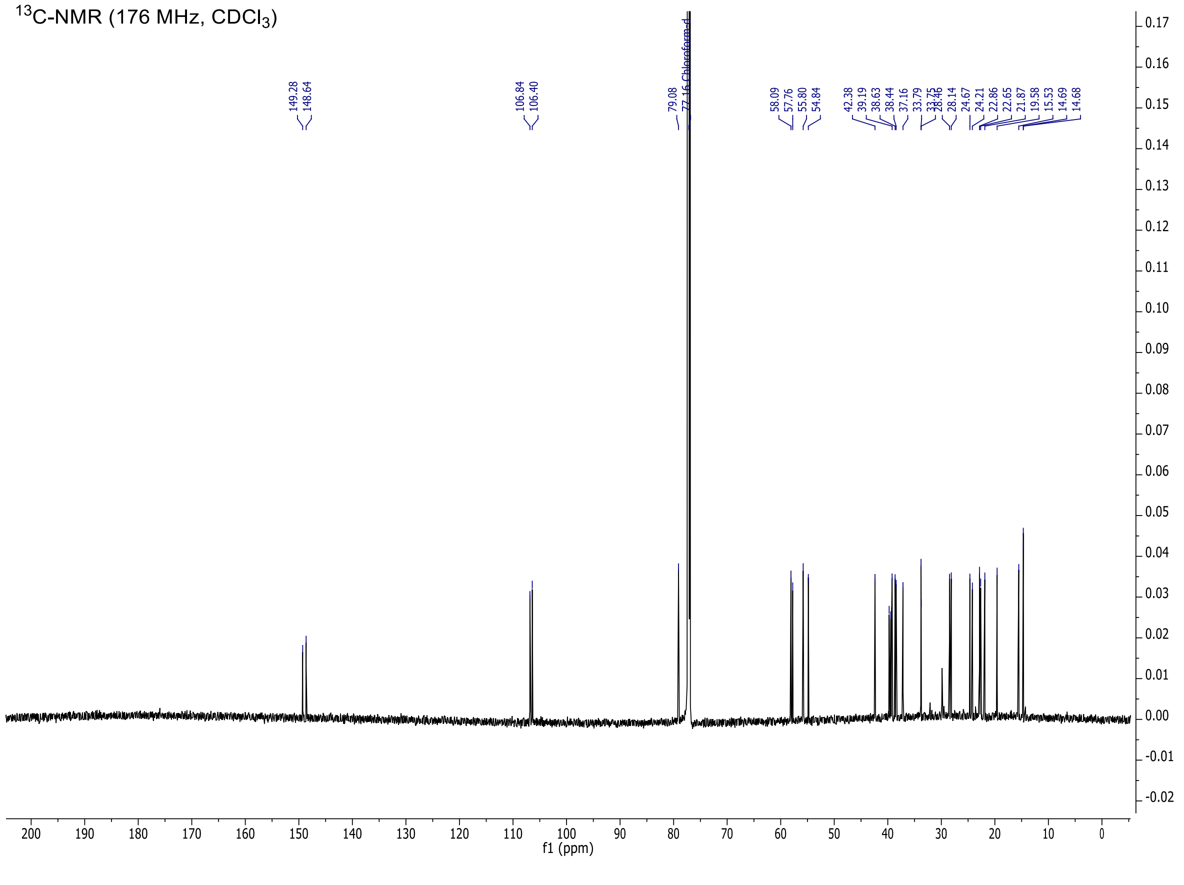

^{1}H-NMR (700 MHz, C_6D_6)

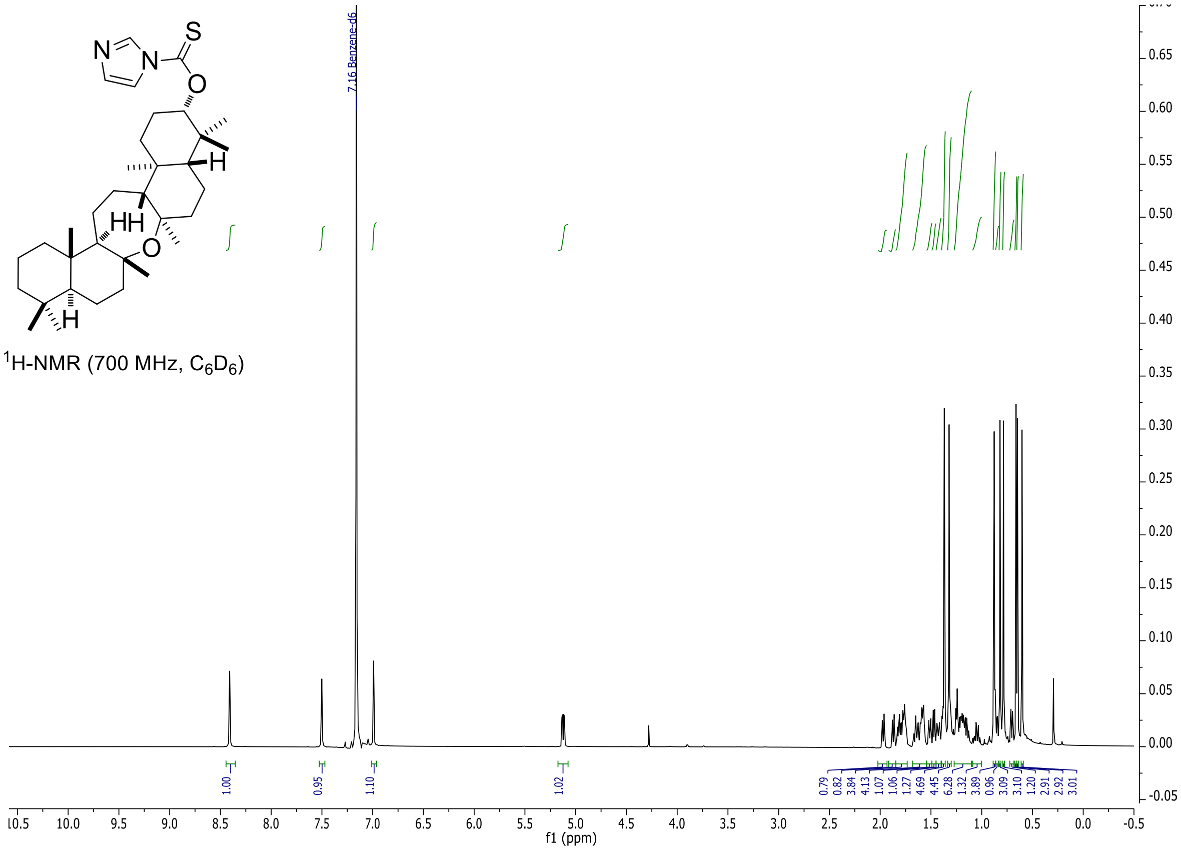

^{13}C-NMR (176 MHz, C_6D_6)

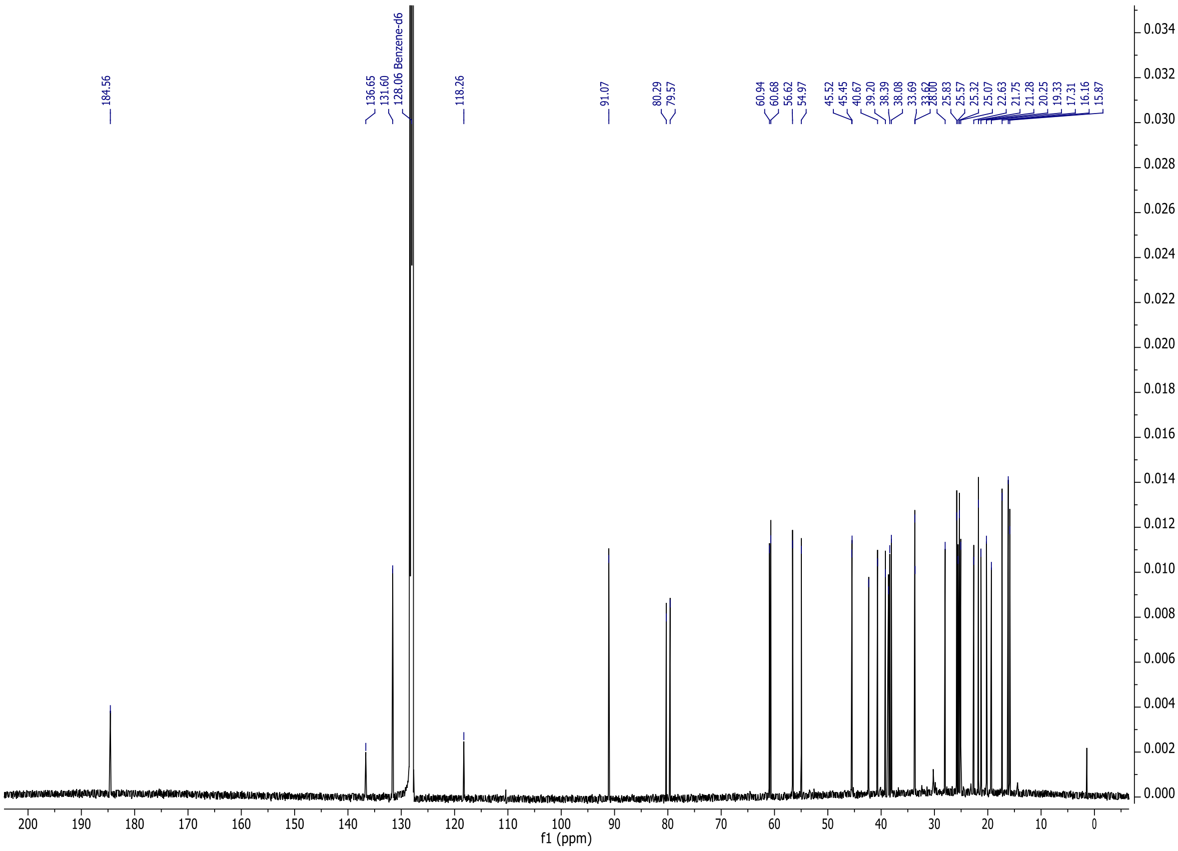

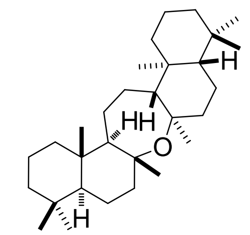

^{1}H-NMR (700 MHz, $CDCl_3$)

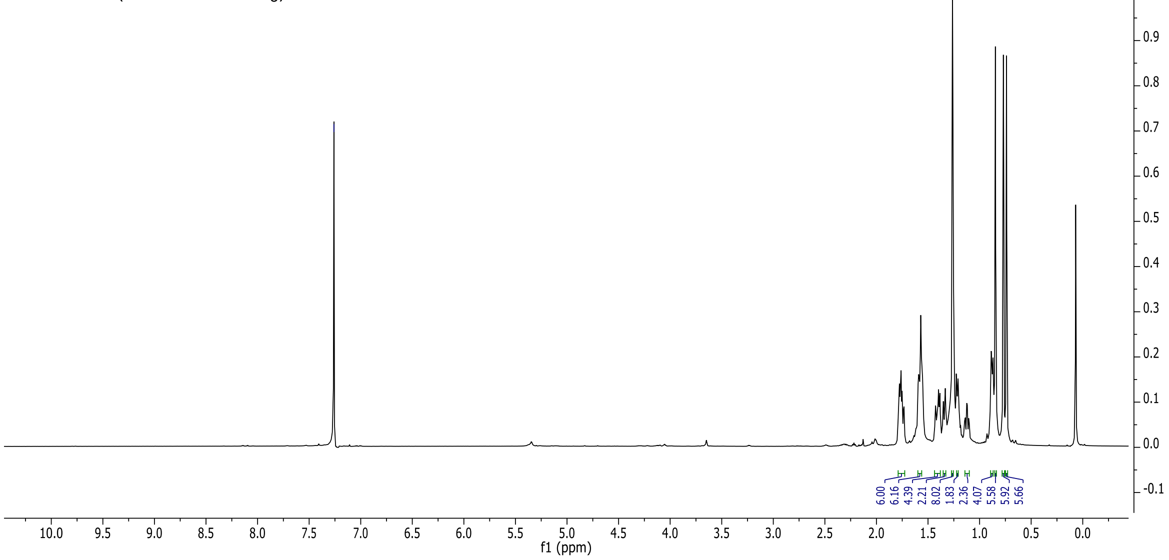

^{13}C-NMR (176 MHz, $CDCl_3$)

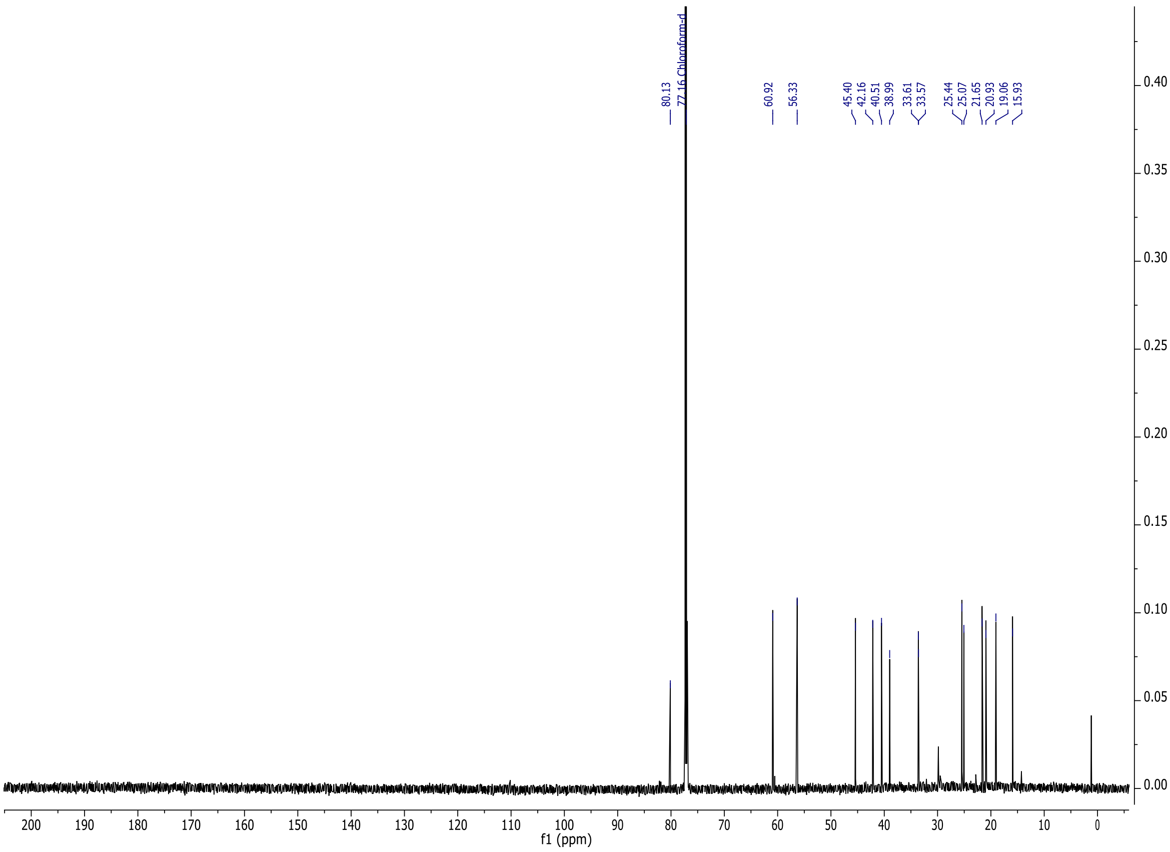

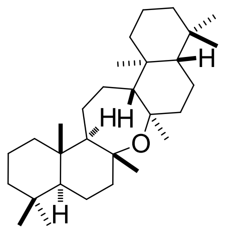

HMQC (700 MHz, $CDCl_3$)

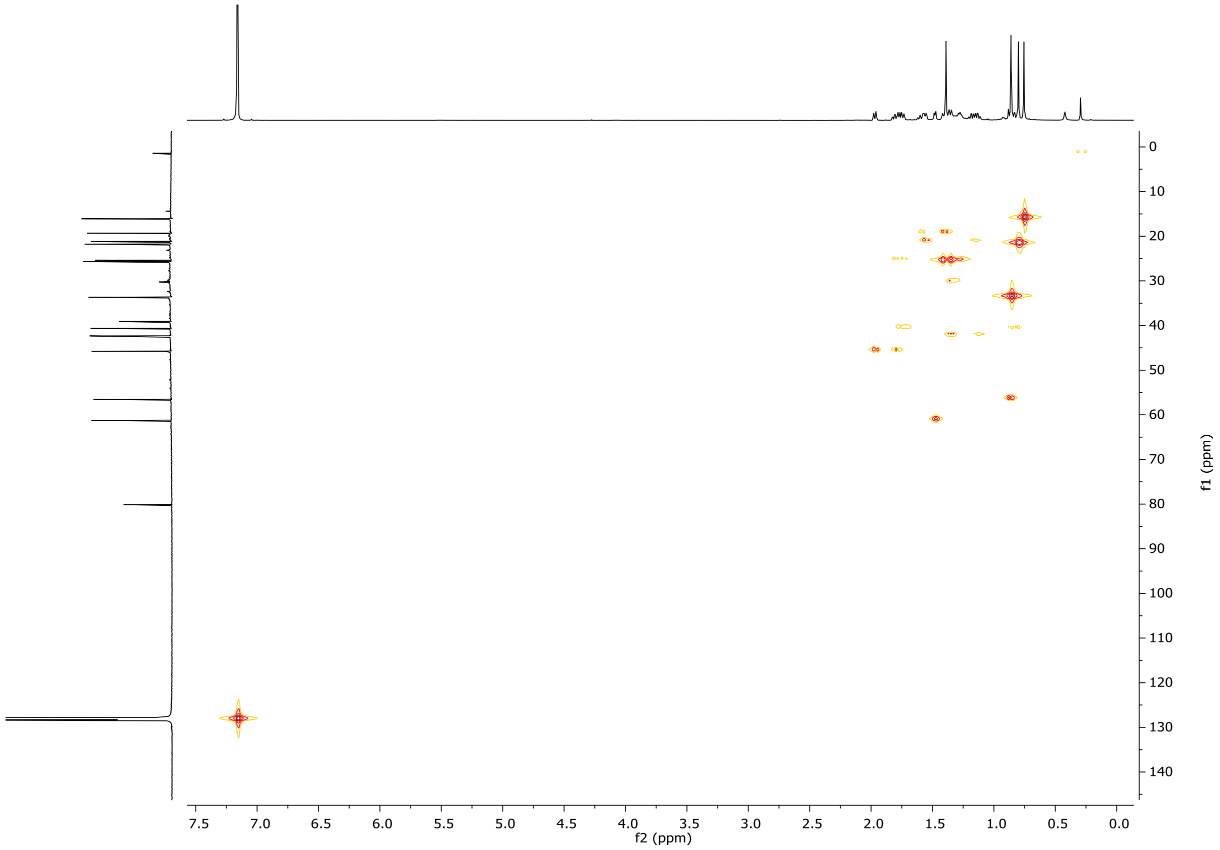

Zoom in HMQC spectrum

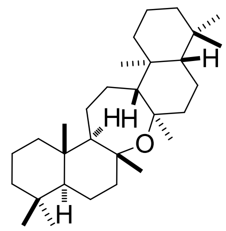

HMQC (700 MHz, $CDCl_3$)

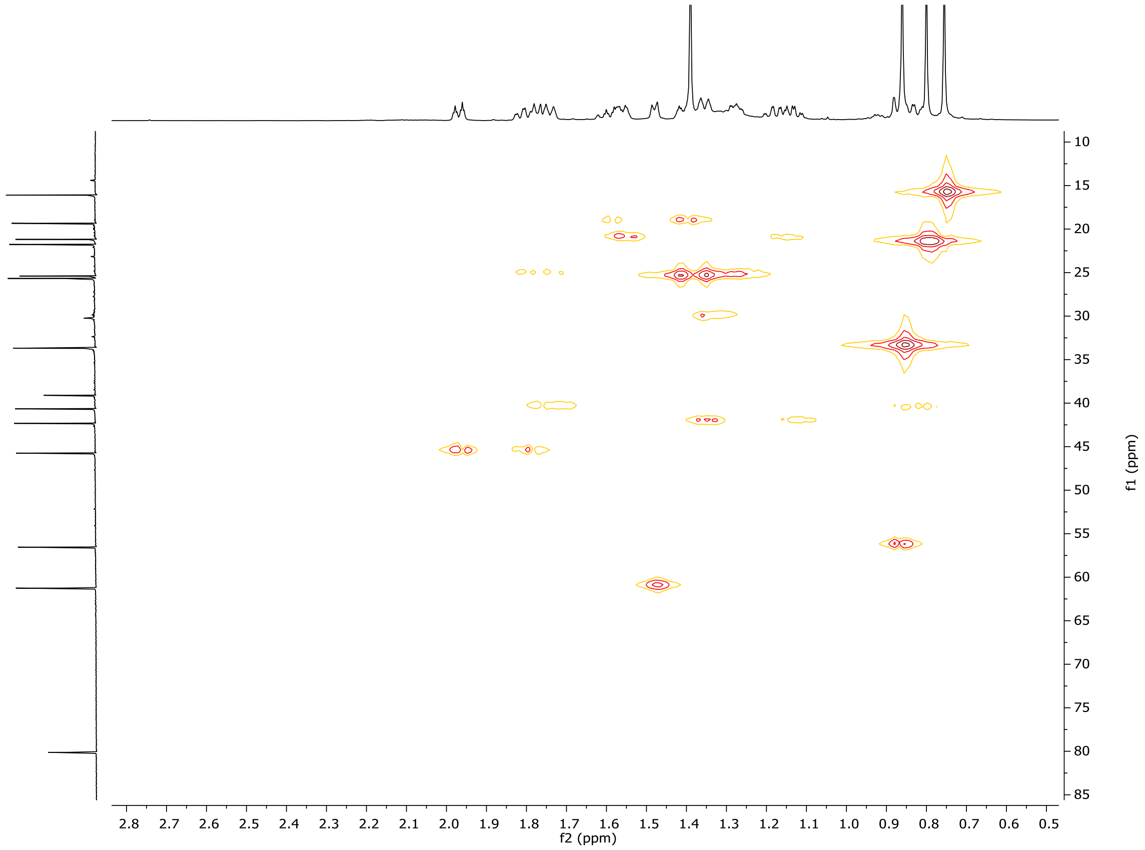

^{1}H-NMR (700 MHz, C_6D_6)

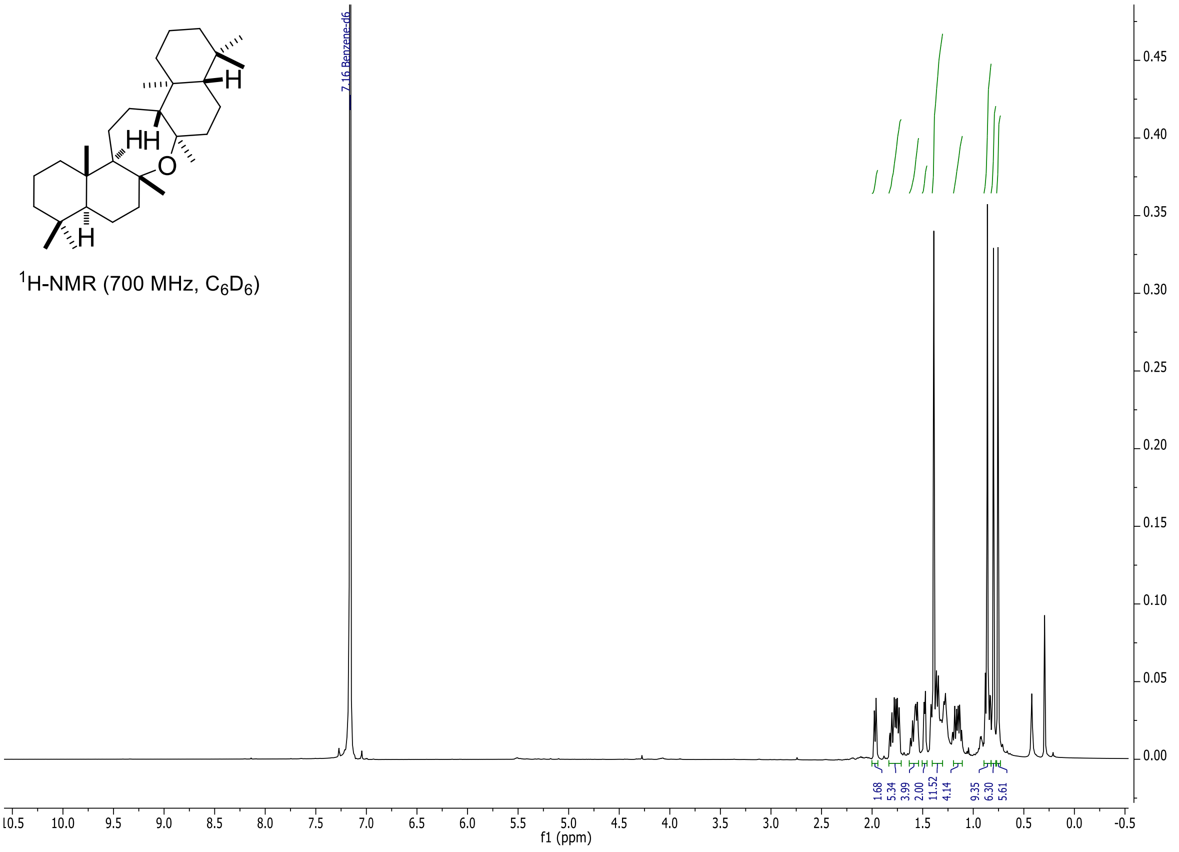

^{13}C-NMR (176 MHz, C_6D_6)

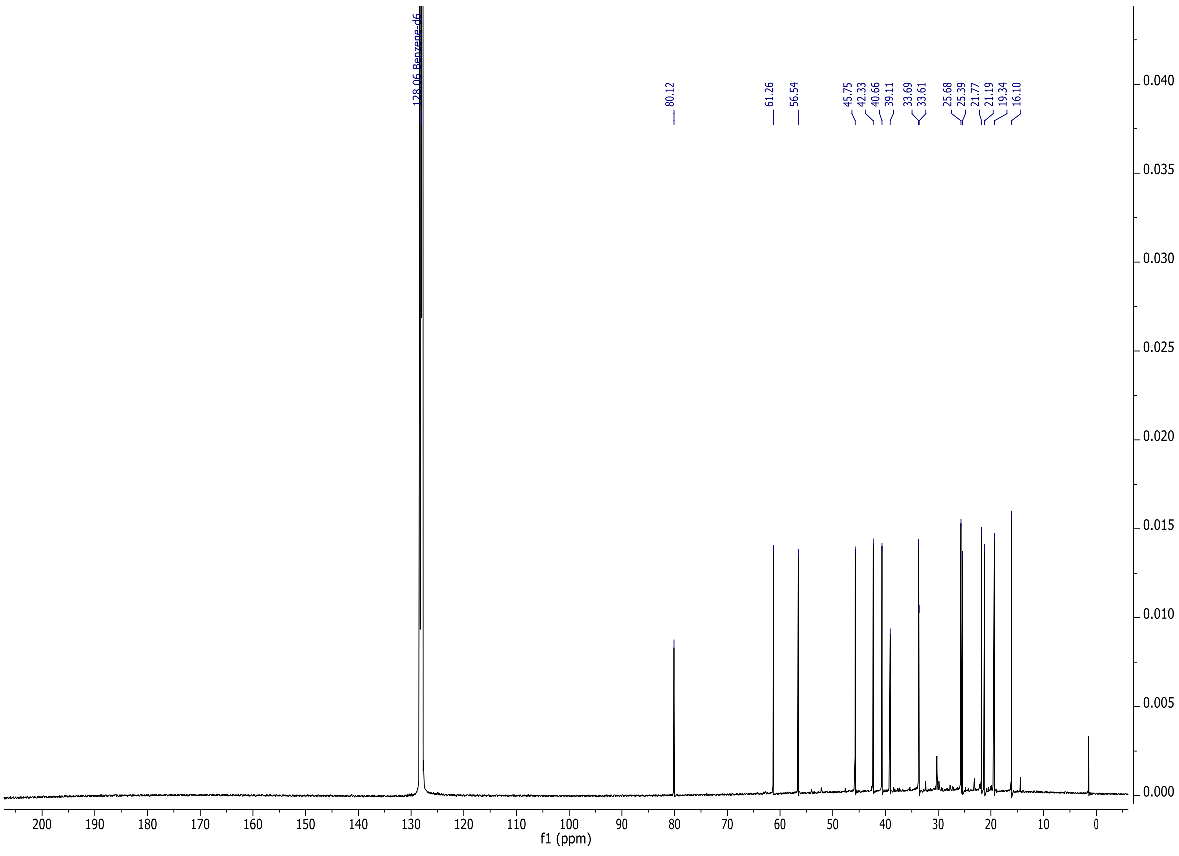

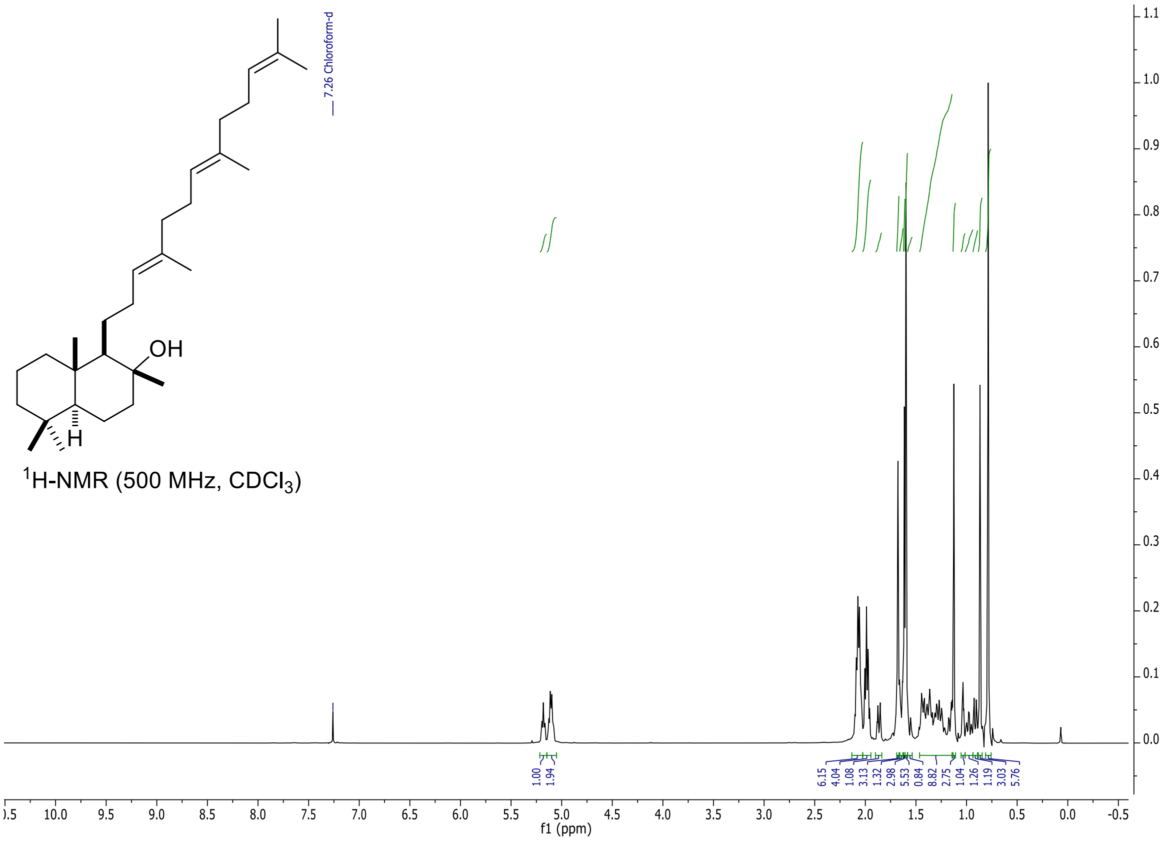

^{1}H-NMR (500 MHz, $CDCl_3$)

^{13}C-NMR (126 MHz, $CDCl_3$)

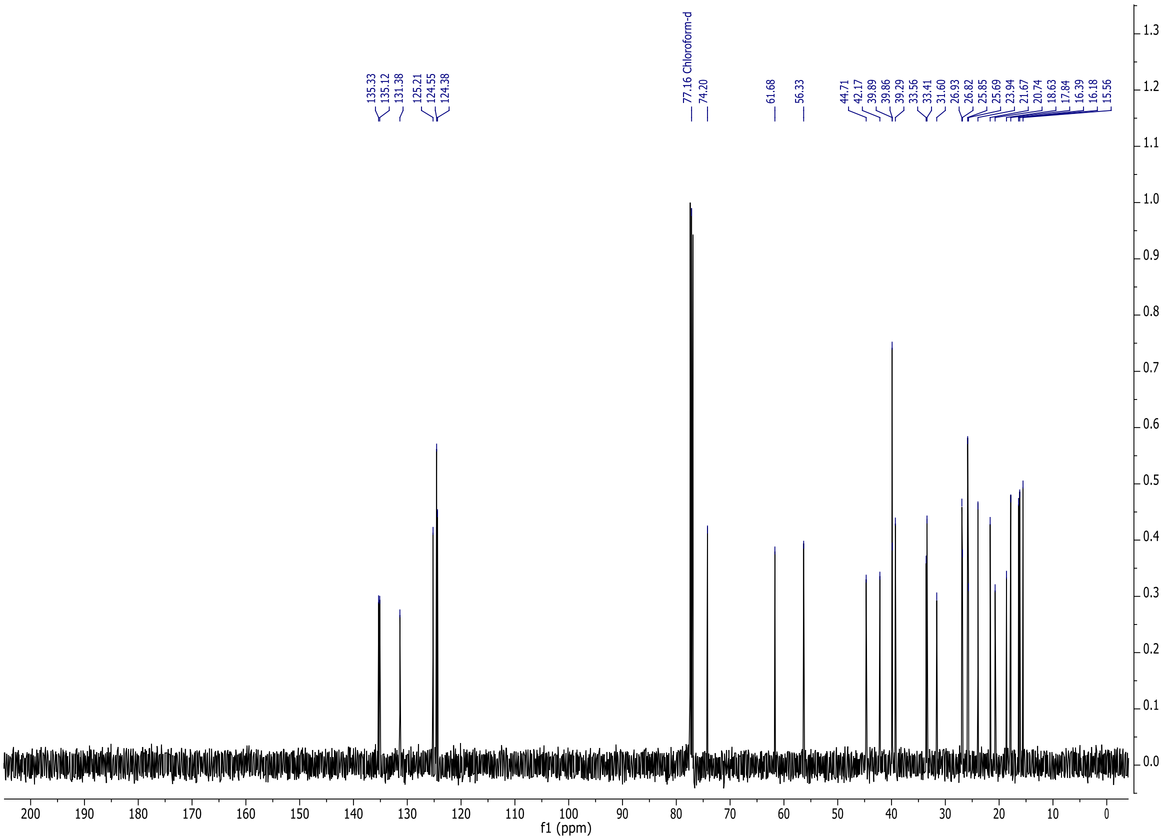

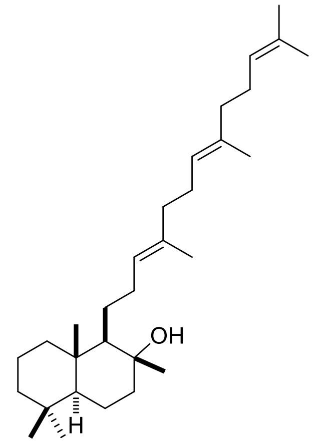

HMQC (500 MHz, $CDCl_3$)

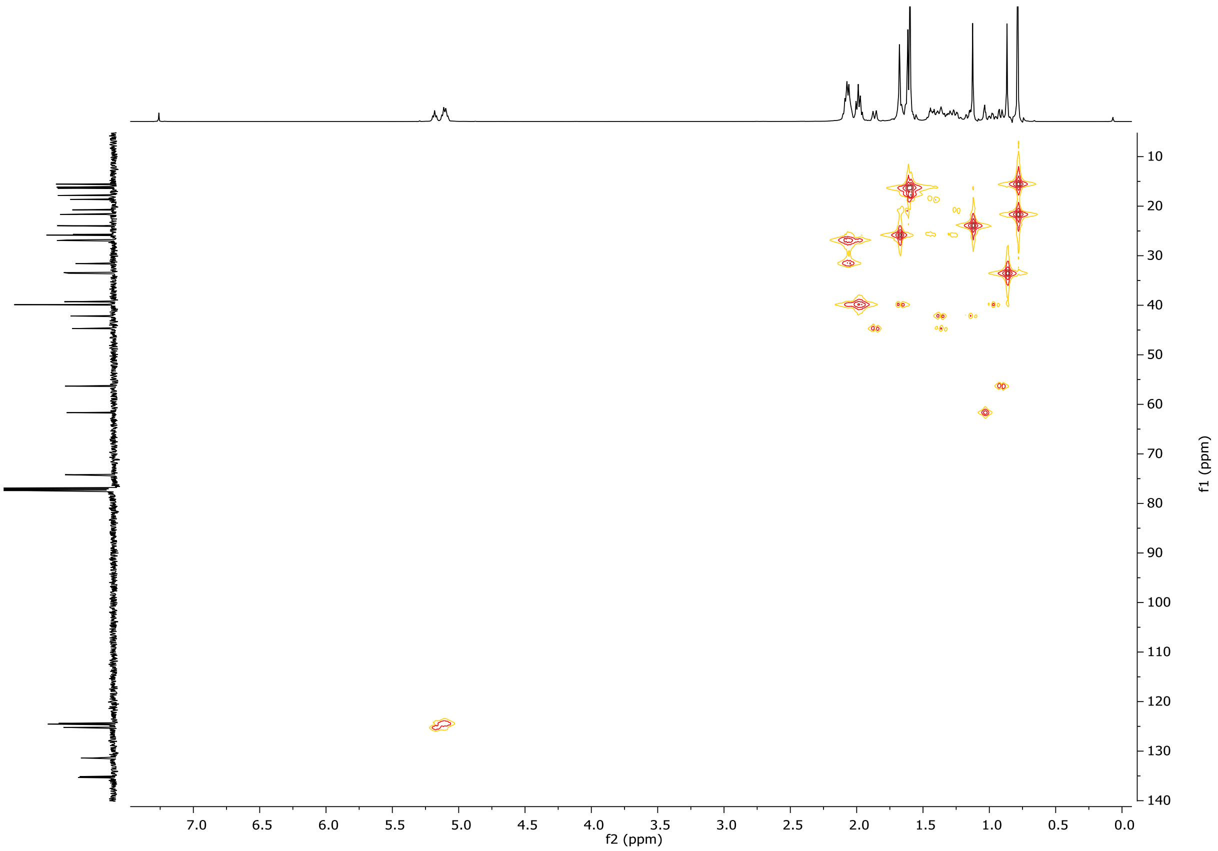

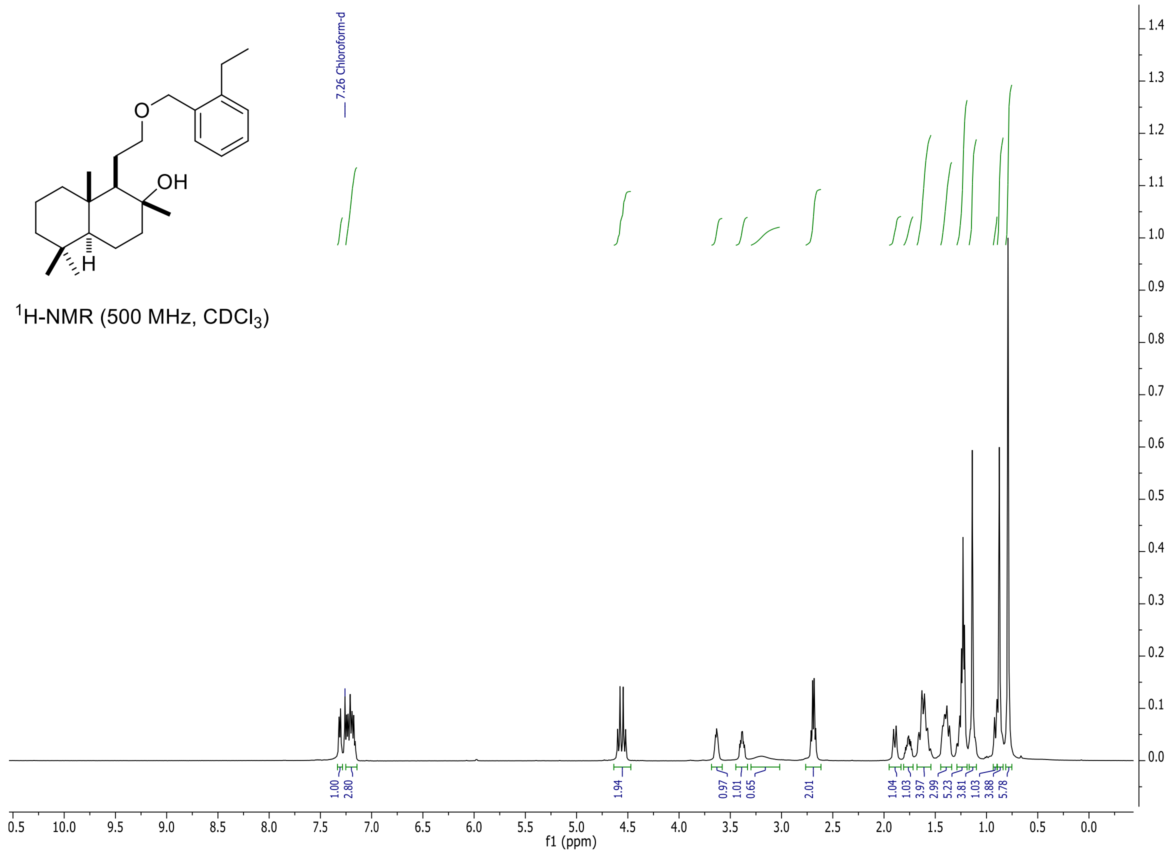

^{1}H-NMR (500 MHz, CDCl$_3$)

^{13}C-NMR (126 MHz, CDCl$_3$)

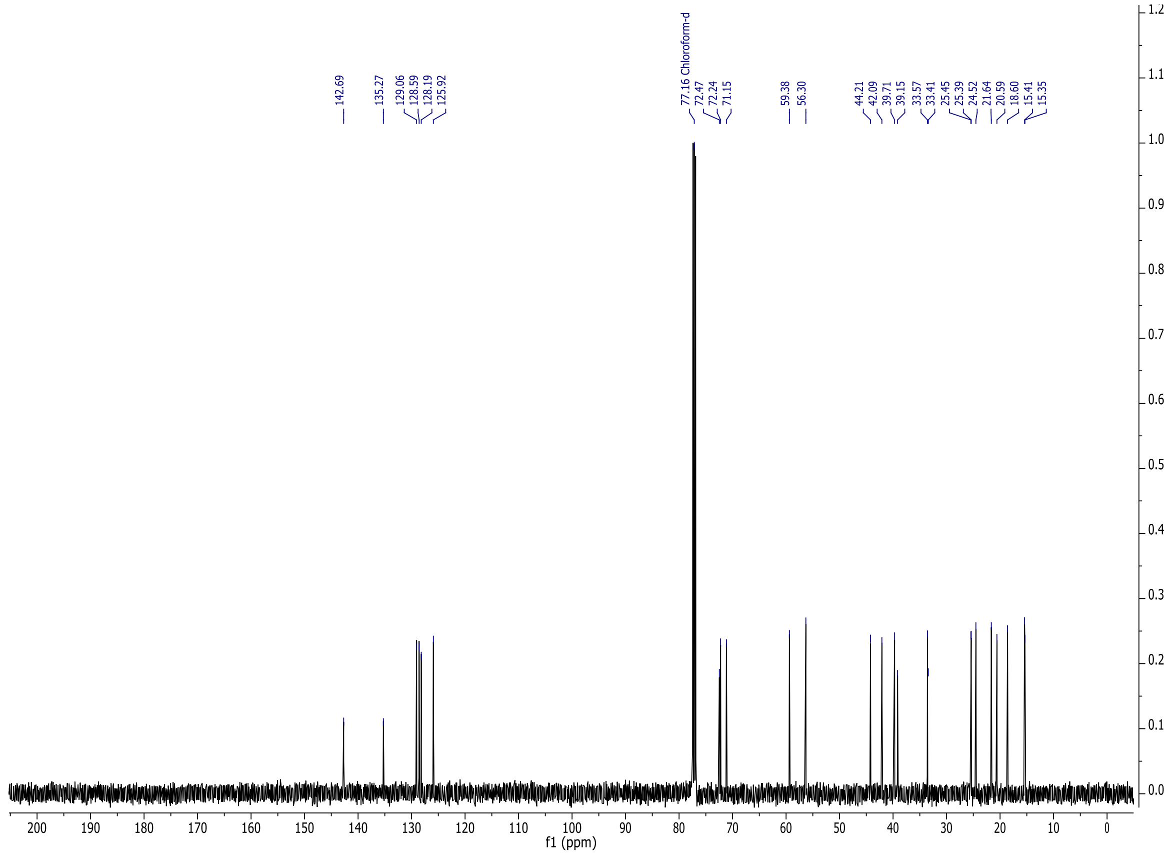

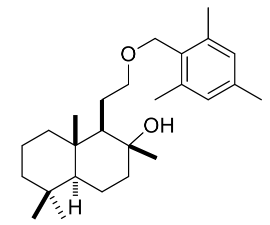

^{1}H-NMR (500 MHz, $CDCl_3$)

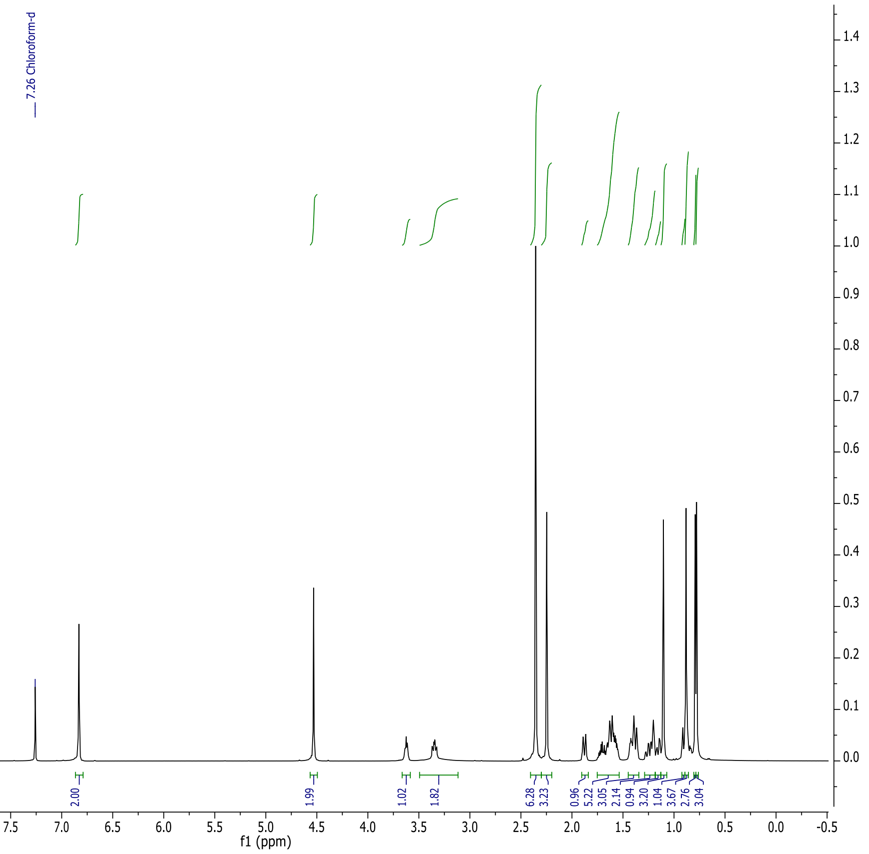

^{13}C-NMR (126 MHz, $CDCl_3$)

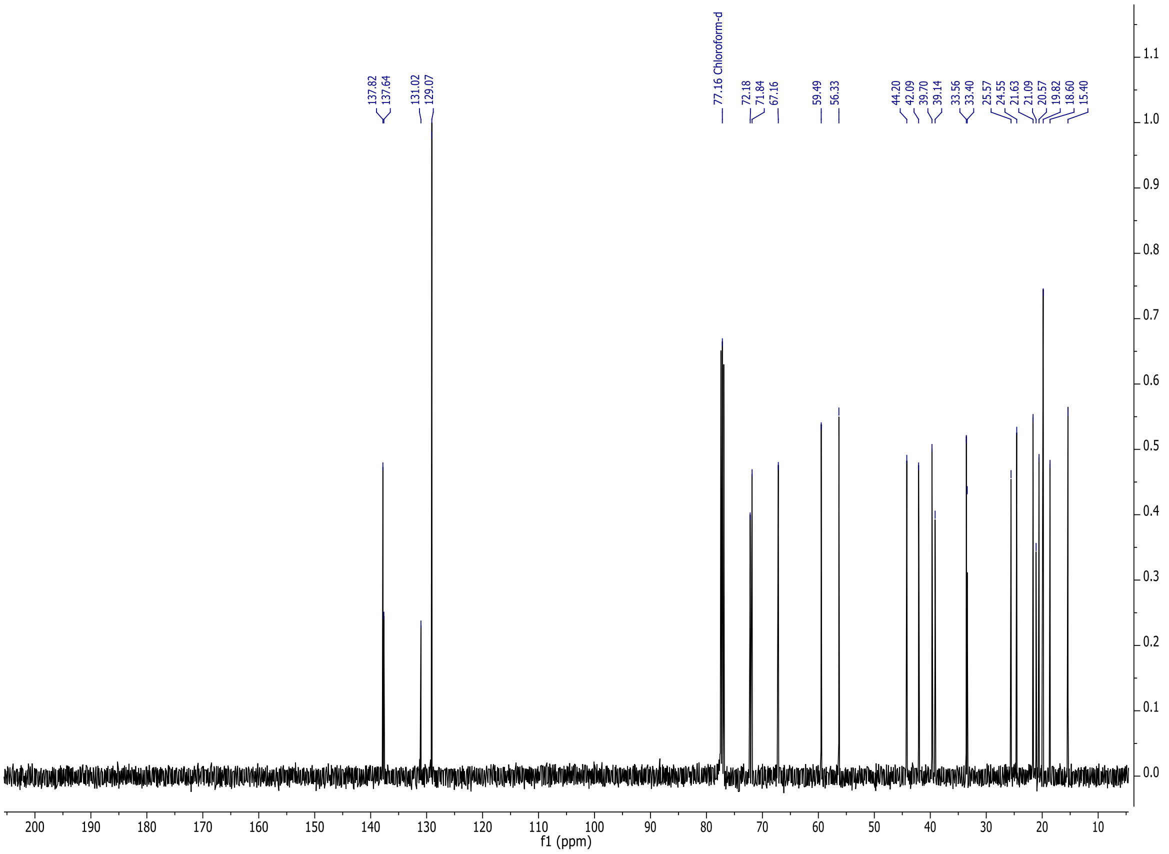

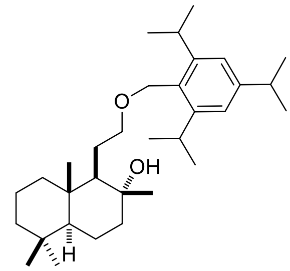

^{1}H-NMR (500 MHz, $CDCl_3$)

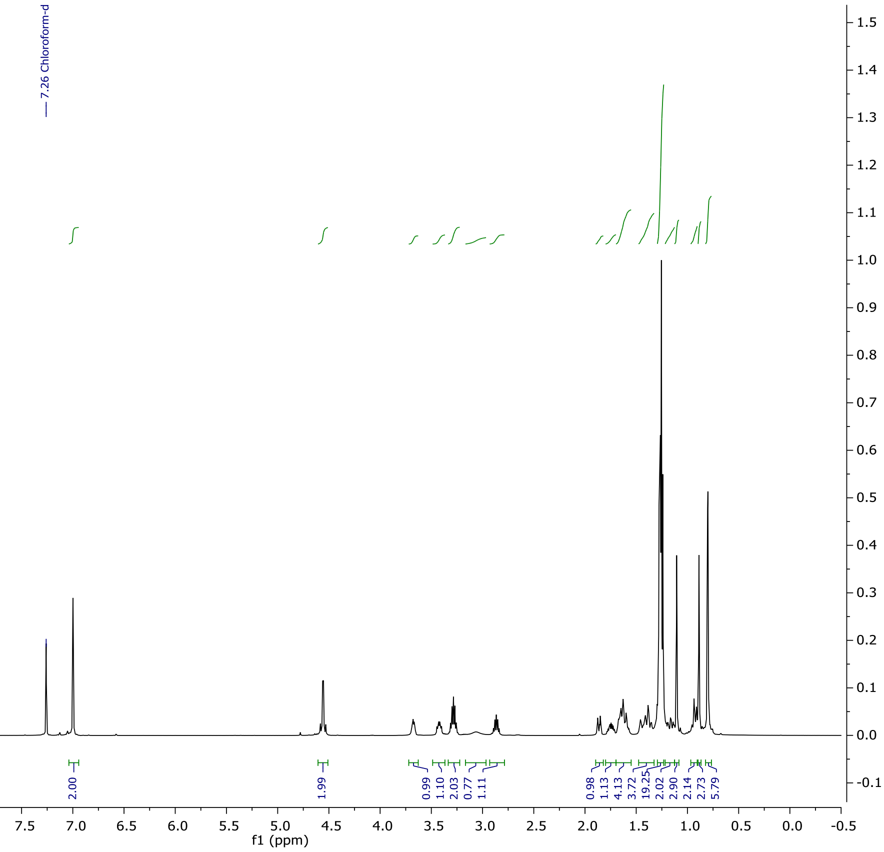

^{13}C-NMR (126 MHz, $CDCl_3$)

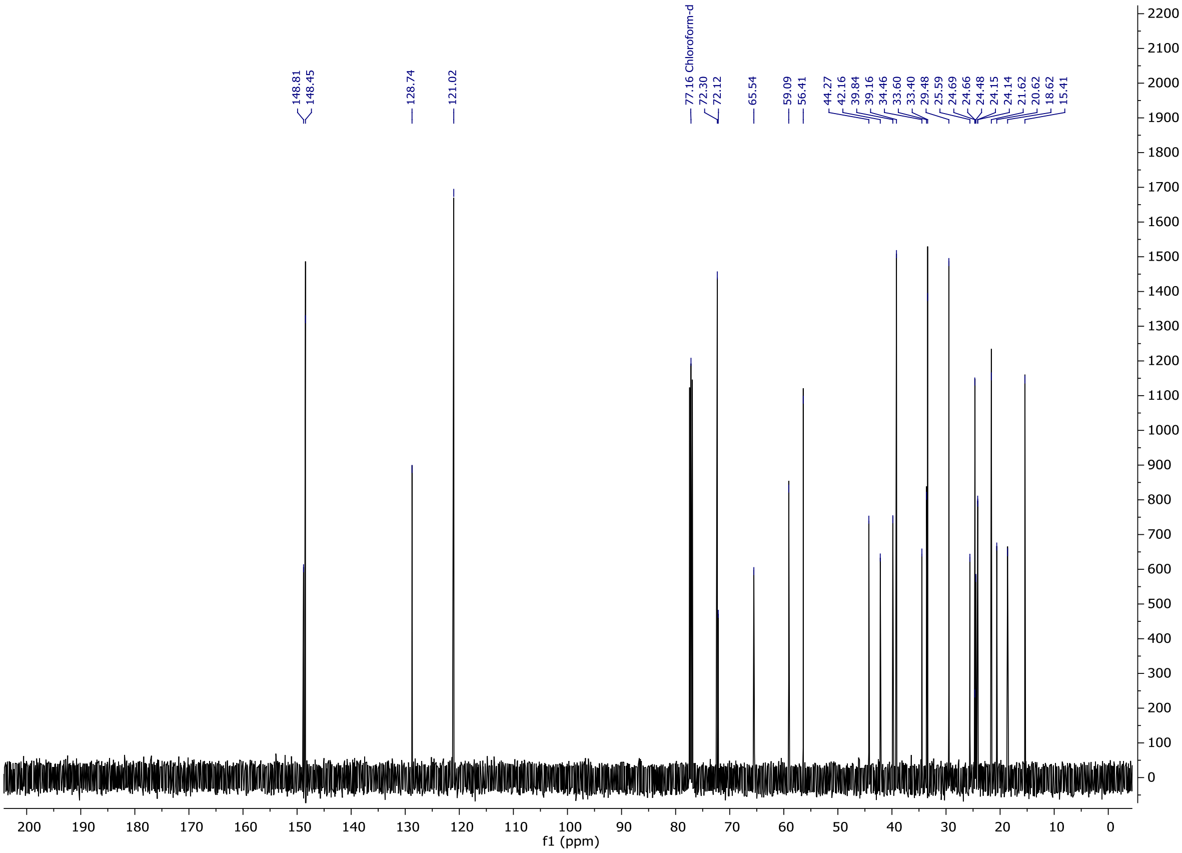

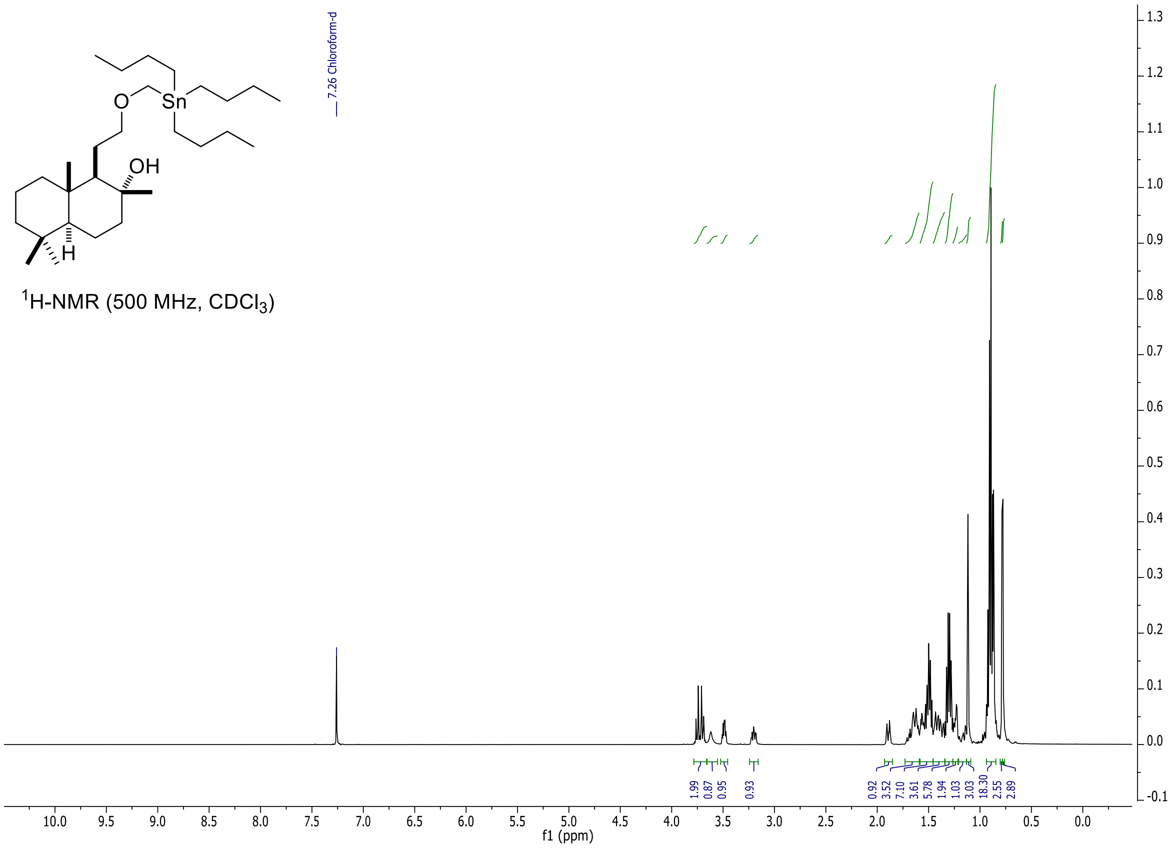

^{1}H-NMR (500 MHz, $CDCl_3$)

^{13}C-NMR (176 MHz, $CDCl_3$)

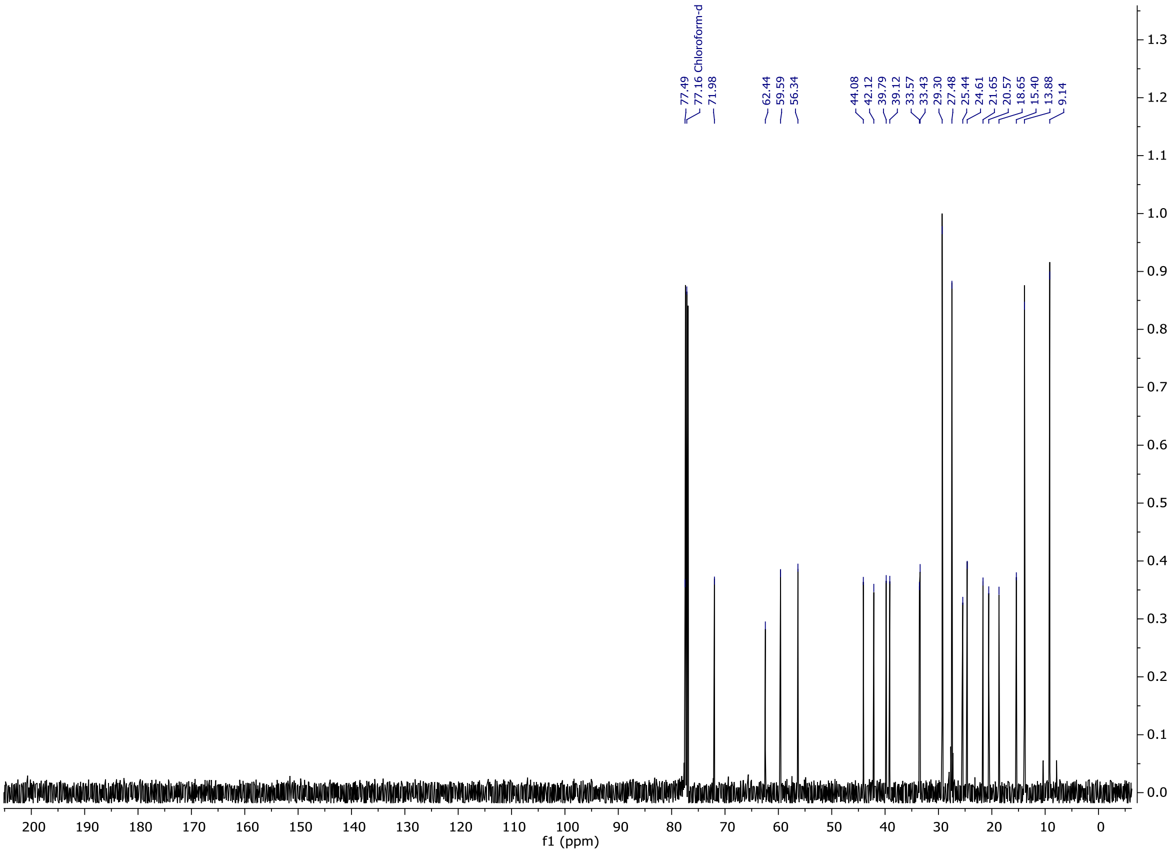

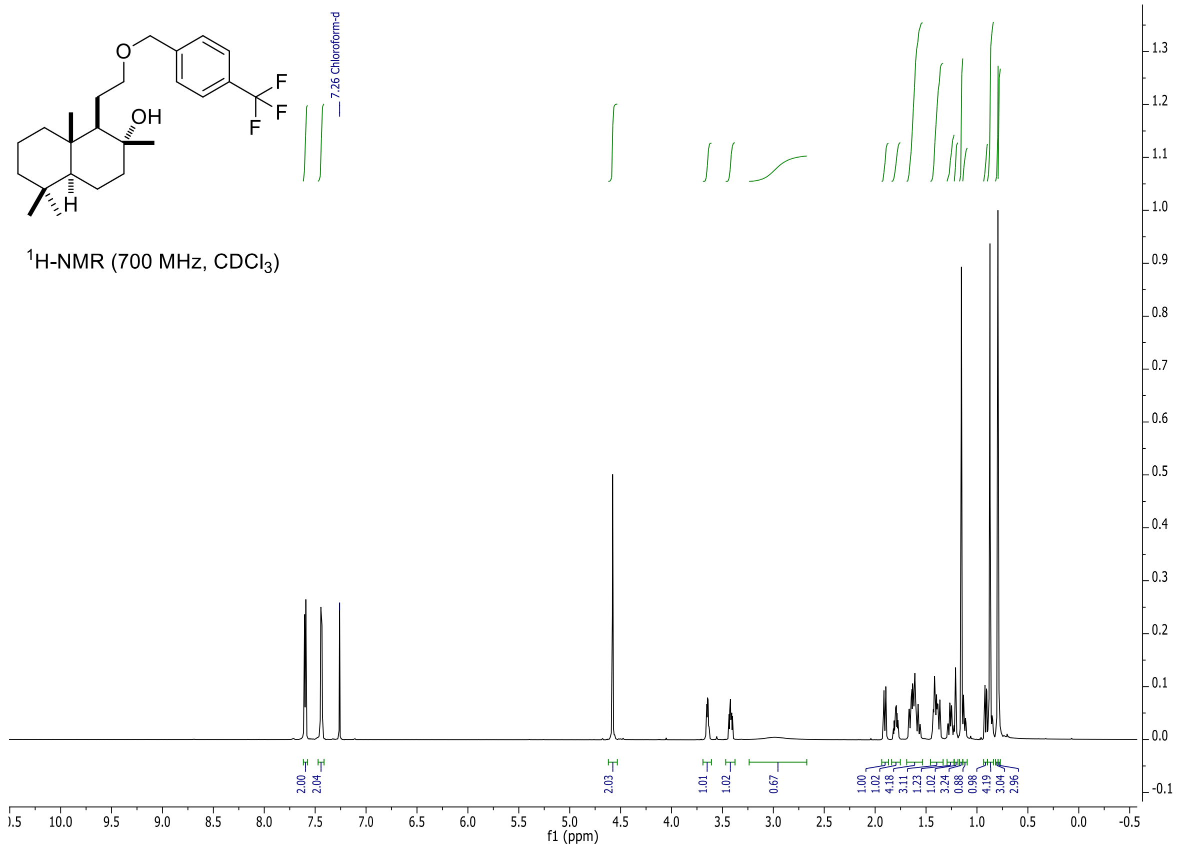

^{1}H-NMR (700 MHz, $CDCl_3$)

^{13}C-NMR (176 MHz, $CDCl_3$)

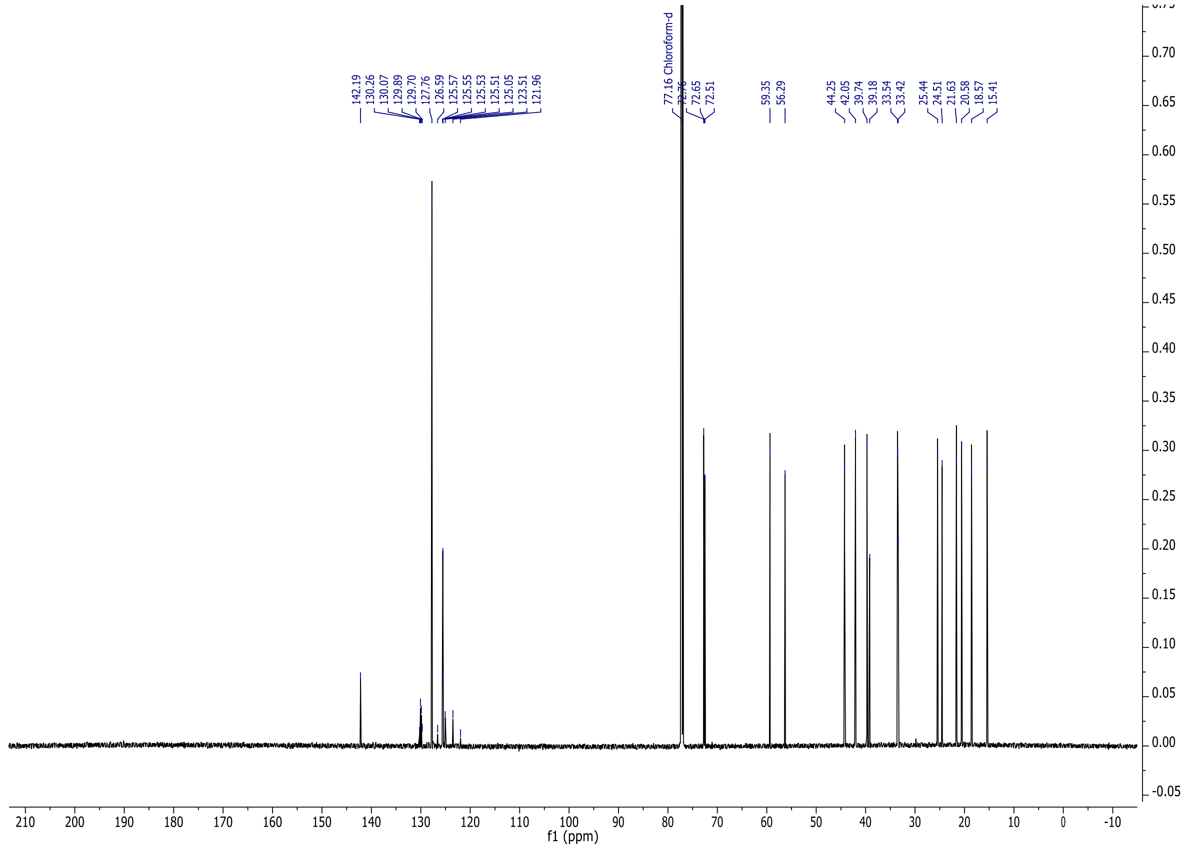

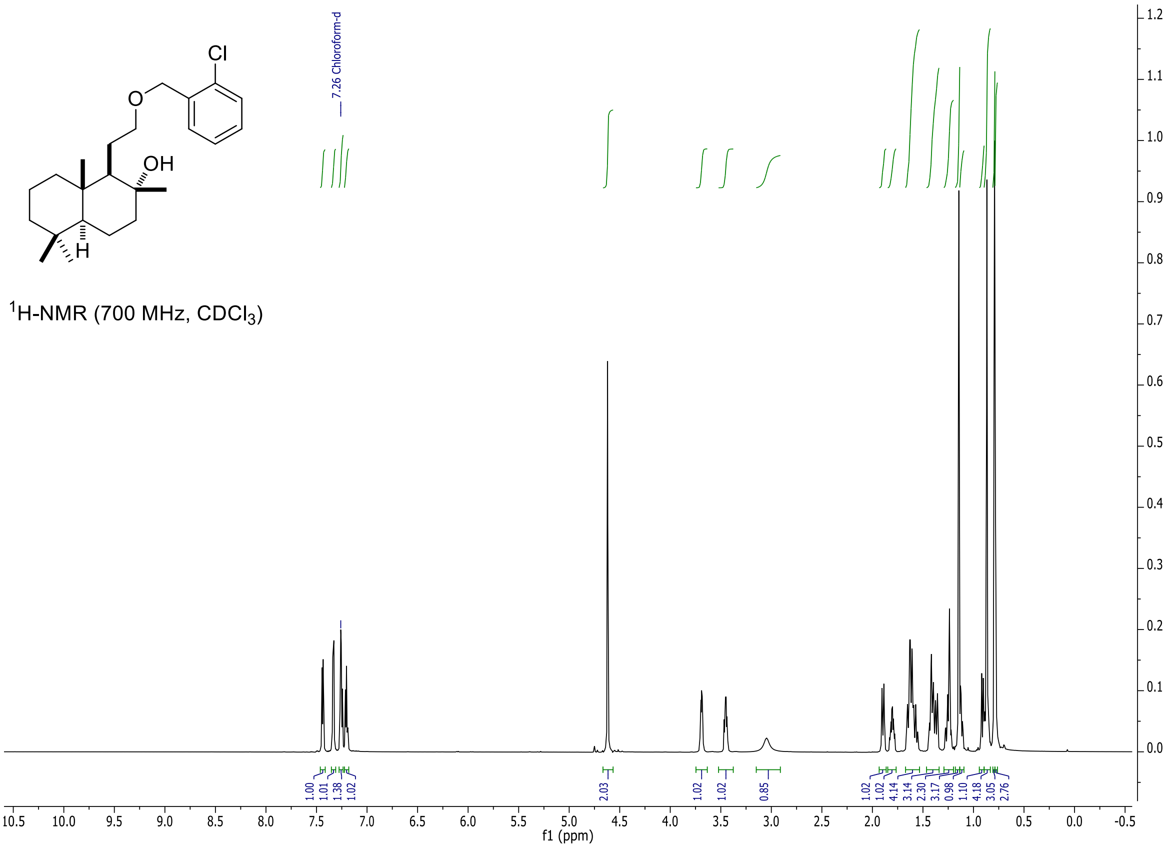

^{1}H-NMR (700 MHz, $CDCl_3$)

^{13}C-NMR (176 MHz, $CDCl_3$)

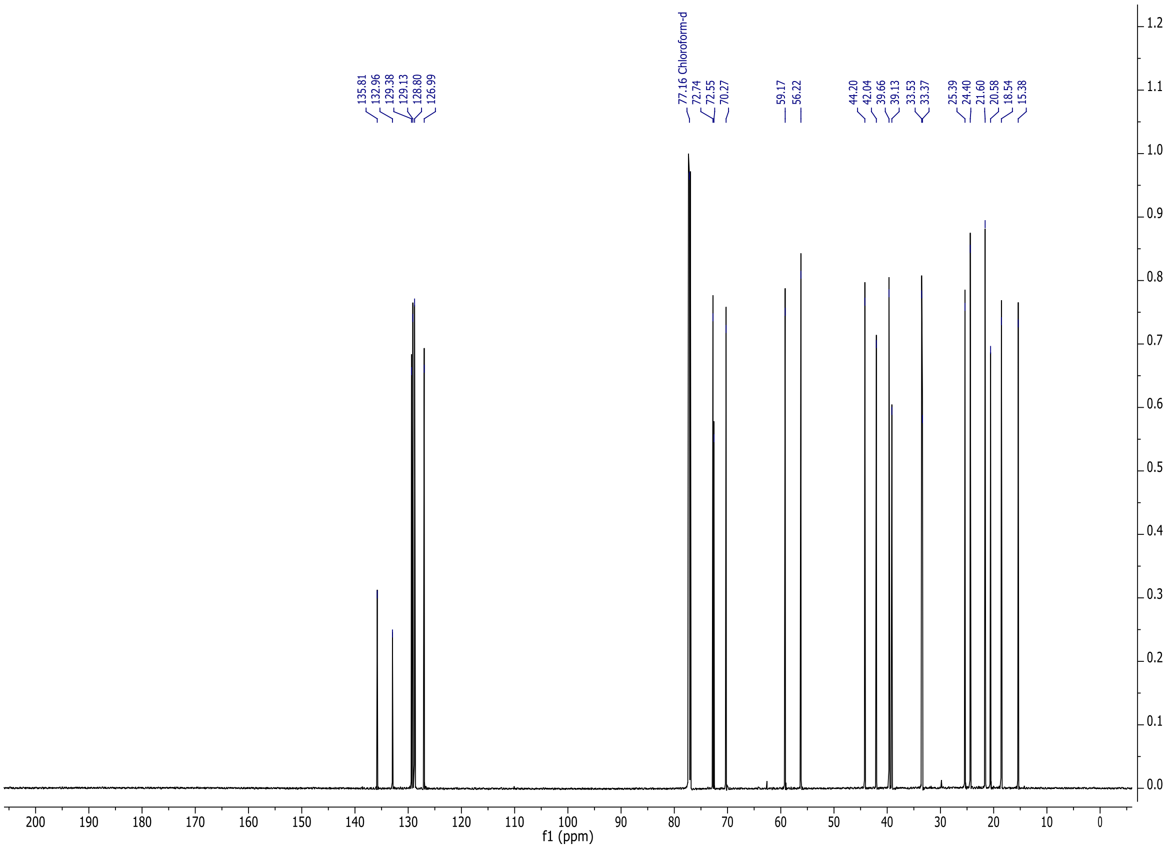

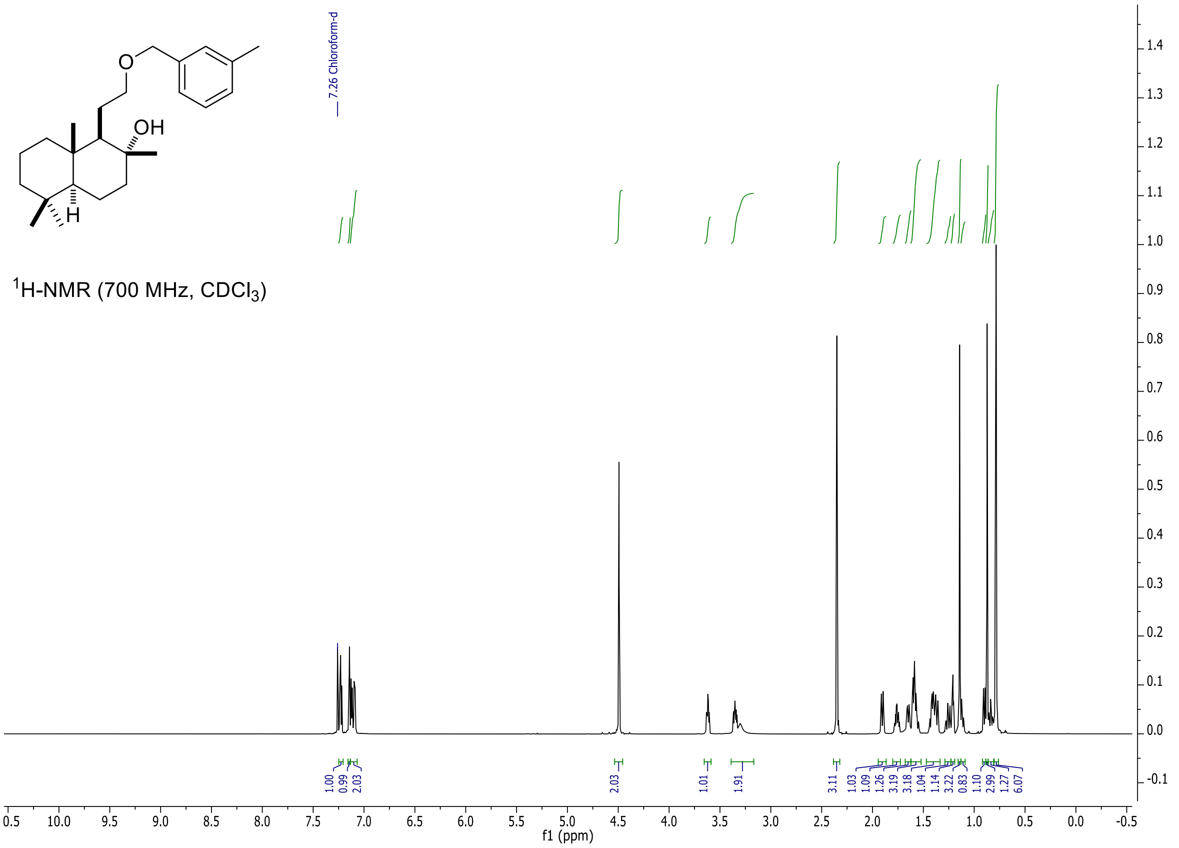

^{1}H-NMR (700 MHz, $CDCl_3$)

^{13}C-NMR (176 MHz, $CDCl_3$)

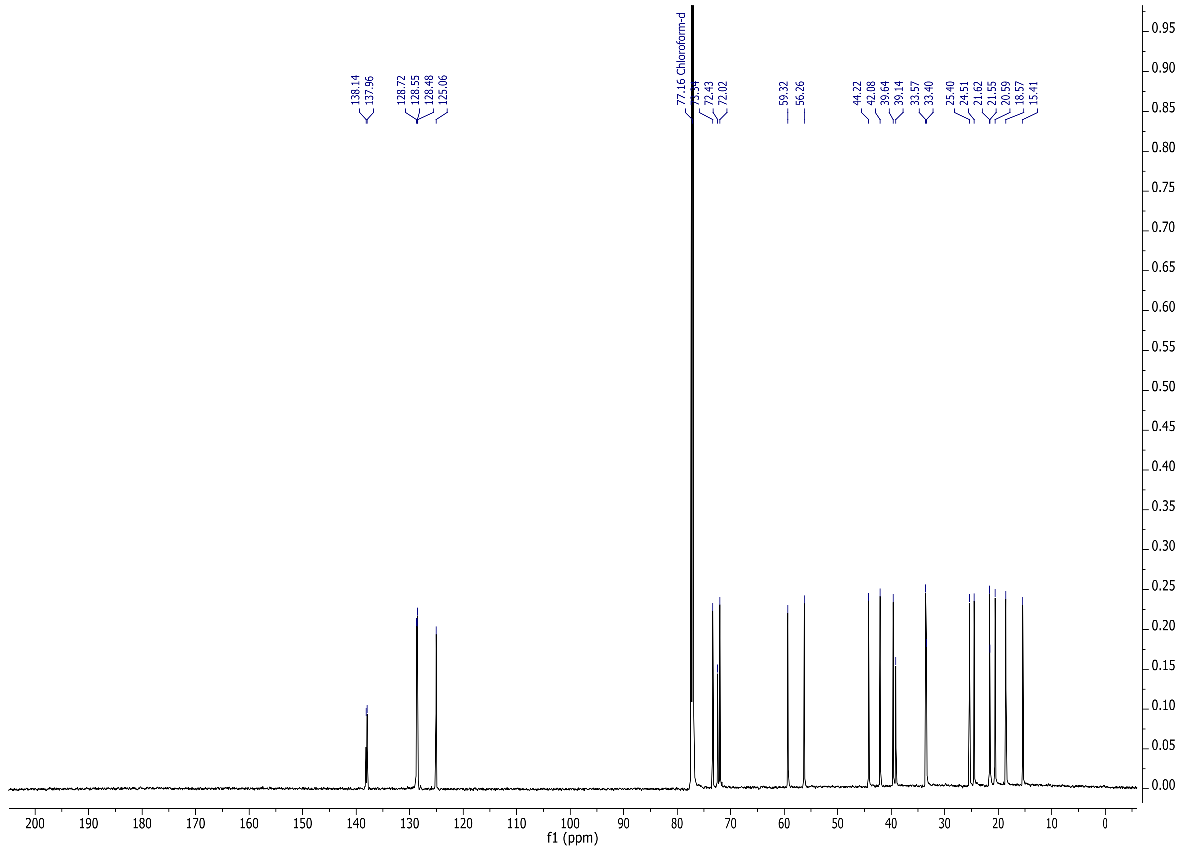

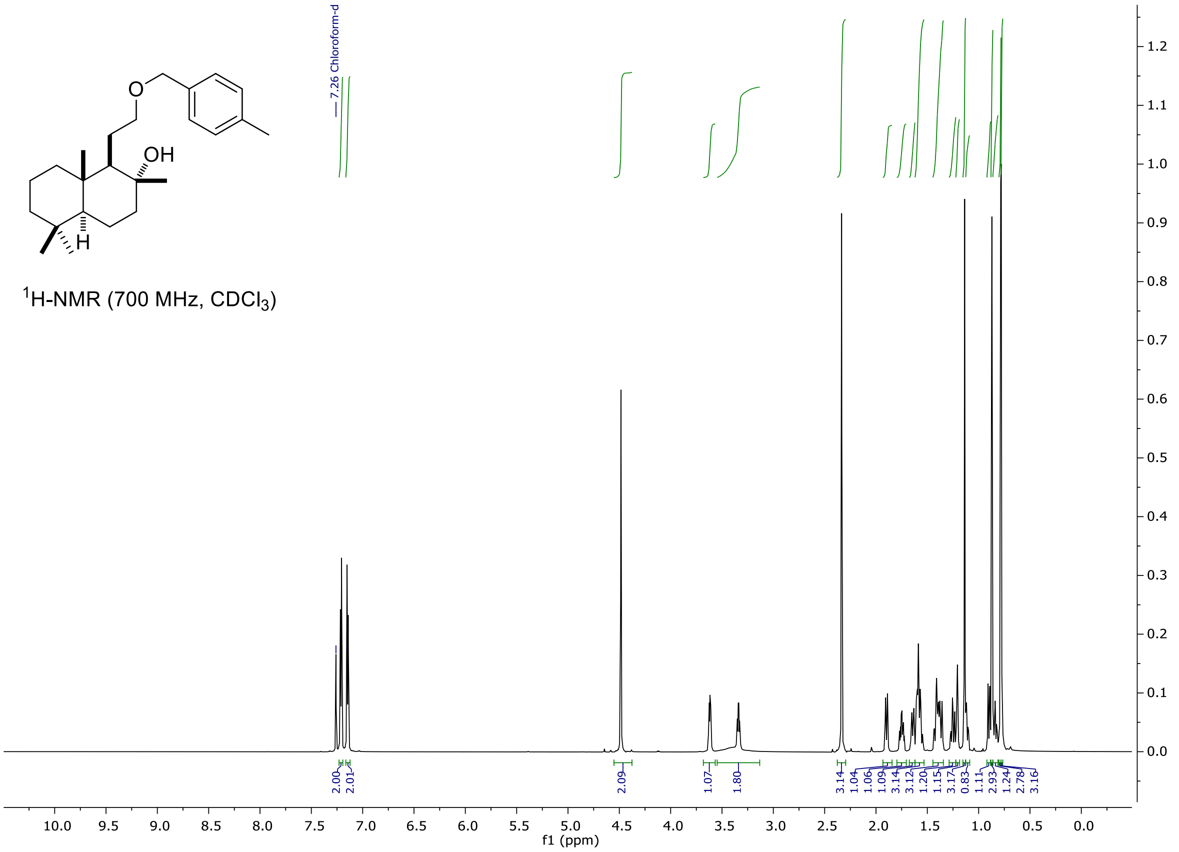

13C-NMR (176 MHz, CDCl3)

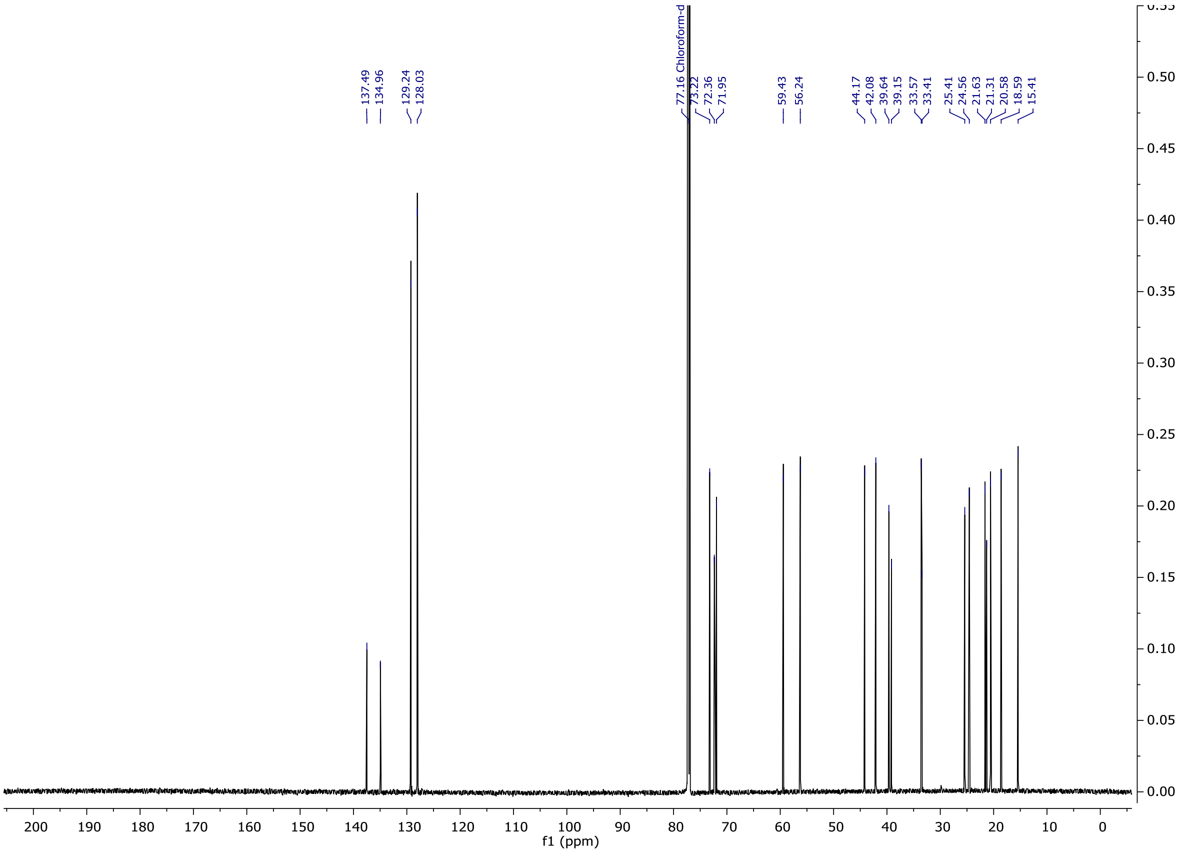

^{1}H-NMR (700 MHz, $CDCl_3$)

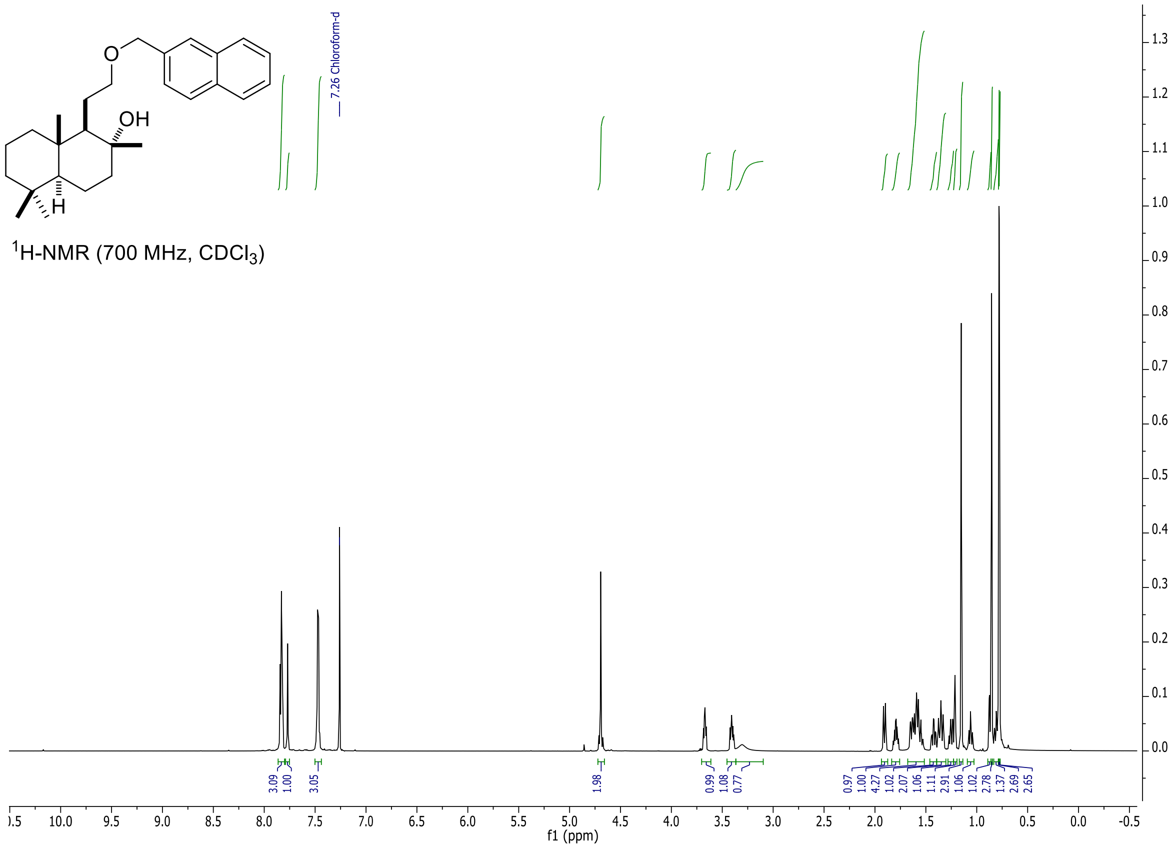

^{13}C-NMR (176 MHz, $CDCl_3$)

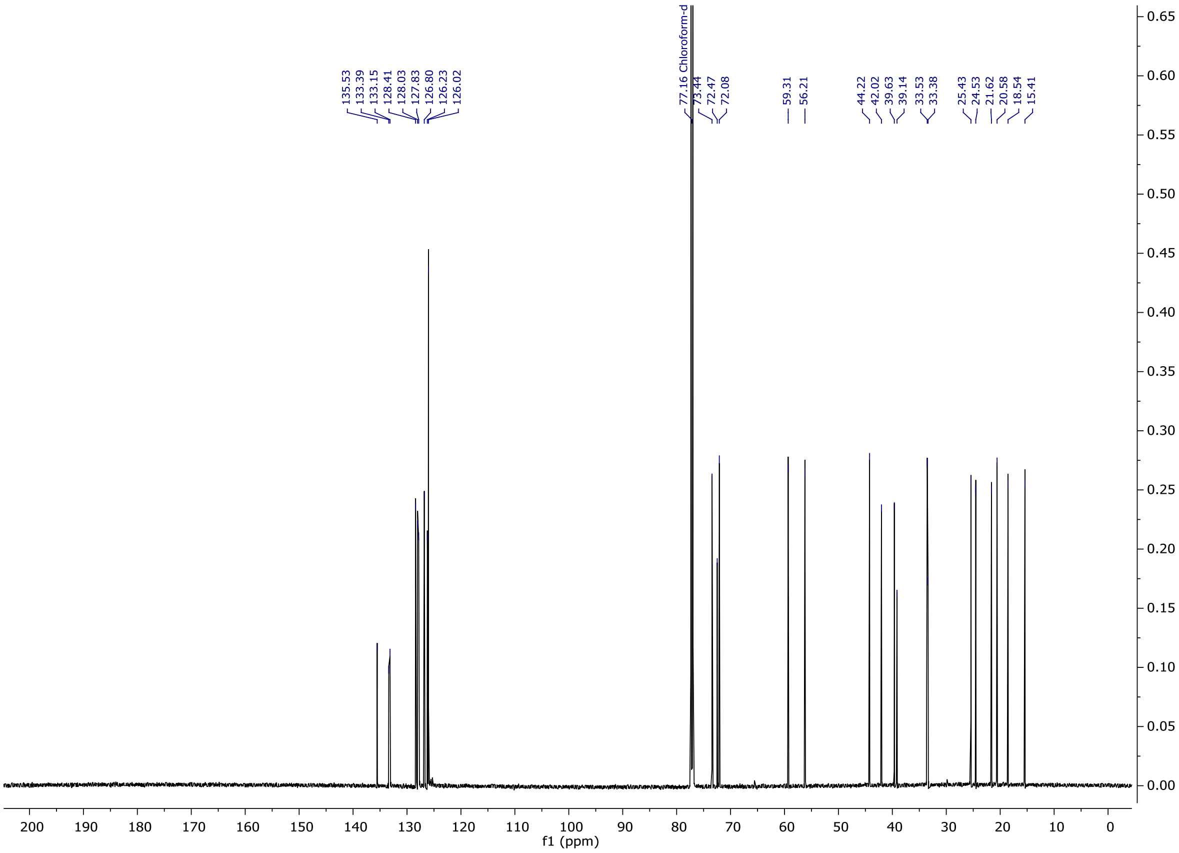

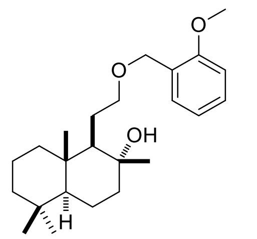

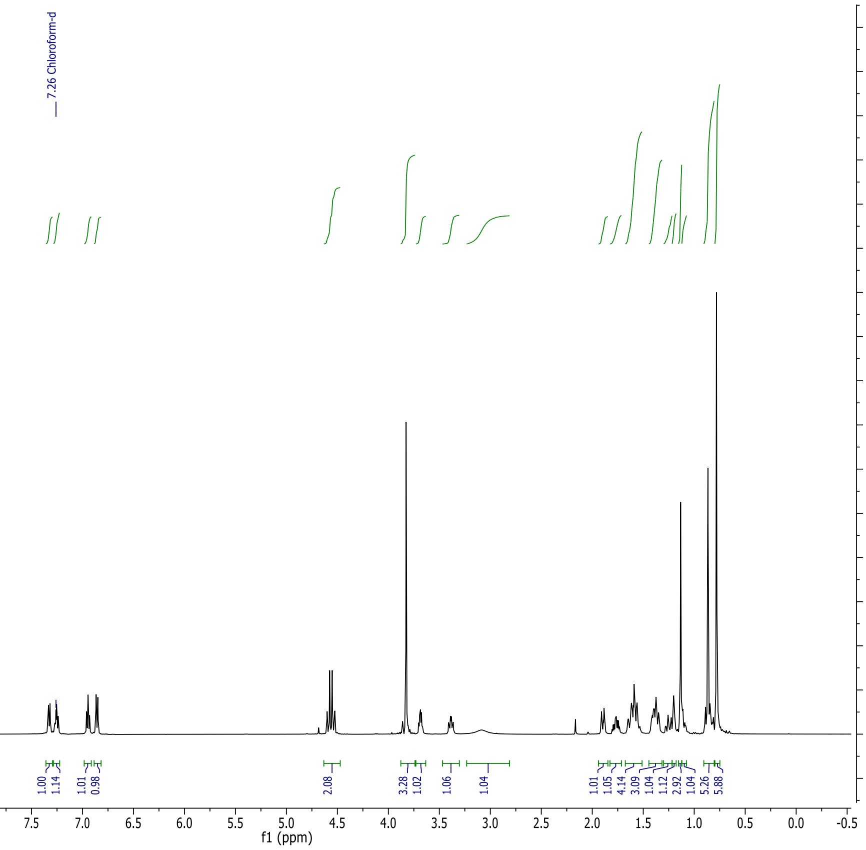

^{1}H-NMR (500 MHz, $CDCl_3$)

^{13}C-NMR (126 MHz, $CDCl_3$)

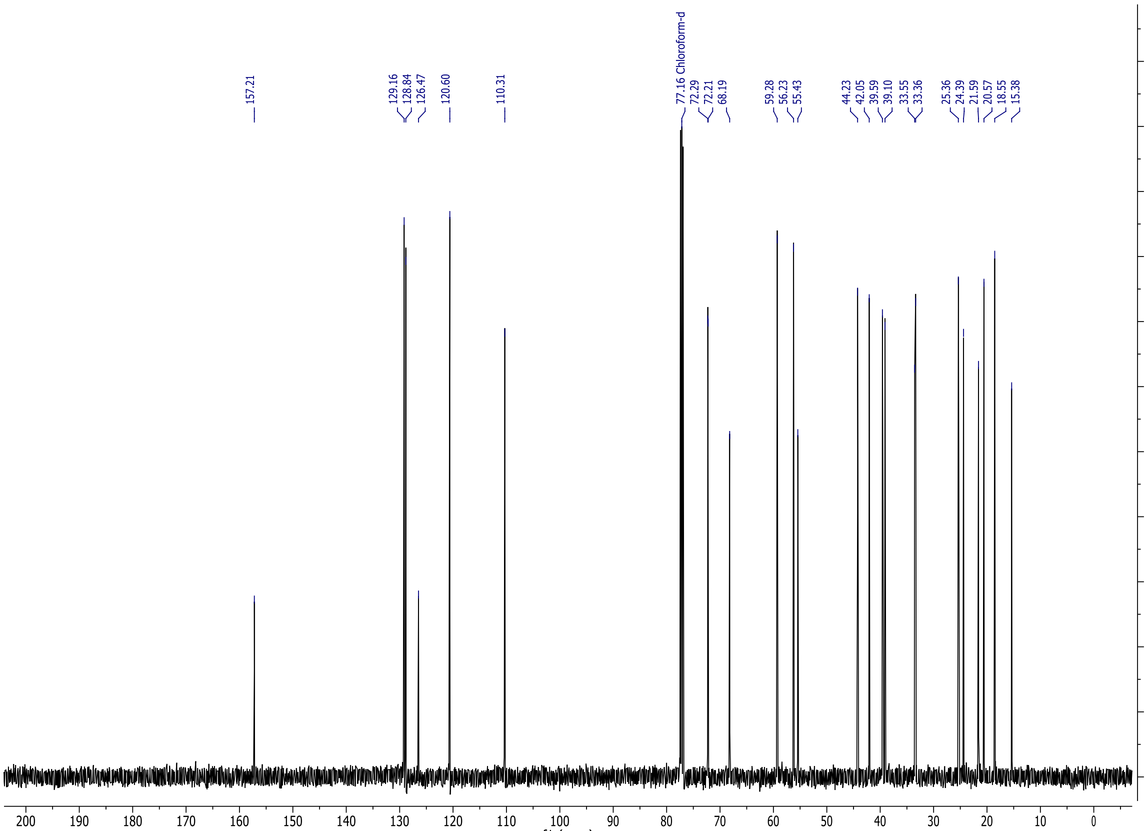

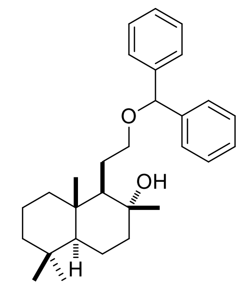

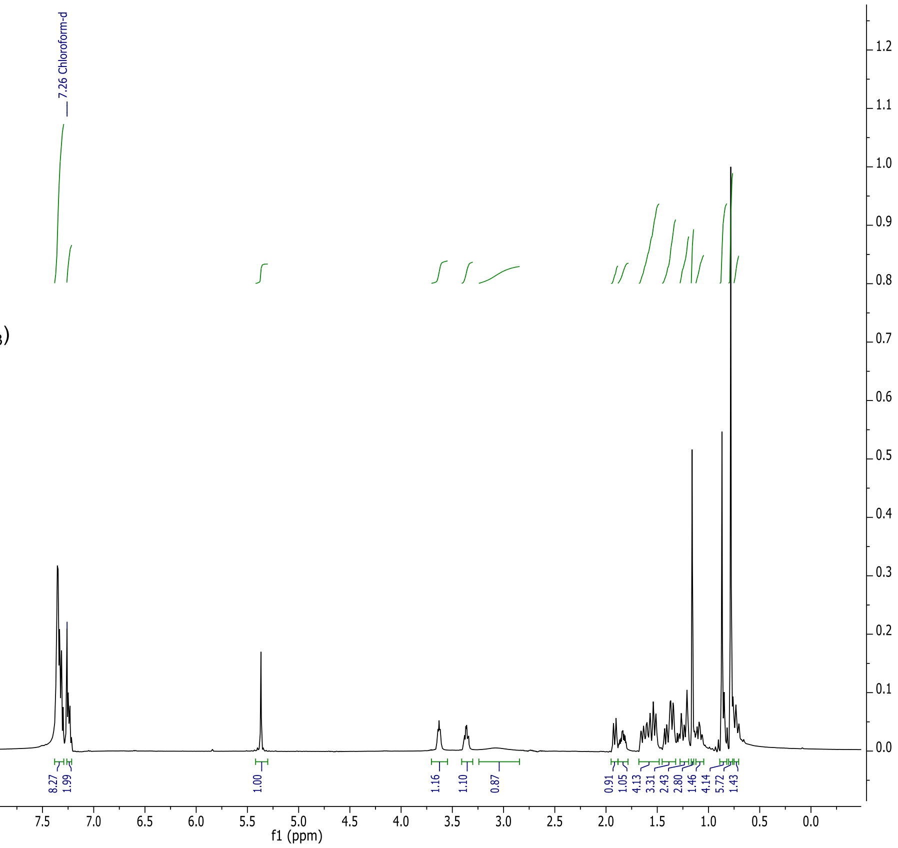

^{1}H-NMR (500 MHz, $CDCl_3$)

^{13}C-NMR (126 MHz, $CDCl_3$)

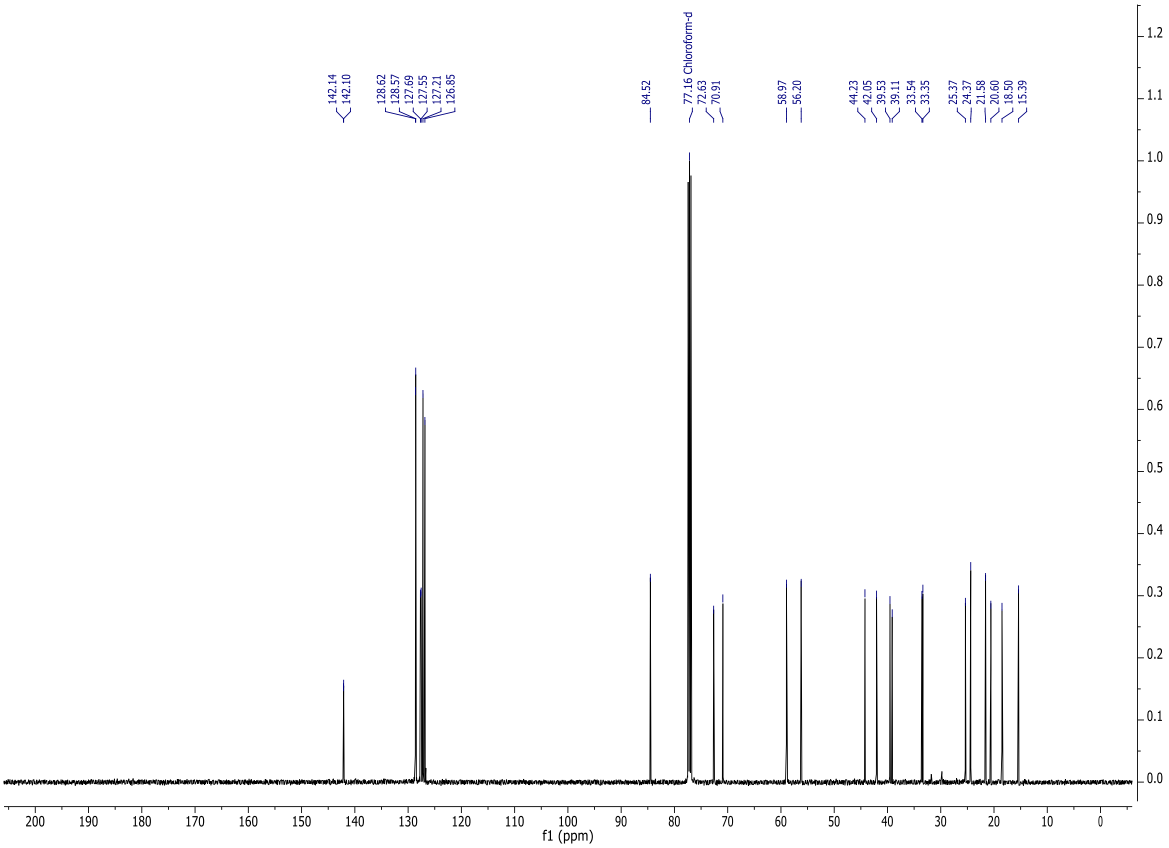

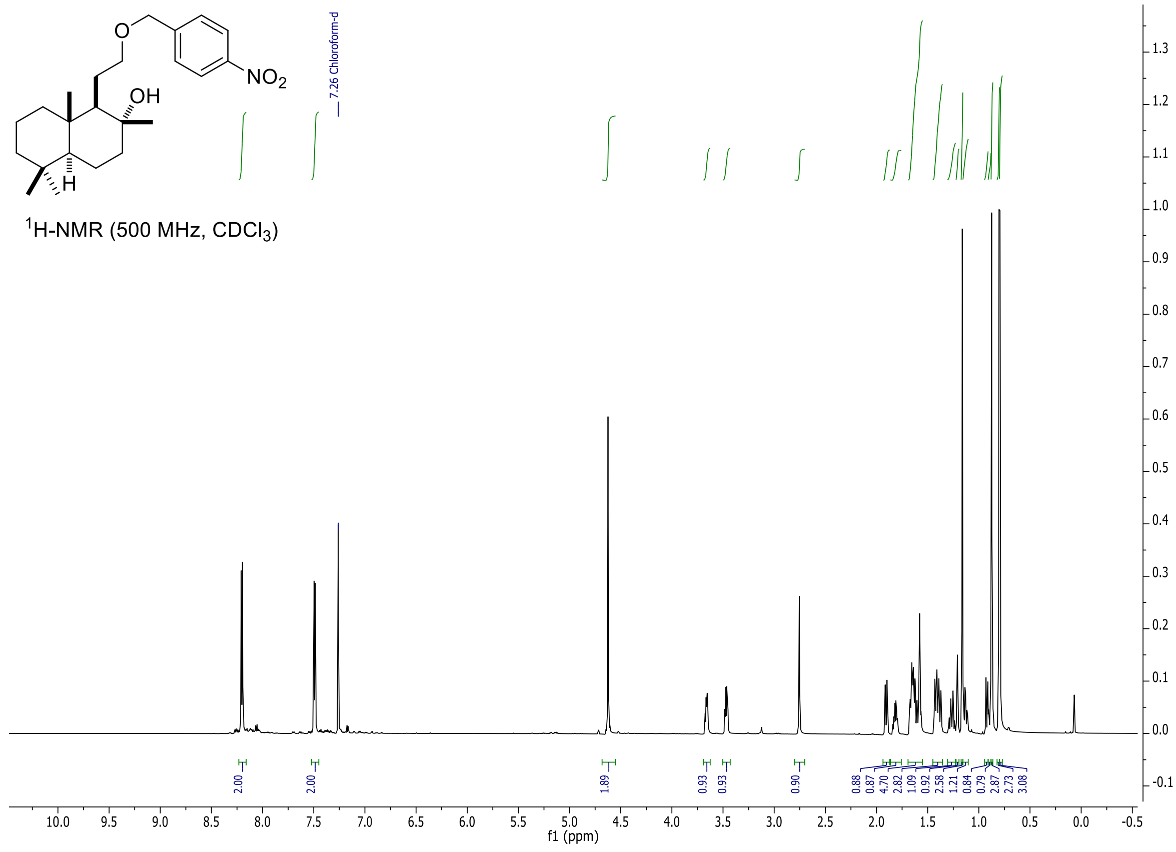
NO2
OH
H
1H-NMR (500 MHz, CDCl3)
7.26 Chloroform-d
f1 (ppm)

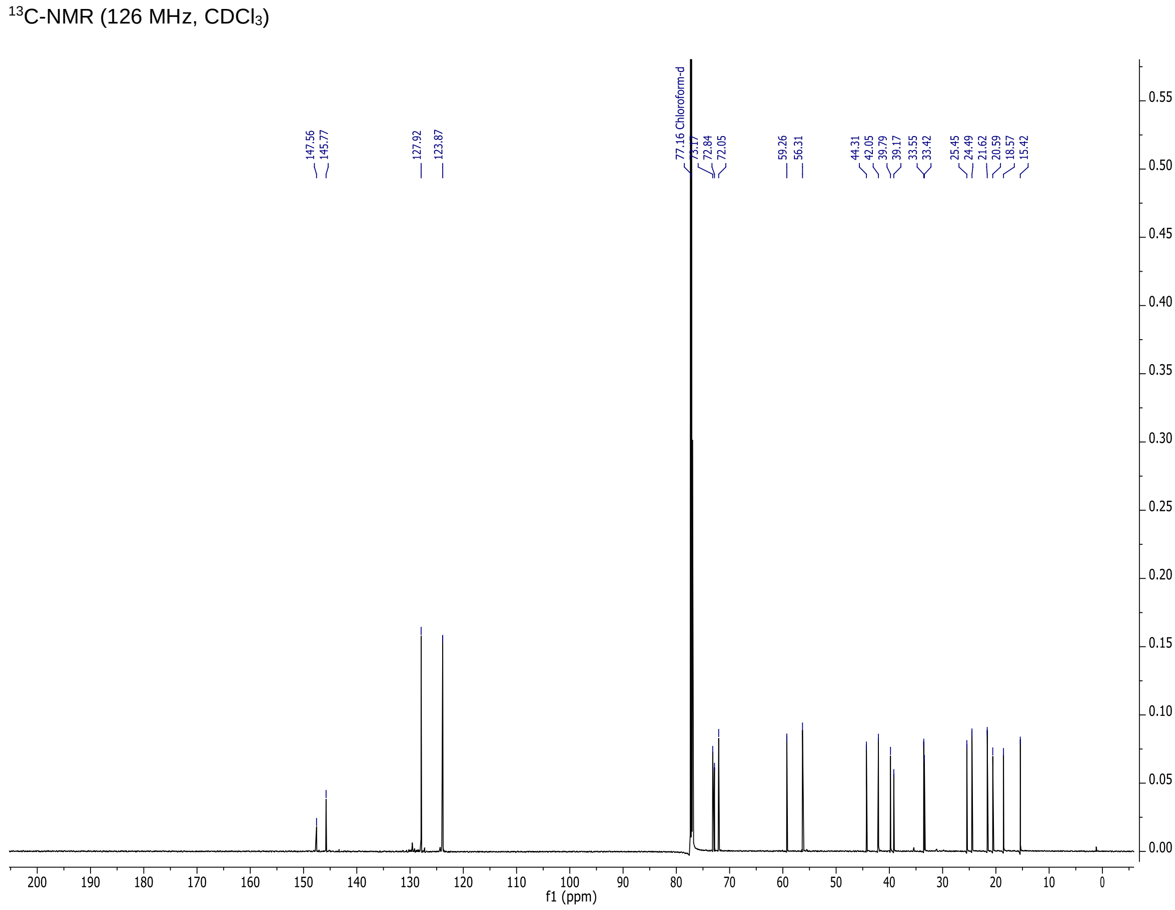
13C-NMR (126 MHz, CDCl3)
147.56
145.77
127.92
123.87
77.16 Chloroform-d
73.17
72.84
72.05
59.26
56.31
44.31
42.05
39.79
39.17
33.55
33.42
25.45
24.49
21.62
20.59
18.57
15.42
f1 (ppm)

^{1}H-NMR (700 MHz, $CDCl_3$)

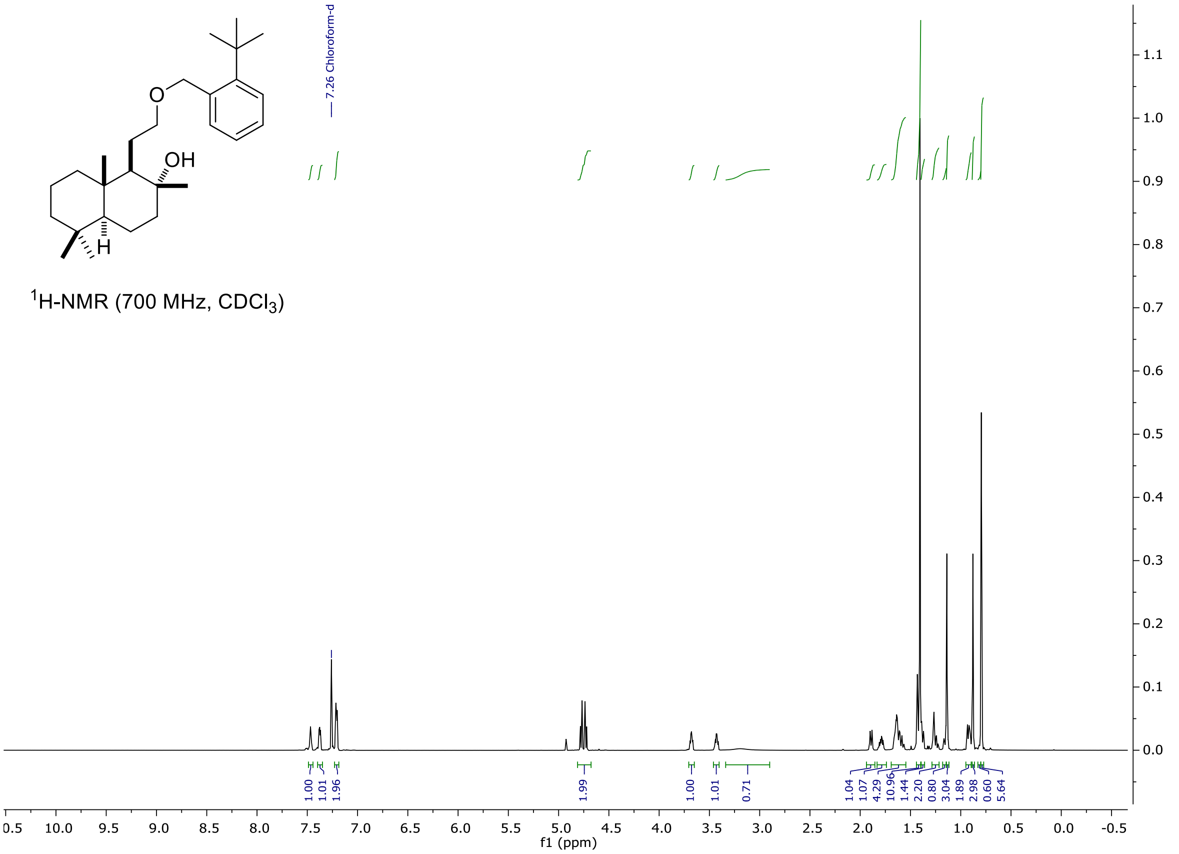

^{13}C-NMR (176 MHz, $CDCl_3$)

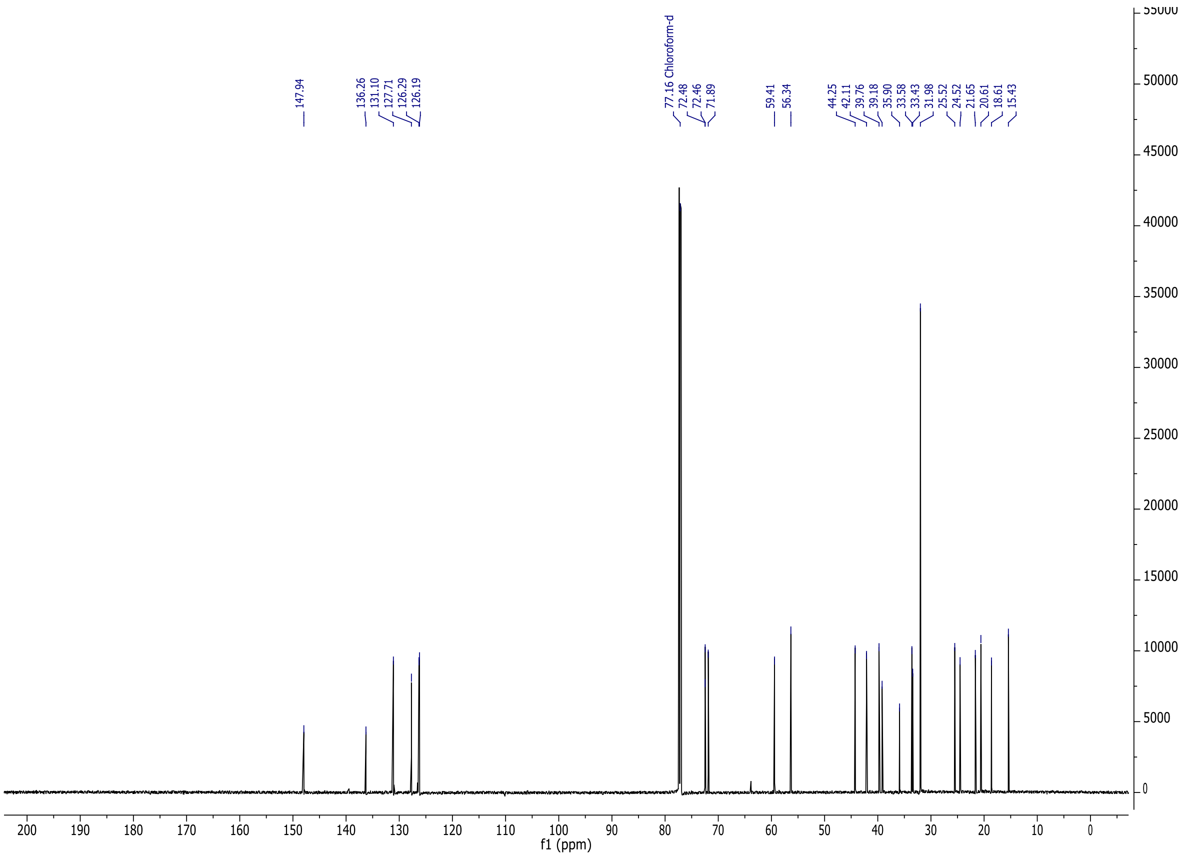

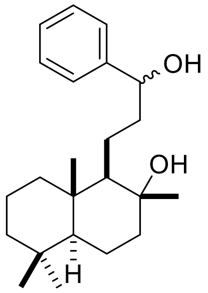

S22

^{1}H-NMR (500 MHz, $CDCl_3$)

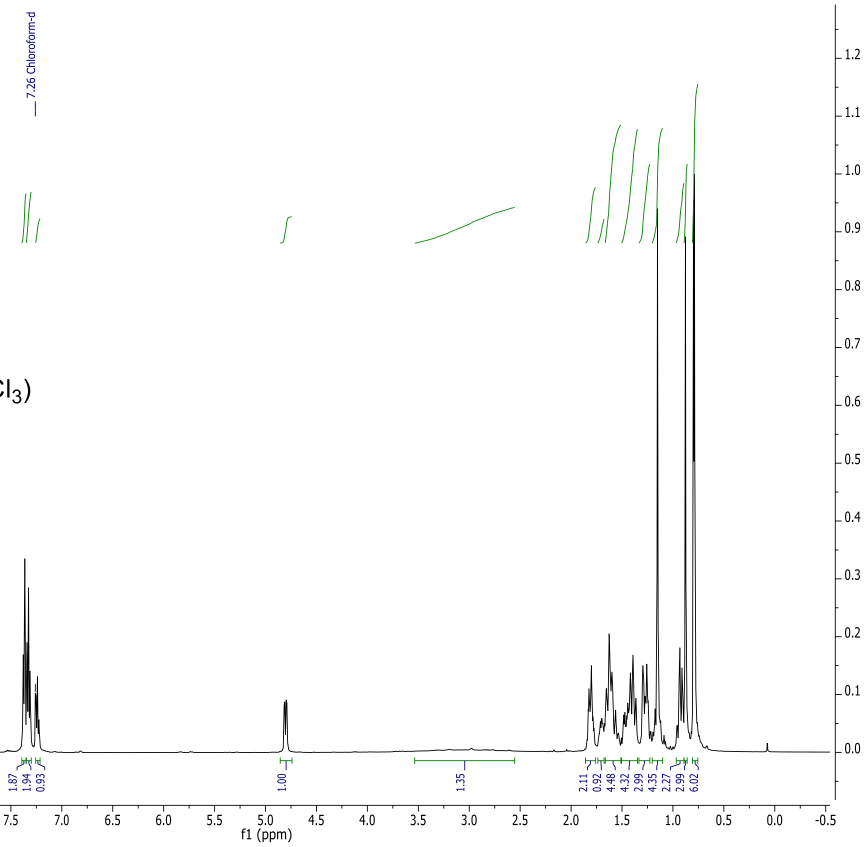

^{13}C-NMR (126 MHz, $CDCl_3$)

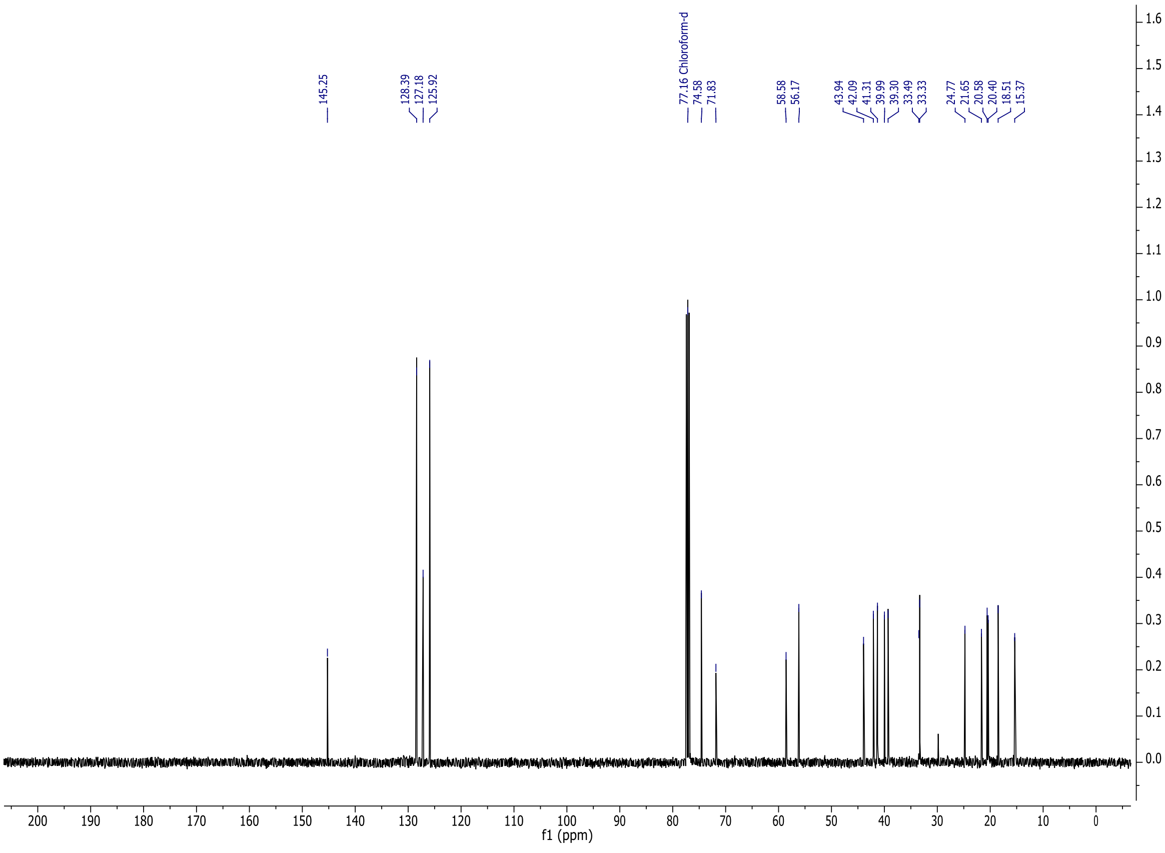

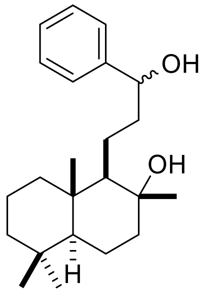

S23

^{1}H-NMR (700 MHz, $CDCl_3$)

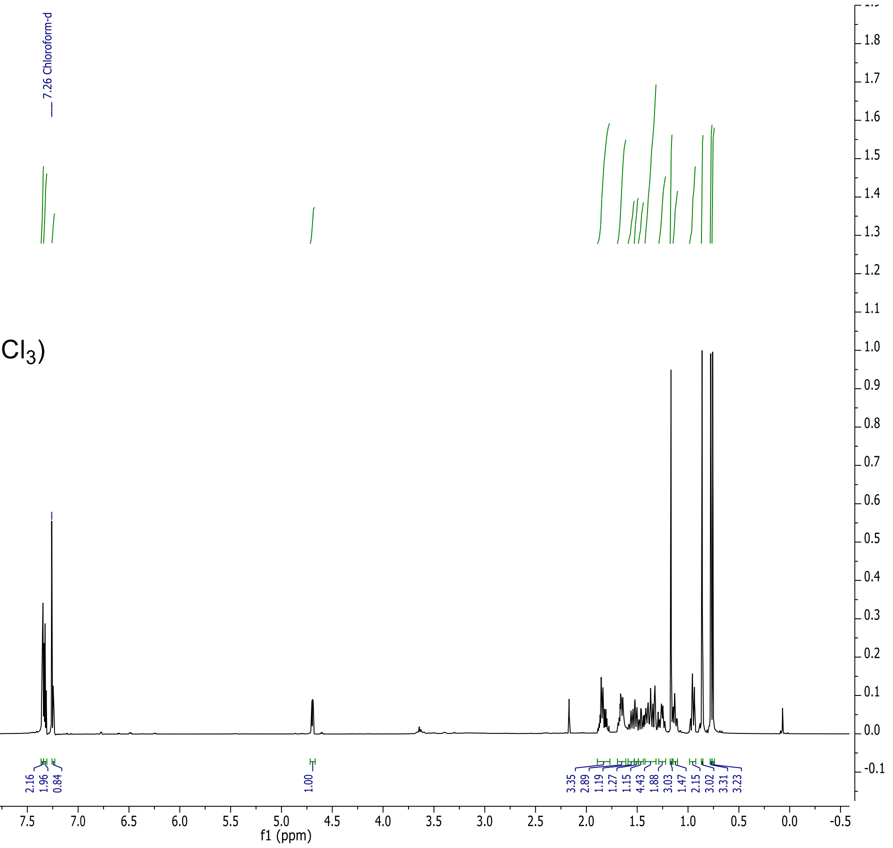

^{13}C-NMR (176 MHz, $CDCl_3$)

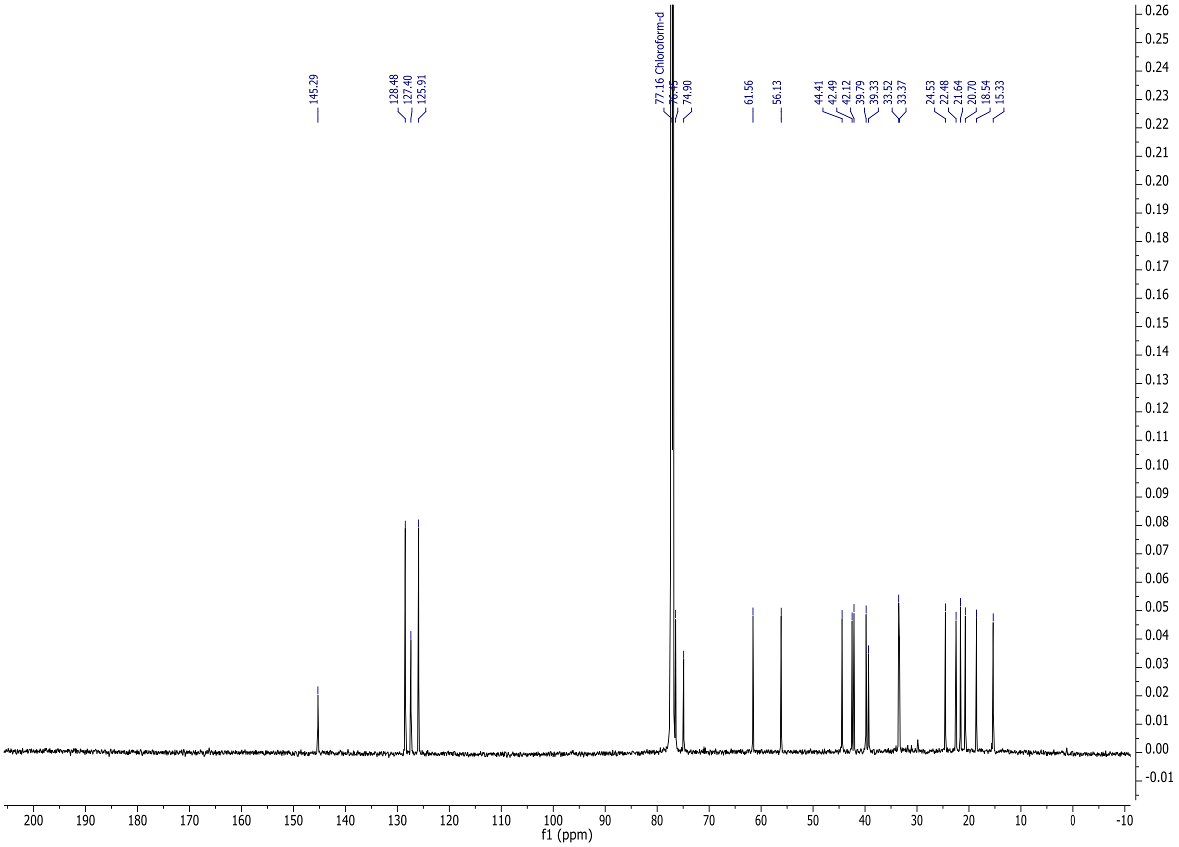

5. Literature Comparison: Polyene Cyclizations terminated by Aliphatic Alcohols

Table 11 Selected examples of ring systems produced via polyene cyclization terminated by aliphatic alcohols.

Entry	Number and ring size of carbocycles formed (6 = Cycloxehane, 5 = Cyclopentane)	Oxygen containing ring formed	Nucleo-phile	Reagents	Yield (ratio of cyclization products)	Lit.
1	6,6	Tetrahydro-furan	Primary alcohol	1. $[Ir(COD)Cl]_2$ (4 mol%), **R1** (16 mol%), $Zn(OTf)_2$ (20 mol%), DCE; 2. $BF_3{\cdot}OEt_2$, CH_2Cl_2	79% (2 steps) (10:1, >99% *ee*)	[19]
2	6,6	Tetrahydro-furan	Primary alcohol	(*R*)-BINOL-Me·$SnCl_4$, CH_2Cl_2	54% (56 (42% *ee*) :26:9:9)	[20]
3	6,6	Tetrahydro-furan	Primary alcohol	(dppe)PtI_2 (10 mol%), $AgBF_4$ (22 mol%), 2.1 Ph_3COMe resin, $EtNO_2$	90%	[21]
4	6,6	Tetrahydro-furan	Primary alcohol	FSO_3H, 2-nitropropane	78% (40:35:2:1)	[22]
5	6,6,5	Tetrahydro-furan	Secondary alcohol	picric acid, $MeNO_2$	7	[23]
6	6,6	Tetrahydro-furan	Primary alcohol	$ClSO_3H$	75% (1.2:1)	[24]
7	6	Tetrahydro-pyran	Tertiary alcohol	(*R*)-BINOL-Me·$SnCl_4$, CH_2Cl_2	58% (88 (56% *ee*) :12)	[20]
8	6	Tetrahydro-pyran	Tertiary alcohol	**R2**·$SnCl_4$	74% (81:19)	[25]
9	6,6	Tetrahydro-pyran	Primary alcohol	FSO_3H, 2-nitropropane	70%	[26]
10	6,6	Tetrahydro-pyran	Secondary alcohol	$Hg(TFA)_2$, $C_6H_5N(CH_3)_2$, then NaCl	15%	[27]
11	6,6	Tetrahydro-pyran	Primary alcohol	PhSeOTf	7%	[28]

12	6	Oxepane	Secondary alcohol	$Hg(TFA)_2$, $MeNO_2$ then KBr, Br_2, O_2, LiBr, py	30% (1:1)	[29]
13	6	Oxepane	Secondary alcohol	2,4,4,6-tetrabromo-cyclohexa-2,5-dienone, $MeNO_2$	12% (19:81)	[30]
14	6	Oxepane	Secondary alcohol	$Hg(TFA)_2$, $MeNO_2$ then KBr, Br_2, O_2, LiBr, py	29% (3.1:1)	[31]
15	6,6	Oxepane	Tertiary alcohol	squalene cyclase from *Alicyclo-bacillus acidocaldarius*	19.3%	[32]
16	6,6	Oxepane	Tertiary alcohol	BmeTC	18% (64:36)	[9]
17	6,6	Oxepane	Tertiary alcohol	$EtAlCl_2$, CH_2Cl_2	32% (1.7:1)	This work

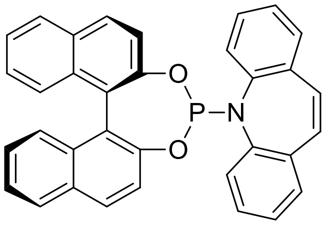

R1

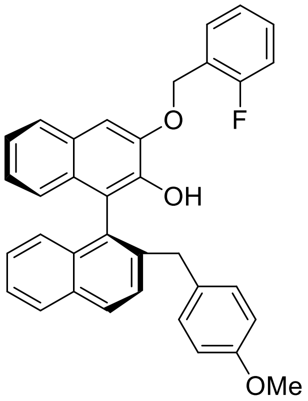

R2

These examples highlight the difficult formation of fused ring systems containing oxepane rings with tertiary alcohols in polyene cyclizations, as previously only enzymatic transformations of this kind were known (Entry 15 and 16). Furthermore, the examples indicate that $EtAlCl_2$, while being widely used in polyene cyclizations terminated by allylsilanes and silyl enol ethers,[33] has not been used frequently in alcohol terminated polyene cyclizations and therefore this constitutes an extension of the previously known scope of terminating nucleophiles.

6. Enzymatic Studies

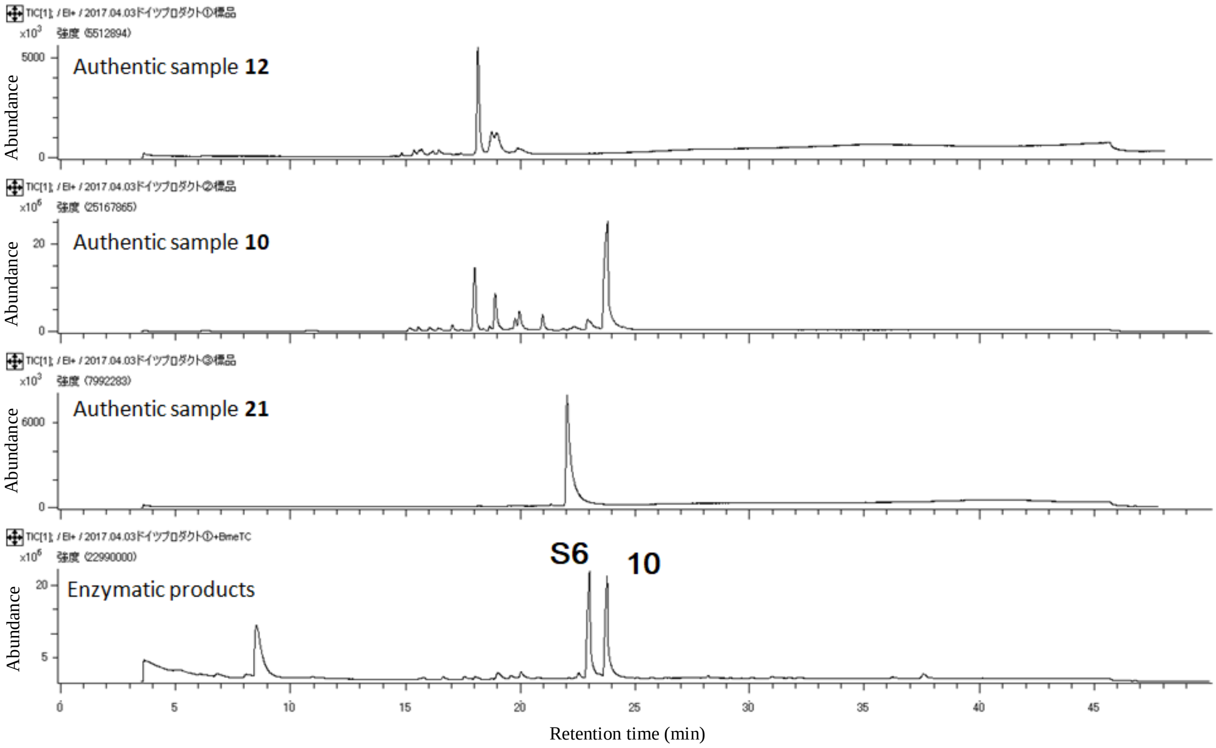

Fig. 2 Top: GC-MS chromatogram of authentic **12**. Second from the top: GC-MS chromatogram of authentic **10** (possible partial degradation due to transportation). Third from the top: GC-MS chromatogram of authentic **21**. Bottom: GC-MS chromatogram of the reaction products of **12** with BmeTC. All measurements performed with "GC-MS Niigata".

The mass spectrometric studies indicated that one of the products formed from the enzymatic reaction of **12** with BmeTC (Fig. 2, Bottom) has a similar GC-MS retention time and comparable GC-MS mass spectrum as authentic **10** (Fig. 2, Second from the top and Fig. 3 respectively). In addition, **21** could not be detected in the enzymatic reaction mixture (within the limits of detection) (Fig. 4).

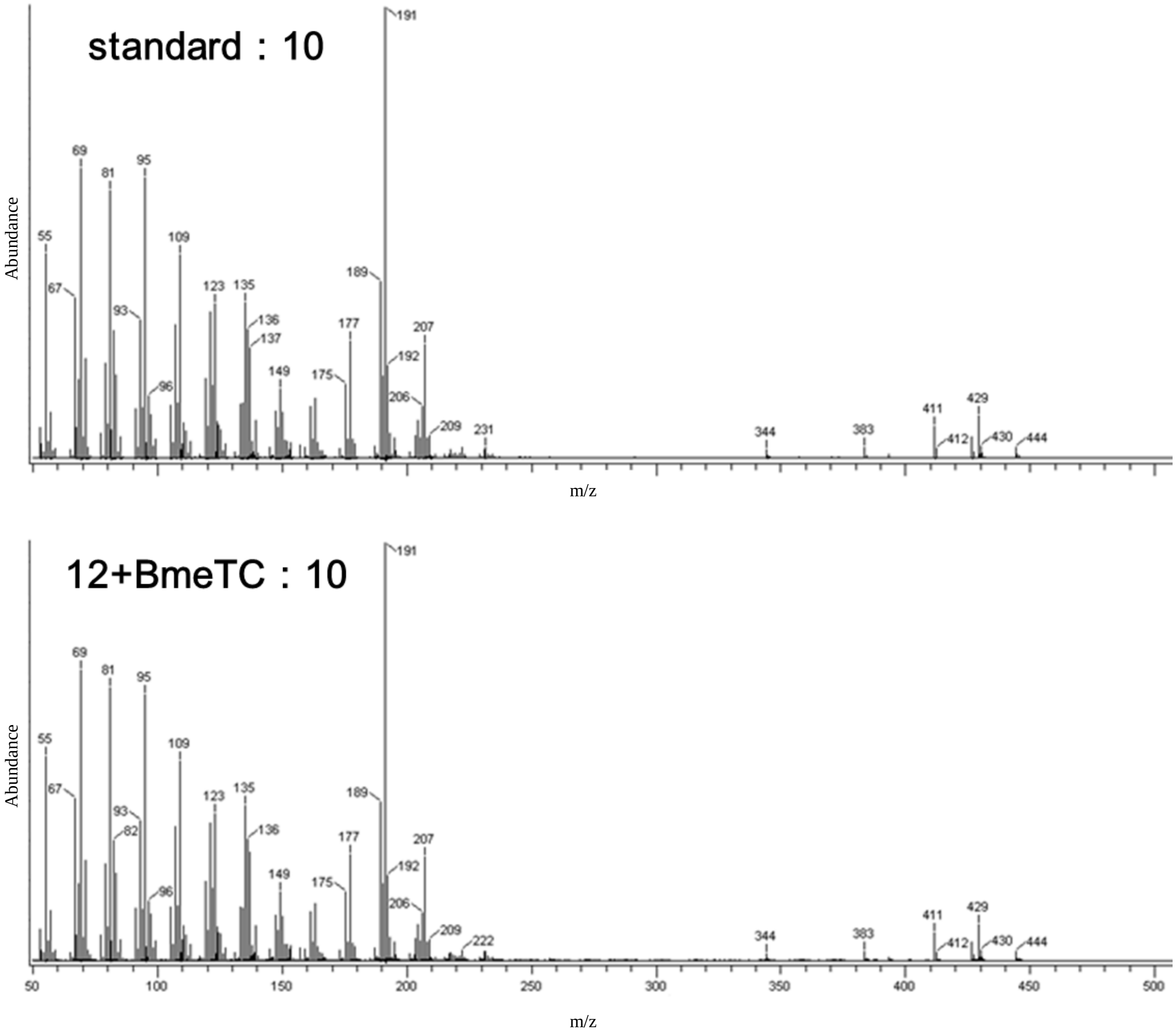

Fig. 3 Top: GC-MS mass spectrum of authentic **10** (measured with "GC-MS Niigata"). Bottom: GC-MS mass spectrum of enzymatic reaction product with a similar retention time as authentic **10** (measured with "GC-MS Niigata").

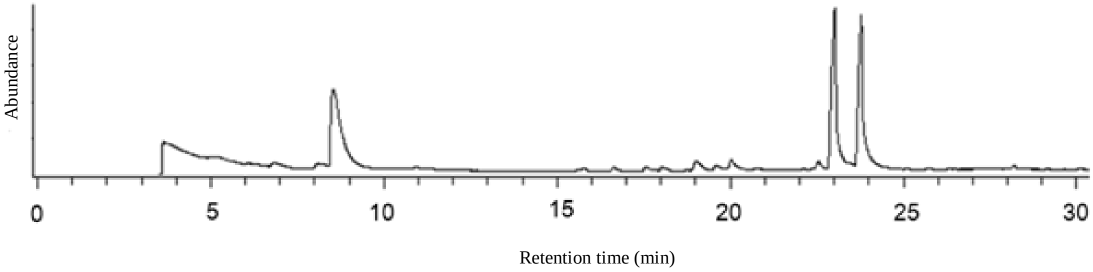

Fig. 4 Zoom in the GC-MS chromatogram of the reaction of **12** with BmeTC (measured with "GC-MS Niigata").

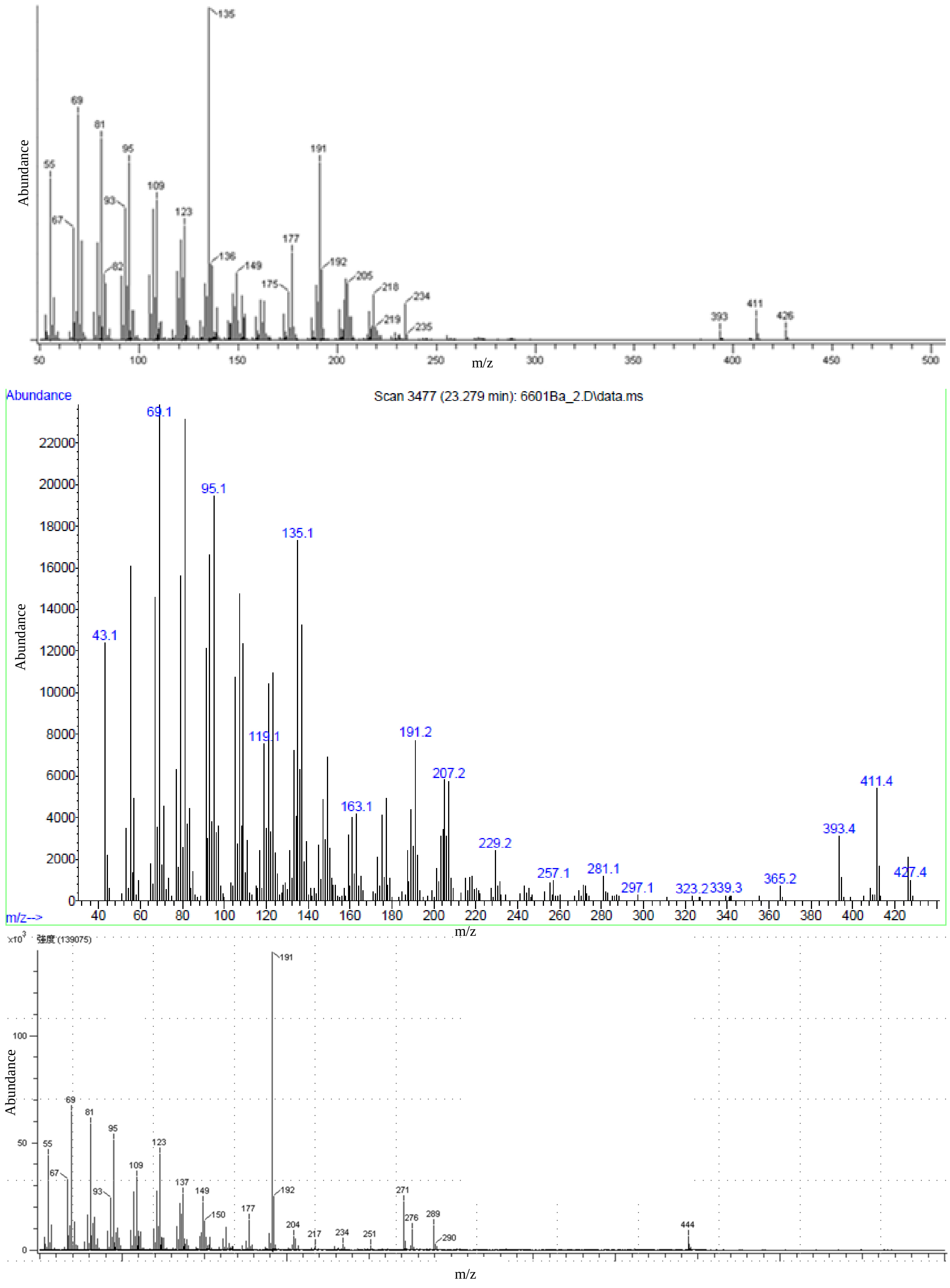

Fig. 5 Top: GC-MS mass spectrum of compound **S6** from the reaction of **12** with BmeTC (measured with "GC-MS Niigata"). Middle: GC-MS mass spectrum of authentic **S6** (measured with "GC-MS Berlin"). Bottom: GC-MS mass spectrum of authentic compound **21** (measured with "GC-MS Niigata").

Mass spectrometric studies indicated that the fragmentation pattern of **S6** synthetized from the enzymatic reaction of **12** with BmeTC (Fig. 2 Bottom and Fig. 5 Top) shows similarities to the fragmentation pattern of **S6** synthesized from α-onocerin **6**[15] by mono deoxygenation (Fig. 5 Middle; **S6**, for the synthesis see page 29 in the Supporting Information).[34] While the relative configuration and the possible hydration level of the elimination product could not be elucidated, its connectivity resembles **S6**. The tetracyclic product results from an epoxypolyene dicyclization followed by elimination.

"GC-MS Niigata":

GC-MS (injection temperature, 300°C; oven temperature, 220–300°C at an increment of 3°C min^{-1}) was performed on a JMS-T100GCV spectrometer (JEOL) equipped with a DB-1 capillary column (30 m × 0.25 mm × 0.25 μm; J&W Scientific, Inc.), using the EI mode operated at 70 eV. HRMS was performed on a JMS-T100LP spectrometer (JEOL) using ESI mode.

"GC-MS Berlin":

GC/MS system consisting of a 5977E MSD single-quadrupole mass spectrometer (EI-Mode (70 eV)) with a 7820A GC by Agilent Technologies (Agilent 190915-433UI, 30 m x 250 μm x 0.25 μm). Injection temperature: 300 °C, column temperature: 220-300 °C (increment 3 °C/min).

Enzymatic assay for BmeTC

Escherichia coli BL21(DE3) harboring pColdTF-BmeTC was grown at 37°C in LB medium (1 L) with 100 μg mL^{-1} ampicillin. Expression of the recombinant protein was induced by adding 0.1 mM isopropyl β-D-1-thiogalactopyranoside when the OD_{600} reached ~0.6. Further cultivation of BL21(DE3) recombinants was performed for 24 h at 15°C. *E. coli* cells expressing recombinant BmeTC were harvested by centrifugation and resuspended in 15 mL/5 g *E. coli* cells of buffer A containing 50 mM Tris-HCl (pH 7.5), 2.5 mM dithiothreitol, 1 mM EDTA, and 0.1% Tween80. The cells were disrupted by sonication with UP200s (Hielscher Ultrasonics GmbH, Teltow, Germany) at 4–10°C for 40 min. The resulting suspension was centrifuged at 10,000 × *g* for 20 min. The pellet was discarded, and the resulting supernatant was used as a cell-free extract. The reaction mixture for analyzing the enzymatic activity of BmeTC contained substrate **12** (0.1 mg) emulsified with Tween80 (2 mg) in buffer A (1 mL), and 4 mL of cell-free extract containing BmeTC in a total volume of 5 mL. The reaction was carried out at 30°C for 64 h and terminated by using a 15% KOH/MeOH solution (6 mL). The lipophilic enzymatic product was extracted from the incubation mixtures with *n*-hexane (5 mL × 3). Tween80 detergent was removed by passing the extract through a short SiO_2 column (*n*-hexane: EtOAc = 100:20) and then subjecting the eluent to GC-MS.

7. Density Functional Calculations

List of Contents

Gaussian Full Reference	115
Methods-QCC analysis	115
Scheme S1	116
Key	116
Figure S1	117
Figure S2	118
Figure S3	119
Figure S4	120
Figure S5	121
Figure S6	122
Figure S7	123
Table S1	124
Coordinates and Energies	124
Figure S1	124
Figure S2	135
Figure S3	153
Figure S4	164
Figure S5	186
Figure S6	196
Figure S7	222

Gaussian Full Reference

Gaussian 09, Revision D.01

M. J. Frisch, G. W. Trucks, H. B. Schlegel, G. E. Scuseria, M. A. Robb, J. R. Cheeseman, G. Scalmani, V. Barone, B. Mennucci, G. A. Petersson, H. Nakatsuji, M. Caricato, X. Li, H. P. Hratchian, A. F. Izmaylov, J. Bloino, G. Zheng, J. L. Sonnenberg, M. Hada, M. Ehara, K. Toyota, R. Fukuda, J. Hasegawa, M. Ishida, T. Nakajima, Y. Honda, O. Kitao, H. Nakai, T. Vreven, J. A. Montgomery, Jr., J. E. Peralta, F. Ogliaro, M. Bearpark, J. J. Heyd, E. Brothers, K. N. Kudin, V. N. Staroverov, T. Keith, R. Kobayashi, J. Normand,

K. Raghavachari, A. Rendell, J. C. Burant, S. S. Iyengar, J. Tomasi, M. Cossi, N. Rega, J. M. Millam, M. Klene, J. E. Knox, J. B. Cross, V. Bakken, C. Adamo, J. Jaramillo, R. Gomperts, R. E. Stratmann, O. Yazyev, A. J. Austin, R. Cammi, C. Pomelli, J. W. Ochterski, R. L. Martin, K. Morokuma, V. G. Zakrzewski, G. A. Voth, P. Salvador, J. J. Dannenberg, S. Dapprich, A. D. Daniels, O. Farkas, J. B. Foresman, J. V. Ortiz, J. Cioslowski, and D. J. Fox, Gaussian, Inc., Wallingford CT, 2013.

Methods-QCC analysis

Calculations were performed with GAUSSIAN09.[35] Geometries were optimized using the B3LYP method with the basis set of 6-31G(d).[36] All stationary points were characterized as minima or transition state structures using frequency calculations at the same level. All reported energies include zero-point energy corrections (unscaled) from the frequency calculations at the same level. Intrinsic reaction coordinate (IRC) calculations were used for further characterization of transition state structures.[37] mPW1PW91//6-31+G(d,p)[38] energies are also shown, since it is known that B3LYP underestimates the relative energies of cyclic structures versus acyclic isomers.[38] These energies do not include zero-point energy corrections. The validity of this computational approach for examining terpene-forming carbocation rearrangements is well-established.[39] Structural images were created with *Ball&Stick*.[40]

Scheme S1 |

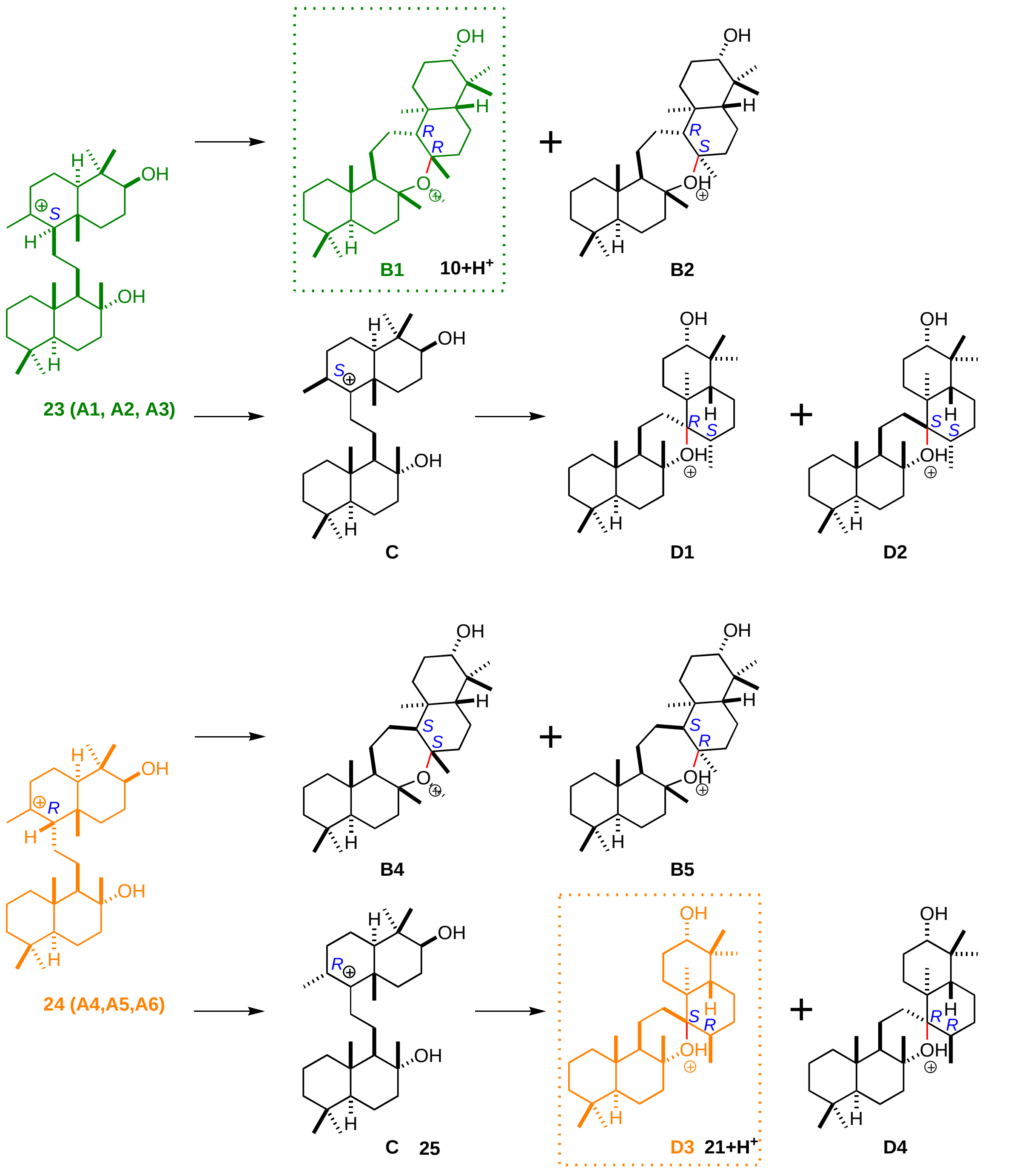

Key

Capital letters are used to label carbocations and subsequent numbers to discern different conformers or stereoisomers.

Structures A1, A2 and A3 in the Supporting Information are conformers of the structure **23** in the manuscript.

B1 in the Supporting Information corresponds **10**+H^+ in the manuscript.

The structures A4, A5 and A6 in the Supporting Information are conformers of the structure **24** in the manuscript.

D3 in the Supporting Information corresponds **21**+H^+ in the manuscript.

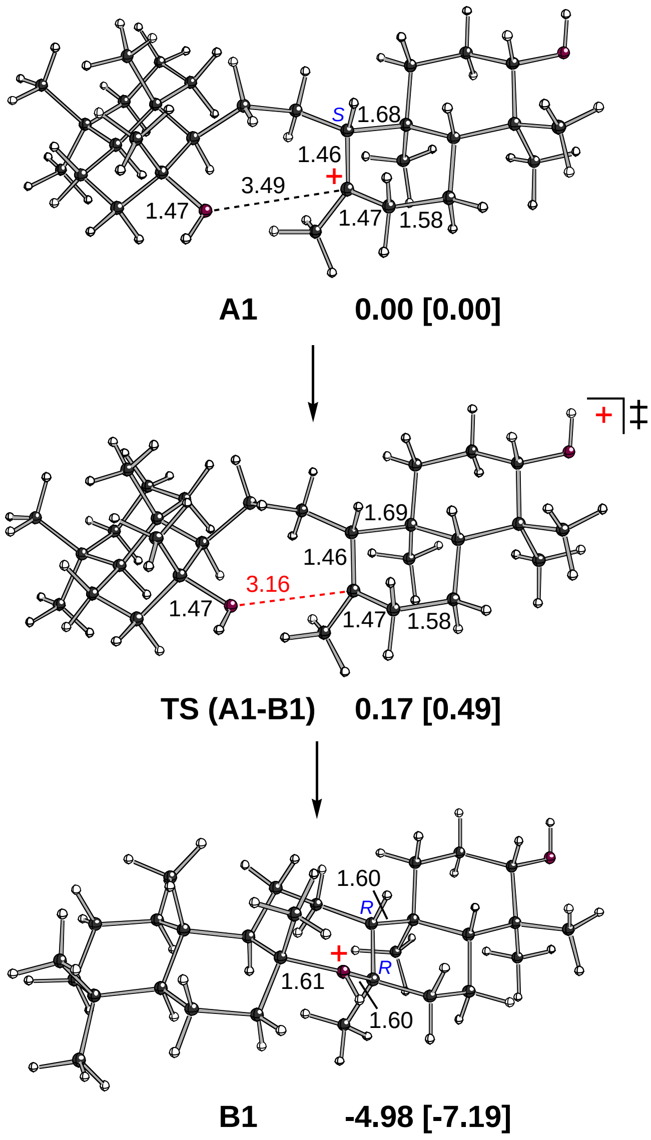

Figure S1 | Formation of **B1 (10+H$^+$)**. Energies (kcal/mol; B3LYP/6-31G(d)//B3LYP/6-31G(d) in normal texts and mPW1PW91/6-31+G(d,p)//B3LYP/6-31G(d) in brackets) are shown.

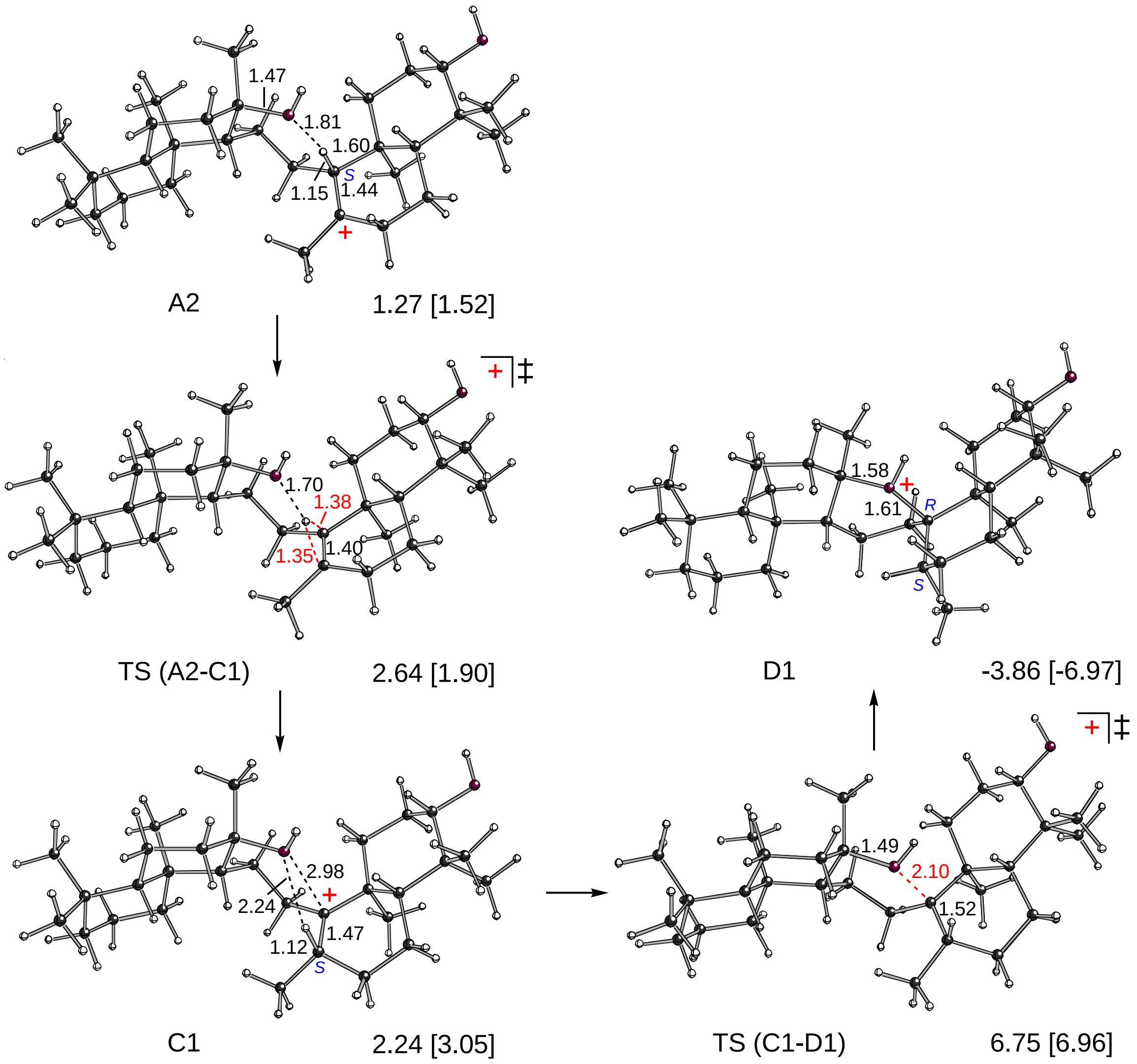

Figure S2 | Formation of **D1**. Energies (kcal/mol; B3LYP/6-31G(d)//B3LYP/6-31G(d) in normal texts and mPW1PW91/6-31+G(d,p)//B3LYP/6-31G(d) in brackets) are shown. 1,2-Hydride shift step has a barrier of <1kcal/mol.

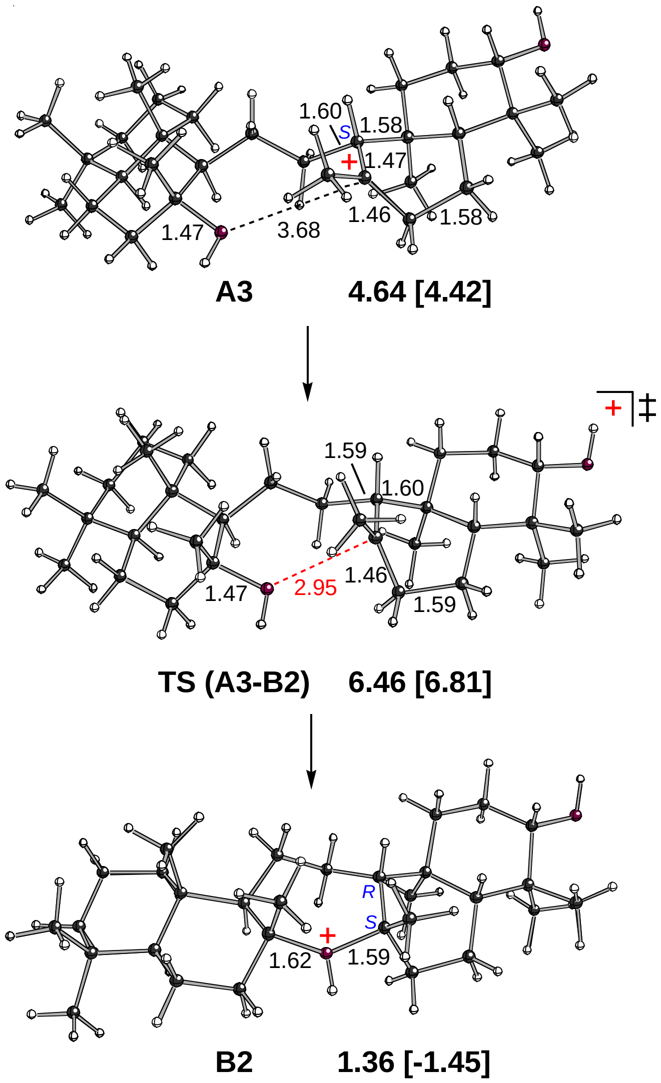

Figure S3 | Formation of **B2**. Energies (kcal/mol; B3LYP/6-31G(d)//B3LYP/6-31G(d) in normal texts and mPW1PW91/6-31+G(d,p)//B3LYP/6-31G(d) in brackets) are shown.

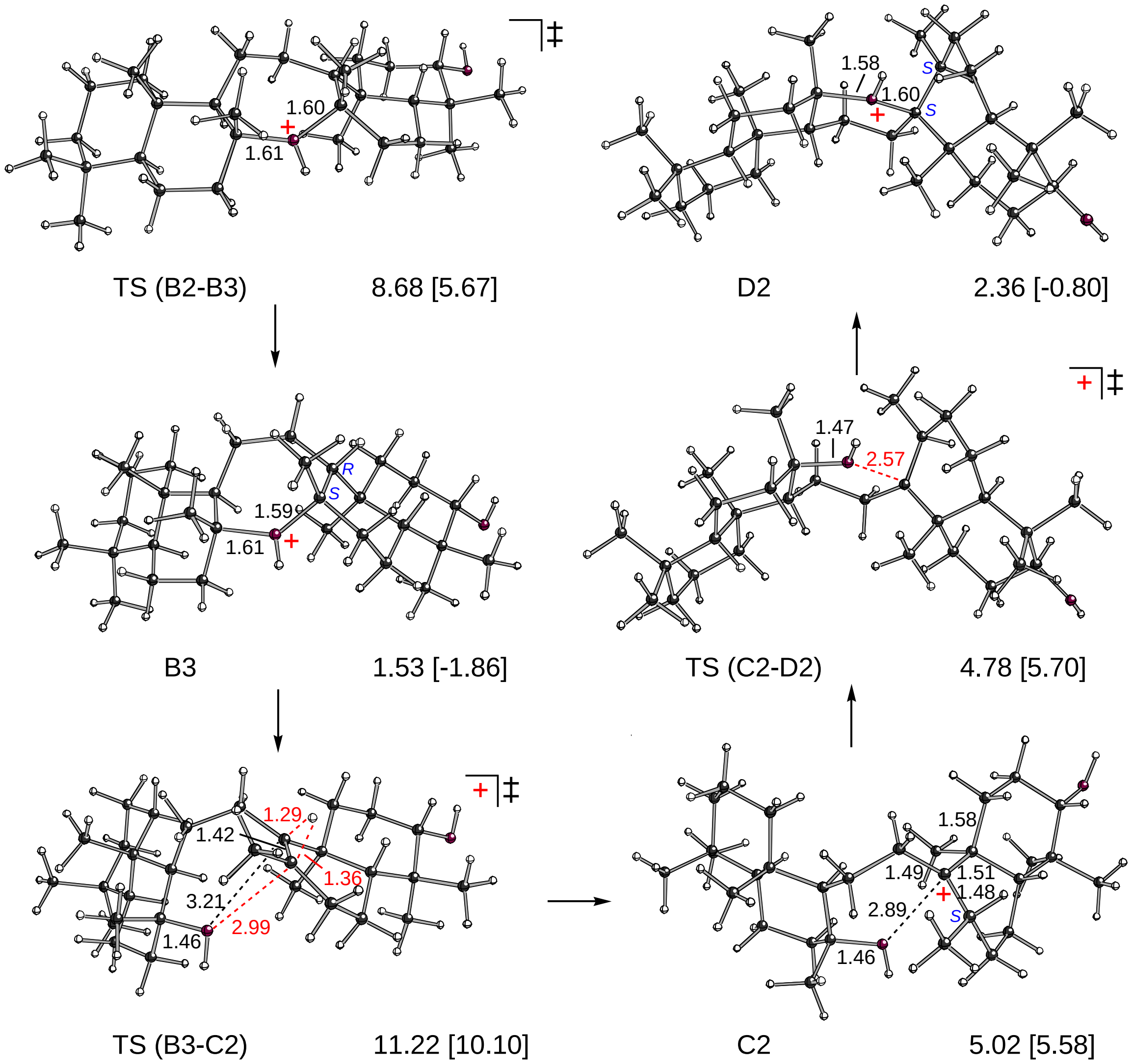

Figure S4| Formation of **D2**. Energies (kcal/mol; B3LYP/6-31G(d)//B3LYP/6-31G(d) in normal texts and mPW1PW91/6-31+G(d,p)//B3LYP/6-31G(d) in brackets) are shown. The B3→C2 conversion is a concerted reaction combined with ring opening/1,2-hydride shifting events. See Figure 2 for a different 1,2-hydride shift, which has a significantly smaller energy barrier.

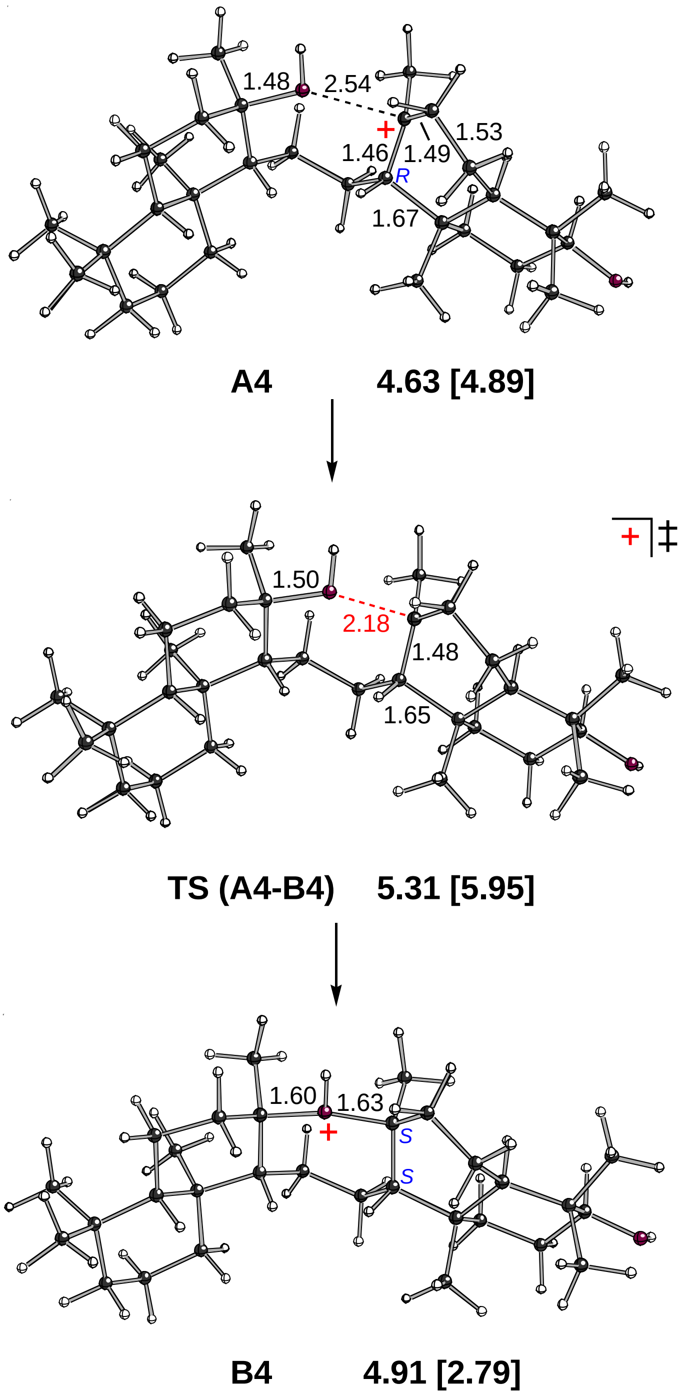

Figure S5 | Formation of **B4**. Energies (kcal/mol; B3LYP/6-31G(d)//B3LYP/6-31G(d) in normal texts and mPW1PW91/6-31+G(d,p)//B3LYP/6-31G(d) in brackets) are shown.

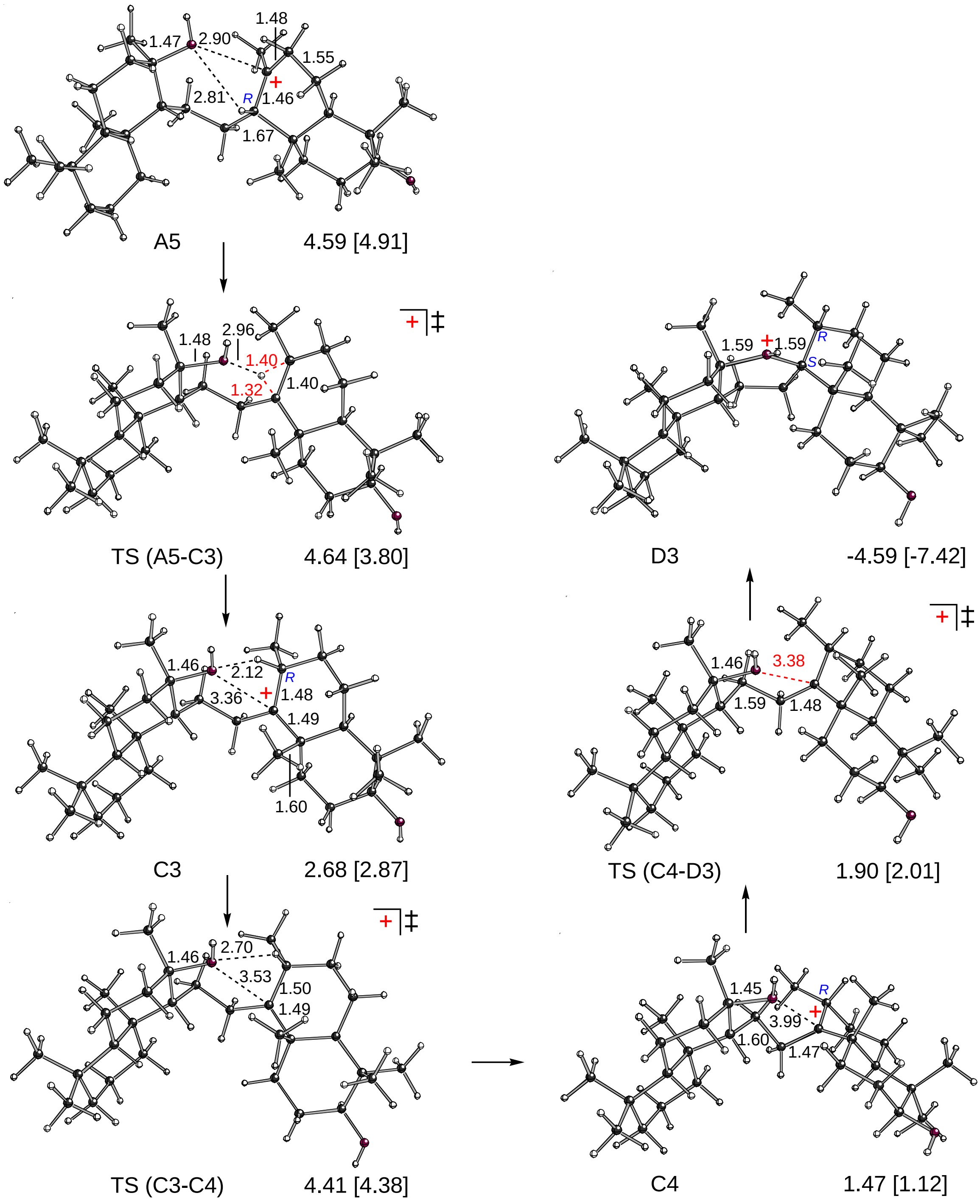

Figure S6 | Formation of **D3** (**$21+H^+$**). Energies (kcal/mol; B3LYP/6-31G(d)//B3LYP/6-31G(d) in normal texts and mPW1PW91/6-31+G(d,p)//B3LYP/6-31G(d) in brackets) are shown. Computed energy barrier for 1,2-hydride shift for **A5** is small. Overall the highest energy step is conformation change of **C** for cyclization.

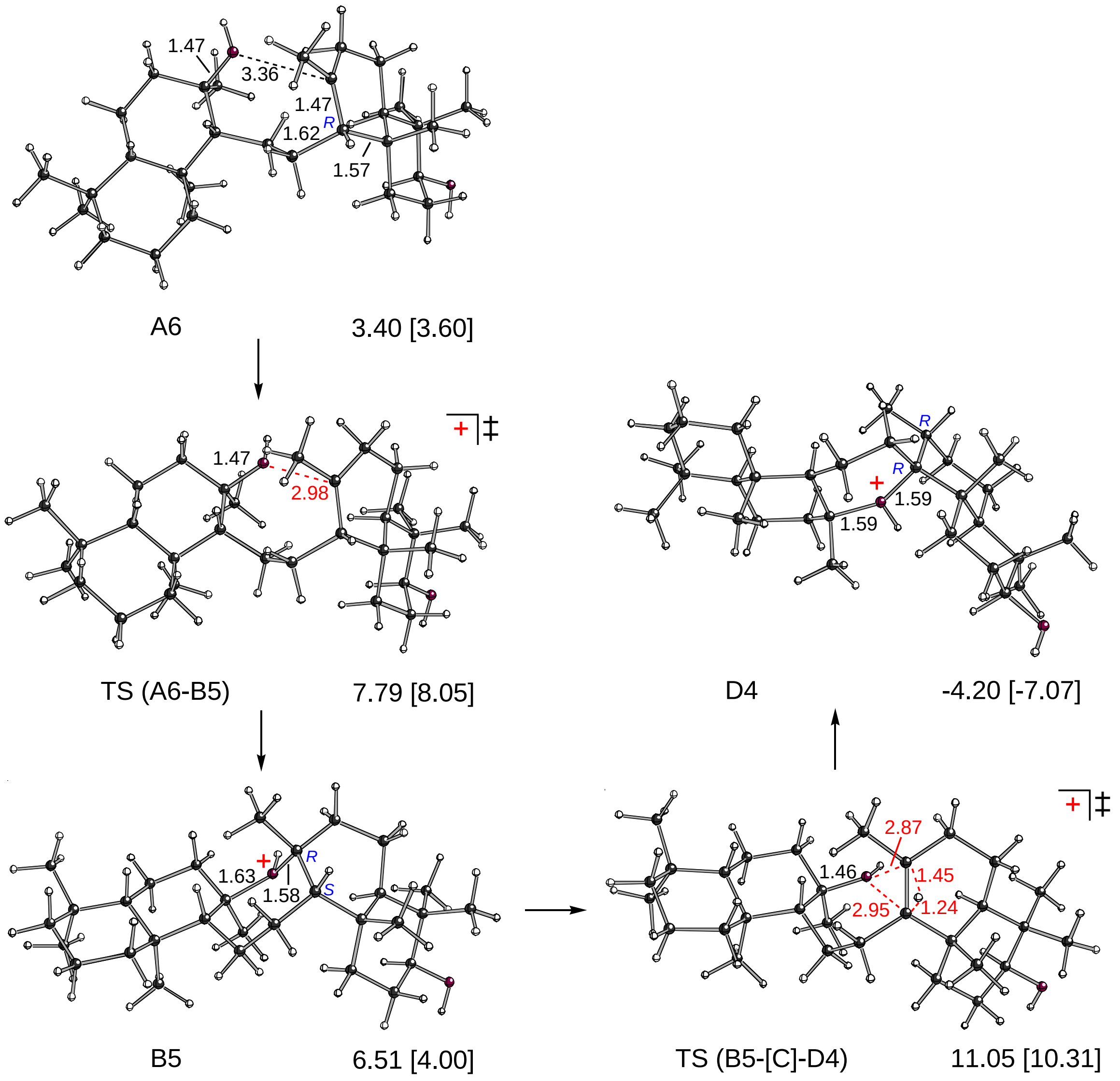

Figure S7 | Formation of **B5** and **D4**. Energies (kcal/mol; B3LYP/6-31G(d)//B3LYP/6-31G(d) in normal texts and mPW1PW91/6-31+G(d,p)//B3LYP/6-31G(d) in brackets) are shown. The **B5**→**D4** conversion is a concerted reaction consisting of asynchronously occurring ring opening/1,2-hydride shift/ring closure events, which is formally known as a dyotropic rearrangement.

Table S1 | Reaction energetics (kcal/mol; mPW1PW91/6-31+G(d,p)//B3LYP/6-31G(d)) for formation of **B** (6-6-7-6-6) and **D** (6-6-6-6-6). Isomers **B1** (10+H$^+$) and **D3** (21+H$^+$) are observed in experiments.

**A1**	**TS (A1-B1)**			**B1 (10+H$^+$)**	Figure S1
0.00	**0.49**			-7.19	
**A2**	**TS (A2-C1)**	**C1**	**TS (C1-D1)**	**D1**	Figure S2
1.52	1.90	3.05	**6.96**	-6.97	
**A3**	**TS (A3-B2)**			**B2**	Figure S3
4.42	**6.81**			-1.45	
**B3**	**TS (B3-C2)**	**C2**	**TS (C2-D2)**	**D2**	Figure S4
-1.86	**10.10**	5.58	5.70	-0.80	
**A4**	**TS (A4-B4)**			**B4**	Figure S5
4.89	**5.95**			2.79	
**A5**	**TS (A5-C3)**	**C3/C4**	**TS (C4-D3)**	**D3 (21+H$^+$)**	Figure S6
4.91	**3.80**	2.87/1.12	2.01	-7.42	
**A6**	**TS (A6-B5)**			**B5**	Figure S7
3.60	**8.05**			4.00	
**B5**	**TS (B5-[C]-D4)**			**D4**	Figure S7
4.00	**10.31**			-7.07	

Coordinates and Energies

Figure S1

A1

B3LYP/6-31G(d)//B3LYP/6-31G(d)

HF = -1325.3001832 hartrees (-831639.117959832 kcal/mol)

Imaginary Frequencies: none found

Zero-point correction = 0.789404 (Hartree/Particle)

mPW1PW91/6-31+G(d,p)//B3LYP/6-31G(d)

HF = -1325.1311583 hartrees (-831533.053144833 kcal/mol)

Coordinates (from last standard orientation):

Center Number	Atomic Number	Coordinates (Angstroms) X	Y	Z
1	6	3.088171	-1.684607	-0.658477
2	6	2.987497	-0.417589	0.223235
3	6	4.209967	0.510871	-0.084898
4	6	5.637672	-0.144175	0.045263
5	6	5.622366	-1.439063	-0.815795
6	6	4.452808	-2.375053	-0.496686
7	1	4.579010	-2.782824	0.512043
8	6	1.640025	0.425771	-0.306173
9	6	1.680118	1.630995	0.520983
10	6	2.754465	2.595250	0.236524
11	1	2.651890	3.520982	0.807811
12	6	4.094035	1.859433	0.633193
13	6	0.314522	-0.356383	-0.340033
14	1	0.501286	-1.318664	-0.820666
15	1	-0.021766	-0.594267	0.673181
16	6	-0.799506	0.412181	-1.117879
17	1	-0.992315	-0.114472	-2.057400
18	1	-0.406616	1.391015	-1.409327
19	6	-2.129072	0.640784	-0.341124
20	1	-1.884742	0.568800	0.728660
21	6	-2.632882	2.107056	-0.488839
22	6	-3.852311	2.328193	0.420801
23	1	-3.498909	2.298749	1.460220
24	6	-2.900297	2.595750	-1.919539
25	1	-3.056677	3.681834	-1.906854

26	1	-2.057633	2.391198	-2.586281
27	1	-3.796253	2.148421	-2.352190
28	6	-3.239466	-0.459559	-0.564538
29	6	-4.395338	-0.144912	0.456944
30	1	-3.887369	-0.114554	1.436264
31	6	-4.952461	1.274248	0.239089
32	1	-5.748625	1.484030	0.959329
33	1	-5.411146	1.371946	-0.751718
34	1	-4.256544	3.336633	0.251813
35	6	-2.629859	-1.838125	-0.189999
36	1	-1.864963	-2.122779	-0.924976
37	1	-2.117727	-1.747863	0.780091
38	6	-5.486269	-1.256221	0.648207
39	6	-4.764917	-2.597172	0.938827
40	1	-4.307391	-2.534803	1.937881
41	6	-3.672437	-2.957988	-0.071880
42	1	-4.116002	-3.175606	-1.050747
43	1	-3.172543	-3.883676	0.239480
44	6	-6.458592	-1.412246	-0.541315
45	1	-7.259619	-2.110279	-0.271053
46	1	-6.935962	-0.460452	-0.800477
47	1	-5.982162	-1.802586	-1.443221
48	6	-6.338359	-0.923690	1.895339
49	1	-7.005939	-0.071319	1.731879
50	1	-6.970910	-1.780690	2.154231
51	1	-5.710070	-0.701672	2.767102
52	6	-3.708842	-0.513770	-2.042705
53	1	-4.037123	-1.516857	-2.321890

54	1	-4.543118	0.157929	-2.254000
55	1	-2.900115	-0.252230	-2.732597
56	1	4.483324	-3.236719	-1.176750
57	8	-1.523658	2.915624	0.025469
58	1	-1.835503	3.834984	0.067875
59	1	-5.508827	-3.402284	0.993290
60	6	2.798693	-0.816379	1.698333
61	1	1.794383	-1.219796	1.863973
62	1	3.497199	-1.603869	1.981387
63	1	2.947773	0.010706	2.398754
64	8	6.869807	-2.091653	-0.619968
65	1	6.945858	-2.812520	-1.263996
66	1	5.525173	-1.121097	-1.869743
67	1	2.950044	-1.407148	-1.712462
68	1	2.293148	-2.393396	-0.407374
69	1	4.135352	1.749944	1.720876
70	1	4.901862	2.537877	0.350157
71	6	0.805257	1.848496	1.664662
72	1	-0.100712	2.334204	1.207345
73	1	1.215770	2.545704	2.399788
74	1	1.925581	0.688783	-1.330796
75	1	4.124567	0.729406	-1.161104
76	6	6.682400	0.809245	-0.583637
77	1	7.619992	0.266798	-0.727236
78	1	6.355552	1.182875	-1.562398
79	1	6.895302	1.670390	0.058101
80	6	6.087133	-0.439893	1.491472
81	1	6.044059	0.461881	2.111884

82	1	5.499983	-1.216430	1.985283
83	1	7.123615	-0.784098	1.480191
84	1	2.807005	2.824250	-0.833104
85	1	0.433253	0.933164	2.129325

TS (A1-B1)

B3LYP/6-31G(d)//B3LYP/6-31G(d)

HF = -1325.2998616 hartrees (-831638.916152616 kcal/mol)

Imaginary Frequencies: 1 (-20.2839 1/cm)

Zero-point correction = 0.789355 (Hartree/Particle)

mPW1PW91/6-31+G(d,p)//B3LYP/6-31G(d)

HF = -1325.1303754 hartrees (-831532.561867254 kcal/mol)

Coordinates (from last standard orientation):

Center Number	Atomic Number	Coordinates (Angstroms) X	Y	Z
1	6	3.206345	-1.687810	-0.759207
2	6	3.004444	-0.505091	0.217195
3	6	4.152062	0.534892	-0.008571
4	6	5.625295	-0.018030	0.089457
5	6	5.715682	-1.239239	-0.868800
6	6	4.619643	-2.282143	-0.634529
7	1	4.771198	-2.753717	0.342204
8	6	1.588874	0.274552	-0.265070

9	6	1.549348	1.402357	0.657480
10	6	2.539811	2.469784	0.446249
11	1	2.362342	3.339498	1.083718
12	6	3.930542	1.814755	0.803770
13	6	0.337619	-0.615151	-0.392448
14	1	0.640104	-1.536829	-0.893781
15	1	-0.035473	-0.920733	0.589161
16	6	-0.793958	0.062619	-1.224234
17	1	-1.072948	-0.621672	-2.030086
18	1	-0.374548	0.941192	-1.723503
19	6	-2.064701	0.487610	-0.429296
20	1	-1.806126	0.437856	0.637740
21	6	-2.412679	1.989615	-0.641081
22	6	-3.588070	2.383237	0.270185
23	1	-3.224887	2.365733	1.306535
24	6	-2.656757	2.437411	-2.089123
25	1	-2.689236	3.534027	-2.128778
26	1	-1.863266	2.106201	-2.764993
27	1	-3.609870	2.077801	-2.479797
28	6	-3.287457	-0.500928	-0.575633
29	6	-4.387077	-0.013899	0.438651
30	1	-3.862065	0.013630	1.409437
31	6	-4.795066	1.443054	0.151428
32	1	-5.556036	1.772762	0.864685
33	1	-5.253210	1.538276	-0.839928
34	1	-3.885803	3.419754	0.057389
35	6	-2.816100	-1.910647	-0.125479
36	1	-2.090446	-2.315731	-0.843261

37	1	-2.287961	-1.815243	0.836038
38	6	-5.586233	-0.992125	0.701457
39	6	-5.004545	-2.384556	1.058127
40	1	-4.534002	-2.319006	2.050917
41	6	-3.965763	-2.909786	0.063330
42	1	-4.439662	-3.137124	-0.899036
43	1	-3.558115	-3.862225	0.424396
44	6	-6.587194	-1.107601	-0.468667
45	1	-7.456370	-1.695144	-0.150424
46	1	-6.960065	-0.125206	-0.779717
47	1	-6.170060	-1.600323	-1.349598
48	6	-6.378751	-0.503411	1.935907
49	1	-6.957648	0.403208	1.731038
50	1	-7.091792	-1.274267	2.250416
51	1	-5.716268	-0.299370	2.786394
52	6	-3.783200	-0.585067	-2.043502
53	1	-4.229944	-1.557130	-2.260414
54	1	-4.537451	0.166561	-2.284651
55	1	-2.961025	-0.457527	-2.755110
56	1	4.719139	-3.085038	-1.376903
57	8	-1.214061	2.690618	-0.172713
58	1	-1.392944	3.642069	-0.260946
59	1	-5.827811	-3.103563	1.157913
60	6	2.835529	-1.034204	1.652632
61	1	1.861401	-1.520112	1.769932
62	1	3.587165	-1.790598	1.880113
63	1	2.918465	-0.258723	2.419585
64	8	7.008234	-1.808328	-0.708070

65	1	7.143906	-2.468780	-1.404934
66	1	5.601492	-0.849127	-1.896348
67	1	3.052923	-1.341785	-1.790391
68	1	2.469256	-2.474232	-0.571469
69	1	3.975361	1.629898	1.881311
70	1	4.685477	2.571086	0.578749
71	6	0.699662	1.460633	1.844908
72	1	-0.186125	2.021095	1.457714
73	1	1.133587	2.057901	2.652224
74	1	1.865096	0.636550	-1.261182
75	1	4.056527	0.825907	-1.066594
76	6	6.599188	1.058149	-0.449028
77	1	7.575494	0.599803	-0.624700
78	1	6.250622	1.484317	-1.398378
79	1	6.744624	1.877828	0.262122
80	6	6.084754	-0.390528	1.514383
81	1	5.972287	0.456172	2.200195
82	1	5.551299	-1.242107	1.940737
83	1	7.143406	-0.658198	1.489762
84	1	2.572853	2.779329	-0.603053
85	1	0.336261	0.496019	2.200853

B1 (10+H$^+$)

B3LYP/6-31G(d)//B3LYP/6-31G(d)

HF = -1325.3116578 hartrees (-831646.318386078 kcal/mol)

Imaginary Frequencies: none found

Zero-point correction = 0.792935 (Hartree/Particle)

mPW1PW91/6-31+G(d,p)//B3LYP/6-31G(d)

HF = -1325.1426158 hartrees (-831540.242840658 kcal/mol)

Coordinates (from last standard orientation):

Center Number	Atomic Number	Coordinates (Angstroms) X	Y	Z
1	6	3.408735	1.856594	0.504080
2	6	2.944387	0.630085	-0.333169
3	6	3.893928	-0.569593	0.030325
4	6	5.443036	-0.332396	-0.072478
5	6	5.769502	0.937428	0.757656
6	6	4.910799	2.143478	0.376624
7	1	5.171860	2.463308	-0.638202
8	6	1.473105	0.232399	0.155256
9	6	1.006946	-1.119971	-0.428217
10	6	2.017480	-2.239143	-0.160932
11	1	1.679169	-3.169034	-0.640064
12	6	3.425988	-1.866948	-0.646228
13	6	0.445852	1.376740	0.004961
14	1	0.939408	2.301110	0.311568
15	1	0.175296	1.527330	-1.046606
16	6	-0.844831	1.277320	0.843519
17	1	-1.293050	2.272956	0.839744
18	1	-0.582936	1.084687	1.890079
19	6	-1.890373	0.261589	0.326521

20	1	-1.740093	0.166279	-0.754906
21	6	-1.691301	-1.152432	0.889192
22	6	-2.582760	-2.170539	0.189504
23	1	-2.316656	-2.219193	-0.873171
24	6	-1.662459	-1.342965	2.401000
25	1	-1.350318	-2.363594	2.647231
26	1	-0.984346	-0.648416	2.898243
27	1	-2.660851	-1.202532	2.816126
28	6	-3.411230	0.735634	0.435402
29	6	-4.280307	-0.354980	-0.296473
30	1	-3.848388	-0.402402	-1.310616
31	6	-4.061286	-1.753607	0.305561
32	1	-4.655356	-2.499848	-0.228028
33	1	-4.384976	-1.798989	1.351415
34	1	-2.433613	-3.170813	0.619619
35	6	-3.555282	2.057775	-0.372391
36	1	-3.035890	2.878027	0.135871
37	1	-3.066762	1.935219	-1.350750
38	6	-5.785669	0.002637	-0.564211
39	6	-5.821292	1.368167	-1.296048
40	1	-5.423128	1.226773	-2.312001
41	6	-5.016174	2.470765	-0.605368
42	1	-5.490299	2.750932	0.343060
43	1	-5.030037	3.378371	-1.220559
44	6	-6.671284	0.035393	0.699894
45	1	-7.720196	0.157789	0.406758
46	1	-6.604429	-0.900740	1.266280
47	1	-6.430053	0.855362	1.379209

48	6	-6.394882	-1.043562	-1.526673
49	1	-6.574480	-2.009208	-1.041998
50	1	-7.363881	-0.687835	-1.894179
51	1	-5.753852	-1.212594	-2.400953
52	6	-3.808298	1.012667	1.907247
53	1	-4.597152	1.763747	1.968865
54	1	-4.180199	0.130958	2.435016
55	1	-2.959822	1.409142	2.474874
56	1	5.162879	2.988088	1.032313
57	8	-0.183581	-1.598166	0.529642
58	1	-0.183052	-2.573876	0.513445
59	1	-6.864884	1.684345	-1.418135
60	6	2.954672	1.026865	-1.830105
61	1	2.059595	1.598562	-2.094107
62	1	3.806375	1.667621	-2.061682
63	1	3.015921	0.173692	-2.510427
64	8	7.155626	1.216963	0.593596
65	1	7.393413	1.939926	1.194352
66	1	5.561393	0.691248	1.815092
67	1	3.169936	1.678432	1.563055
68	1	2.860963	2.757030	0.207058
69	1	3.446869	-1.785145	-1.738197
70	1	4.088937	-2.697546	-0.391907
71	6	0.479788	-1.161889	-1.855310
72	1	-0.004868	-2.122566	-2.057015
73	1	1.320372	-1.075341	-2.547295
74	1	1.585000	0.043336	1.232012
75	1	3.728201	-0.724412	1.110179

76	6	6.181885	-1.514219	0.602580
77	1	7.227083	-1.238930	0.763998
78	1	5.743400	-1.762006	1.577900
79	1	6.172666	-2.418039	-0.015311
80	6	5.984833	-0.211436	-1.513116
81	1	5.705809	-1.085462	-2.112343
82	1	5.636658	0.679857	-2.038361
83	1	7.075924	-0.162623	-1.486783
84	1	2.068214	-2.415606	0.920758
85	1	-0.219189	-0.358893	-2.088065

Figure S2

A2

B3LYP/6-31G(d)//B3LYP/6-31G(d)

HF = -1325.2962234 hartrees (-831636.633145734 kcal/mol)

Imaginary Frequencies: none found

Zero-point correction = 0.787463 (Hartree/Particle)

mPW1PW91/6-31+G(d,p)//B3LYP/6-31G(d)

HF = -1325.1287411 hartrees (-831531.536327661 kcal/mol)

Coordinates (from last standard orientation):

Center Number	Atomic Number	Coordinates (Angstroms) X	Y	Z
1	6	-2.758466	-0.755368	-1.438397

2	6	-2.824475	0.618397	-0.728126
3	6	-3.631027	0.446104	0.607055
4	6	-5.026988	-0.275552	0.521992
5	6	-4.836550	-1.595689	-0.273092
6	6	-4.140508	-1.393782	-1.620788
7	1	-4.779278	-0.795273	-2.279397
8	6	-1.344116	1.082068	-0.355442
9	6	-1.264225	2.165050	0.592246
10	6	-2.302998	2.351263	1.637932
11	1	-2.332198	3.409893	1.928698
12	6	-3.696831	1.774950	1.372721
13	6	-0.286394	1.020375	-1.493841
14	1	-0.821122	1.024078	-2.447779
15	1	0.326255	1.926240	-1.509334
16	6	0.642103	-0.217382	-1.472973
17	1	1.190218	-0.205994	-2.418972
18	1	0.020144	-1.113828	-1.513014
19	6	1.641494	-0.302070	-0.279498
20	1	1.518252	0.613495	0.317441
21	6	1.271492	-1.425714	0.734867
22	6	2.217325	-1.375249	1.945123
23	1	1.990938	-0.458473	2.505112
24	6	1.167145	-2.850558	0.176477
25	1	0.730014	-3.508377	0.939364
26	1	0.536838	-2.910620	-0.714571
27	1	2.145003	-3.264823	-0.072937
28	6	3.163426	-0.272945	-0.705921
29	6	4.014386	-0.190596	0.615437

30	1	3.623301	0.704886	1.129615
31	6	3.700828	-1.369072	1.555018
32	1	4.292751	-1.292387	2.471465
33	1	3.979516	-2.326274	1.098921
34	1	1.997909	-2.216676	2.616519
35	6	3.411069	1.039686	-1.501854
36	1	2.887304	1.013407	-2.465288
37	1	2.981833	1.881811	-0.934644
38	6	5.543637	0.124891	0.449430
39	6	5.681276	1.393914	-0.430173
40	1	5.327193	2.258142	0.152364
41	6	4.896706	1.338686	-1.743141
42	1	5.331540	0.590086	-2.416037
43	1	4.988490	2.296968	-2.269088
44	6	6.376325	-1.035645	-0.137114
45	1	7.442288	-0.785706	-0.082257
46	1	6.233716	-1.962076	0.430554
47	1	6.149337	-1.248187	-1.183948
48	6	6.145336	0.466182	1.832782
49	1	6.247816	-0.414963	2.474721
50	1	7.148786	0.889414	1.708938
51	1	5.537881	1.207777	2.366853
52	6	3.512861	-1.482161	-1.612303
53	1	4.332228	-1.248354	-2.294580
54	1	3.818569	-2.365518	-1.048099
55	1	2.662307	-1.772763	-2.237586
56	1	-4.019169	-2.366773	-2.115235
57	8	-0.067261	-1.037264	1.204538

58	1	-0.425695	-1.764690	1.739329
59	1	6.743754	1.578542	-0.633718
60	6	-3.394961	1.691088	-1.684907
61	1	-3.545583	2.654959	-1.186680
62	1	-2.718413	1.862617	-2.527959
63	1	-4.352702	1.389599	-2.108917
64	8	-6.118881	-2.190185	-0.428755
65	1	-6.004686	-3.063677	-0.833982
66	1	-4.193170	-2.252714	0.341771
67	1	-2.139131	-1.432780	-0.835441
68	1	-2.266980	-0.662249	-2.413189
69	1	-4.286042	2.512916	0.820457
70	1	-4.197615	1.645145	2.335170
71	6	-0.102387	3.083855	0.682647
72	1	-0.041917	3.582985	1.652616
73	1	-0.264940	3.863317	-0.082800
74	1	-0.989185	0.298769	0.409526
75	1	-3.013978	-0.236817	1.214655
76	6	-5.472439	-0.665001	1.952881
77	1	-6.314736	-1.358278	1.890629
78	1	-4.666458	-1.163010	2.507359
79	1	-5.802426	0.201214	2.535028
80	6	-6.158808	0.574702	-0.096546
81	1	-6.279056	1.524065	0.435673
82	1	-6.011703	0.799995	-1.154887
83	1	-7.101136	0.028775	-0.015840
84	1	-1.842287	1.855519	2.516742
85	1	0.849140	2.609694	0.435469

TS (A2-C1)

B3LYP/6-31G(d)//B3LYP/6-31G(d)

HF = -1325.2931782 hartrees (-831634.722252282 kcal/mol)

Imaginary Frequencies: 1 (-311.9691 1/cm)

Zero-point correction = 0.786609 (Hartree/Particle)

mPW1PW91/6-31+G(d,p)//B3LYP/6-31G(d)

HF = -1325.1281336 hartrees (-831531.155115336 kcal/mol)

Coordinates (from last standard orientation):

Center Number	Atomic Number	Coordinates (Angstroms) X	Y	Z
1	6	2.659911	-0.829568	1.422540
2	6	2.808383	0.591293	0.818590
3	6	3.604450	0.468773	-0.529885
4	6	4.954543	-0.337265	-0.502028
5	6	4.697321	-1.696615	0.201685
6	6	4.005476	-1.552456	1.558958
7	1	4.671534	-1.040110	2.261302
8	6	1.429916	1.259708	0.570990
9	6	1.258945	2.142754	-0.502655
10	6	2.347721	2.411484	-1.529768
11	1	2.391815	3.498883	-1.666790
12	6	3.728323	1.843106	-1.200678

13	6	0.322966	1.055694	1.604031
14	1	0.823083	0.955699	2.571167
15	1	-0.288120	1.958337	1.679021
16	6	-0.612369	-0.181659	1.447112
17	1	-1.159207	-0.235532	2.391321
18	1	-0.002211	-1.083816	1.417081
19	6	-1.620444	-0.160603	0.261103
20	1	-1.600581	0.845352	-0.175397
21	6	-1.172824	-1.070972	-0.919867
22	6	-2.126140	-0.894395	-2.109695
23	1	-1.995122	0.123232	-2.501146
24	6	-0.948538	-2.556780	-0.613281
25	1	-0.462282	-3.033536	-1.473705
26	1	-0.308932	-2.717170	0.258130
27	1	-1.888052	-3.084594	-0.445683
28	6	-3.133363	-0.342757	0.693132
29	6	-4.004217	-0.124211	-0.601487
30	1	-3.702816	0.876302	-0.957369
31	6	-3.597687	-1.094704	-1.724762
32	1	-4.208400	-0.921312	-2.614994
33	1	-3.777682	-2.136929	-1.436081
34	1	-1.840516	-1.585530	-2.915240
35	6	-3.489559	0.797458	1.689694
36	1	-2.962766	0.659935	2.642145
37	1	-3.138182	1.754420	1.272022
38	6	-5.553520	0.018882	-0.393378
39	6	-5.793779	1.113782	0.676653
40	1	-5.522473	2.087652	0.241882

41	6	-4.994535	0.919307	1.967268
42	1	-5.354643	0.036889	2.509858
43	1	-5.164919	1.769133	2.639382
44	6	-6.271134	-1.291624	-0.002694
45	1	-7.355627	-1.132408	-0.014905
46	1	-6.057464	-2.098045	-0.713489
47	1	-6.010335	-1.650043	0.995222
48	6	-6.199652	0.521298	-1.705580
49	1	-6.231436	-0.251169	-2.480988
50	1	-7.234994	0.826866	-1.516336
51	1	-5.668208	1.391069	-2.111861
52	6	-3.354733	-1.706710	1.395158
53	1	-4.198570	-1.669102	2.086095
54	1	-3.557104	-2.522916	0.698504
55	1	-2.481312	-1.994299	1.990595
56	1	3.826639	-2.550059	1.981391
57	8	0.142013	-0.518015	-1.305355
58	1	0.450113	-0.980646	-2.102647
59	1	-6.866277	1.168815	0.902347
60	6	3.464191	1.540830	1.865337
61	1	3.601773	2.555465	1.482520
62	1	2.853306	1.609897	2.770982
63	1	4.440536	1.164937	2.166749
64	8	5.948857	-2.360232	0.320184
65	1	5.790716	-3.258758	0.648618
66	1	4.025370	-2.279148	-0.456016
67	1	2.015569	-1.424164	0.763263
68	1	2.169857	-0.788887	2.401323

69	1	4.271129	2.538923	-0.554164
70	1	4.305039	1.775036	-2.126084
71	6	0.046417	3.027142	-0.699862
72	1	-0.217215	3.077414	-1.760793
73	1	0.327602	4.041290	-0.388938
74	1	0.894271	0.853724	-0.634930
75	1	2.946878	-0.139593	-1.174072
76	6	5.366235	-0.652543	-1.961319
77	1	6.161363	-1.402061	-1.954847
78	1	4.526625	-1.056227	-2.542035
79	1	5.749443	0.228729	-2.485031
80	6	6.138607	0.405734	0.157021
81	1	6.255316	1.412267	-0.257536
82	1	6.053487	0.495164	1.242399
83	1	7.062234	-0.143110	-0.039037
84	1	1.984248	2.019782	-2.490843
85	1	-0.834926	2.733637	-0.134998

C1

B3LYP/6-31G(d)//B3LYP/6-31G(d)

HF = -1325.2964621 hartrees (-831636.782932371 kcal/mol)

Imaginary Frequencies: none found

Zero-point correction = 0.789253 (Hartree/Particle)

mPW1PW91/6-31+G(d,p)//B3LYP/6-31G(d)

HF = -1325.1262987 hartrees (-831530.003697237 kcal/mol)

Coordinates (from last standard orientation):

Center Number	Atomic Number	Coordinates (Angstroms) X	Y	Z
1	6	-2.699340	-0.599742	-1.575606
2	6	-2.733304	0.694268	-0.721313
3	6	-3.458241	0.433881	0.649090
4	6	-4.801103	-0.396628	0.609601
5	6	-4.598945	-1.638785	-0.299569
6	6	-4.068776	-1.273425	-1.686717
7	1	-4.795742	-0.646617	-2.214481
8	6	-1.381880	1.300818	-0.516636
9	6	-1.094293	2.020695	0.736587
10	6	-2.312874	2.577384	1.510718
11	1	-2.501717	3.583679	1.117141
12	6	-3.603006	1.734121	1.458975
13	6	-0.341487	1.219019	-1.579880
14	1	-0.847985	1.195253	-2.549794
15	1	0.289323	2.109560	-1.550887
16	6	0.602233	-0.049160	-1.577200
17	1	1.179503	0.071552	-2.496753
18	1	-0.022089	-0.927428	-1.726415
19	6	1.547406	-0.240855	-0.361325
20	1	1.424534	0.624661	0.300230
21	6	1.094305	-1.432940	0.532202
22	6	1.975715	-1.500745	1.788392
23	1	1.748466	-0.622140	2.406704

24	6	1.005824	-2.811076	-0.139960
25	1	0.495150	-3.506947	0.537947
26	1	0.442132	-2.792913	-1.076925
27	1	1.989512	-3.234711	-0.346654
28	6	3.084875	-0.225162	-0.727445
29	6	3.870524	-0.277005	0.637293
30	1	3.480774	0.588799	1.200637
31	6	3.476337	-1.513129	1.466023
32	1	4.028827	-1.525774	2.409683
33	1	3.746820	-2.441075	0.948027
34	1	1.702084	-2.384659	2.381409
35	6	3.409961	1.136897	-1.402373
36	1	2.937159	1.200405	-2.391182
37	1	2.977482	1.946905	-0.793934
38	6	5.414322	0.000388	0.570090
39	6	5.630945	1.330130	-0.195943
40	1	5.270006	2.156002	0.435615
41	6	4.914740	1.404089	-1.547041
42	1	5.361788	0.696364	-2.255510
43	1	5.060341	2.397249	-1.989300
44	6	6.240673	-1.136245	-0.068593
45	1	7.309377	-0.918290	0.041059
46	1	6.056535	-2.096440	0.426538
47	1	6.046692	-1.268084	-1.135189
48	6	5.956020	0.213398	2.002868
49	1	6.001538	-0.717187	2.577820
50	1	6.976136	0.611793	1.958775
51	1	5.345399	0.931632	2.564277

52	6	3.438080	-1.368466	-1.714200
53	1	4.292130	-1.107556	-2.341806
54	1	3.692050	-2.305121	-1.214134
55	1	2.608023	-1.580535	-2.397477
56	1	-3.957842	-2.185176	-2.288221
57	8	-0.261993	-1.050792	0.928595
58	1	-0.593233	-1.743820	1.524139
59	1	6.707102	1.497858	-0.330855
60	6	-3.395554	1.872497	-1.556282
61	1	-3.331661	2.846308	-1.064251
62	1	-2.947489	1.952302	-2.549423
63	1	-4.452786	1.634810	-1.674415
64	8	-5.841870	-2.323330	-0.376866
65	1	-5.699809	-3.169427	-0.828587
66	1	-3.846186	-2.284836	0.189197
67	1	-2.012687	-1.298255	-1.085410
68	1	-2.302483	-0.397976	-2.576141
69	1	-4.408533	2.341039	1.037680
70	1	-3.915588	1.483948	2.475205
71	6	0.040744	3.060755	0.736625
72	1	0.159131	3.453426	1.750453
73	1	-0.208046	3.906015	0.085047
74	1	-0.735188	1.105309	1.281052
75	1	-2.752570	-0.211710	1.191253
76	6	-5.085559	-0.915749	2.040151
77	1	-5.898768	-1.644694	2.004505
78	1	-4.206732	-1.412918	2.470613
79	1	-5.387967	-0.113032	2.719424

80	6	-6.050099	0.392247	0.152360
81	1	-6.222754	1.270165	0.781060
82	1	-6.009865	0.724884	-0.888748
83	1	-6.927025	-0.252413	0.238979
84	1	-1.988580	2.724623	2.544974
85	1	1.006761	2.659339	0.427881

TS (C1-D1)

B3LYP/6-31G(d)//B3LYP/6-31G(d)

HF = -1325.2910615 hartrees (-831633.394001865 kcal/mol)

Imaginary Frequencies: 1 (-86.5045 1/cm)

Zero-point correction = 0.791042 (Hartree/Particle)

mPW1PW91/6-31+G(d,p)//B3LYP/6-31G(d)

HF = -1325.1200739 hartrees (-831526.097572989 kcal/mol)

Coordinates (from last standard orientation):

Center Number	Atomic Number	Coordinates (Angstroms) X	Y	Z
1	6	2.413072	-1.030218	1.175921
2	6	2.665061	0.427898	0.703897
3	6	3.635767	0.466737	-0.528104
4	6	4.892532	-0.492040	-0.510644
5	6	4.440028	-1.894878	-0.026408
6	6	3.702905	-1.841429	1.313734

7	1	4.360793	-1.439364	2.091237
8	6	1.364212	1.177223	0.366092
9	6	1.455336	2.201306	-0.758531
10	6	2.832877	2.914675	-0.730003
11	1	2.919585	3.515529	0.181408
12	6	4.009025	1.928396	-0.881455
13	6	0.321663	1.355443	1.452656
14	1	0.860720	1.440847	2.401019
15	1	-0.181898	2.310771	1.294305
16	6	-0.787748	0.285249	1.661507
17	1	-1.365043	0.648075	2.514754
18	1	-0.343138	-0.660752	1.978348
19	6	-1.707668	0.102199	0.437393
20	1	-1.755749	1.081874	-0.056752
21	6	-1.039105	-0.817481	-0.612193
22	6	-1.833048	-0.784179	-1.923506
23	1	-1.754106	0.223794	-2.348788
24	6	-0.733752	-2.262879	-0.204554
25	1	-0.025333	-2.704307	-0.918356
26	1	-0.311215	-2.356789	0.797332
27	1	-1.632850	-2.877816	-0.245530
28	6	-3.218447	-0.237918	0.736356
29	6	-3.954140	-0.168761	-0.656888
30	1	-3.729235	0.848161	-1.021689
31	6	-3.316638	-1.120196	-1.688153
32	1	-3.832513	-1.034864	-2.648443
33	1	-3.426300	-2.167884	-1.383196
34	1	-1.390445	-1.477396	-2.651294

35	6	-3.806936	0.889806	1.630551
36	1	-3.387202	0.835324	2.642724
37	1	-3.507514	1.865174	1.217346
38	6	-5.522719	-0.200644	-0.626944
39	6	-6.001309	0.902339	0.351019
40	1	-5.791154	1.882005	-0.103667
41	6	-5.338894	0.852244	1.729497
42	1	-5.663246	-0.041813	2.275930
43	1	-5.675694	1.704297	2.332271
44	6	-6.127513	-1.568879	-0.245151
45	1	-7.215268	-1.537905	-0.377027
46	1	-5.749392	-2.371824	-0.888288
47	1	-5.937626	-1.854830	0.791624
48	6	-6.068451	0.167889	-2.026211
49	1	-5.932390	-0.636676	-2.756546
50	1	-7.145012	0.363160	-1.964279
51	1	-5.591395	1.073096	-2.422335
52	6	-3.356367	-1.584339	1.490032
53	1	-4.271944	-1.617823	2.082664
54	1	-3.382007	-2.453428	0.829625
55	1	-2.528380	-1.726930	2.193775
56	1	3.441391	-2.860321	1.628823
57	8	0.261617	-0.124861	-0.862254
58	1	0.853913	-0.722680	-1.350764
59	1	-7.092133	0.842585	0.455681
60	6	3.263565	1.227842	1.924019
61	1	3.135999	2.309621	1.851168
62	1	2.812313	0.897758	2.862592

63	1	4.333388	1.035531	1.979512
64	8	5.591855	-2.722949	0.036213
65	1	5.309958	-3.630831	0.227014
66	1	3.735419	-2.293267	-0.781993
67	1	1.777712	-1.537549	0.446571
68	1	1.862656	-1.027843	2.122525
69	1	4.839332	2.266730	-0.256985
70	1	4.373602	1.953151	-1.912153
71	6	0.307240	3.208732	-0.885681
72	1	0.445882	3.782739	-1.806957
73	1	0.292185	3.926927	-0.058410
74	1	1.469277	1.609338	-1.680897
75	1	3.036959	0.081116	-1.368919
76	6	5.401788	-0.637502	-1.965288
77	1	6.165294	-1.417810	-2.008499
78	1	4.592290	-0.921007	-2.650424
79	1	5.850149	0.289582	-2.335589
80	6	6.092459	-0.012852	0.340974
81	1	6.446158	0.971715	0.022904
82	1	5.888993	0.029644	1.414390
83	1	6.918418	-0.714977	0.210135
84	1	2.830480	3.632402	-1.556200
85	1	-0.671241	2.725502	-0.946973

D1

B3LYP/6-31G(d)//B3LYP/6-31G(d)

HF = -1325.3101807 hartrees (-831645.391491057 kcal/mol)

Imaginary Frequencies: none found

Zero-point correction = 0.793255 (Hartree/Particle)

mPW1PW91/6-31+G(d,p)//B3LYP/6-31G(d)

HF = -1325.1422616 hartrees (-831540.020576616 kcal/mol)

Coordinates (from last standard orientation):

Center Number	Atomic Number	Coordinates (Angstroms) X	Y	Z
1	6	-2.806774	-0.547384	-1.641904
2	6	-2.698602	0.572846	-0.565177
3	6	-3.309722	0.039609	0.791482
4	6	-4.736303	-0.621568	0.718015
5	6	-4.710180	-1.691832	-0.406045
6	6	-4.214876	-1.149125	-1.748020
7	1	-4.936268	-0.420550	-2.131338
8	6	-1.189884	1.004921	-0.316646
9	6	-0.966456	1.945963	0.899845
10	6	-1.689353	1.428637	2.159818
11	1	-1.603099	2.184929	2.948171
12	6	-3.160619	1.079255	1.917246
13	6	-0.409338	1.409954	-1.571195
14	1	-0.652986	0.714546	-2.375734
15	1	-0.779887	2.381215	-1.905751
16	6	1.129182	1.510140	-1.412213
17	1	1.391279	2.509195	-1.052780

18	1	1.558236	1.433521	-2.416344
19	6	1.776525	0.481494	-0.454006
20	1	1.707138	0.906203	0.554280
21	6	0.979841	-0.824656	-0.349050
22	6	1.491385	-1.683542	0.806273
23	1	1.299514	-1.155338	1.747079
24	6	0.736840	-1.646890	-1.608954
25	1	-0.034105	-2.405945	-1.431254
26	1	0.451040	-1.052782	-2.477026
27	1	1.648733	-2.188853	-1.863761
28	6	3.327339	0.247725	-0.647233
29	6	3.793534	-0.609598	0.587456
30	1	3.470985	-0.019732	1.462014
31	6	3.008555	-1.933493	0.673830
32	1	3.319163	-2.506463	1.551672
33	1	3.211753	-2.575793	-0.190627
34	1	0.948207	-2.636798	0.845521
35	6	4.020491	1.635013	-0.560051
36	1	3.773671	2.236473	-1.443601
37	1	3.622496	2.176716	0.311360
38	6	5.342348	-0.747850	0.796938
39	6	5.947373	0.680621	0.790359
40	1	5.625603	1.196137	1.707469
41	6	5.545610	1.535889	-0.414763
42	1	5.992851	1.135431	-1.332587
43	1	5.957468	2.545891	-0.302708
44	6	6.054341	-1.646035	-0.237560
45	1	7.095657	-1.798577	0.068193

46	1	5.592267	-2.637945	-0.302521
47	1	6.074508	-1.220111	-1.242957
48	6	5.613833	-1.349534	2.195037
49	1	5.366078	-2.415105	2.250450
50	1	6.678266	-1.257729	2.438618
51	1	5.050968	-0.826285	2.978084
52	6	3.650806	-0.387007	-2.023759
53	1	4.642432	-0.092573	-2.370869
54	1	3.640437	-1.479133	-2.011554
55	1	2.947547	-0.058073	-2.795853
56	1	-4.190717	-1.963838	-2.484079
57	8	-0.435948	-0.329867	0.161055
58	1	-1.063661	-1.077151	0.151433
59	1	7.040365	0.606150	0.850040
60	6	-3.452000	1.815833	-1.122259
61	1	-2.835588	2.392860	-1.814867
62	1	-4.330550	1.500574	-1.683657
63	1	-3.799667	2.492305	-0.342783
64	8	-6.019391	-2.231062	-0.522172
65	1	-5.992350	-2.979238	-1.138414
66	1	-4.007486	-2.482985	-0.079360
67	1	-2.119207	-1.377346	-1.420535
68	1	-2.505292	-0.165299	-2.623166
69	1	-3.734881	1.983205	1.688156
70	1	-3.582645	0.682944	2.844274
71	6	-1.278904	3.435286	0.634853
72	1	-0.899154	4.014876	1.482252
73	1	-2.345669	3.647081	0.551326

74	1	0.106934	1.910908	1.106499
75	1	-2.670631	-0.810606	1.097892
76	6	-5.007231	-1.371500	2.045890
77	1	-5.879260	-2.018125	1.922277
78	1	-4.157471	-2.002913	2.336947
79	1	-5.218491	-0.686224	2.871985
80	6	-5.897157	0.375032	0.509052
81	1	-5.884980	1.153843	1.278419
82	1	-5.886805	0.865568	-0.465982
83	1	-6.847323	-0.157641	0.590176
84	1	-1.170298	0.537147	2.534035
85	1	-0.786058	3.819643	-0.263136

Figure S3

A3

B3LYP/6-31G(d)//B3LYP/6-31G(d) HF = -1325.2929549 hartrees (-831634.582129299 kcal/mol)

Imaginary Frequencies: none found

Zero-point correction = 0.789575 (Hartree/Particle)

mPW1PW91/6-31+G(d,p)//B3LYP/6-31G(d)

HF = -1325.124118 hartrees (-831528.63528618 kcal/mol)

Coordinates (from last standard orientation):

Center	Atomic	Coordinates (Angstroms)		
Number	Number	X	Y	Z

1	6	2.922860	-1.689957	-0.440161
2	6	2.977334	-0.346321	0.332521
3	6	4.291916	0.419871	-0.055259
4	6	5.639395	-0.381109	0.010131
5	6	5.431035	-1.695542	-0.793371
6	6	4.217213	-2.505120	-0.322358
7	1	4.387369	-2.854686	0.701737
8	6	1.813259	0.552794	-0.243050
9	6	2.023464	2.000088	-0.076071
10	6	2.954556	2.426038	0.969487
11	1	2.610544	1.995733	1.919120
12	6	4.370703	1.789366	0.657158
13	6	0.350310	0.267603	0.331934
14	1	0.383431	-0.749577	0.732993
15	1	0.140045	0.930694	1.176230
16	6	-0.772821	0.372373	-0.714722
17	1	-0.746169	-0.529478	-1.334276
18	1	-0.574369	1.198975	-1.399549
19	6	-2.165389	0.567125	-0.050444
20	1	-2.022700	0.398998	1.026283
21	6	-2.619784	2.056506	-0.118541
22	6	-3.893280	2.231826	0.720772
23	1	-3.626465	2.081356	1.775274
24	6	-2.807205	2.667635	-1.516002
25	1	-2.913489	3.754847	-1.416371
26	1	-1.956600	2.479910	-2.177762
27	1	-3.705173	2.301334	-2.015022
28	6	-3.261255	-0.486829	-0.463025

29	6	-4.506578	-0.216216	0.461634
30	1	-4.097923	-0.295291	1.483568
31	6	-4.998936	1.237771	0.334137
32	1	-5.853462	1.405700	0.995505
33	1	-5.359048	1.447019	-0.680282
34	1	-4.258702	3.264770	0.626433
35	6	-2.723991	-1.904483	-0.115877
36	1	-1.891819	-2.173671	-0.779899
37	1	-2.317615	-1.887178	0.907240
38	6	-5.644862	-1.296777	0.441516
39	6	-4.995710	-2.678833	0.708303
40	1	-4.662186	-2.706683	1.756511
41	6	-3.798261	-2.999175	-0.190500
42	1	-4.127705	-3.140526	-1.227290
43	1	-3.355900	-3.956274	0.112393
44	6	-6.477266	-1.327497	-0.858461
45	1	-7.329686	-2.004907	-0.730903
46	1	-6.885455	-0.339516	-1.100004
47	1	-5.915492	-1.677545	-1.727202
48	6	-6.624940	-1.026988	1.607037
49	1	-7.255676	-0.149564	1.430296
50	1	-7.296779	-1.883727	1.734285
51	1	-6.094002	-0.879926	2.555820
52	6	-3.557312	-0.427050	-1.983417
53	1	-3.900149	-1.391181	-2.363054
54	1	-4.325177	0.303494	-2.245981
55	1	-2.655866	-0.172690	-2.552245
56	1	4.123725	-3.408069	-0.939821

57	8	-1.522936	2.787013	0.527578
58	1	-1.864255	3.671944	0.741607
59	1	-5.759084	-3.461455	0.610403
60	6	2.856831	-0.629978	1.852713
61	1	1.816758	-0.742763	2.169933
62	1	3.356093	-1.566430	2.107050
63	1	3.311768	0.138704	2.483633
64	8	6.630856	-2.451279	-0.693796
65	1	6.567010	-3.209121	-1.295133
66	1	5.258437	-1.405536	-1.846125
67	1	2.750125	-1.483349	-1.506405
68	1	2.067677	-2.286882	-0.097830
69	1	4.889007	1.720564	1.615969
70	1	4.922390	2.503599	0.041979
71	1	1.732568	0.352679	-1.319700
72	1	4.162813	0.639518	-1.128988
73	6	6.735848	0.442951	-0.705150
74	1	7.635763	-0.165732	-0.820442
75	1	6.414139	0.759967	-1.705605
76	1	7.009311	1.338237	-0.136083
77	6	6.145187	-0.695211	1.435140
78	1	6.335244	0.219077	2.007661
79	1	5.461904	-1.317210	2.016135
80	1	7.091929	-1.235023	1.363821
81	1	3.016235	3.510431	1.077198
82	6	1.319571	2.985865	-0.895804
83	1	0.303041	3.074566	-0.427430
84	1	1.782973	3.974932	-0.863965

85	1	1.145932	2.656731	-1.923574

TS (A3-B2)

B3LYP/6-31G(d)//B3LYP/6-31G(d)

HF = -1325.2898625 hartrees (-831632.641617375 kcal/mol)

Imaginary Frequencies: 1 (-47.7848 1/cm)

Zero-point correction = 0.789374 (Hartree/Particle)

mPW1PW91/6-31+G(d,p)//B3LYP/6-31G(d)

HF = -1325.1203105 hartrees (-831526.246041855 kcal/mol)

Coordinates (from last standard orientation):

Center Number	Atomic Number	Coordinates (Angstroms) X	Y	Z
1	6	3.374040	-1.875824	-0.655840
2	6	3.014158	-0.603785	0.151243
3	6	4.182914	0.438125	0.021628
4	6	5.648570	-0.068488	0.280324
5	6	5.848497	-1.359762	-0.562630
6	6	4.768884	-2.419857	-0.318198
7	1	4.828099	-2.777691	0.715164
8	6	1.771186	0.073419	-0.597303
9	6	1.775659	1.535900	-0.496973
10	6	2.316973	2.137142	0.722507
11	1	1.809525	1.694025	1.585025

12	6	3.852925	1.739334	0.782211
13	6	0.360916	-0.460668	-0.092619
14	1	0.491080	-1.544297	-0.013117
15	1	0.200470	-0.073359	0.913524
16	6	-0.857720	-0.145155	-0.990261
17	1	-1.064395	-1.019250	-1.612348
18	1	-0.627211	0.657810	-1.693548
19	6	-2.104664	0.242769	-0.144155
20	1	-1.907531	-0.119439	0.874319
21	6	-2.197399	1.785971	0.035258
22	6	-3.293592	2.104087	1.062968
23	1	-2.957211	1.736201	2.041464
24	6	-2.395762	2.629462	-1.234647
25	1	-2.210184	3.683440	-0.991672
26	1	-1.712268	2.349578	-2.041067
27	1	-3.412251	2.566552	-1.624756
28	6	-3.453101	-0.468400	-0.539752
29	6	-4.495368	-0.079111	0.576861
30	1	-4.011173	-0.408032	1.512276
31	6	-4.638527	1.448630	0.711265
32	1	-5.351784	1.691826	1.503820
33	1	-5.047721	1.890061	-0.205434
34	1	-3.412112	3.193901	1.152892
35	6	-3.235838	-2.005425	-0.452540
36	1	-2.571619	-2.345990	-1.256886
37	1	-2.726182	-2.238052	0.495078
38	6	-5.852968	-0.865910	0.577663
39	6	-5.526903	-2.381331	0.574999

40	1	-5.101638	-2.644233	1.555172
41	6	-4.540689	-2.812309	-0.513438
42	1	-5.002415	-2.717605	-1.504117
43	1	-4.306672	-3.877790	-0.397766
44	6	-6.800795	-0.509952	-0.588064
45	1	-7.770987	-0.996382	-0.433166
46	1	-6.987270	0.568689	-0.642275
47	1	-6.430967	-0.833611	-1.563194
48	6	-6.614051	-0.571558	1.891428
49	1	-7.036397	0.438511	1.914361
50	1	-7.451394	-1.270516	2.000203
51	1	-5.967953	-0.691336	2.770066
52	6	-3.879269	-0.112838	-1.986191
53	1	-4.498499	-0.896897	-2.425770
54	1	-4.452729	0.814002	-2.054437
55	1	-3.003553	-0.006811	-2.636814
56	1	4.970315	-3.291745	-0.954324
57	8	-0.891372	2.155051	0.593992
58	1	-0.990657	3.052860	0.953085
59	1	-6.460668	-2.951181	0.484623
60	6	2.703452	-0.999040	1.610834
61	1	1.818555	-1.637197	1.672600
62	1	3.527773	-1.573801	2.031855
63	1	2.542620	-0.146176	2.276114
64	8	7.146509	-1.860303	-0.270458
65	1	7.340003	-2.587446	-0.881991
66	1	5.793624	-1.062166	-1.626094
67	1	3.357409	-1.638137	-1.729165

68	1	2.613966	-2.650743	-0.496392
69	1	4.113605	1.664454	1.839953
70	1	4.420650	2.573671	0.363635
71	1	1.818329	-0.189488	-1.661941
72	1	4.184718	0.697026	-1.052105
73	6	6.635057	0.998053	-0.254606
74	1	7.645793	0.583391	-0.268616
75	1	6.383800	1.305188	-1.278145
76	1	6.651536	1.893311	0.376227
77	6	6.011000	-0.327178	1.759762
78	1	5.862014	0.567094	2.374019
79	1	5.449495	-1.144530	2.216083
80	1	7.068037	-0.593973	1.822586
81	1	2.190099	3.220838	0.757521
82	6	1.464430	2.398003	-1.651581
83	1	1.030027	1.867389	-2.499090
84	1	0.840059	3.244078	-1.349846
85	1	2.428664	2.830178	-1.976785

B2

B3LYP/6-31G(d)//B3LYP/6-31G(d)

HF = -1325.3020195 hartrees (-831640.270256445 kcal/mol)

Imaginary Frequencies: none found

Zero-point correction = 0.793403 (Hartree/Particle)

mPW1PW91/6-31+G(d,p)//B3LYP/6-31G(d)

HF = -1325.1334742 hartrees (-831534.506395242 kcal/mol)

Coordinates (from last standard orientation):

Center Number	Atomic Number	Coordinates (Angstroms) X	Y	Z
1	6	3.480131	1.821521	0.714942
2	6	2.953457	0.699213	-0.221929
3	6	3.929279	-0.517103	-0.128106
4	6	5.476930	-0.246168	-0.269767
5	6	5.836695	0.912304	0.698826
6	6	4.960951	2.152893	0.499022
7	1	5.141294	2.577651	-0.494248
8	6	1.519596	0.250388	0.358965
9	6	1.231266	-1.262424	0.133150
10	6	1.907584	-1.809046	-1.120124
11	1	1.537887	-1.254321	-1.986307
12	6	3.449451	-1.695665	-1.009814
13	6	0.414951	1.226756	-0.111648
14	1	0.850205	2.225723	-0.022161
15	1	0.211576	1.085550	-1.179190
16	6	-0.924545	1.261907	0.657752
17	1	-1.309014	2.279559	0.562874
18	1	-0.749674	1.118715	1.730811
19	6	-1.999889	0.299278	0.115490
20	1	-1.911324	0.329263	-0.978942
21	6	-1.715791	-1.162014	0.485769
22	6	-2.645111	-2.123039	-0.250121

23	1	-2.460350	-2.047028	-1.329972
24	6	-1.565437	-1.522989	1.951997
25	1	-1.192715	-2.543450	2.068473
26	1	-0.920417	-0.838406	2.503312
27	1	-2.551059	-1.486142	2.418882
28	6	-3.511130	0.716866	0.389355
29	6	-4.403644	-0.302106	-0.414857
30	1	-4.032739	-0.220082	-1.450683
31	6	-4.119542	-1.754529	0.006890
32	1	-4.732741	-2.450440	-0.571146
33	1	-4.375059	-1.927354	1.058230
34	1	-2.449122	-3.159075	0.058786
35	6	-3.737053	2.124866	-0.231872
36	1	-3.207670	2.888349	0.349362
37	1	-3.304624	2.141428	-1.243257
38	6	-5.929157	0.041413	-0.550417
39	6	-6.039802	1.486159	-1.099688
40	1	-5.702010	1.484794	-2.146575
41	6	-5.219019	2.517704	-0.323450
42	1	-5.638426	2.661133	0.679723
43	1	-5.293273	3.494514	-0.816040
44	6	-6.739017	-0.113100	0.754778
45	1	-7.805400	0.021166	0.540792
46	1	-6.624766	-1.113146	1.189153
47	1	-6.470967	0.615910	1.522112
48	6	-6.569838	-0.893007	-1.603549
49	1	-6.690394	-1.919411	-1.240366
50	1	-7.570298	-0.528432	-1.862005

51	1	-5.982075	-0.923770	-2.529535
52	6	-3.816074	0.797927	1.906562
53	1	-4.630204	1.495637	2.108709
54	1	-4.113158	-0.156718	2.348111
55	1	-2.948220	1.168218	2.462338
56	1	5.261308	2.925459	1.219867
57	8	-0.301103	-1.450963	-0.249595
58	1	-0.364605	-2.352565	-0.618089
59	1	-7.095881	1.783029	-1.119823
60	6	2.846715	1.255762	-1.662582
61	1	2.212433	2.144855	-1.706301
62	1	3.825598	1.555939	-2.032737
63	1	2.449069	0.533428	-2.380741
64	8	7.217012	1.208759	0.519447
65	1	7.476810	1.860958	1.188209
66	1	5.666621	0.542065	1.727004
67	1	3.355183	1.499100	1.758794
68	1	2.875476	2.729371	0.598810
69	1	3.842855	-1.603322	-2.025496
70	1	3.851311	-2.632011	-0.612423
71	1	1.594599	0.372366	1.446775
72	1	3.844477	-0.842760	0.918987
73	6	6.231923	-1.510524	0.208321
74	1	7.292645	-1.277147	0.329080
75	1	5.854679	-1.868069	1.175605
76	1	6.151520	-2.331371	-0.512356
77	6	5.989893	0.073721	-1.692847
78	1	5.641353	-0.660096	-2.426579

79	1	5.710059	1.066737	-2.050752
80	1	7.081729	0.041810	-1.691775
81	1	1.632652	-2.863085	-1.271315
82	6	1.489957	-2.151983	1.346227
83	1	1.074316	-1.740921	2.266195
84	1	1.093829	-3.162279	1.192506
85	1	2.567038	-2.253578	1.492847

Figure S4

TS (B2-B3)

B3LYP/6-31G(d)//B3LYP/6-31G(d)

HF = -1325.2907561 hartrees (-831633.202360311 kcal/mol)

Imaginary Frequencies: 1 (-129.4894 1/cm)

Zero-point correction = 0.793808 (Hartree/Particle)

mPW1PW91/6-31+G(d,p)//B3LYP/6-31G(d)

HF = -1325.1221209 hartrees (-831527.382085959 kcal/mol)

Coordinates (from last standard orientation):

Center Number	Atomic Number	Coordinates (Angstroms) X	Y	Z
1	6	3.095294	-1.803146	-0.802485
2	6	2.664913	-0.514738	-0.048138
3	6	3.899434	0.448843	0.069618
4	6	5.294642	-0.156807	0.494840

5	6	5.541133	-1.436428	-0.346210
6	6	4.382667	-2.428787	-0.262081
7	1	4.262890	-2.774544	0.770788
8	6	1.612787	0.237532	-0.951273
9	6	1.243782	1.670000	-0.480523
10	6	2.186208	2.353700	0.561165
11	1	1.644835	2.503177	1.505094
12	6	3.527573	1.663827	0.927291
13	6	0.450882	-0.711544	-1.399023
14	1	0.841977	-1.271797	-2.253115
15	1	0.290995	-1.459177	-0.616352
16	6	-0.952292	-0.199246	-1.790017
17	1	-1.404720	-0.998764	-2.380449
18	1	-0.905540	0.655841	-2.470735
19	6	-1.853867	0.083286	-0.566041
20	1	-1.471422	-0.548109	0.246721
21	6	-1.694523	1.510585	-0.039475
22	6	-2.351022	1.670550	1.329777
23	1	-1.845994	1.015307	2.049691
24	6	-2.011228	2.689947	-0.948481
25	1	-1.567987	3.609679	-0.553937
26	1	-1.687136	2.555435	-1.978901
27	1	-3.091408	2.843502	-0.960921
28	6	-3.376147	-0.350965	-0.692616
29	6	-4.004266	-0.170720	0.740644
30	1	-3.361123	-0.788426	1.389793
31	6	-3.839846	1.269596	1.257799
32	1	-4.248680	1.365421	2.266918

33	1	-4.393262	1.984134	0.638345
34	1	-2.254332	2.706166	1.685731
35	6	-3.414941	-1.869421	-1.026495
36	1	-3.062631	-2.047098	-2.049155
37	1	-2.720870	-2.399922	-0.357660
38	6	-5.433838	-0.777684	0.966596
39	6	-5.389811	-2.263023	0.524778
40	1	-4.777927	-2.820258	1.249666
41	6	-4.812403	-2.487353	-0.874439
42	1	-5.491709	-2.086040	-1.636126
43	1	-4.745300	-3.562859	-1.077099
44	6	-6.572299	-0.025286	0.244185
45	1	-7.538519	-0.426229	0.570830
46	1	-6.572459	1.043953	0.486091
47	1	-6.536714	-0.121473	-0.843048
48	6	-5.760020	-0.764609	2.478334
49	1	-5.985278	0.239788	2.852853
50	1	-6.645237	-1.380727	2.671517
51	1	-4.936621	-1.176177	3.075426
52	6	-4.089227	0.420505	-1.830614
53	1	-4.936149	-0.144687	-2.221712
54	1	-4.481138	1.393108	-1.523707
55	1	-3.410062	0.584044	-2.674713
56	1	4.623252	-3.320241	-0.857230
57	8	-0.130766	1.596327	0.341321
58	1	-0.072945	2.293973	1.019737
59	1	-6.399852	-2.686960	0.587335
60	6	2.049626	-0.894418	1.317230

61	1	1.706221	-0.025141	1.886737
62	1	1.188411	-1.558175	1.189832
63	1	2.763274	-1.427906	1.946512
64	8	6.765616	-2.014984	0.093202
65	1	6.982768	-2.750303	-0.500169
66	1	5.638409	-1.119651	-1.401081
67	1	3.256185	-1.556770	-1.863178
68	1	2.290236	-2.545167	-0.777342
69	1	3.481311	1.356266	1.974196
70	1	4.318220	2.415258	0.867377
71	6	1.064255	2.611559	-1.669924
72	1	2.060859	2.780390	-2.092168
73	1	0.672520	3.586777	-1.368480
74	1	2.167066	0.456782	-1.873899
75	1	4.072198	0.808804	-0.957900
76	6	6.407331	0.855341	0.129266
77	1	7.384207	0.380579	0.249358
78	1	6.321693	1.187949	-0.913159
79	1	6.389407	1.742265	0.771206
80	6	5.435601	-0.474208	2.000814
81	1	5.318835	0.426508	2.611957
82	1	4.721279	-1.218643	2.361537
83	1	6.435243	-0.871557	2.189288
84	1	2.387945	3.362479	0.188771
85	1	0.443607	2.197165	-2.464269

B3

B3LYP/6-31G(d)//B3LYP/6-31G(d)

HF = -1325.3024083 hartrees (-831640.514232333 kcal/mol)

Imaginary Frequencies: none found

Zero-point correction = 0.794067 (Hartree/Particle)

mPW1PW91/6-31+G(d,p)//B3LYP/6-31G(d)

HF = -1325.134127 hartrees (-831534.91603377 kcal/mol)

Coordinates (from last standard orientation):

Center Number	Atomic Number	Coordinates (Angstroms) X	Y	Z
1	6	-3.001500	-1.068621	1.738510
2	6	-2.383536	-0.257565	0.563335
3	6	-3.578801	0.298264	-0.292730
4	6	-4.689842	-0.711310	-0.764356
5	6	-5.159229	-1.510590	0.480093
6	6	-4.007513	-2.129218	1.274777
7	1	-3.523303	-2.905792	0.671923
8	6	-1.615039	0.986743	1.193543
9	6	-1.300371	2.159580	0.241158
10	6	-2.404589	2.442525	-0.789044
11	1	-2.020050	3.142501	-1.548780
12	6	-3.054054	1.209560	-1.411954
13	6	-0.458675	0.589720	2.183344
14	1	-0.720362	0.973159	3.175741
15	1	-0.481214	-0.497138	2.290342

16	6	1.024298	0.974041	1.953329
17	1	1.564772	0.494052	2.772519
18	1	1.183776	2.042034	2.120342
19	6	1.648533	0.531027	0.610590
20	1	1.071840	-0.325608	0.249441
21	6	1.496039	1.590561	-0.491777
22	6	1.919059	1.050082	-1.855877
23	1	1.252997	0.226664	-2.138618
24	6	2.028267	2.999403	-0.261766
25	1	1.587001	3.698255	-0.980688
26	1	1.859209	3.381239	0.743533
27	1	3.104806	3.004987	-0.441111
28	6	3.137628	-0.026724	0.705616
29	6	3.504827	-0.572579	-0.724856
30	1	2.708143	-1.304082	-0.942015
31	6	3.363375	0.516300	-1.801681
32	1	3.595663	0.111326	-2.789846
33	1	4.068571	1.339260	-1.639736
34	1	1.830801	1.833373	-2.622077
35	6	3.126584	-1.243225	1.674686
36	1	2.955135	-0.914216	2.705793
37	1	2.284265	-1.899813	1.409997
38	6	4.818066	-1.424797	-0.842767
39	6	4.744987	-2.553150	0.216706
40	1	3.968946	-3.268328	-0.093977
41	6	4.422070	-2.067074	1.631058
42	1	5.258666	-1.483656	2.034248
43	1	4.313399	-2.928096	2.301199

44	6	6.124574	-0.615693	-0.691838
45	1	6.980271	-1.259480	-0.925095
46	1	6.163142	0.230455	-1.387757
47	1	6.282680	-0.225473	0.315694
48	6	4.858439	-2.108121	-2.229927
49	1	5.083658	-1.405930	-3.039748
50	1	5.644704	-2.871102	-2.243781
51	1	3.910642	-2.608957	-2.463895
52	6	4.100734	1.048311	1.265598
53	1	4.971074	0.594761	1.741416
54	1	4.484242	1.730436	0.503199
55	1	3.608740	1.651899	2.035989
56	1	-4.415033	-2.641561	2.156896
57	8	-0.095482	1.728653	-0.693621
58	1	-0.220623	2.188961	-1.545292
59	1	5.690909	-3.109137	0.216333
60	6	-1.434584	-1.178061	-0.229863
61	1	-0.985596	-0.687801	-1.097025
62	1	-0.622236	-1.539085	0.409133
63	1	-1.953548	-2.063141	-0.593588
64	8	-6.074969	-2.504464	0.031840
65	1	-6.468022	-2.928295	0.810347
66	1	-5.680445	-0.796299	1.143563
67	1	-3.515667	-0.372883	2.418517
68	1	-2.219337	-1.554662	2.331917
69	1	-2.350628	0.676170	-2.061581
70	1	-3.869311	1.547410	-2.056961
71	6	-0.950775	3.461113	0.960728

72	1	-1.851089	3.802690	1.480929
73	1	-0.669587	4.245140	0.250950
74	1	-2.381951	1.456482	1.826786
75	1	-4.119848	0.962570	0.402934
76	6	-5.911125	0.096432	-1.269797
77	1	-6.769166	-0.573798	-1.364886
78	1	-6.184509	0.898678	-0.572528
79	1	-5.735299	0.544580	-2.253303
80	6	-4.271133	-1.662521	-1.908373
81	1	-3.809001	-1.108443	-2.733127
82	1	-3.578024	-2.447185	-1.598496
83	1	-5.156824	-2.166333	-2.302176
84	1	-3.176387	3.011305	-0.256277
85	1	-0.167669	3.357903	1.708152

TS (B3-C2)

B3LYP/6-31G(d)//B3LYP/6-31G(d)

HF = -1325.2805712 hartrees (-831626.811233712 kcal/mol)

Imaginary Frequencies: 1 (-454.5552 1/cm)

Zero-point correction = 0.787672 (Hartree/Particle)

mPW1PW91/6-31+G(d,p)//B3LYP/6-31G(d)

HF = -1325.1150602 hartrees (-831522.951426102 kcal/mol)

Coordinates (from last standard orientation):

Center Atomic Coordinates (Angstroms)

Number	Number	X	Y	Z
1	6	2.607434	-1.278025	-1.475053
2	6	2.337990	-0.205168	-0.381441
3	6	3.730035	0.272498	0.166881
4	6	4.742977	-0.829324	0.647701
5	6	4.860675	-1.890051	-0.478907
6	6	3.504781	-2.410707	-0.962011
7	1	3.014466	-2.966685	-0.155465
8	6	1.584685	1.006287	-1.009776
9	6	1.947066	2.333509	-0.660793
10	6	2.966500	2.637740	0.412938
11	1	2.451542	3.331766	1.091506
12	6	3.521764	1.424728	1.158320
13	6	0.388589	0.747994	-1.942244
14	1	0.664978	1.090579	-2.948485
15	1	0.269090	-0.331035	-2.036850
16	6	-0.993600	1.367889	-1.595735
17	1	-1.600962	1.183348	-2.486790
18	1	-0.909755	2.451191	-1.533824
19	6	-1.721559	0.815227	-0.336919
20	1	-1.059357	0.078226	0.126604
21	6	-1.873286	1.896428	0.773651
22	6	-2.477867	1.257641	2.034879
23	1	-1.727793	0.575634	2.457267
24	6	-2.636751	3.174730	0.394261
25	1	-2.503690	3.920535	1.188041
26	1	-2.276507	3.619731	-0.538101

27	1	-3.709521	3.004531	0.291457
28	6	-3.038315	0.001736	-0.658504
29	6	-3.528183	-0.619947	0.702487
30	1	-2.656154	-1.192677	1.063219
31	6	-3.766927	0.471868	1.760831
32	1	-4.099269	0.022645	2.701057
33	1	-4.569986	1.152892	1.454878
34	1	-2.657803	2.037951	2.788534
35	6	-2.657151	-1.175211	-1.599907
36	1	-2.369038	-0.798151	-2.590010
37	1	-1.772089	-1.683960	-1.185915
38	6	-4.661095	-1.702458	0.613340
39	6	-4.217742	-2.779117	-0.410352
40	1	-3.379865	-3.344484	0.025008
41	6	-3.772434	-2.217249	-1.763078
42	1	-4.626665	-1.782161	-2.295861
43	1	-3.410235	-3.032818	-2.401169
44	6	-6.053444	-1.145730	0.245340
45	1	-6.802237	-1.940876	0.339665
46	1	-6.357465	-0.336896	0.919347
47	1	-6.113239	-0.767223	-0.777260
48	6	-4.798026	-2.410698	1.981300
49	1	-5.267776	-1.774383	2.738367
50	1	-5.427889	-3.301678	1.877091
51	1	-3.824438	-2.737855	2.367429
52	6	-4.095304	0.887858	-1.367568
53	1	-4.746312	0.297422	-2.014942
54	1	-4.746243	1.418276	-0.670001

55	1	-3.623766	1.641464	-2.007230
56	1	3.662639	-3.129436	-1.776627
57	8	-0.492954	2.286932	1.070389
58	1	-0.531086	2.827773	1.877076
59	1	-5.032414	-3.500833	-0.551920
60	6	1.390571	-0.774266	0.706439
61	1	1.007419	0.005150	1.369136
62	1	0.532280	-1.270054	0.244043
63	1	1.903292	-1.518275	1.312714
64	8	5.676271	-2.944557	0.013901
65	1	5.887380	-3.541036	-0.720893
66	1	5.357556	-1.399271	-1.335761
67	1	3.096216	-0.800865	-2.338215
68	1	1.671525	-1.707924	-1.843873
69	1	2.835646	1.128129	1.956808
70	1	4.458823	1.711333	1.640485
71	6	1.337168	3.558730	-1.292154
72	1	2.076815	4.362966	-1.342618
73	1	0.523955	3.879725	-0.633094
74	1	2.512618	1.618268	-1.665818
75	1	4.239808	0.709166	-0.711966
76	6	6.135943	-0.170767	0.803594
77	1	6.892153	-0.955073	0.889478
78	1	6.392623	0.453016	-0.062267
79	1	6.203404	0.449460	1.703050
80	6	4.399526	-1.495748	1.999449
81	1	4.118209	-0.750440	2.751167
82	1	3.598566	-2.235952	1.935120

83	1	5.277864	-2.027621	2.371817
84	1	3.772389	3.228729	-0.042681
85	1	0.929656	3.386361	-2.288858

C2

B3LYP/6-31G(d)//B3LYP/6-31G(d)

HF = -1325.2928466 hartrees (-831634.514169966 kcal/mol)

Imaginary Frequencies: none found

Zero-point correction = 0.790064 (Hartree/Particle)

mPW1PW91/6-31+G(d,p)//B3LYP/6-31G(d)

HF = -1325.1222667 hartrees (-831527.473576917 kcal/mol)

Coordinates (from last standard orientation):

Center Number	Atomic Number	Coordinates (Angstroms) X	Y	Z
1	6	2.859758	-1.200822	-1.641338
2	6	2.433681	-0.255610	-0.448989
3	6	3.749739	0.274688	0.212183
4	6	4.789176	-0.789969	0.711955
5	6	5.073341	-1.744851	-0.480593
6	6	3.805798	-2.300787	-1.141603
7	1	3.293188	-2.973976	-0.446784
8	6	1.610061	0.872260	-1.013421
9	6	2.188032	2.229535	-0.948258

10	6	2.793027	2.590525	0.450385
11	1	1.994065	3.068299	1.022539
12	6	3.388014	1.390427	1.208272
13	6	0.410207	0.574154	-1.839512
14	1	0.709105	0.944079	-2.838291
15	1	0.265135	-0.501736	-1.938033
16	6	-0.974290	1.233174	-1.531373
17	1	-1.553426	1.078083	-2.444171
18	1	-0.857906	2.309409	-1.432069
19	6	-1.722583	0.630217	-0.311298
20	1	-1.177306	-0.273710	-0.015271
21	6	-1.609682	1.549063	0.938274
22	6	-2.224130	0.838511	2.153490
23	1	-1.586696	-0.020099	2.403633
24	6	-2.164895	2.975497	0.812238
25	1	-1.846566	3.559041	1.685320
26	1	-1.801716	3.495519	-0.078575
27	1	-3.255238	2.999257	0.793933
28	6	-3.178184	0.108308	-0.630298
29	6	-3.687425	-0.609364	0.677484
30	1	-2.912964	-1.369585	0.879324
31	6	-3.652061	0.339136	1.890451
32	1	-4.002461	-0.180538	2.786554
33	1	-4.332855	1.187133	1.750845
34	1	-2.206460	1.512069	3.022146
35	6	-3.073125	-0.973281	-1.742775
36	1	-2.789462	-0.517821	-2.700353
37	1	-2.267107	-1.675408	-1.476694

38	6	-5.001736	-1.457878	0.548251
39	6	-4.822555	-2.439571	-0.637635
40	1	-4.077077	-3.196154	-0.349757
41	6	-4.365454	-1.777641	-1.939392
42	1	-5.158061	-1.133342	-2.338238
43	1	-4.195520	-2.545161	-2.704519
44	6	-6.289362	-0.623539	0.374982
45	1	-7.163272	-1.282463	0.436904
46	1	-6.395877	0.125621	1.167684
47	1	-6.346177	-0.103822	-0.583899
48	6	-5.180657	-2.317597	1.821343
49	1	-5.481077	-1.723941	2.690958
50	1	-5.965918	-3.064781	1.658158
51	1	-4.260415	-2.857247	2.078308
52	6	-4.090712	1.256392	-1.132758
53	1	-4.882359	0.884443	-1.785753
54	1	-4.581801	1.799032	-0.322869
55	1	-3.523814	1.988114	-1.719125
56	1	4.091598	-2.915142	-2.006097
57	8	-0.163219	1.665323	1.130393
58	1	-0.023927	2.131132	1.971746
59	1	-5.761688	-2.983251	-0.801492
60	6	1.496270	-1.054428	0.513585
61	1	1.055126	-0.409366	1.272832
62	1	0.683855	-1.530914	-0.040344
63	1	2.067927	-1.838797	1.001568
64	8	5.897132	-2.796742	0.001759
65	1	6.244940	-3.292436	-0.755559

66	1	5.617881	-1.155963	-1.240634
67	1	3.369588	-0.601424	-2.407853
68	1	1.983620	-1.652831	-2.113519
69	1	2.671547	1.013822	1.945613
70	1	4.268903	1.714609	1.766242
71	6	1.454530	3.422182	-1.563664
72	1	2.153142	4.260902	-1.641451
73	1	0.625549	3.742152	-0.928218
74	1	3.081283	2.031100	-1.585532
75	1	4.299361	0.766595	-0.607239
76	6	6.114039	-0.052894	1.029478
77	1	6.916670	-0.788253	1.128021
78	1	6.392533	0.649355	0.233366
79	1	6.059494	0.503654	1.970493
80	6	4.392519	-1.577349	1.982060
81	1	3.934134	-0.920560	2.729170
82	1	3.711254	-2.410383	1.792979
83	1	5.287227	-2.016347	2.429323
84	1	3.557958	3.351611	0.267398
85	1	1.072703	3.220861	-2.569066

TS (C2-D2)

B3LYP/6-31G(d)//B3LYP/6-31G(d)

HF = -1325.2929496 hartrees (-831634.578803496 kcal/mol)

Imaginary Frequencies: 1 (-41.8966 1/cm)

Zero-point correction = 0.789791 (Hartree/Particle)

mPW1PW91/6-31+G(d,p)//B3LYP/6-31G(d)

HF = -1325.1220757 hartrees (-831527.353722507 kcal/mol)

Coordinates (from last standard orientation):

Center Number	Atomic Number	X	Y	Z
1	6	2.947406	-1.102628	-1.696453
2	6	2.458099	-0.282257	-0.431903
3	6	3.749227	0.282456	0.253523
4	6	4.832779	-0.752808	0.711001
5	6	5.196135	-1.594360	-0.544965
6	6	3.980163	-2.165046	-1.288053
7	1	3.521274	-2.949379	-0.678048
8	6	1.580156	0.841450	-0.943824
9	6	2.136620	2.212431	-0.867631
10	6	2.754829	2.567196	0.522471
11	1	1.972959	3.062641	1.105029
12	6	3.341135	1.358382	1.273295
13	6	0.423869	0.520549	-1.830327
14	1	0.761087	0.901089	-2.812284
15	1	0.320599	-0.559424	-1.940931
16	6	-0.997209	1.115677	-1.588423
17	1	-1.540463	0.892744	-2.509140
18	1	-0.948669	2.201241	-1.526368
19	6	-1.741258	0.506043	-0.373702
20	1	-1.267337	-0.463173	-0.169710

21	6	-1.475825	1.323754	0.919210
22	6	-2.059567	0.578612	2.126762
23	1	-1.486155	-0.346790	2.268912
24	6	-1.924784	2.792757	0.929424
25	1	-1.473598	3.297949	1.792881
26	1	-1.622974	3.338943	0.032011
27	1	-3.005681	2.893605	1.033115
28	6	-3.255097	0.138697	-0.632761
29	6	-3.747140	-0.620276	0.659403
30	1	-3.039364	-1.461862	0.753546
31	6	-3.542035	0.226065	1.929968
32	1	-3.879113	-0.328357	2.810053
33	1	-4.148464	1.139269	1.901309
34	1	-1.925850	1.181824	3.036014
35	6	-3.322171	-0.871310	-1.814093
36	1	-3.063247	-0.379143	-2.759940
37	1	-2.569290	-1.658629	-1.652751
38	6	-5.143377	-1.331343	0.570105
39	6	-5.137463	-2.240930	-0.684405
40	1	-4.456665	-3.084654	-0.496136
41	6	-4.695985	-1.538590	-1.970142
42	1	-5.445789	-0.799179	-2.277135
43	1	-4.646555	-2.265859	-2.789783
44	6	-6.349725	-0.367813	0.541362
45	1	-7.280004	-0.943121	0.614223
46	1	-6.333338	0.324528	1.390801
47	1	-6.410306	0.227715	-0.372122
48	6	-5.324371	-2.256947	1.795932

49	1	-5.507409	-1.699685	2.720598
50	1	-6.188710	-2.913045	1.641898
51	1	-4.447473	-2.897827	1.951852
52	6	-4.080245	1.394434	-1.011188
53	1	-4.944790	1.135560	-1.624647
54	1	-4.463042	1.939128	-0.145836
55	1	-3.481354	2.096082	-1.603138
56	1	4.323319	-2.660527	-2.206432
57	8	-0.011836	1.319264	1.010961
58	1	0.233084	1.723636	1.859477
59	1	-6.135141	-2.678146	-0.818227
60	6	1.594878	-1.225715	0.454969
61	1	1.324981	-0.754277	1.398490
62	1	0.667954	-1.506055	-0.049673
63	1	2.142615	-2.140572	0.661335
64	8	6.063141	-2.639006	-0.126338
65	1	6.461756	-3.046693	-0.910660
66	1	5.727718	-0.917875	-1.237888
67	1	3.403848	-0.411883	-2.418524
68	1	2.105156	-1.585968	-2.198524
69	1	2.609538	0.948228	1.978003
70	1	4.197279	1.681655	1.868775
71	6	1.364084	3.394968	-1.453777
72	1	2.031297	4.261250	-1.499914
73	1	0.518281	3.664593	-0.817450
74	1	3.015888	2.045056	-1.530867
75	1	4.278682	0.823762	-0.547692
76	6	6.108039	0.027254	1.115360

77	1	6.947697	-0.669647	1.178920
78	1	6.364215	0.801360	0.380760
79	1	6.003087	0.507382	2.093538
80	6	4.449880	-1.647962	1.910591
81	1	3.989008	-1.056348	2.709550
82	1	3.771003	-2.464293	1.657363
83	1	5.351243	-2.112059	2.317256
84	1	3.531510	3.316681	0.339090
85	1	0.998975	3.210046	-2.468334

D2

B3LYP/6-31G(d)//B3LYP/6-31G(d)

HF = -1325.2999789 hartrees (-831638.989759539 kcal/mol)

Imaginary Frequencies: none found

Zero-point correction = 0.792954 (Hartree/Particle)

mPW1PW91/6-31+G(d,p)//B3LYP/6-31G(d)

HF = -1325.132435 hartrees (-831533.85428685 kcal/mol)

Coordinates (from last standard orientation):

Center Number	Atomic Number	Coordinates (Angstroms) X	Y	Z
1	6	-2.927357	-0.725053	1.820794
2	6	-2.372001	-0.289756	0.429388
3	6	-3.630581	0.232965	-0.365118

4	6	-4.789608	-0.788226	-0.621544
5	6	-5.229431	-1.286169	0.785019
6	6	-4.073025	-1.746757	1.683266
7	1	-3.699025	-2.704851	1.307007
8	6	-1.306872	0.901860	0.594880
9	6	-1.988322	2.273783	0.434983
10	6	-2.604454	2.429016	-0.983213
11	1	-1.865433	2.871527	-1.667468
12	6	-3.193160	1.109032	-1.548095
13	6	-0.393414	0.745288	1.820396
14	1	-0.883784	1.266554	2.650024
15	1	-0.357710	-0.308971	2.104969
16	6	1.069240	1.212210	1.686691
17	1	1.555616	1.003433	2.642848
18	1	1.137459	2.292717	1.538143
19	6	1.764942	0.430897	0.558488
20	1	1.405616	-0.604310	0.645891
21	6	1.247004	0.903183	-0.806125
22	6	1.636801	-0.077245	-1.906358
23	1	1.156533	-1.042937	-1.714448
24	6	1.479613	2.348233	-1.233575
25	1	0.752319	2.641282	-1.999993
26	1	1.428712	3.063069	-0.413571
27	1	2.463953	2.439808	-1.694720
28	6	3.334647	0.285269	0.605091
29	6	3.695404	-0.736589	-0.539791
30	1	3.099268	-1.630926	-0.291046
31	6	3.170855	-0.268283	-1.912008

32	1	3.399960	-1.012393	-2.679250
33	1	3.666000	0.655264	-2.232806
34	1	1.293116	0.279195	-2.886384
35	6	3.717560	-0.369145	1.961060
36	1	3.555495	0.338857	2.782934
37	1	3.051273	-1.225438	2.145346
38	6	5.167392	-1.279559	-0.555822
39	6	5.477958	-1.833702	0.858961
40	1	4.885643	-2.748725	1.008561
41	6	5.171199	-0.862424	2.002063
42	1	5.862898	-0.011867	1.975485
43	1	5.349576	-1.357766	2.963885
44	6	6.230432	-0.244600	-0.982927
45	1	7.198083	-0.744920	-1.103249
46	1	5.986644	0.214990	-1.947722
47	1	6.373951	0.558799	-0.257339
48	6	5.262233	-2.468972	-1.539366
49	1	5.228474	-2.152070	-2.587187
50	1	6.213793	-2.993122	-1.396302
51	1	4.457946	-3.197182	-1.375561
52	6	4.034113	1.663231	0.509662
53	1	5.029562	1.635254	0.954678
54	1	4.161259	2.020659	-0.514521
55	1	3.471619	2.423941	1.062362
56	1	-4.467534	-1.955821	2.686624
57	8	-0.321704	0.742428	-0.657707
58	1	-0.771983	1.050077	-1.465774
59	1	6.530339	-2.141416	0.900579

60	6	-1.654607	-1.501780	-0.209262
61	1	-1.528581	-1.403940	-1.289959
62	1	-0.662399	-1.643272	0.231136
63	1	-2.207237	-2.420927	-0.032453
64	8	-6.171271	-2.335173	0.596880
65	1	-6.583182	-2.536947	1.451216
66	1	-5.719358	-0.428383	1.279650
67	1	-3.295790	0.156481	2.364027
68	1	-2.146854	-1.172907	2.443472
69	1	-2.479409	0.564875	-2.188286
70	1	-4.029824	1.346368	-2.208232
71	6	-1.226848	3.541129	0.844201
72	1	-1.917109	4.390451	0.804340
73	1	-0.399708	3.774207	0.170161
74	1	-2.821829	2.195047	1.145599
75	1	-4.116666	0.936282	0.326456
76	6	-5.997149	-0.026960	-1.222434
77	1	-6.891318	-0.649367	-1.132394
78	1	-6.188749	0.918437	-0.698580
79	1	-5.857218	0.194954	-2.285510
80	6	-4.473266	-1.955183	-1.581336
81	1	-3.998453	-1.586285	-2.498496
82	1	-3.830399	-2.724396	-1.151500
83	1	-5.404137	-2.450733	-1.867084
84	1	-3.401930	3.176250	-0.913105
85	1	-0.841468	3.488876	1.866348

Figure S5

A4

B3LYP/6-31G(d)//B3LYP/6-31G(d)

HF = -1325.2928738 hartrees (-831634.531238238 kcal/mol)

Imaginary Frequencies: none found

Zero-point correction = 0.789474 (Hartree/Particle)

mPW1PW91/6-31+G(d,p)//B3LYP/6-31G(d)

HF = -1325.1233606 hartrees (-831528.160010106 kcal/mol)

Coordinates (from last standard orientation):

Center Number	Atomic Number	Coordinates (Angstroms) X	Y	Z
1	6	3.227911	-0.974819	-1.596547
2	6	2.615809	-0.549170	-0.246475
3	6	3.696484	0.283089	0.512934
4	6	5.048724	-0.440900	0.819818
5	6	5.579556	-0.955953	-0.551517
6	6	4.548341	-1.740486	-1.376251
7	1	4.364267	-2.704711	-0.891415
8	6	1.264440	0.404292	-0.454775
9	6	1.629785	1.810196	-0.288777
10	6	2.184534	2.198737	1.036184
11	1	1.311119	2.417784	1.668655
12	6	3.042297	1.067080	1.664478
13	6	0.408751	0.007299	-1.668675

14	1	0.964972	0.188805	-2.595592
15	1	0.263569	-1.077099	-1.618162
16	6	-0.979640	0.677878	-1.790315
17	1	-1.472806	0.206506	-2.645584
18	1	-0.846405	1.722015	-2.083046
19	6	-1.884486	0.602928	-0.528604
20	1	-1.329151	0.053015	0.244455
21	6	-2.088586	2.006103	0.108953
22	6	-2.849630	1.869526	1.436954
23	1	-2.187625	1.359878	2.148904
24	6	-2.724100	3.083725	-0.779335
25	1	-2.656349	4.057110	-0.274672
26	1	-2.231882	3.177141	-1.751051
27	1	-3.783432	2.895807	-0.955758
28	6	-3.206154	-0.241096	-0.702382
29	6	-3.874852	-0.334578	0.719389
30	1	-3.083210	-0.763909	1.357768
31	6	-4.151308	1.065397	1.300376
32	1	-4.602245	0.982412	2.293511
33	1	-4.876243	1.614180	0.687546
34	1	-3.053713	2.866654	1.850729
35	6	-2.794813	-1.679884	-1.122986
36	1	-2.379374	-1.677734	-2.139190
37	1	-1.989778	-2.024815	-0.455094
38	6	-5.054775	-1.357008	0.874953
39	6	-4.569840	-2.733001	0.349533
40	1	-3.821538	-3.126965	1.053818
41	6	-3.945695	-2.692437	-1.048074

42	1	-4.709331	-2.460553	-1.800297
43	1	-3.565207	-3.687178	-1.310956
44	6	-6.364327	-0.938916	0.171882
45	1	-7.167285	-1.629841	0.454177
46	1	-6.684063	0.065007	0.473602
47	1	-6.297307	-0.953820	-0.918059
48	6	-5.377850	-1.535693	2.376692
49	1	-5.886498	-0.664633	2.803009
50	1	-6.044597	-2.394565	2.515060
51	1	-4.471532	-1.723484	2.966132
52	6	-4.122362	0.357682	-1.801044
53	1	-4.735893	-0.411103	-2.274388
54	1	-4.810707	1.116027	-1.422004
55	1	-3.536101	0.820009	-2.602347
56	1	4.982394	-1.974494	-2.357358
57	8	-0.718329	2.454728	0.424778
58	1	-0.784625	3.391840	0.676407
59	1	-5.408042	-3.441259	0.367333
60	6	2.072730	-1.779017	0.512138
61	1	1.150422	-2.139389	0.045220
62	1	2.776477	-2.609519	0.497474
63	1	1.849179	-1.553848	1.559638
64	8	6.731072	-1.746578	-0.289952
65	1	7.162167	-1.954134	-1.133457
66	1	5.864285	-0.060557	-1.134128
67	1	3.427592	-0.086405	-2.212780
68	1	2.535366	-1.604180	-2.164324
69	1	2.432056	0.414828	2.297027

70	1	3.780433	1.533888	2.319587
71	1	4.006205	1.055296	-0.214903
72	6	6.066435	0.603767	1.337719
73	1	7.070285	0.172878	1.302530
74	1	6.068715	1.513672	0.723388
75	1	5.868363	0.892156	2.375392
76	6	4.978299	-1.570461	1.869564
77	1	4.453463	-1.234338	2.771310
78	1	4.489053	-2.477039	1.510622
79	1	5.991711	-1.855262	2.161266
80	1	2.753305	3.130734	0.945408
81	1	0.716152	0.175932	0.467135
82	6	1.723149	2.775907	-1.401443
83	1	1.615909	3.809475	-1.060324
84	1	2.762890	2.684859	-1.768808
85	1	1.059665	2.566600	-2.238199

TS (A4-B4)

B3LYP/6-31G(d)//B3LYP/6-31G(d)

HF = -1325.2923137 hartrees (-831634.179769887 kcal/mol)

Imaginary Frequencies: 1 (-38.2880 1/cm)

Zero-point correction = 0.789995 (Hartree/Particle)

mPW1PW91/6-31+G(d,p)//B3LYP/6-31G(d)

HF = -1325.1216819 hartrees (-831527.106609069 kcal/mol)

Coordinates (from last standard orientation):

Center Number	Atomic Number	Coordinates (Angstroms) X	Y	Z
1	6	3.283441	-1.164204	-1.463082
2	6	2.682609	-0.648747	-0.136436
3	6	3.731549	0.326789	0.492171
4	6	5.151160	-0.259665	0.794056
5	6	5.664713	-0.863814	-0.545237
6	6	4.666710	-1.803805	-1.234506
7	1	4.580215	-2.724027	-0.647612
8	6	1.258952	0.162046	-0.356467
9	6	1.488070	1.623962	-0.303719
10	6	2.077471	2.155624	0.973466
11	1	1.257773	2.348685	1.674031
12	6	3.075086	1.147436	1.611855
13	6	0.437569	-0.390589	-1.537505
14	1	1.016828	-0.316438	-2.464017
15	1	0.310979	-1.463438	-1.353816
16	6	-0.954029	0.224897	-1.803005
17	1	-1.428301	-0.414040	-2.552800
18	1	-0.829322	1.188062	-2.304083
19	6	-1.879626	0.388832	-0.568976
20	1	-1.397776	-0.119726	0.277791
21	6	-1.957409	1.870399	-0.112783
22	6	-2.754286	1.985071	1.196073
23	1	-2.173080	1.499463	1.989752
24	6	-2.439001	2.901588	-1.138962
25	1	-2.289603	3.913721	-0.739412

26	1	-1.913292	2.836804	-2.094499
27	1	-3.506339	2.798630	-1.336831
28	6	-3.280862	-0.332863	-0.685775
29	6	-3.986796	-0.173035	0.711517
30	1	-3.257819	-0.592072	1.426302
31	6	-4.130473	1.309333	1.098510
32	1	-4.618652	1.402547	2.072657
33	1	-4.771191	1.847134	0.389782
34	1	-2.859886	3.041911	1.476293
35	6	-3.016220	-1.848711	-0.907695
36	1	-2.588029	-2.021355	-1.902956
37	1	-2.263454	-2.186700	-0.178883
38	6	-5.272000	-1.039956	0.956218
39	6	-4.922257	-2.513727	0.628312
40	1	-4.232002	-2.885180	1.400610
41	6	-4.272150	-2.716295	-0.742402
42	1	-4.992578	-2.501874	-1.541059
43	1	-3.996263	-3.770347	-0.868399
44	6	-6.513081	-0.584309	0.158629
45	1	-7.388788	-1.155797	0.487538
46	1	-6.738711	0.474419	0.330586
47	1	-6.417542	-0.734552	-0.918886
48	6	-5.645901	-0.986821	2.455966
49	1	-6.071255	-0.021271	2.749121
50	1	-6.401912	-1.748630	2.678117
51	1	-4.778611	-1.186771	3.097714
52	6	-4.098201	0.211833	-1.885817
53	1	-4.775419	-0.544947	-2.285589

54	1	-4.714630	1.077020	-1.632211
55	1	-3.443764	0.505858	-2.713438
56	1	5.076520	-2.105039	-2.207831
57	8	-0.544026	2.222332	0.229108
58	1	-0.511940	3.187107	0.356677
59	1	-5.829341	-3.126107	0.709560
60	6	2.276795	-1.844737	0.755822
61	1	1.373715	-2.321951	0.360432
62	1	3.047153	-2.613488	0.790407
63	1	2.066811	-1.541091	1.786163
64	8	6.889700	-1.530017	-0.269252
65	1	7.291742	-1.797431	-1.110223
66	1	5.847348	-0.011231	-1.225184
67	1	3.390268	-0.331668	-2.173360
68	1	2.624685	-1.899189	-1.936193
69	1	2.561182	0.499779	2.329092
70	1	3.805551	1.724845	2.182266
71	1	3.947479	1.052772	-0.311264
72	6	6.103685	0.904222	1.160569
73	1	7.136773	0.550890	1.109793
74	1	6.002251	1.750041	0.467977
75	1	5.932300	1.273235	2.177251
76	6	5.215552	-1.282442	1.948255
77	1	4.696026	-0.904180	2.836236
78	1	4.793091	-2.256202	1.697762
79	1	6.259195	-1.453961	2.221480
80	1	2.568308	3.114862	0.775593
81	1	0.729828	-0.053237	0.577741

82	6	1.650943	2.445905	-1.530988
83	1	1.544054	3.515970	-1.333005
84	1	2.698238	2.293626	-1.845453
85	1	1.013697	2.140725	-2.358958

B4

B3LYP/6-31G(d)//B3LYP/6-31G(d)

HF = -1325.2957415 hartrees (-831636.330748665 kcal/mol)

Imaginary Frequencies: none found

Zero-point correction = 0.792784 (Hartree/Particle)

mPW1PW91/6-31+G(d,p)//B3LYP/6-31G(d)

HF = -1325.1267061 hartrees (-831530.259344811 kcal/mol)

Coordinates (from last standard orientation):

Center Number	Atomic Number	Coordinates (Angstroms) X	Y	Z
1	6	3.322161	-1.451534	-1.184072
2	6	2.728509	-0.728752	0.053285
3	6	3.739307	0.408446	0.423613
4	6	5.220091	-0.004959	0.724897
5	6	5.711388	-0.811863	-0.508441
6	6	4.760831	-1.936969	-0.931927
7	1	4.782514	-2.725406	-0.172349
8	6	1.258580	-0.092477	-0.198782

9	6	1.239207	1.434076	-0.379656
10	6	2.030918	2.210752	0.707711
11	1	1.340728	2.620876	1.454104
12	6	3.088232	1.359162	1.434004
13	6	0.485509	-0.900952	-1.267967
14	1	1.076884	-0.961253	-2.185687
15	1	0.430421	-1.927102	-0.887482
16	6	-0.939788	-0.472876	-1.685720
17	1	-1.392716	-1.346117	-2.160458
18	1	-0.879168	0.267292	-2.488087
19	6	-1.856258	0.043788	-0.556829
20	1	-1.474661	-0.345458	0.396192
21	6	-1.779024	1.574775	-0.412850
22	6	-2.560584	2.066081	0.805102
23	1	-2.090661	1.665375	1.710512
24	6	-2.020425	2.454599	-1.633643
25	1	-1.766976	3.496806	-1.405659
26	1	-1.457008	2.149193	-2.515147
27	1	-3.080310	2.438985	-1.893527
28	6	-3.353287	-0.490143	-0.611875
29	6	-4.080347	0.047206	0.675167
30	1	-3.449792	-0.311262	1.506563
31	6	-4.021234	1.581973	0.750839
32	1	-4.517461	1.942550	1.655622
33	1	-4.547253	2.049197	-0.089539
34	1	-2.521544	3.162267	0.867152
35	6	-3.302933	-2.040848	-0.502229
36	1	-2.871636	-2.478120	-1.410005

37	1	-2.634433	-2.315738	0.327334
38	6	-5.489924	-0.562708	1.000028
39	6	-5.350174	-2.106066	1.000269
40	1	-4.758851	-2.400361	1.880197
41	6	-4.678367	-2.675420	-0.251292
42	1	-5.326131	-2.541711	-1.126012
43	1	-4.551459	-3.759043	-0.141398
44	6	-6.616862	-0.116035	0.043659
45	1	-7.580782	-0.472041	0.424779
46	1	-6.683590	0.976074	-0.023351
47	1	-6.508494	-0.507029	-0.970191
48	6	-5.907081	-0.143825	2.429060
49	1	-6.197965	0.910293	2.490700
50	1	-6.774678	-0.731679	2.748964
51	1	-5.103863	-0.320803	3.155178
52	6	-4.033593	-0.104488	-1.950047
53	1	-4.820603	-0.812121	-2.214278
54	1	-4.500222	0.883848	-1.932613
55	1	-3.316094	-0.119514	-2.777387
56	1	5.142934	-2.396687	-1.853575
57	8	-0.275904	1.877302	0.038608
58	1	-0.233679	2.839233	0.196597
59	1	-6.341505	-2.556893	1.134481
60	6	2.490357	-1.771774	1.174599
61	1	1.615508	-2.387006	0.934185
62	1	3.327069	-2.456698	1.300385
63	1	2.301580	-1.301467	2.145025
64	8	7.005973	-1.317469	-0.203829

65	1	7.369440	-1.728532	-1.003346
66	1	5.779265	-0.095209	-1.347916
67	1	3.328810	-0.768407	-2.045943
68	1	2.709648	-2.312055	-1.471796
69	1	2.629364	0.803025	2.258278
70	1	3.812881	2.038912	1.888283
71	1	3.847716	0.993173	-0.504697
72	6	6.089017	1.274302	0.803214
73	1	7.144280	0.995190	0.744469
74	1	5.875181	1.963773	-0.023907
75	1	5.944712	1.815793	1.743996
76	6	5.434984	-0.781511	2.041620
77	1	4.930801	-0.281272	2.876686
78	1	5.085131	-1.813893	2.005494
79	1	6.502541	-0.818964	2.271094
80	1	2.521382	3.064798	0.226031
81	1	0.745177	-0.231996	0.759095
82	6	1.484615	1.965461	-1.780227
83	1	1.330958	3.048655	-1.833808
84	1	2.533450	1.777955	-2.034160
85	1	0.869660	1.477381	-2.536380

Figure S6

A5 (24)

B3LYP/6-31G(d)//B3LYP/6-31G(d)

HF = -1325.2922916 hartrees (-831634.165901916 kcal/mol)

Imaginary Frequencies: none found

Zero-point correction = 0.788821 (Hartree/Particle)

mPW1PW91/6-31+G(d,p)//B3LYP/6-31G(d)

HF = -1325.1233298 hartrees (-831528.140682798 kcal/mol)

Coordinates (from last standard orientation):

Center Number	Atomic Number	Coordinates (Angstroms) X	Y	Z
1	6	3.155252	-0.937906	-1.615106
2	6	2.574710	-0.481198	-0.261580
3	6	3.698299	0.291103	0.496007
4	6	5.004671	-0.510014	0.802673
5	6	5.504436	-1.053965	-0.569837
6	6	4.429996	-1.778172	-1.395447
7	1	4.193012	-2.732078	-0.913038
8	6	1.283607	0.561525	-0.477861
9	6	1.748048	1.931517	-0.323016
10	6	2.302878	2.290314	1.004884
11	1	1.414090	2.544609	1.607990
12	6	3.089545	1.116288	1.646402
13	6	0.382580	0.215041	-1.672528
14	1	0.912917	0.406729	-2.613096
15	1	0.209881	-0.865442	-1.647682
16	6	-0.987317	0.932046	-1.717296
17	1	-1.493377	0.572689	-2.618963
18	1	-0.824538	1.998749	-1.889788

19	6	-1.896182	0.736269	-0.469104
20	1	-1.302138	0.208150	0.290771
21	6	-2.230833	2.089576	0.224044
22	6	-2.997459	1.827301	1.530335
23	1	-2.299482	1.355532	2.234992
24	6	-2.950266	3.137394	-0.637763
25	1	-3.991377	2.874582	-0.828797
26	1	-2.955670	4.098630	-0.107565
27	1	-2.457815	3.293753	-1.601912
28	6	-3.138994	-0.213524	-0.696869
29	6	-3.805468	-0.428966	0.712422
30	1	-2.982072	-0.810275	1.340948
31	6	-4.216075	0.913114	1.344879
32	1	-4.670646	0.746011	2.325582
33	1	-4.980956	1.417440	0.742762
34	1	-3.300614	2.784939	1.977489
35	6	-2.604919	-1.589831	-1.181905
36	1	-2.187039	-1.500744	-2.193263
37	1	-1.777499	-1.899210	-0.523665
38	6	-4.890167	-1.558702	0.812222
39	6	-4.285065	-2.860109	0.226964
40	1	-3.505398	-3.218933	0.916023
41	6	-3.665681	-2.698810	-1.163871
42	1	-4.445717	-2.495391	-1.907466
43	1	-3.202049	-3.643686	-1.473448
44	6	-6.229145	-1.224055	0.119455
45	1	-6.966864	-2.000361	0.354024
46	1	-6.640904	-0.272374	0.474083

47	1	-6.154189	-1.169943	-0.968725
48	6	-5.201869	-1.835952	2.301421
49	1	-5.783398	-1.032407	2.764974
50	1	-5.794588	-2.753391	2.393564
51	1	-4.284996	-1.975351	2.887973
52	6	-4.101047	0.353120	-1.773861
53	1	-4.621722	-0.445607	-2.305733
54	1	-4.872521	1.006393	-1.361102
55	1	-3.561681	0.928215	-2.533367
56	1	4.849507	-2.033646	-2.377515
57	8	-0.917831	2.648133	0.570815
58	1	-1.097582	3.469547	1.058674
59	1	-5.057079	-3.639937	0.207216
60	6	1.955786	-1.674761	0.494470
61	1	1.014109	-1.976876	0.024844
62	1	2.607059	-2.547356	0.478886
63	1	1.744937	-1.438645	1.542131
64	8	6.607856	-1.910173	-0.307676
65	1	7.031788	-2.136062	-1.150090
66	1	5.840375	-0.176633	-1.151981
67	1	3.403391	-0.060800	-2.228722
68	1	2.425471	-1.525855	-2.180482
69	1	2.443690	0.501931	2.281891
70	1	3.853927	1.541656	2.299110
71	1	4.045935	1.045509	-0.234919
72	6	6.081341	0.473484	1.321338
73	1	7.058502	-0.014774	1.283659
74	1	6.135089	1.382880	0.708806

75	1	5.901557	0.770216	2.359956
76	6	4.868499	-1.635273	1.850558
77	1	4.354838	-1.274058	2.748963
78	1	4.336144	-2.514470	1.485470
79	1	5.863714	-1.972595	2.148461
80	1	2.909728	3.198414	0.937384
81	1	0.737411	0.376568	0.457518
82	6	1.701676	2.946724	-1.387122
83	1	2.535748	3.652060	-1.326503
84	1	1.603721	2.527095	-2.389873
85	1	0.780148	3.519465	-1.181003

TS (A5-C3)

B3LYP/6-31G(d)//B3LYP/6-31G(d)

HF = -1325.2898909 hartrees (-831632.659438659 kcal/mol)

Imaginary Frequencies: 1 (-223.2825 1/cm)

Zero-point correction = 0.786511 (Hartree/Particle)

mPW1PW91/6-31+G(d,p)//B3LYP/6-31G(d)

HF = -1325.1250975 hartrees (-831529.249932225 kcal/mol)

Coordinates (from last standard orientation):

Center Number	Atomic Number	Coordinates (Angstroms) X	Y	Z
1	6	2.652964	-1.298294	-1.435850

2	6	2.382911	-0.261916	-0.301888
3	6	3.784961	0.264377	0.164320
4	6	4.840660	-0.795309	0.633908
5	6	4.971614	-1.841982	-0.504777
6	6	3.626660	-2.397213	-0.982187
7	1	3.185640	-3.008435	-0.187700
8	6	1.565583	0.931197	-0.904242
9	6	1.982091	2.258510	-0.722159
10	6	3.075457	2.634359	0.253416
11	1	2.715342	3.470214	0.868930
12	6	3.606303	1.473532	1.091872
13	6	0.405737	0.612159	-1.862328
14	1	0.737584	0.895229	-2.870783
15	1	0.269893	-0.468431	-1.888877
16	6	-0.987866	1.246693	-1.602321
17	1	-1.569062	1.032421	-2.502814
18	1	-0.910474	2.333568	-1.572186
19	6	-1.742687	0.718782	-0.346473
20	1	-1.141199	-0.096738	0.072471
21	6	-1.776402	1.763223	0.805355
22	6	-2.394498	1.135280	2.062867
23	1	-1.701330	0.368747	2.433174
24	6	-2.416698	3.123610	0.500161
25	1	-3.502848	3.055749	0.427097
26	1	-2.192499	3.820136	1.317638
27	1	-2.045977	3.569763	-0.426771
28	6	-3.134875	0.038306	-0.660051
29	6	-3.645625	-0.585203	0.693323

30	1	-2.818130	-1.243059	1.010057
31	6	-3.758709	0.485354	1.793897
32	1	-4.109724	0.036116	2.727076
33	1	-4.500249	1.248997	1.531664
34	1	-2.480622	1.897344	2.850627
35	6	-2.881804	-1.135024	-1.647946
36	1	-2.590114	-0.753194	-2.634473
37	1	-2.033743	-1.732956	-1.278887
38	6	-4.870628	-1.562506	0.602495
39	6	-4.549286	-2.636671	-0.467589
40	1	-3.754845	-3.289607	-0.075560
41	6	-4.090137	-2.068062	-1.812446
42	1	-4.915484	-1.540484	-2.305853
43	1	-3.818005	-2.888663	-2.487487
44	6	-6.216898	-0.873103	0.294674
45	1	-7.030580	-1.602377	0.382912
46	1	-6.429684	-0.066962	1.005894
47	1	-6.269827	-0.452980	-0.711844
48	6	-5.031985	-2.302609	1.950863
49	1	-5.425700	-1.655284	2.741437
50	1	-5.738749	-3.132450	1.836500
51	1	-4.080754	-2.725207	2.298042
52	6	-4.117595	1.040177	-1.318395
53	1	-4.849998	0.528850	-1.945468
54	1	-4.684030	1.629869	-0.594271
55	1	-3.590352	1.743713	-1.972054
56	1	3.797678	-3.076829	-1.827456
57	8	-0.347502	2.018193	1.085212

58	1	-0.286566	2.534491	1.906337
59	1	-5.428480	-3.277909	-0.609816
60	6	1.521652	-0.920465	0.800861
61	1	0.554943	-1.231434	0.392737
62	1	1.998623	-1.812730	1.200178
63	1	1.321721	-0.243028	1.635208
64	8	5.818363	-2.883021	-0.034205
65	1	6.041002	-3.460868	-0.780387
66	1	5.447274	-1.327728	-1.359387
67	1	3.073932	-0.780738	-2.309292
68	1	1.725712	-1.774619	-1.766855
69	1	2.920813	1.248435	1.917262
70	1	4.550306	1.776449	1.549912
71	1	4.231241	0.670394	-0.759772
72	6	6.215382	-0.094910	0.765360
73	1	6.998333	-0.855686	0.818963
74	1	6.429320	0.550883	-0.095997
75	1	6.286479	0.512167	1.673615
76	6	4.540267	-1.469549	1.990920
77	1	4.245047	-0.727988	2.741935
78	1	3.760921	-2.232828	1.942488
79	1	5.440492	-1.970566	2.354283
80	1	3.886615	3.054931	-0.358498
81	1	0.990603	1.640966	0.052557
82	6	1.397634	3.430762	-1.473071
83	1	2.204170	4.142557	-1.674968
84	1	0.934270	3.152363	-2.419583
85	1	0.659078	3.952999	-0.855432

C3

B3LYP/6-31G(d)//B3LYP/6-31G(d)

HF = -1325.2959605 hartrees (-831636.468173355 kcal/mol)

Imaginary Frequencies: none found

Zero-point correction = 0.789460 (Hartree/Particle)

mPW1PW91/6-31+G(d,p)//B3LYP/6-31G(d)

HF = -1325.1265879 hartrees (-831530.185173129 kcal/mol)

Coordinates (from last standard orientation):

Center Number	Atomic Number	Coordinates (Angstroms) X	Y	Z
1	6	2.605344	-1.451439	-1.268568
2	6	2.473652	-0.219745	-0.323261
3	6	3.901072	0.291963	0.072495
4	6	4.930594	-0.778840	0.590715
5	6	4.931415	-1.971940	-0.400764
6	6	3.529133	-2.520187	-0.676976
7	1	3.108560	-2.941366	0.243308
8	6	1.615281	0.866967	-0.873598
9	6	1.889185	2.305708	-0.638430
10	6	3.183057	2.676230	0.120886
11	1	2.974571	3.557288	0.738365
12	6	3.772332	1.541287	0.956337

13	6	0.387068	0.509044	-1.639272
14	1	0.717327	0.694589	-2.681468
15	1	0.195749	-0.562310	-1.591685
16	6	-0.942797	1.286227	-1.410752
17	1	-1.506293	1.196875	-2.342447
18	1	-0.734941	2.348464	-1.292451
19	6	-1.794592	0.765053	-0.213693
20	1	-1.203920	-0.007608	0.294736
21	6	-1.971978	1.853106	0.886964
22	6	-2.704472	1.244473	2.094840
23	1	-2.024253	0.523650	2.567254
24	6	-2.626799	3.173578	0.455973
25	1	-3.694499	3.064880	0.260355
26	1	-2.522397	3.906386	1.266001
27	1	-2.161065	3.603756	-0.435653
28	6	-3.121819	0.023587	-0.641056
29	6	-3.746455	-0.572850	0.675837
30	1	-2.935209	-1.189928	1.099771
31	6	-4.008385	0.529851	1.717614
32	1	-4.435505	0.098260	2.627304
33	1	-4.747760	1.253071	1.353833
34	1	-2.898260	2.031581	2.837080
35	6	-2.732545	-1.169377	-1.558551
36	1	-2.350946	-0.805301	-2.522013
37	1	-1.909412	-1.724953	-1.081346
38	6	-4.922287	-1.595684	0.495859
39	6	-4.460306	-2.691096	-0.499202
40	1	-3.689479	-3.302038	-0.005682

41	6	-3.884810	-2.151464	-1.811268
42	1	-4.672169	-1.671654	-2.404836
43	1	-3.517944	-2.983824	-2.424401
44	6	-6.254649	-0.967882	0.031755
45	1	-7.048851	-1.722254	0.075270
46	1	-6.561647	-0.141251	0.682376
47	1	-6.225919	-0.591548	-0.993169
48	6	-5.193678	-2.300413	1.845667
49	1	-5.685282	-1.643775	2.570615
50	1	-5.857405	-3.158647	1.689924
51	1	-4.268368	-2.677111	2.299422
52	6	-4.074231	0.967173	-1.420710
53	1	-4.700677	0.414555	-2.123358
54	1	-4.751055	1.523691	-0.770106
55	1	-3.520620	1.702344	-2.014797
56	1	3.597447	-3.351257	-1.390895
57	8	-0.600078	2.157736	1.290039
58	1	-0.652023	2.774794	2.039244
59	1	-5.299211	-3.367406	-0.706995
60	6	1.557326	-0.596232	0.928383
61	1	0.810394	-1.334893	0.630280
62	1	2.204596	-1.025914	1.691751
63	1	1.035127	0.254639	1.376154
64	8	5.778523	-2.977339	0.139371
65	1	5.939447	-3.646529	-0.543597
66	1	5.345604	-1.603430	-1.356724
67	1	3.018839	-1.110519	-2.227811
68	1	1.630996	-1.897683	-1.483645

69	1	3.152102	1.337328	1.837110
70	1	4.748781	1.853355	1.334848
71	1	4.336709	0.631901	-0.881463
72	6	6.342836	-0.146476	0.550191
73	1	7.090069	-0.932146	0.686802
74	1	6.540307	0.345707	-0.410357
75	1	6.489594	0.590015	1.346173
76	6	4.691808	-1.273692	2.036237
77	1	4.520948	-0.435123	2.719472
78	1	3.855653	-1.972624	2.131125
79	1	5.576789	-1.810597	2.384827
80	1	3.937712	2.990466	-0.611375
81	1	1.010439	2.580404	-0.015123
82	6	1.789037	3.160145	-1.941891
83	1	2.588298	2.884388	-2.637200
84	1	0.831057	3.075850	-2.455520
85	1	1.934398	4.207838	-1.665122

TS (C3-C4)

B3LYP/6-31G(d)//B3LYP/6-31G(d)

HF = -1325.2931965 hartrees (-831634.733735715 kcal/mol)

Imaginary Frequencies: 1 (-20.5860 1/cm)

Zero-point correction = 0.789442 (Hartree/Particle)

mPW1PW91/6-31+G(d,p)//B3LYP/6-31G(d)

HF = -1325.1241801 hartrees (-831528.674254551 kcal/mol)

Coordinates (from last standard orientation):

Center Number	Atomic Number	Coordinates (Angstroms) X	Y	Z
1	6	2.034834	-1.430082	-0.813299
2	6	2.384113	-0.061758	-0.162978
3	6	3.947414	0.152668	-0.160686
4	6	4.811103	-1.042417	0.382405
5	6	4.362375	-2.335571	-0.347562
6	6	2.858521	-2.585301	-0.236226
7	1	2.602004	-2.775746	0.812049
8	6	1.655554	1.091394	-0.772373
9	6	2.145994	2.494215	-0.591334
10	6	3.665940	2.643517	-0.333697
11	1	3.831461	3.607495	0.158630
12	6	4.309601	1.509911	0.454879
13	6	0.405569	0.885396	-1.548962
14	1	0.692143	1.280642	-2.541685
15	1	0.184864	-0.168304	-1.687795
16	6	-0.908809	1.637446	-1.130364
17	1	-1.476867	1.781181	-2.052582
18	1	-0.653288	2.628511	-0.762916
19	6	-1.763677	0.899357	-0.062924
20	1	-1.138245	0.104431	0.364542
21	6	-2.042846	1.831287	1.155531
22	6	-2.757759	1.026058	2.253787
23	1	-2.037467	0.302393	2.658112

24	6	-2.784054	3.143686	0.859291
25	1	-3.839156	2.987645	0.630891
26	1	-2.739645	3.786660	1.747365
27	1	-2.333949	3.699384	0.031449
28	6	-3.026568	0.139932	-0.627303
29	6	-3.634186	-0.666522	0.581417
30	1	-2.789334	-1.276438	0.946448
31	6	-3.995666	0.271399	1.748716
32	1	-4.406587	-0.305459	2.582233
33	1	-4.781489	0.979429	1.459790
34	1	-3.029824	1.697246	3.080530
35	6	-4.027313	1.112664	-1.303793
36	1	-4.602359	0.614410	-2.086495
37	1	-4.751622	1.535776	-0.605792
38	1	-3.515435	1.952297	-1.785883
39	1	2.596222	-3.500357	-0.782814
40	8	-0.705856	2.182103	1.618193
41	1	-0.808254	2.693754	2.437678
42	6	1.724846	0.007805	1.293917
43	1	0.760381	0.523847	1.337816
44	1	1.568569	-1.017755	1.633724
45	1	2.389410	0.526279	1.983624
46	8	5.100570	-3.420093	0.199928
47	1	4.969329	-4.198613	-0.362774
48	1	4.613924	-2.210277	-1.416343
49	1	2.219813	-1.366839	-1.894436
50	1	0.973053	-1.660456	-0.683161
51	1	4.020490	1.560698	1.510184

52	1	5.393501	1.650146	0.435664
53	1	4.208489	0.217137	-1.229984
54	6	6.290866	-0.795265	0.003245
55	1	6.862575	-1.709751	0.177989
56	1	6.398382	-0.527241	-1.055501
57	1	6.745310	-0.001539	0.603707
58	6	4.740677	-1.225445	1.914053
59	1	5.061495	-0.316014	2.432640
60	1	3.744408	-1.487550	2.278964
61	1	5.415290	-2.031319	2.211591
62	1	4.155185	2.714396	-1.314225
63	1	1.592862	2.773798	0.332302
64	6	1.740052	3.504312	-1.692706
65	1	2.163313	4.478547	-1.432852
66	1	2.168941	3.210280	-2.657278
67	1	0.666128	3.636176	-1.815647
68	6	-2.539688	-0.886845	-1.685704
69	1	-2.162939	-0.365910	-2.576611
70	1	-1.693550	-1.453033	-1.264177
71	6	-4.729904	-1.735324	0.232193
72	6	-4.172859	-2.649743	-0.888965
73	1	-3.367529	-3.267714	-0.463557
74	6	-3.617386	-1.897536	-2.101607
75	1	-4.426898	-1.393738	-2.642734
76	1	-3.184926	-2.611461	-2.813490
77	6	-6.094786	-1.142075	-0.179497
78	1	-6.831345	-1.948611	-0.272211
79	1	-6.475671	-0.444641	0.574951

80	1	-6.070022	-0.616626	-1.136513
81	6	-4.974149	-2.632718	1.467574
82	1	-5.519686	-2.112557	2.261704
83	1	-5.576326	-3.503382	1.183321
84	1	-4.032406	-3.005775	1.889386
85	1	-4.957290	-3.348192	-1.206957

C4

B3LYP/6-31G(d)//B3LYP/6-31G(d)

HF = -1325.2989312 hartrees (-831638.332317312 kcal/mol)

Imaginary Frequencies: none found

Zero-point correction = 0.790497 (Hartree/Particle)

mPW1PW91/6-31+G(d,p)//B3LYP/6-31G(d)

HF = -1325.1293716 hartrees (-831531.931972716 kcal/mol)

Coordinates (from last standard orientation):

Center Number	Atomic Number	Coordinates (Angstroms) X	Y	Z
1	6	1.868049	-1.075232	0.666412
2	6	2.591587	0.281283	0.470396
3	6	3.714288	0.091175	-0.713583
4	6	4.670346	-1.145754	-0.516819
5	6	3.815761	-2.409979	-0.228325
6	6	2.838053	-2.230924	0.934057

7	1	3.394897	-2.102375	1.867942
8	6	1.763541	1.350927	-0.163143
9	6	2.344003	2.715216	-0.239891
10	6	3.458816	2.527420	-1.354836
11	1	3.958440	3.497985	-1.446198
12	6	4.437627	1.415975	-0.995479
13	6	0.471961	1.089033	-0.813452
14	1	0.310925	1.734489	-1.678023
15	1	0.343658	0.053047	-1.114754
16	6	-0.583337	1.461541	0.329778
17	1	-1.014533	2.424892	0.053413
18	1	-0.057344	1.620877	1.277270
19	6	-1.665001	0.378061	0.597019
20	1	-1.201030	-0.591274	0.373819
21	6	-1.951361	0.289170	2.129976
22	6	-2.916599	-0.879108	2.394106
23	1	-2.366201	-1.808809	2.199127
24	6	-2.436600	1.582093	2.804741
25	1	-3.458368	1.846808	2.528625
26	1	-2.423514	1.443164	3.893005
27	1	-1.788033	2.433985	2.577484
28	6	-2.944626	0.454379	-0.323238
29	6	-3.806514	-0.818841	0.023758
30	1	-3.109830	-1.661898	-0.123631
31	6	-4.175321	-0.846058	1.519016
32	1	-4.773019	-1.733889	1.744224
33	1	-4.802189	0.012214	1.788017
34	1	-3.189825	-0.889719	3.458583

35	6	-3.708715	1.792957	-0.146429
36	1	-4.244432	2.066856	-1.057114
37	1	-4.448570	1.763339	0.655078
38	1	-3.030738	2.624566	0.072079
39	1	2.252224	-3.151284	1.055962
40	8	-0.653933	-0.035161	2.696275
41	1	-0.780382	-0.174591	3.649118
42	6	3.167130	0.756396	1.835085
43	1	2.365441	1.194391	2.439293
44	1	3.554936	-0.092960	2.395568
45	1	3.975567	1.483343	1.749302
46	8	4.720086	-3.480575	0.008681
47	1	4.214600	-4.307007	0.049066
48	1	3.224065	-2.613491	-1.139737
49	1	1.305200	-1.338533	-0.235931
50	1	1.138585	-0.979061	1.477383
51	1	5.049681	1.720064	-0.140360
52	1	5.128661	1.276401	-1.832592
53	1	3.125063	-0.149571	-1.611988
54	6	5.400424	-1.388256	-1.862920
55	1	5.910597	-2.353475	-1.816522
56	1	4.699783	-1.416764	-2.706674
57	1	6.153891	-0.621778	-2.067483
58	6	5.743504	-0.942252	0.572382
59	1	6.360073	-0.063050	0.360412
60	1	5.333082	-0.832888	1.577741
61	1	6.402530	-1.813027	0.585652
62	1	2.969419	2.324948	-2.315893

63	1	2.885537	2.938051	0.682817
64	6	1.430789	3.899173	-0.577459
65	1	2.041178	4.805724	-0.629310
66	1	0.929188	3.790478	-1.544085
67	1	0.672654	4.058280	0.193921
68	6	-2.496421	0.341612	-1.806449
69	1	-1.963465	1.253854	-2.107901
70	1	-1.784344	-0.492140	-1.903666
71	6	-4.993746	-1.152215	-0.948234
72	6	-4.444536	-1.165359	-2.397366
73	1	-3.788030	-2.041053	-2.512120
74	6	-3.654833	0.087763	-2.781985
75	1	-4.319130	0.959158	-2.824587
76	1	-3.250255	-0.026103	-3.795338
77	6	-6.201966	-0.196923	-0.835672
78	1	-7.028726	-0.580051	-1.445201
79	1	-6.567731	-0.128410	0.194902
80	1	-5.989656	0.816831	-1.182582
81	6	-5.508286	-2.579339	-0.648069
82	1	-6.061450	-2.636700	0.294979
83	1	-6.192992	-2.902212	-1.440853
84	1	-4.686517	-3.304959	-0.605220
85	1	-5.276894	-1.314384	-3.096921

TS (C4-D3)

B3LYP/6-31G(d)//B3LYP/6-31G(d)

HF = -1325.2980053 hartrees (-831637.751305803 kcal/mol)

Imaginary Frequencies: 1 (-32.0139 1/cm)

Zero-point correction = 0.790246 (Hartree/Particle)

mPW1PW91/6-31+G(d,p)//B3LYP/6-31G(d)

HF = -1325.1279553 hartrees (-831531.043230303 kcal/mol)

Coordinates (from last standard orientation):

Center Number	Atomic Number	X	Y	Z
1	6	1.572432	-0.945899	0.766770
2	6	2.443075	0.299288	0.464154
3	6	3.624617	-0.142050	-0.610547
4	6	4.412450	-1.445520	-0.209383
5	6	3.400191	-2.570629	0.137681
6	6	2.391144	-2.161190	1.210442
7	1	2.912124	-1.988656	2.157901
8	6	1.793304	1.348217	-0.365489
9	6	2.509757	2.641086	-0.503109
10	6	3.704317	2.237147	-1.477278
11	1	4.316548	3.138808	-1.584313
12	6	4.513791	1.062640	-0.944028
13	6	0.617249	1.115048	-1.234534
14	1	0.794615	1.613711	-2.193479
15	1	0.434131	0.059884	-1.428166
16	6	-0.685727	1.772402	-0.600783
17	1	-1.216888	2.226492	-1.440047

18	1	-0.384129	2.593506	0.048898
19	6	-1.600356	0.798171	0.188619
20	1	-1.045246	-0.137920	0.312864
21	6	-1.804148	1.284703	1.652988
22	6	-2.592208	0.219589	2.433496
23	1	-1.939742	-0.655701	2.552449
24	6	-2.430188	2.676070	1.835523
25	1	-3.495680	2.690917	1.600821
26	1	-2.328299	2.978216	2.885534
27	1	-1.937448	3.440076	1.226533
28	6	-2.916332	0.377833	-0.571135
29	6	-3.600019	-0.733555	0.310196
30	1	-2.813993	-1.499246	0.431090
31	6	-3.887894	-0.215676	1.732579
32	1	-4.353378	-1.002311	2.333424
33	1	-4.607184	0.611861	1.715530
34	1	-2.810010	0.591874	3.444419
35	6	-3.826286	1.600553	-0.862010
36	1	-4.431859	1.446236	-1.756965
37	1	-4.520138	1.819562	-0.048459
38	1	-3.240928	2.509549	-1.038731
39	1	1.699528	-2.994258	1.392138
40	8	-0.442547	1.346999	2.168628
41	1	-0.503168	1.581452	3.109433
42	6	2.985468	0.877021	1.801077
43	1	2.214335	1.506347	2.254466
44	1	3.191940	0.068433	2.501270
45	1	3.901782	1.459678	1.700073

46	8	4.156268	-3.705877	0.535603
47	1	3.553666	-4.459556	0.630009
48	1	2.839361	-2.798662	-0.787514
49	1	1.004740	-1.244273	-0.120598
50	1	0.844425	-0.666314	1.533613
51	1	5.096478	1.373191	-0.071763
52	1	5.240316	0.768549	-1.708315
53	1	3.079926	-0.410748	-1.527882
54	6	5.198842	-1.911721	-1.461894
55	1	5.603427	-2.908511	-1.270769
56	1	4.554690	-1.974177	-2.347659
57	1	6.037581	-1.248127	-1.692309
58	6	5.424256	-1.238964	0.936512
59	1	6.166402	-0.478258	0.675527
60	1	4.962776	-0.949647	1.882391
61	1	5.957563	-2.176196	1.109308
62	1	3.286166	2.010490	-2.465799
63	1	2.979009	2.908529	0.446024
64	6	1.776314	3.869203	-1.056727
65	1	2.499244	4.683344	-1.165343
66	1	1.329038	3.697836	-2.040509
67	1	0.993705	4.215273	-0.377007
68	6	-2.505588	-0.272449	-1.921085
69	1	-2.078258	0.482572	-2.595216
70	1	-1.711783	-1.011820	-1.729863
71	6	-4.780388	-1.526696	-0.354271
72	6	-4.292138	-2.057207	-1.727560
73	1	-3.547278	-2.847538	-1.548919

74	6	-3.661842	-0.991226	-2.628922
75	1	-4.420786	-0.268967	-2.951367
76	1	-3.286914	-1.458240	-3.548012
77	6	-6.083719	-0.715745	-0.520877
78	1	-6.888166	-1.380201	-0.857264
79	1	-6.408645	-0.271855	0.426799
80	1	-6.002683	0.088801	-1.254780
81	6	-5.114063	-2.761366	0.514480
82	1	-5.617099	-2.492601	1.449134
83	1	-5.788727	-3.430708	-0.031625
84	1	-4.212412	-3.333901	0.765786
85	1	-5.130297	-2.540229	-2.245929

D3 (21+H$^+$)

B3LYP/6-31G(d)//B3LYP/6-31G(d)

HF = -1325.3107582 hartrees (-831645.753878082 kcal/mol)

Imaginary Frequencies: none found

Zero-point correction = 0.792668 (Hartree/Particle)

mPW1PW91/6-31+G(d,p)//B3LYP/6-31G(d)

HF = -1325.1429776 hartrees (-831540.469873776 kcal/mol)

Coordinates (from last standard orientation):

Center Atomic Coordinates (Angstroms)

Number Number X Y Z

1	6	-1.521175	-1.265123	-0.530189
2	6	-2.292501	0.082822	-0.561601
3	6	-3.554983	-0.002420	0.389883
4	6	-4.453565	-1.296330	0.293949
5	6	-3.532241	-2.544183	0.298619
6	6	-2.427033	-2.481952	-0.758278
7	1	-2.871677	-2.482467	-1.758946
8	6	-1.422266	1.287129	0.003632
9	6	-2.183882	2.634455	-0.074978
10	6	-3.510635	2.517827	0.713455
11	1	-4.074792	3.443887	0.554395
12	6	-4.362538	1.307185	0.326351
13	6	-0.776743	1.052048	1.391807
14	1	-1.326531	1.657176	2.117138
15	1	-0.932910	0.015613	1.695364
16	6	0.729899	1.352480	1.541676
17	1	1.022720	1.003215	2.534909
18	1	0.914698	2.430437	1.531642
19	6	1.541161	0.626678	0.456241
20	1	1.067200	-0.355806	0.330653
21	6	1.348418	1.331687	-0.890646
22	6	1.819555	0.451799	-2.040345
23	1	1.220171	-0.466702	-2.075625
24	6	1.814780	2.773170	-1.058878
25	1	2.887963	2.797507	-1.250960
26	1	1.319028	3.218600	-1.926672
27	1	1.616645	3.397483	-0.187620
28	6	3.050134	0.273602	0.751781

29	6	3.506149	-0.637059	-0.451922
30	1	2.781889	-1.469538	-0.440030
31	6	3.298692	0.058552	-1.812608
32	1	3.582776	-0.612066	-2.627585
33	1	3.939196	0.941733	-1.912700
34	1	1.706914	0.969553	-3.001916
35	6	3.098739	-0.591835	2.041256
36	1	2.863335	0.022762	2.918530
37	1	2.320769	-1.368005	1.981639
38	6	4.888544	-1.362631	-0.298119
39	6	4.868306	-2.125919	1.051834
40	1	4.165078	-2.967183	0.961173
41	6	4.457582	-1.274500	2.256290
42	1	5.229767	-0.526848	2.474127
43	1	4.398662	-1.906323	3.150523
44	6	6.115328	-0.429109	-0.381968
45	1	7.031031	-1.030864	-0.402162
46	1	6.107925	0.171663	-1.298893
47	1	6.199417	0.255176	0.464646
48	6	5.037861	-2.421572	-1.415308
49	1	5.231049	-1.974258	-2.396255
50	1	5.886913	-3.077249	-1.192271
51	1	4.145665	-3.055160	-1.496593
52	6	3.901833	1.545981	0.985021
53	1	4.767723	1.332940	1.613617
54	1	4.288709	1.991743	0.066325
55	1	3.322054	2.313255	1.509775
56	1	-1.815778	-3.392135	-0.696673

57	8	-0.233824	1.469165	-1.033213
58	1	-0.527935	1.154725	-1.908172
59	1	5.855776	-2.571058	1.226897
60	6	-2.693372	0.344820	-2.039302
61	1	-1.829405	0.338008	-2.724997
62	1	-3.328072	-0.459417	-2.405730
63	1	-3.236381	1.275538	-2.202031
64	8	-4.356032	-3.689151	0.117138
65	1	-3.822333	-4.481462	0.282554
66	1	-3.046256	-2.586019	1.291019
67	1	-1.028978	-1.405944	0.437389
68	1	-0.722410	-1.259144	-1.283225
69	1	-4.784942	1.453611	-0.673781
70	1	-5.216275	1.249758	1.007337
71	1	-3.137090	-0.056385	1.405947
72	6	-5.326205	-1.372062	1.571799
73	1	-5.806002	-2.352485	1.622911
74	1	-4.725373	-1.241775	2.480850
75	1	-6.116697	-0.615570	1.576476
76	6	-5.416548	-1.334847	-0.914398
77	1	-6.022124	-0.424877	-0.965997
78	1	-4.917854	-1.463054	-1.878461
79	1	-6.098302	-2.180958	-0.803526
80	1	-3.290764	2.482592	1.789249
81	1	-2.439810	2.801641	-1.127444
82	6	-1.400463	3.869072	0.391544
83	1	-2.032570	4.754997	0.272774
84	1	-1.122346	3.815173	1.449419

85	1	-0.497591	4.035256	-0.200006

Figure S7

A6

B3LYP/6-31G(d)//B3LYP/6-31G(d)

HF = -1325.294389 hartrees (-831635.48204139 kcal/mol)

Imaginary Frequencies: none found

Zero-point correction = 0.789027 (Hartree/Particle)

mPW1PW91/6-31+G(d,p)//B3LYP/6-31G(d)

HF = -1325.1254244 hartrees (-831529.455065244 kcal/mol)

Coordinates (from last standard orientation):

Center Number	Atomic Number	Coordinates (Angstroms) X	Y	Z
1	6	-3.019086	-1.644931	-1.359856
2	6	-3.111126	-0.107744	-1.242373
3	6	-3.307490	0.283008	0.261438
4	6	-4.443081	-0.466502	1.057736
5	6	-4.286795	-1.990850	0.792216
6	6	-4.208509	-2.348758	-0.696096
7	1	-5.154948	-2.100228	-1.187814
8	6	-1.807396	0.564191	-1.809026
9	6	-1.673409	1.989701	-1.496958
10	6	-2.184930	2.530700	-0.245605

11	1	-2.306914	3.619923	-0.299241
12	6	-3.371842	1.811950	0.418092
13	6	-0.360573	-0.082886	-1.494128
14	1	-0.455570	-1.102364	-1.875030
15	1	0.339358	0.419769	-2.166950
16	6	0.190740	-0.071832	-0.050619
17	1	0.123557	-1.085460	0.355867
18	1	-0.451421	0.528962	0.593795
19	6	1.646781	0.475645	0.070599
20	1	1.856276	1.014618	-0.863823
21	6	1.755953	1.597057	1.143680
22	6	3.169707	2.201450	1.115428
23	1	3.268362	2.763865	0.177521
24	6	1.334864	1.221244	2.570502
25	1	2.055073	0.559153	3.053594
26	1	1.271178	2.129986	3.182110
27	1	0.354613	0.735217	2.599675
28	6	2.764653	-0.633856	0.149030
29	6	4.144003	0.117649	0.053559
30	1	4.063659	0.706409	-0.876109
31	6	4.287682	1.151573	1.185269
32	1	5.246756	1.671011	1.103755
33	1	4.293122	0.666253	2.168363
34	1	3.273770	2.927446	1.934484
35	6	2.614967	-1.514680	1.417347
36	1	3.011609	-2.518471	1.255002
37	1	3.135978	-1.109504	2.287025
38	1	1.564566	-1.642036	1.700531

39	1	-4.085605	-3.435010	-0.798583
40	8	0.817559	2.623844	0.669557
41	1	0.956523	3.405847	1.230234
42	6	-4.234772	0.413748	-2.174167
43	1	-4.022223	0.136592	-3.213797
44	1	-5.212234	0.004185	-1.925061
45	1	-4.325271	1.507434	-2.143976
46	8	-5.374745	-2.646135	1.431278
47	1	-5.232265	-3.603490	1.372976
48	1	-3.335245	-2.303999	1.260883
49	1	-2.105907	-1.997021	-0.862494
50	1	-2.936377	-1.939121	-2.414662
51	1	-4.294394	2.207928	-0.018421
52	1	-3.388119	2.092498	1.474130
53	1	-2.387036	-0.047308	0.760954
54	6	-4.212495	-0.237034	2.571350
55	1	-4.851719	-0.914980	3.142194
56	1	-3.171459	-0.434609	2.858126
57	1	-4.461072	0.784852	2.877037
58	6	-5.884522	-0.008110	0.742029
59	1	-5.993806	1.075974	0.850391
60	1	-6.226784	-0.283146	-0.257909
61	1	-6.571295	-0.480117	1.447843
62	1	-1.250319	2.413150	0.371774
63	1	-1.828950	0.477201	-2.905114
64	6	-0.885291	2.881386	-2.377325
65	1	-1.527548	3.708842	-2.712614
66	1	-0.083868	3.341450	-1.780463

67	1	-0.462968	2.376606	-3.247533
68	6	2.624363	-1.541213	-1.103534
69	1	1.703645	-2.137105	-1.036401
70	1	2.523457	-0.903482	-1.995096
71	6	5.413467	-0.773340	-0.187277
72	6	5.134733	-1.690229	-1.405822
73	1	5.106240	-1.063750	-2.310114
74	6	3.822804	-2.475941	-1.320151
75	1	3.876829	-3.223272	-0.519198
76	1	3.671910	-3.044357	-2.246119
77	6	5.848593	-1.610672	1.034611
78	1	6.806973	-2.098557	0.821843
79	1	5.997454	-0.984439	1.921570
80	1	5.138237	-2.397401	1.297055
81	6	6.605544	0.135344	-0.568600
82	1	6.979501	0.714892	0.281789
83	1	7.439828	-0.476289	-0.930920
84	1	6.338961	0.837181	-1.368671
85	1	5.978154	-2.379807	-1.539189

TS (A6-B5)

B3LYP/6-31G(d)//B3LYP/6-31G(d)

HF = -1325.2867663 hartrees (-831630.698720913 kcal/mol)

Imaginary Frequencies: 1 (-45.5886 1/cm)

Zero-point correction = 0.788399 (Hartree/Particle)

mPW1PW91/6-31+G(d,p)//B3LYP/6-31G(d)

HF = -1325.1183373 hartrees (-831525.007839123 kcal/mol)

Coordinates (from last standard orientation):

Center Number	Atomic Number	Coordinates (Angstroms) X	Y	Z
1	6	-2.900444	-1.349194	-1.583287
2	6	-3.023787	0.167586	-1.291685
3	6	-3.330725	0.400465	0.233238
4	6	-4.420632	-0.512899	0.914466
5	6	-4.180313	-1.984276	0.478543
6	6	-4.086081	-2.151906	-1.039481
7	1	-5.032082	-1.864005	-1.510085
8	6	-1.682669	0.920943	-1.657827
9	6	-1.508085	2.234464	-1.020257
10	6	-2.468664	2.793452	-0.050923
11	1	-2.873221	3.701669	-0.533706
12	6	-3.588876	1.892875	0.489065
13	6	-0.338594	0.083771	-1.776245
14	1	-0.562787	-0.698221	-2.503801
15	1	0.412375	0.716292	-2.256883
16	6	0.238046	-0.535628	-0.478224
17	1	0.434296	-1.591590	-0.685314
18	1	-0.527580	-0.533778	0.297917
19	6	1.530345	0.142963	0.067946
20	1	1.698010	1.045676	-0.535659
21	6	1.328305	0.705685	1.501810

22	6	2.580916	1.487373	1.934022
23	1	2.630994	2.398717	1.323223
24	6	0.909198	-0.300990	2.582515
25	1	1.722208	-0.974401	2.856849
26	1	0.624225	0.242040	3.493272
27	1	0.052174	-0.909221	2.279127
28	6	2.849297	-0.700048	-0.137013
29	6	4.045459	0.226821	0.296649
30	1	3.916902	1.133777	-0.319226
31	6	3.882830	0.694837	1.754376
32	1	4.717756	1.341121	2.039891
33	1	3.910036	-0.153033	2.449032
34	1	2.468926	1.812832	2.977716
35	6	2.790094	-2.050870	0.622573
36	1	3.390503	-2.814490	0.124620
37	1	3.157969	-1.984566	1.648597
38	1	1.768947	-2.444168	0.668302
39	1	-3.935608	-3.213221	-1.277846
40	8	0.223186	1.656248	1.340522
41	1	0.037261	2.012038	2.225894
42	6	-4.105105	0.758461	-2.233236
43	1	-3.840935	0.555980	-3.278693
44	1	-5.089634	0.324010	-2.061165
45	1	-4.208988	1.845070	-2.132135
46	8	-5.225868	-2.770576	1.035749
47	1	-5.033564	-3.704210	0.858520
48	1	-3.210638	-2.297197	0.908962
49	1	-1.995902	-1.750668	-1.113765

50	1	-2.792175	-1.512528	-2.663070
51	1	-4.537025	2.194365	0.037457
52	1	-3.691590	2.082176	1.560566
53	1	-2.393030	0.155938	0.754142
54	6	-4.226314	-0.450391	2.448944
55	1	-4.864095	-1.197660	2.927176
56	1	-3.187892	-0.663917	2.733518
57	1	-4.497816	0.527316	2.860900
58	6	-5.881073	-0.101126	0.619987
59	1	-6.083423	0.919199	0.960541
60	1	-6.151772	-0.162351	-0.436728
61	1	-6.555267	-0.768187	1.160798
62	1	-1.845118	3.216138	0.752910
63	1	-1.789454	1.260728	-2.712777
64	6	-0.371837	3.108802	-1.379663
65	1	-0.644554	4.168012	-1.335875
66	1	0.367522	2.942169	-0.576625
67	1	0.092449	2.868357	-2.337016
68	6	2.999658	-0.987627	-1.656537
69	1	2.220272	-1.684377	-1.992310
70	1	2.842995	-0.049335	-2.211402
71	6	5.488083	-0.264117	-0.079150
72	6	5.502757	-0.601805	-1.592210
73	1	5.417313	0.338619	-2.157362
74	6	4.378990	-1.540896	-2.039903
75	1	4.524419	-2.540568	-1.612059
76	1	4.421709	-1.675550	-3.127721
77	6	5.991783	-1.466086	0.748914

78	1	7.047654	-1.651657	0.519928
79	1	5.925933	-1.271999	1.825470
80	1	5.450678	-2.392274	0.543281
81	6	6.490515	0.893775	0.136024
82	1	6.668151	1.103439	1.196079
83	1	7.459556	0.634427	-0.305641
84	1	6.147489	1.820223	-0.341492
85	1	6.478429	-1.029001	-1.857085

B5

B3LYP/6-31G(d)//B3LYP/6-31G(d)

HF = -1325.2936448 hartrees (-831635.015048448 kcal/mol)

Imaginary Frequencies: none found

Zero-point correction = 0.793242 (Hartree/Particle)

mPW1PW91/6-31+G(d,p)//B3LYP/6-31G(d)

HF = -1325.1247813 hartrees (-831529.051513563 kcal/mol)

Coordinates (from last standard orientation):

Center Number	Atomic Number	Coordinates (Angstroms) X	Y	Z
1	6	2.573994	-1.630491	0.597848
2	6	2.820153	-0.180470	1.088409
3	6	3.475928	0.582417	-0.114755
4	6	4.783881	-0.022840	-0.734167

5	6	4.487902	-1.508827	-1.075076
6	6	3.857698	-2.297297	0.076960
7	1	4.596090	-2.430954	0.873596
8	6	1.497889	0.596604	1.578759
9	6	1.001753	1.779916	0.731494
10	6	2.137813	2.690462	0.193616
11	1	2.124433	3.609968	0.788397
12	6	3.545590	2.080034	0.198783
13	6	0.361885	-0.309759	2.129584
14	1	0.840004	-1.058703	2.769961
15	1	-0.250782	0.288990	2.815522
16	6	-0.601995	-1.060358	1.179025
17	1	-1.167664	-1.740181	1.820269
18	1	-0.050550	-1.706649	0.496158
19	6	-1.604873	-0.170254	0.409120
20	1	-1.808370	0.702317	1.034294
21	6	-1.048124	0.388447	-0.909126
22	6	-1.953664	1.458925	-1.500689
23	1	-2.023823	2.305702	-0.808295
24	6	-0.538771	-0.586567	-1.962642
25	1	-1.380330	-1.071212	-2.459063
26	1	0.021930	-0.050989	-2.736371
27	1	0.104863	-1.361535	-1.546958
28	6	-3.064758	-0.791726	0.210829
29	6	-3.956661	0.351244	-0.406663
30	1	-3.860440	1.180653	0.314945
31	6	-3.368683	0.888139	-1.722891
32	1	-3.988333	1.697703	-2.116349

33	1	-3.347872	0.117714	-2.501681
34	1	-1.541239	1.832410	-2.448261
35	6	-3.017517	-2.105313	-0.608134
36	1	-3.845350	-2.765201	-0.344173
37	1	-3.079404	-1.951605	-1.687990
38	1	-2.099178	-2.664920	-0.401293
39	1	3.611551	-3.307958	-0.277564
40	8	0.352091	1.156918	-0.563664
41	1	0.517480	1.765303	-1.307712
42	6	3.695749	-0.248223	2.367713
43	1	3.096047	-0.626195	3.204852
44	1	4.550261	-0.914626	2.269374
45	1	4.078496	0.734692	2.661646
46	8	5.705950	-2.102289	-1.508659
47	1	5.516438	-3.004493	-1.809325
48	1	3.760005	-1.502277	-1.908670
49	1	1.850663	-1.619292	-0.226304
50	1	2.143664	-2.249646	1.392637
51	1	4.016023	2.249889	1.173013
52	1	4.157031	2.620122	-0.528216
53	1	2.745100	0.477515	-0.933234
54	6	5.078171	0.693431	-2.075991
55	1	5.826713	0.123132	-2.632071
56	1	4.179310	0.770206	-2.702466
57	1	5.477404	1.702289	-1.929009
58	6	6.048516	0.120303	0.140068
59	1	6.170179	1.153910	0.483535
60	1	6.053888	-0.527758	1.017612

61	1	6.930245	-0.140005	-0.450089
62	1	1.879025	3.022624	-0.824430
63	1	1.839405	1.143877	2.469255
64	6	-0.035321	2.675109	1.414291
65	1	0.439793	3.137714	2.285355
66	1	-0.342543	3.486313	0.745614
67	1	-0.923126	2.155989	1.767128
68	6	-3.637671	-1.110118	1.622175
69	1	-3.109130	-1.959501	2.068976
70	1	-3.462277	-0.247708	2.282794
71	6	-5.504984	0.093992	-0.451566
72	6	-5.958229	-0.292244	0.978666
73	1	-5.879905	0.598855	1.619251
74	6	-5.141886	-1.421096	1.611306
75	1	-5.331546	-2.366748	1.089405
76	1	-5.473476	-1.582922	2.643817
77	6	-5.948065	-0.974757	-1.473673
78	1	-7.042174	-0.988029	-1.535627
79	1	-5.573771	-0.755132	-2.480335
80	1	-5.629156	-1.985662	-1.211802
81	6	-6.229952	1.410436	-0.817333
82	1	-6.108654	1.680158	-1.871875
83	1	-7.305509	1.301543	-0.638946
84	1	-5.878866	2.250772	-0.205328
85	1	-7.021516	-0.562220	0.958990

TS (B5-[C]-D4)

B3LYP/6-31G(d)//B3LYP/6-31G(d)

HF = -1325.2809765 hartrees (-831627.065563515 kcal/mol)

Imaginary Frequencies: 1 (-370.9670 1/cm)

Zero-point correction = 0.787814 (Hartree/Particle)

mPW1PW91/6-31+G(d,p)//B3LYP/6-31G(d)

HF = -1325.1147309 hartrees (-831522.744787059 kcal/mol)

Coordinates (from last standard orientation):

Center Number	Atomic Number	Coordinates (Angstroms) X	Y	Z
1	6	2.628872	-0.817782	1.422067
2	6	2.841014	0.617723	0.868700
3	6	3.597422	0.474176	-0.497210
4	6	4.935743	-0.344512	-0.494938
5	6	4.652675	-1.714294	0.179041
6	6	3.949083	-1.593794	1.533091
7	1	4.629880	-1.140241	2.260889
8	6	1.456588	1.339188	0.634989
9	6	1.234173	2.129146	-0.528459
10	6	2.302002	2.326797	-1.567264
11	1	2.303071	3.372024	-1.896961
12	6	3.698757	1.833594	-1.198620
13	6	0.320308	1.068373	1.634902
14	1	0.786127	0.957765	2.618619
15	1	-0.321437	1.950477	1.716206

16	6	-0.583694	-0.176975	1.404744
17	1	-1.119068	-0.291803	2.351073
18	1	0.040567	-1.064068	1.323370
19	6	-1.605516	-0.121698	0.234095
20	1	-1.607960	0.902801	-0.152134
21	6	-1.150445	-0.972377	-0.989449
22	6	-2.136564	-0.778291	-2.152757
23	1	-2.034151	0.254837	-2.512769
24	6	-0.907026	-2.468429	-0.738484
25	1	-1.834205	-3.019165	-0.575002
26	1	-0.427772	-2.906481	-1.623767
27	1	-0.249606	-2.652959	0.115347
28	6	-3.106691	-0.352653	0.681824
29	6	-4.004688	-0.103731	-0.588470
30	1	-3.730723	0.916068	-0.911112
31	6	-3.596829	-1.022419	-1.754012
32	1	-4.227394	-0.830145	-2.626718
33	1	-3.750689	-2.077974	-1.500571
34	1	-1.849928	-1.432202	-2.988573
35	6	-3.290576	-1.748082	1.332825
36	1	-4.109807	-1.750851	2.054312
37	1	-3.508660	-2.535093	0.608020
38	1	-2.393841	-2.052316	1.883582
39	1	3.727222	-2.599636	1.913714
40	8	0.134134	-0.381038	-1.369226
41	1	0.453531	-0.886769	-2.135007
42	6	3.565604	1.474529	1.943671
43	1	2.898460	1.675593	2.790118

44	1	4.439216	0.965745	2.346700
45	1	3.903689	2.439479	1.550927
46	8	5.893459	-2.397920	0.296543
47	1	5.718829	-3.305337	0.590423
48	1	3.979729	-2.271290	-0.498763
49	1	1.967998	-1.360465	0.737562
50	1	2.140146	-0.794961	2.401636
51	1	4.203340	2.574647	-0.568711
52	1	4.288084	1.764857	-2.115671
53	1	2.909741	-0.131254	-1.109298
54	6	5.339112	-0.639097	-1.960732
55	1	6.113978	-1.409657	-1.969503
56	1	4.489546	-1.007609	-2.550246
57	1	5.748157	0.241800	-2.465710
58	6	6.128631	0.372854	0.173998
59	1	6.252391	1.385262	-0.225984
60	1	6.045411	0.447180	1.260114
61	1	7.047233	-0.180319	-0.033634
62	1	1.913451	1.750243	-2.422740
63	1	1.724327	2.539134	0.770821
64	6	-0.034661	2.881856	-0.814974
65	1	0.190847	3.892707	-1.166839
66	1	-0.526271	2.347976	-1.636365
67	1	-0.734239	2.932810	0.016621
68	6	-3.472322	0.739651	1.727587
69	1	-2.925159	0.579075	2.665063
70	1	-3.151214	1.720218	1.340920
71	6	-5.553125	-0.003098	-0.348975

72	6	-5.799317	1.043665	0.767017
73	1	-5.556057	2.039675	0.366876
74	6	-4.974266	0.815655	2.035462
75	1	-5.305207	-0.096631	2.546019
76	1	-5.152570	1.632927	2.745100
77	6	-6.234271	-1.343682	0.002848
78	1	-7.322023	-1.208261	0.020583
79	1	-6.018714	-2.115118	-0.745121
80	1	-5.942895	-1.736412	0.979168
81	6	-6.233941	0.534706	-1.629009
82	1	-6.261786	-0.207269	-2.433708
83	1	-7.272443	0.808735	-1.410518
84	1	-5.729204	1.431613	-2.009571
85	1	-6.869003	1.066302	1.011615

D4

B3LYP/6-31G(d)//B3LYP/6-31G(d)

HF = -1325.3103885 hartrees (-831645.521887635 kcal/mol)

Imaginary Frequencies: none found

Zero-point correction = 0.792922 (Hartree/Particle)

mPW1PW91/6-31+G(d,p)//B3LYP/6-31G(d)

HF = -1325.1424285 hartrees (-831540.125308035 kcal/mol)

Coordinates (from last standard orientation):

Center Number	Atomic Number	Coordinates (Angstroms) X	Y	Z
1	6	2.534710	-1.146382	1.241613
2	6	2.652369	0.344169	0.832313
3	6	3.407585	0.449893	-0.551019
4	6	4.746461	-0.370375	-0.705844
5	6	4.480144	-1.823441	-0.230552
6	6	3.868693	-1.900872	1.169662
7	1	4.588898	-1.527255	1.904735
8	6	1.217628	1.019271	0.713920
9	6	1.323033	2.490907	0.233348
10	6	2.166055	2.618687	-1.053975
11	1	2.311671	3.683893	-1.265201
12	6	3.524759	1.914039	-1.005046
13	6	0.326026	0.831151	1.969457
14	1	0.966872	0.570365	2.812800
15	1	-0.112581	1.799218	2.222511
16	6	-0.819394	-0.200492	1.907017
17	1	-1.425108	-0.050408	2.804000
18	1	-0.424722	-1.218579	1.976422
19	6	-1.676482	-0.009788	0.646295
20	1	-1.771536	1.074526	0.503489
21	6	-0.904123	-0.498478	-0.584300
22	6	-1.563397	-0.026226	-1.874053
23	1	-1.529010	1.067675	-1.916924
24	6	-0.491470	-1.963725	-0.672515
25	1	-1.339366	-2.560267	-1.011504

26	1	0.295879	-2.092247	-1.425700
27	1	-0.144061	-2.382926	0.271479
28	6	-3.171448	-0.515014	0.675225
29	6	-3.814985	0.009720	-0.664300
30	1	-3.644448	1.098994	-0.629086
31	6	-3.039644	-0.483850	-1.901970
32	1	-3.485674	-0.079282	-2.814187
33	1	-3.095690	-1.574237	-1.999584
34	1	-1.025441	-0.409668	-2.750260
35	6	-3.252439	-2.046261	0.896129
36	1	-4.194422	-2.329292	1.368210
37	1	-3.182638	-2.628332	-0.025506
38	1	-2.457286	-2.387370	1.568398
39	1	3.691123	-2.953288	1.429063
40	8	0.459667	0.310093	-0.491043
41	1	1.123576	-0.058659	-1.105444
42	6	3.409564	1.104674	1.959920
43	1	2.777224	1.283576	2.833052
44	1	4.254198	0.512650	2.309431
45	1	3.803460	2.069670	1.639796
46	8	5.710599	-2.530396	-0.300933
47	1	5.540854	-3.465368	-0.108017
48	1	3.754581	-2.266530	-0.940133
49	1	1.831406	-1.655625	0.573858
50	1	2.120384	-1.232103	2.252462
51	1	4.201022	2.461625	-0.341123
52	1	3.979829	1.958748	-1.998332
53	1	2.761512	-0.051611	-1.303746

54	6	5.112608	-0.441706	-2.208673
55	1	5.914030	-1.170826	-2.349338
56	1	4.258338	-0.760154	-2.820996
57	1	5.466812	0.519038	-2.593782
58	6	5.960536	0.237936	0.030342
59	1	6.132124	1.272294	-0.283829
60	1	5.869174	0.227966	1.118073
61	1	6.853984	-0.337820	-0.220986
62	1	1.577578	2.230064	-1.897807
63	1	1.859635	2.995285	1.048405
64	6	-0.005909	3.237880	0.037310
65	1	0.210122	4.277308	-0.229196
66	1	-0.590805	2.810715	-0.783849
67	1	-0.634485	3.265419	0.931551
68	6	-3.893274	0.189778	1.856834
69	1	-3.528916	-0.205065	2.813063
70	1	-3.642271	1.261162	1.841941
71	6	-5.374439	-0.106266	-0.784535
72	6	-5.994670	0.550656	0.475940
73	1	-5.830201	1.636780	0.415618
74	6	-5.420954	0.044113	1.801837
75	1	-5.711517	-1.000305	1.968214
76	1	-5.860242	0.608264	2.633090
77	6	-5.896226	-1.548412	-0.962922
78	1	-6.973054	-1.525137	-1.165469
79	1	-5.421952	-2.049325	-1.814890
80	1	-5.748677	-2.176674	-0.081931
81	6	-5.855698	0.712766	-2.004751

82	1	-5.603634	0.236626	-2.958418
83	1	-6.946629	0.811941	-1.977454
84	1	-5.433757	1.725542	-2.005321
85	1	-7.082020	0.405311	0.457928

----

8. Crystallographic Data

Alkene **13**

CCDC 1529115 contains the supplementary crystallographic data for this paper. These data can be obtained free of charge from The Cambridge Crystallographic Data Centre via www.ccdc.cam.ac.uk/structures

Cupacinoxepin **4**

CCDC 1529116 contains the supplementary crystallographic data for this paper. These data can be obtained free of charge from The Cambridge Crystallographic Data Centre via www.ccdc.cam.ac.uk/structures

Oxane **22**

CCDC 1529117 contains the supplementary crystallographic data for this paper. These data can be obtained free of charge from The Cambridge Crystallographic Data Centre via www.ccdc.cam.ac.uk/structures

Oxepane **10**

CCDC 1529118 contains the supplementary crystallographic data for this paper. These data can be obtained free of charge from The Cambridge Crystallographic Data Centre via www.ccdc.cam.ac.uk/structures

9. References

1. J. H. George, M. McArdle, J. E. Baldwin and R. M. Adlington, *Tetrahedron*, 2010, **66**, 6321-6330.
2. D. J. Clausen, S. Wan and P. E. Floreancig, *Angew. Chem. Int. Ed.*, 2011, **50**, 5178-5181.
3. V. Domingo, L. Lorenzo, J. F. Quilez del Moral and A. F. Barrero, *Org. Biomol. Chem.*, 2013, **11**, 559-562.
4. T. R. Hoye, C. S. Jeffrey and F. Shao, *Nat. Protoc.*, 2007, **2**, 2451-2458.
5. J. A. Soderquist and A. Negron, *Org. Synth.*, 1992, **70**, 169.
6. R. K. Boeckman, P. Shao and J. J. Mullins, *Org. Synth.*, 2000, **77**, 141.
7. M. S. Gachet, O. Kunert, M. Kaiser, R. Brun, M. Zehl, W. Keller, R. A. Muñoz, R. Bauer and W. Schuehly, *J. Nat. Prod.*, 2011, **74**, 559-566.
8. H. Ageta, K. Shiojima and K. Masuda, *Chem. Pharm. Bull.*, 1982, **30**, 2272-2274.
9. D. Ueda, T. Hoshino and T. Sato, *J. Am. Chem. Soc.*, 2013, **135**, 18335-18338.
10. Y. Arai, M. Hirohara, H. Ageta and H. Y. Hsű, *Tetrahedron Lett.*, 1992, **33**, 1325-1328.
11. M. Kinoshita, D. Nakamura, N. Fujiwara and H. Akita, *J. Mol. Catal. B: Enzym.*, 2003, **22**, 161-172.
12. T. Sato, H. Hoshino, S. Yoshida, M. Nakajima and T. Hoshino, *J. Am. Chem. Soc.*, 2011, **133**, 17540-17543.
13. M. Nishizawa, H. Takao, N. Kanoh, K. Asoh, S. Hatakeyama and H. Yamada, *Tetrahedron Lett.*, 1994, **35**, 5693-5696.
14. D. H. R. Barton and K. H. Overton, *J. Chem. Soc. (Resumed)*, 1955, 2639-2652.
15. S. Berger and D. Sicker, *Classics in Spectroscopy: Isolation and Structure Elucidation of Natural Products*, Wiley-VCH, Weinheim, 2009.
16. K. K. W. Kuan, H. P. Pepper, W. M. Bloch and J. H. George, *Org. Lett.*, 2012, **14**, 4710-4713.
17. J.-P. Zahra, F. Chauvet, I. Coste-Manière, P. Martres, P. Perfetti and B. Waegell, *Bull. Soc. Chim. Fr.*, 1997, **134**, 1001-1024.
18. M. Matsushita, Y. Nagaoka, H. Hioki, Y. Fukuyama and M. Kodama, *Chem. Lett.*, 1996, **25**, 1039-1040.
19. J. Deng, S. Zhou, W. Zhang, J. Li, R. Li and A. Li, *J. Am. Chem. Soc.*, 2014, **136**, 8185-8188.
20. S. Nakamura, K. Ishihara and H. Yamamoto, *J. Am. Chem. Soc.*, 2000, **122**, 8131-8140.
21. C. A. Mullen and M. R. Gagné, *J. Am. Chem. Soc.*, 2007, **129**, 11880-11881.
22. R. L. Snowden, J. C. Eichenberger, S. M. Linder, P. Sonnay, C. Vial and K. H. Schulte-Elte, *J. Org. Chem.*, 1992, **57**, 955-960.
23. K. B. Sharpless, *J. Am. Chem. Soc.*, 1970, **92**, 6999-7001.
24. A. F. Barrero, J. Altarejos, E. J. Alvarez-Manzaneda, J. M. Ramos and S. Salido, *J. Org. Chem.*, 1996, **61**, 2215-2218.
25. M. Uyanik, H. Ishibashi, K. Ishihara and H. Yamamoto, *Org. Lett.*, 2005, **7**, 1601-1604.
26. P. F. Vlad, N. D. Ungur and V. B. Perutskii, *Chemistry of Heterocyclic Compounds*, 1990, **26**, 746-750.
27. A. S. Gopalan, R. Prieto, B. Mueller and D. Peters, *Tetrahedron Lett.*, 1992, **33**, 1679-1682.
28. S. Murata and T. Suzuki, *Tetrahedron Lett.*, 1990, **31**, 6535-6538.
29. T. R. Hoye, A. J. Caruso, J. F. Dellaria and M. J. Kurth, *J. Am. Chem. Soc.*, 1982, **104**, 6704-6709.
30. H.-M. Shieh and G. D. Prestwich, *Tetrahedron Lett.*, 1982, **23**, 4643-4646.
31. J. D. White, T. Nishiguchi and R. W. Skeean, *J. Am. Chem. Soc.*, 1982, **104**, 3923-3928.
32. T. Abe and T. Hoshino, *Org. Biomol. Chem.*, 2005, **3**, 3127-3139.

33. H.-C. Carmen, A.-C. Miriam, M.-D. Manuel and R.-G. Ignacio, *Curr. Org. Chem.*, 2014, **18**, 525-546.
34. M. Göhl and K. Seifert, *Eur. J. Org. Chem.*, 2014, 6975-6982.
35. Gaussian 09, Revision B.01, M. J. Frisch, G. W. Trucks, H. B. Schlegel, G. E. Scuseria, M. A. Robb, J. R. Cheeseman, G. Scalmani, V. Barone, B. Mennucci, G. A. Petersson, H. Nakatsuji, M. Caricato, X. Li, H. P. Hratchian, A. F. Izmaylov, J. Bloino, G. Zheng, J. L. Sonnenberg, M. Hada, M. Ehara, K. Toyota, R. Fukuda, J. Hasegawa, M. Ishida, T. Nakajima, Y. Honda, O. Kitao, H. Nakai, T. Vreven, J. A. Montgomery, Jr., J. E. Peralta, F. Ogliaro, M. Bearpark, J. J. Heyd, E. Brothers, K. N. Kudin, V. N. Staroverov, T. Keith, R. Kobayashi, J. Normand, K. Raghavachari, A. Rendell, J. C. Burant, S. S. Iyengar, J. Tomasi, M. Cossi, N. Rega, J. M. Millam, M. Klene, J. E. Knox, J. B. Cross, V. Bakken, C. Adamo, J. Jaramillo, R. Gomperts, R. E. Stratmann, O. Yazyev, A. J. Austin, R. Cammi, C. Pomelli, J. W. Ochterski, R. L. Martin, K. Morokuma, V. G. Zakrzewski, G. A. Voth, P. Salvador, J. J. Dannenberg, S. Dapprich, A. D. Daniels, O. Farkas, J. B. Foresman, J. V. Ortiz, J. Cioslowski,and D. J. Fox, Gaussian, Inc., Wallingford CT, 2010.
36. (a) A. D. Becke, *J. Chem. Phys.* 1993, **98**, 1372-1377. (b) A. D. Becke, *J. Chem. Phys.* 1993, **98**, 5648-5652. (c) C. Lee, W. Yang and R. G. Parr, *Phys. Rev. B* 1988, **37**, 785-789. (d) P. J. Stephens, F. J. Devlin, C. F. Chabalowski and M. J. Frisch, *J. Phys. Chem.* 1994, **98**, 11623-11627. (e) J. Tirado-Rives and W. L. Jorgensen, *J. Chem. Theory Comput.* 2008, **4**, 297-306.
37. (a) C. Gonzalez and H. B. Schlegel, *J. Phys. Chem.* 1990, **94**, 5523-5527. (b) K. Fukui, *Acc. Chem. Res.* 1981, **14**, 363-368.
38. S. P. T. Matsuda, W. K. Wilson and Q. Xiong, *Org. Biomol. Chem.* 2006, **4**, 530-543.
39. D. J. Tantillo, *Nat. Prod. Rep.* 2011, **28**, 1035-1053. This is part 8 of our series on theoretical studies of diterpene-forming carbodation rearrangements. For part 6 and 7, see refs. M. M. Vaughan, F. X. Webster, D. Kiemle, Y. J. Hong, D. J. Tantillo, Q. Wang, R. M. Coates, A. T. Wray, W. Askew, C. O'Donnell, J. G. Tokuhisa, W. Boland and D. Tholl, *Plant Cell*, 2013, **25**, 1108-1125Y and Y. J. Hong and D. J. Tantillo, *Nature Chem.* 2014, **6**, 104-111.
40. N. Muller and A. Falk, *Ball & Stick 4.0a12*, molecular graphics software for MacOS, Johannes Kepler University Linz, 2004.
